# Palladium-Catalyzed C–P Bond-Forming Reactions of Aryl Nonaflates Accelerated by Iodide

**DOI:** 10.1021/acs.joc.1c02172

**Published:** 2021-11-02

**Authors:** Holly McErlain, Leanne M. Riley, Andrew Sutherland

**Affiliations:** School of Chemistry, The Joseph Black Building, University of Glasgow, Glasgow G12 8QQ, United Kingdom

## Abstract

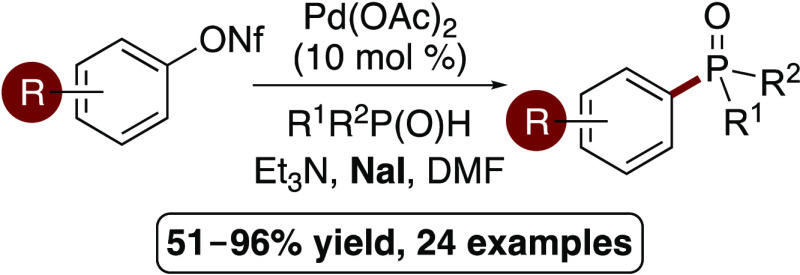

An iodide-accelerated, palladium-catalyzed C–P bond-forming reaction of aryl nonaflates is described. The protocol was optimized for the synthesis of aryl phosphine oxides and was found to be tolerant of a wide range of aryl nonaflates. The general nature of this transformation was established with coupling to other P(O)H compounds for the synthesis of aryl phosphonates and an aryl phosphinate. The straightforward synthesis of stable, isolable aryl nonaflates, in combination with the rapid C–P bond-forming reaction allows facile preparation of aryl phosphorus target compounds from readily available phenol starting materials. The synthetic utility of this general strategy was demonstrated with the efficient preparation of an organic light-emitting diode (OLED) material and a phosphonophenylalanine mimic.

## Introduction

**Scheme 1:**
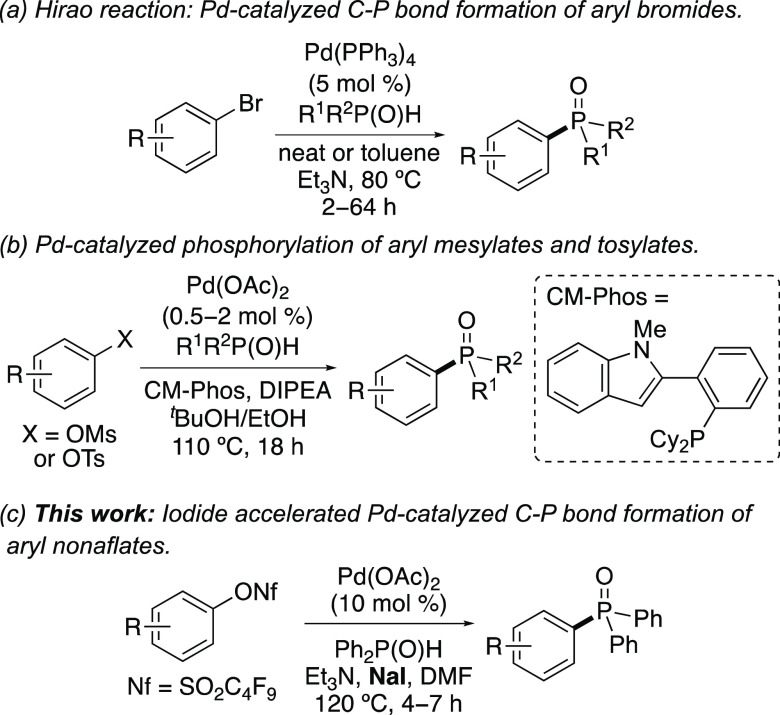
Selected Palladium-Catalyzed Reactions for the Synthesis of Aryl C–P Bonds

Aryl phosphorus compounds are important due to their widespread application in organic, medicinal, and materials chemistry.^1,2^ As a consequence, carbon–phosphorus bond formation is a highly active area of research in organophosphorus chemistry. Traditionally, aryl C–P bonds were formed via the reaction of Grignard or organolithium reagents with electrophilic phosphorus compounds.^3^ In 1981, pioneering work by Hirao and co-workers demonstrated that aryl C–P bonds could be generated by palladium-catalyzed cross-coupling reactions of aryl bromides with P(O)–H compounds (Scheme 1a).^4^ Since the discovery of the Hirao reaction, efforts have focused on extending the range of electrophilic aryl substrates, elucidation of the reaction mechanism and optimization of the reaction conditions.^5,6^

Despite the availability of aryl halides as coupling reagents, many complex arenes, particularly natural product-based (e.g., steroids and amino acids), exist only in phenolic form. For this reason, Hirao-type reactions using activated sulfonates have been reported. Aryl triflates have been explored as substrates,^5,6e,7^ but the high cost and reactive nature of reagents limits applications. This has led to the development of metal-catalyzed aryl C–P bond-forming reactions using mesylates and tosylates.^5,8^ For example, the Kwong group has demonstrated the effective phosphorylation of aryl mesylates and tosylates using low catalyst loadings of Pd(OAc)_2_ in combination with the CM-Phos ligand (Scheme 1b).^9^ Transformations at 110 °C and a reaction time of 18 h gave a wide range of phosphonate esters in high yields. Recently, the Ding and Xu groups independently reported Pd-catalyzed C–P bond-forming reactions of aryl fluorosulfonates.^10^ Following synthesis of these from phenols and sulfuryl fluoride gas, these compounds were readily coupled with a range of P(O)–H compounds using Pd(OAc)_2_ and either dppf or DPEPhos ligands.

Although advances in palladium-catalyzed C–P bond-forming reactions with aryl sulfonates have been achieved, we were interested in developing a method with a short reaction time, which avoided the need for additional ligands or gaseous reagents, and that could also be applied for the preparation of a range of aryl phosphorus compounds. Aryl nonafluorobutylsulfonates [nonaflates, ArOSO_2_(CF_2_)_3_CF_3_] are easily prepared from phenols and the inexpensive, industrial product nonaflyl fluoride. In addition, these are stable and can be readily purified by flash column chromatography. For these reasons, aryl nonaflates have been used for a wide range of palladium-catalyzed cross-coupling reactions.^11,12^ However, utilization of these for analogous C–P bond-forming reactions are relatively rare. Apart from a few specific examples,^13^ the only methodology study was reported by the Lipshutz group, who demonstrated the efficient synthesis of triarylphosphine boranes via the reaction of aryl nonaflates with diphenylphosphine-borane.^14^ Herein, we disclose a palladium-catalyzed C–P bond-forming reaction with aryl nonaflates that can be accelerated by iodide, resulting in short reaction times (Scheme 1c). The method does not require additional ligands or substrates prepared by gaseous reagents. Furthermore, we demonstrate that the method can be used as part of an effective strategy for the synthesis of important organophosphorus compounds from phenol starting materials.

## Results and Discussion

Initial studies focused on the reaction of diphenylphosphine oxide with the nonactivated starting material, *p*-tolyl nonaflate (**1a**) (Table 1). As previous work has shown palladium acetate as an effective catalyst for C–P bond formation,^6^ this was used in combination with triethylamine, originally utilized as a base by the Hirao group.^4^ In addition, dimethylformamide (DMF) was chosen as an effective solvent for working with aryl nonaflates. Using 1 equiv of diphenylphosphine oxide, at a reaction temperature of 90 °C, showed only 70% conversion by ^1^H nuclear magnetic resonance (NMR) spectroscopy, resulting in a 41% isolated yield (entry 1). Mechanistic work of palladium-catalyzed C–P bond-forming reactions by the Stawinski,^6a,6c^ Montchamp,^6f,6g^ and Keglevich groups^6h,6i^ have shown that excess amounts of the P(O)H coupling partner are required to reduce the palladium(II) catalyst and act as a ligand (see Scheme 6). Therefore, using 1.5 equiv of diphenylphosphine oxide and an increased reaction temperature of 110 °C, allowed full conversion after 24 h and a 58% isolated yield (entry 2). A further increase of reaction temperature to 120 °C resulted in further improvement in isolated yield (79%); however, the reaction still required 24 h to reach completion (entry 3). As ionic additives such as chloride and acetate ions are well known to promote Pd-mediated cross-coupling reactions,^15,16^ and facilitate the Hirao reaction,^6a,6c,6e^ these were investigated to improve the reaction time. Interestingly, the addition of stoichiometric quantities of NaOAc or NaCl (entries 4 and 5) led to no improvement in the reaction time and gave phosphine oxide **2a** in lower isolated yields. In contrast, the addition of NaI (1 equiv) resulted in a significantly faster reaction time of 4 h, which gave **2a** in 78% yield (entry 6). This effect was observed to a lesser extent using 0.1 equiv of NaI (entry 7). In this case, the reaction was complete after 8 h.

**Table 1:**
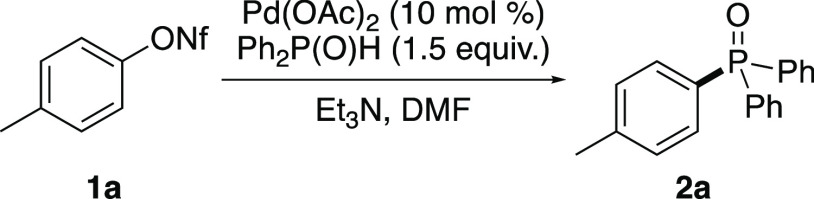
Optimization Studies for Palladium-Catalyzed Synthesis of (*p*-Tolyl)diphenylphosphine Oxide (**2a**)

entry	additive (equiv)	time (h)	temperature (°C)	isolated yield (%)
1^a^		24	90	41
2		24	110	58
3		24	120	79
4	NaOAc (1)	22	120	55
5	NaCl (1)	32	120	64
6	NaI (1)	4	120	78
7	NaI (0.1)	8	120	76

a Using 1 equiv of Ph_2_P(O)H.

**Scheme 2:**
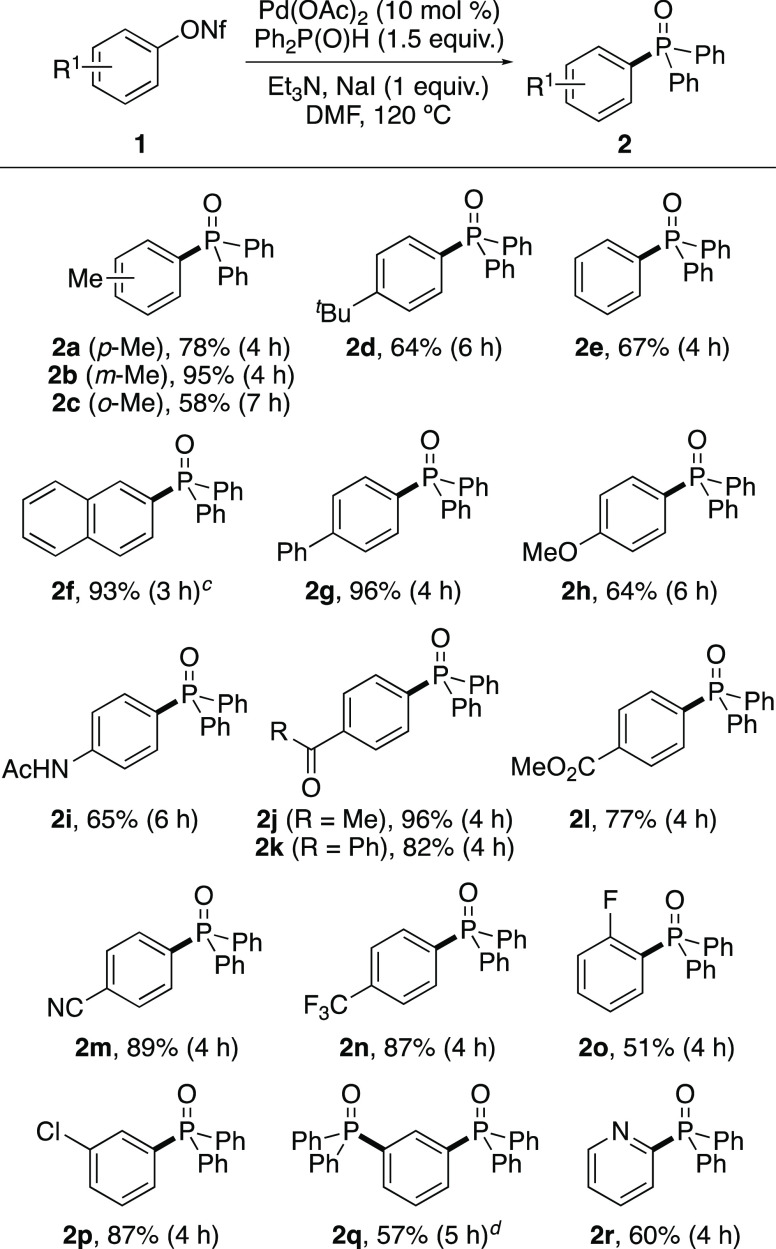
Reaction Scope of Aryl Nonaflates^,^ Isolated yields. Reactions performed at 0.2 or 0.4 mmol scale. Reaction performed at 90 °C. From 3-bromophenyl nonaflate using Ph_2_P(O)H (3 equiv).

Having identified rapid and efficient conditions for the synthesis of **2a**, the scope of the iodide-accelerated reaction was investigated for the coupling of diphenylphosphine oxide with various aryl nonaflates (Scheme 2). Using NaI (1 equiv) throughout, the process was found to be compatible with a wide range of substituents and functional groups, forming the majority of diphenylphosphine oxides after 4 h reaction times. Some variations to the standard conditions were observed. For example, the reaction of naphthyl analogue **1f** was found to proceed at 90 °C and was complete after 3 h, while aryl nonaflates with *ortho*-substituents (**1c**) or with strong electron-donating groups (**1h**) required slightly longer reaction times. Although the reaction conditions tolerated chloride substituents (**1p**), attempted coupling of 3-bromophenyl nonaflate (**1q**) with diphenylphosphine oxide (1.5 equiv) gave a mixture of compounds. Analysis of the reaction mixture by ^1^H NMR spectroscopy showed the presence of bis-phosphine oxide **2q** as the major product, along with mono-phosphine oxide by-products. As a selective reaction was not possible, **1q** was allowed to react with 3 equiv of diphenylphosphine oxide, which gave bis-phosphine oxide **2q** in 57% yield. Pyridin-2-yl nonaflate (**1r**) was also a substrate for this transformation, giving clean conversion to **2r** in 60% yield. From the series of nonaflates investigated, only a *p*-nitrophenyl analogue failed to generate the desired product. In this case, the reaction conditions led to decomposition of the nonaflate.

**Scheme 3:**
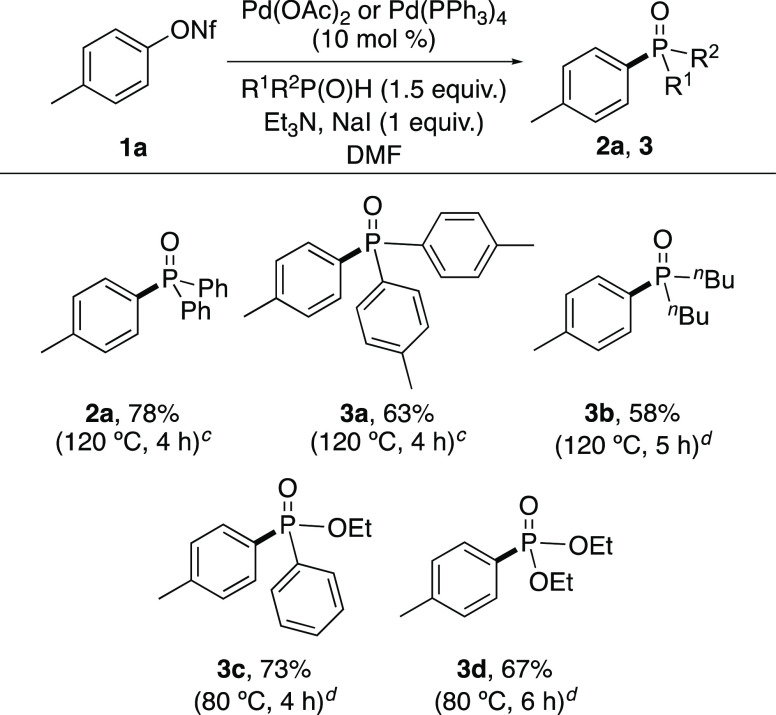
Reaction Scope for the Synthesis of Various Aryl Phosphorus Compounds^,^ Isolated yields. Reactions performed at 0.2 or 0.4 mmol scale. Reaction performed using Pd(OAc)_2_ (10 mol %). Reaction performed using Pd(PPh_3_)_4_ (10 mol %).

Using *p*-tolyl nonaflate (**1a**) as a standard substrate, the study then investigated the use of the reaction for the preparation of other aryl C–P bonds (Scheme 3). In a similar manner to the synthesis of diphenylphosphine oxide **2a**, the iodide-accelerated reaction with Pd(OAc)_2_ permitted the synthesis of phosphine oxide **3a**. While Pd(OAc)_2_ did allow the preparation of other aryl C–P-containing compounds, the reactions were less efficient, leading to the products in moderate yields (40–50%). For this reason, a brief screen for alternative catalysts was performed that identified Pd(PPh_3_)_4_ as an effective substitute.^17^ Reaction of **1a** with di-*n*-butylphosphine oxide in the presence of Pd(PPh_3_)_4_ and NaI gave dialkylphosphine oxide **3b** in 58% yield, after a reaction time of 5 h. Reaction of **1a** with the more reactive coupling partners, ethyl phenylphosphinate and diethyl phosphite was found to proceed at 80 °C and after reaction times of 4 and 6 h, respectively, gave phosphinate **3c** and phosphonate **3d** in good yields.

**Scheme 4:**
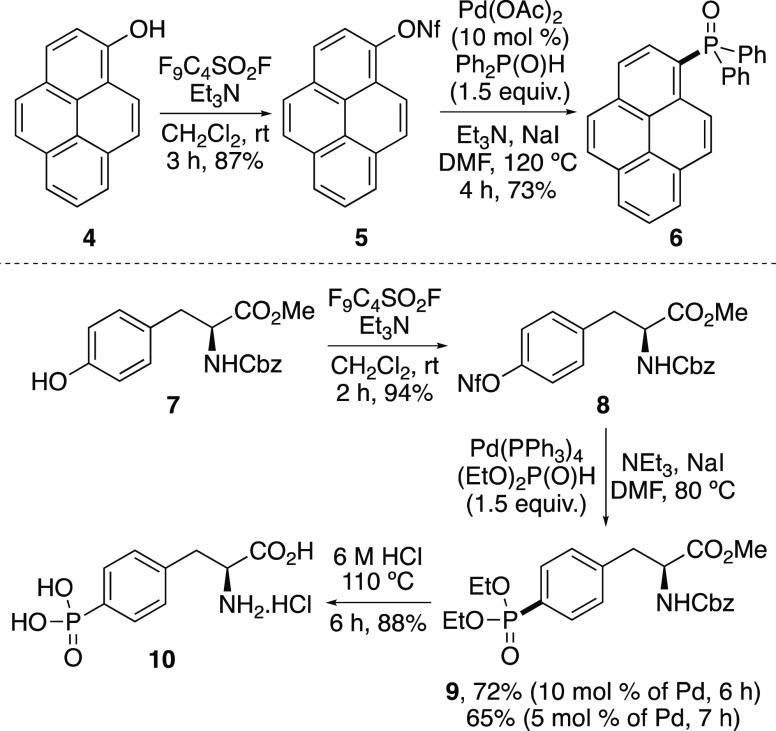
Synthesis of Various Aryl Phosphorus Target Compounds

The study next investigated the combination of the mild conditions for nonaflate synthesis with the accelerated aryl C–P bond-forming reaction for the simple conversion of phenols to aryl phosphorus-containing targets (Scheme 4). Pyrene nonaflate **5** was prepared in 87% yield by the treatment of 1-hydroxypyrene (**4**) with nonaflyl fluoride, under basic conditions. Reaction of **5** with diphenylphosphine oxide, using Pd(OAc)_2_ and NaI gave phosphine oxide **6**, a blue light-emitting diode material in 73% yield.^18^ In a similar manner, commercially available l-tyrosine derivative **7** was converted to the corresponding aryl nonaflate **8** under mild conditions, in 94% yield. Iodide-accelerated phosphorylation of **8**, performed at a 1 mmol scale, was found to proceed at 80 °C, and after a reaction time of 6 h, gave phosphonate ester **9** in 72% yield. With this transformation, a lower loading of the palladium catalyst was investigated. Using 5 mol % Pd(PPh_3_)_4_ showed no significant difference in reaction efficiency. Again, at a 1 mmol scale, the transformation was complete in 7 h and produced phosphonate ester in 65% yield. Acid-mediated deprotection allowed the isolation of phosphonophenylalanine **10**, a compound used for various medicinal chemistry applications, such as a component of peptides that act as thrombin inhibitors and as competitive *N*-methyl-d-aspartic acid antagonists.^7a,19^

**Figure 1:**
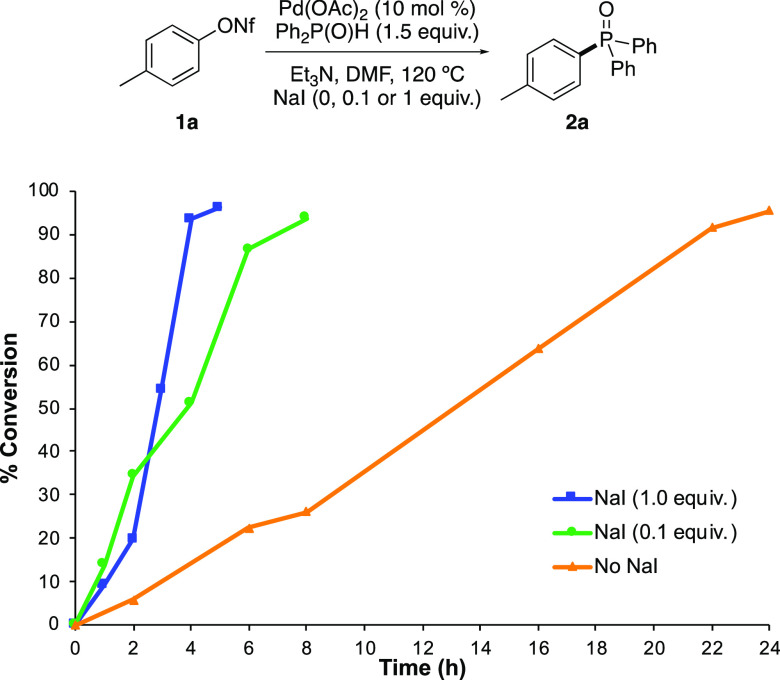
Conversion graph for the synthesis of **2a** (measured using ^1^H NMR spectroscopy and dimethyl terephthalate as an internal standard).

Having demonstrated the utility of this method, the possible role of iodide in accelerating the C–P bond-forming process was considered. Initially, the different rates observed during the reaction of *p*-tolyl nonaflate (**1a**) with diphenylphosphine oxide in the presence of NaI (0, 0.1, and 1 equiv) were further investigated. A conversion graph generated by ^1^H NMR spectroscopy confirmed that while the reaction with NaI (1 equiv) was complete after 4 h (∼95% conversion), only 12% conversion was observed at the same time during the reaction without NaI (Figure 1).

**Scheme 5:**
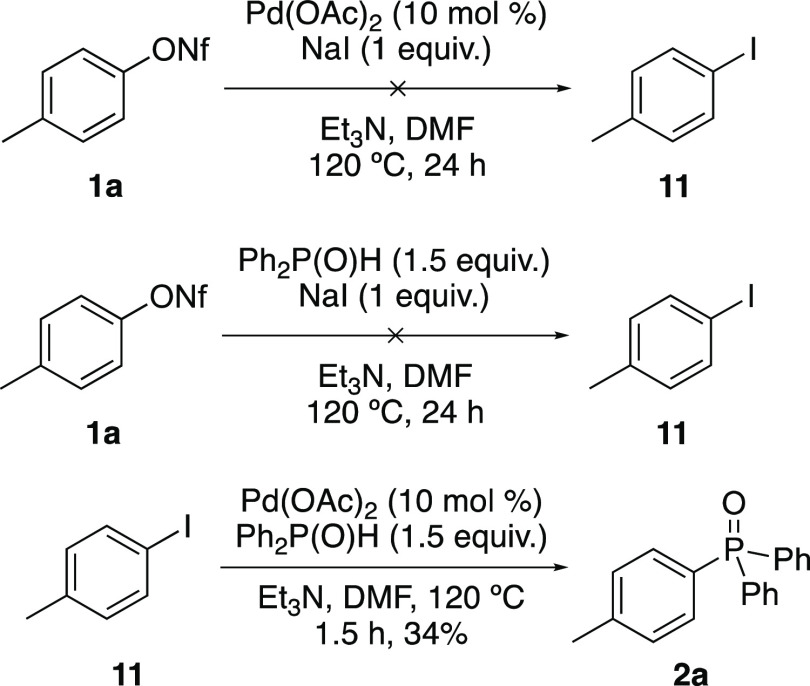
Experiments to Investigate the Role of *p*-Tolyl Iodide as an Intermediate

Halide and acetate additives have been shown to promote Pd-catalyzed cross-coupling reactions by the formation of more nucleophilic anionic palladium complexes.^15,16^ However, no accelerating effects were observed when acetate or chloride ions were employed during this transformation (Table 1). It has also been proposed that iodide accelerating effects during Pd-catalyzed cross-coupling reactions are due to the faster oxidative addition of aryl iodide intermediates formed in situ via a Finkelstein reaction.^20^ Using the optimized conditions for the coupling of *p*-tolyl nonaflate (**1a**) with diphenylphosphine oxide, control experiments were conducted to determine whether *p*-tolyl iodide (**11**) was an intermediate (Scheme 5). Repeating the reaction under the same conditions, but in the absence of either diphenylphosphine oxide or Pd(OAc)_2_, no iodide could be detected (by ^1^H NMR spectroscopy), even after 24 h. A final experiment to probe the mechanism investigated the use of *p*-tolyl iodide (**11**) as the starting material. Previous work using aryl iodides as substrates for palladium-catalyzed C–P cross-coupling reactions reported lower yields compared to other halide leaving groups.^21^ It was proposed that this was due to competing reduction of the ArPdI intermediate. Reaction of *p*-tolyl iodide (**11**) using our non-catalyzed, standard conditions was found to be fast, with completion observed after 1.5 h. However, this gave phosphine oxide **2a** in only 34% isolated yield. This is in contrast to *p*-tolyl nonaflate (**1a**), which under the same conditions required a reaction time of 24 h but gave **2a** in 79% yield (Table 1, entry 3). This difference in reaction times and isolated yields of **2a** suggest that an aryl iodide and the subsequent oxidative addition product, ArPdI are not intermediates during the reaction with aryl nonaflates and that the reaction of these proceeds via an alternative mechanism in the presence of iodide.

**Scheme 6:**
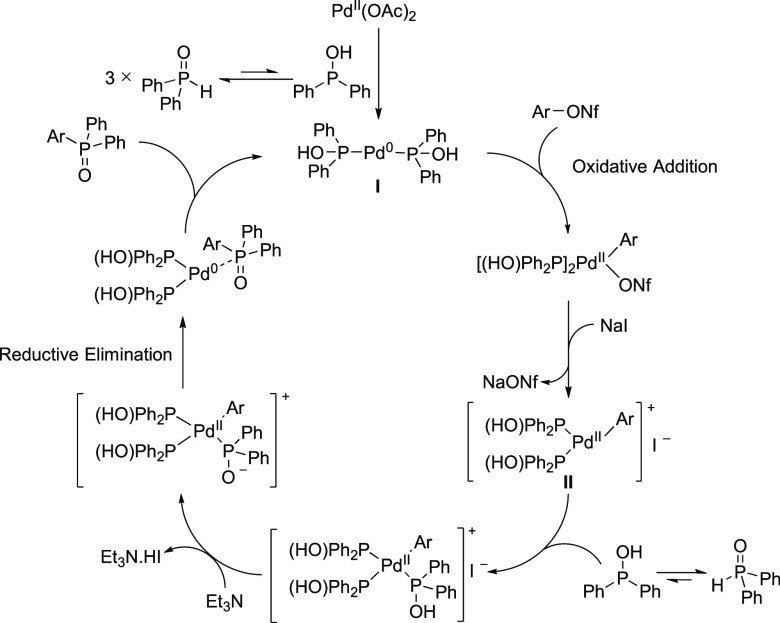
Proposed Mechanism for the Iodide-Accelerated, Palladium-Catalyzed C–P Bond-Forming Reaction

Based on these results and previous mechanistic studies by the Stawinski,^6a,6c^ Montchamp,^6f,6g^ and Keglevich groups,^6h,6i^ that implicated the role of the tautomer form of diphenylphosphine oxide as a reducing agent to form Pd(0), as a ligand and as the nucleophilic coupling partner, we propose the following catalytic cycle (Scheme 6). Initially, the active palladium species **I** is formed by reduction and coordination with the tautomeric form of the excess P(O)H coupling reagent (30 mol % required for 10 mol % Pd catalyst). Following oxidative addition of the aryl nonaflate by Pd(0) species **I**, the presence of NaI may result in the formation of sodium nonaflate and a coordinatively unsaturated Pd(0) complex **II**. With the iodide anion weakly bound, this may accelerate coordination and subsequent reaction with the phosphorus nucleophile,^22^ and following reductive elimination, allow overall faster access to the coupled product. There are other possible roles of iodide that could result in accelerated reactions. For example, the larger *trans* effect of the iodide when complexed to a Pd intermediate, in comparison to the other ionic additives, could also lead to an accelerated transformation through faster substitution reactions.^16^

## Conclusions

In summary, aryl nonaflates, which are isolable intermediates, readily prepared from abundant phenols, were found to be effective substrates for palladium-catalyzed C–P bond-forming reactions. Optimization studies revealed that the addition of NaI resulted in accelerated reactions, allowing the rapid synthesis of a wide range of aryl phosphine oxides. Extension of the process with other P(O)H coupling reagents resulted in the synthesis of further aryl phosphorus compounds, such as an aryl phosphinate and aryl phosphonates. This included the three-step synthesis of pharmaceutically relevant, phosphonophenylalanine **10** from a commercially available tyrosine derivative in 60% overall yield. Preliminary mechanistic studies suggested that the addition of iodide may accelerate the reaction via a coordinatively unsaturated Pd(0) complex or through the *trans* effect of a Pd–I intermediate. Investigation of further applications of this transformation is currently underway.

## Experimental Section

All reagents and starting materials, including methyl (2*S*)-2-[(benzyloxycarbonyl)amino]-3-(4-hydroxyphenyl)propanoate (**7**), were obtained from commercial sources and used as received, unless otherwise stated. Anhydrous dichloromethane was purified using a PureSolv 500 MD solvent purification system. All reactions were performed under an atmosphere of air unless otherwise stated. All reactions performed at elevated temperatures were heated using an oil bath. Dry glassware was oven-dried at 140 °C for a minimum of 16 h, cooled to room temperature *in vacuo*, and then purged with argon. Brine is defined as a saturated aqueous solution of sodium chloride. Merck aluminum-backed plates precoated with silica gel 60 (UV_254_) were used for thin-layer chromatography and were visualized under UV light (254/365 nm) and then stained with iodine, potassium permanganate, vanillin, or ninhydrin solution. Flash column chromatography was carried out using Merck Geduran Si 60 (40–63 μm). ^1^H and ^13^C NMR spectra were recorded on Bruker DPX 400, Bruker AVI 400, and Bruker AVIII 400 (^1^H 400 MHz; ^13^C 101 MHz) spectrometers or a Bruker AVIII 500 (^1^H 500 MHz; ^13^C 126 MHz) spectrometer with chemical shift values reported in ppm relative to tetramethylsilane (δ_H_ 0.00 and δ_C_ 0.0), CDCl_3_ (δ_H_ 7.26 and δ_C_ 77.2) or 3-(trimethylsilyl)propionic-2,2,3,3-*d*_4_ acid sodium salt in D_2_O (δ_H_ 0.00 and δ_C_ 0.0). Assignments of ^1^H and ^13^C NMR signals are based on COSY, DEPT, HSQC, and HMBC experiments. Mass spectra were obtained using a JEOL JMS-700 spectrometer or a Bruker microTOFq high-resolution mass spectrometer. Melting points were determined on a Gallenkamp melting point apparatus and are uncorrected. Infrared spectra were recorded neat on a Shimadzu FTIR-84005 spectrometer. Optical rotations were determined as solutions irradiating with the sodium D line (λ = 598 nm) using an Autopol V polarimeter. [α]_D_ values are reported in units 10^–1^ deg cm^2^ g^–1^. Di-(*n*-butyl)phosphine oxide was prepared as previously described via the reaction of *n*-butylmagnesium chloride with diethyl phosphite.^23^

### 4-Methylphenyl nonafluorobutanesulfonate (**1a**)^24^

In an oven-dried flask under argon, *p*-cresol (1.08 g, 10.0 mmol) was dissolved in anhydrous dichloromethane (33 mL) and cooled to 0 °C. Triethylamine (3.48 mL, 25.0 mmol) was then added followed by perfluoro-1-butanesulfonyl fluoride (2.70 mL, 15.0 mmol). The reaction mixture was warmed to room temperature and stirred for 2 h. The crude mixture was then diluted with dichloromethane (50 mL) and washed with water (3 × 50 mL). The organic layer was dried (MgSO_4_), filtered, and concentrated *in vacuo*. The crude material was purified by flash column chromatography eluting with 5% diethyl ether in petroleum ether (40–60) to give 4-methylphenyl nonafluorobutanesulfonate (**1a**) as a colorless oil (3.40 g, 87%). Spectroscopic data were consistent with the literature.^24^^1^H NMR (400 MHz, CDCl_3_) δ 2.38 (s, 3H), 7.16 (d, *J* = 8.6 Hz, 2H), 7.24 (d, *J* = 8.6 Hz, 2H); ^13^C{^1^H} NMR (101 MHz, CDCl_3_) δ 21.0 (CH_3_), 121.2 (2 × CH), 130.8 (2 × CH), 138.6 (C), 148.0 (C); MS (ESI) *m*/*z* 413 (M + Na^+^, 100).

### 3-Methylphenyl nonafluorobutanesulfonate (**1b**)

The reaction was carried out according to the previously described procedure for 4-methylphenyl nonafluorobutanesulfonate (**1a**) using *m*-cresol (0.209 mL, 2.00 mmol), anhydrous dichloromethane (5 mL), triethylamine (0.700 mL, 5.02 mmol), and perfluoro-1-butanesulfonyl fluoride (0.540 mL, 3.00 mmol). The reaction mixture was stirred at room temperature for 3 h. The crude material was purified by flash column chromatography eluting with 100% petroleum ether (40–60) to give 3-methylphenyl nonafluorobutanesulfonate (**1b**) as a colorless oil (0.588 g, 76%). IR (neat) 2970, 1740, 1425, 1354, 1231, 1198, 1142, 1117, 930 cm^–1^; ^1^H NMR (400 MHz, CDCl_3_) δ 2.41 (s, 3H), 7.06–7.12 (m, 2H), 7.17–7.22 (m, 1H), 7.29–7.36 (m, 1H); ^13^C{^1^H} NMR (101 MHz, CDCl_3_) *δ* 21.5 (CH_3_), 118.4 (CH), 122.0 (CH), 129.3 (CH), 130.0 (CH), 141.0 (C), 149.9 (C); MS (EI) *m/z* 390 (M^+^, 59), 326 (24), 151 (38), 107 (100), 91 (38), 77 (49); HRMS (EI) *m*/*z*: [M]^+^ calcd for C_11_H_7_F_9_O_3_S 389.9972; found 389.9953.

### 2-Methylphenyl nonafluorobutanesulfonate (**1c**)^24^

The reaction was carried out according to the previously described procedure for 4-methylphenyl nonafluorobutanesulfonate (**1a**) using *o*-cresol (0.270 g, 2.50 mmol), anhydrous dichloromethane (8 mL), triethylamine (0.870 mL, 6.25 mmol), and perfluoro-1-butanesulfonyl fluoride (0.680 mL, 3.77 mmol). The reaction mixture was stirred at room temperature for 2 h. The crude material was purified by flash column chromatography eluting with 5% diethyl ether in hexane to give 2-methylphenyl nonafluorobutanesulfonate (**1c**) as a colorless oil (0.708 g, 72%). Spectroscopic data were consistent with the literature.^24^^1^H NMR (400 MHz, CDCl_3_) δ 2.40 (s, 3H), 7.23–7.34 (m, 4H); ^13^C{^1^H} NMR (101 MHz, CDCl_3_) δ 16.6 (CH_3_), 121.4 (CH), 127.8 (CH), 128.4 (CH), 131.1 (C), 132.3 (CH), 148.8 (C); MS (ESI) *m*/*z* 413 (M + Na^+^, 100).

### 4-*tert*-Butylphenyl nonafluorobutanesulfonate (**1d**)^25^

The reaction was carried out according to the previously described procedure for 4-methylphenyl nonafluorobutanesulfonate (**1a**) using 4-*tert*-butylphenol (0.300 g, 2.00 mmol), anhydrous dichloromethane (6 mL), triethylamine (0.700 mL, 5.02 mmol), and perfluoro-1-butanesulfonyl fluoride (0.540 mL, 3.01 mmol). The reaction mixture was stirred at room temperature for 5 h. The crude material was purified by flash column chromatography eluting with 100% hexane to give 4-*tert*-butylphenyl nonafluorobutanesulfonate (**1d**) as a colorless oil (0.802 g, 93%). Spectroscopic data were consistent with the literature.^25^^1^H NMR (400 MHz, CDCl_3_) δ 1.33 (s, 9H), 7.20 (d, *J* = 9.0 Hz, 2H), 7.45 (d, *J* = 9.0 Hz, 2H); ^13^C{^1^H} NMR (101 MHz, CDCl_3_) *δ* 31.4 (3 × CH_3_), 34.9 (C), 120.9 (2 × CH), 127.3 (2 × CH), 147.8 (C), 151.8 (C); MS (ESI) *m*/*z* 455 (M + Na^+^, 100).

### Phenyl nonafluorobutanesulfonate (**1e**)^26^

The reaction was carried out according to the previously described procedure for 4-methylphenyl nonafluorobutanesulfonate (**1a**) using phenol (0.200 g, 2.12 mmol), anhydrous dichloromethane (7 mL), triethylamine (0.740 mL, 5.31 mmol), and perfluoro-1-butanesulfonyl fluoride (0.570 mL, 3.17 mmol). The reaction mixture was stirred at room temperature for 3 h. The crude material was purified by flash column chromatography eluting with 10% diethyl ether in petroleum ether (40–60) to give phenyl nonafluorobutanesulfonate (**1e**) as a colorless oil (0.720 g, 90%). Spectroscopic data were consistent with the literature.^26^^1^H NMR (400 MHz, CDCl_3_) δ 7.27–7.33 (m, 2H), 7.37–7.42 (m, 1H), 7.43–7.50 (m, 2H); ^13^C{^1^H} NMR (101 MHz, CDCl_3_) δ 121.5 (2 × CH), 128.5 (CH), 130.4 (2 × CH), 150.0 (C); MS (EI) *m*/*z* 376 (M^+^, 42), 312 (13), 219 (4), 143 (10), 93 (73), 84 (35), 77 (94), 69 (35), 65 (100).

### 2-Naphthyl nonafluorobutanesulfonate (**1f**)^27^

The reaction was carried out according to the previously described procedure for 4-methylphenyl nonafluorobutanesulfonate (**1a**) using 2-naphthol (1.00 g, 6.94 mmol), anhydrous dichloromethane (15 mL), triethylamine (2.42 mL, 17.4 mmol), and perfluoro-1-butanesulfonyl fluoride (1.87 mL, 10.4 mmol). The reaction mixture was stirred at room temperature for 1 h. The crude material was purified by flash column chromatography eluting with 5% ethyl acetate in petroleum ether (40–60) to give 2-naphthyl nonafluorobutanesulfonate (**1f**) as a colorless oil (1.97 g, 67%). Spectroscopic data were consistent with the literature.^27^^1^H NMR (400 MHz, CDCl_3_) δ 7.39 (dd, *J* = 9.0, 2.5 Hz, 1H), 7.54–7.63 (m, 2H), 7.77 (d, *J* = 2.5 Hz, 1H), 7.84–7.92 (m, 2H), 7.93 (d, *J* = 9.0 Hz, 1H); ^13^C{^1^H} NMR (101 MHz, CDCl_3_) δ 119.4 (CH), 119.7 (CH), 127.3 (CH), 127.7 (CH), 128.1 (CH), 128.2 (CH), 130.7 (CH), 132.5 (C), 133.5 (C), 147.5 (C); MS (ESI) *m*/*z* 425 [(M – H)^−^, 100].

### (1,1′-Biphenyl)-4-yl nonafluorobutanesulfonate (**1g**)^28^

The reaction was carried out according to the previously described procedure for 4-methylphenyl nonafluorobutanesulfonate (**1a**) using 4-phenylphenol (0.340 g, 2.00 mmol), anhydrous dichloromethane (6 mL), triethylamine (0.700 mL, 5.02 mmol), and perfluoro-1-butanesulfonyl fluoride (0.540 mL, 3.01 mmol). The reaction mixture was stirred at room temperature for 2 h. The crude material was purified by flash column chromatography eluting with 10% diethyl ether in hexane to give (1,1′-biphenyl)-4-yl nonafluorobutanesulfonate (**1g**) as a white solid (0.838 g, 93%). Mp 45–47 °C (lit.^28^ 45.5–46.7 °C); ^1^H NMR (400 MHz, CDCl_3_) δ 7.36 (d, *J* = 8.8 Hz, 2H), 7.38–7.42 (m, 1H), 7.44–7.49 (m, 2H), 7.54–7.58 (m, 2H), 7.65 (d, *J* = 8.8 Hz, 2H); ^13^C{^1^H} NMR (101 MHz, CDCl_3_) δ 121.8 (2 × CH), 127.3 (2 × CH), 128.2 (CH), 129.0 (2 × CH), 129.1 (2 × CH), 139.5 (C), 141.8 (C), 149.3 (C); MS (ESI) *m*/*z* 475 (M + Na^+^, 100).

### 4-Methoxyphenyl nonafluorobutanesulfonate (**1h**)^25^

The reaction was carried out according to the previously described procedure for 4-methylphenyl nonafluorobutanesulfonate (**1a**) using 4-methoxyphenol (0.372 g, 3.00 mmol), anhydrous dichloromethane (10 mL), triethylamine (1.05 mL, 7.50 mmol), and perfluoro-1-butanesulfonyl fluoride (0.810 mL, 4.50 mmol). The reaction mixture was stirred at room temperature for 2 h. The crude material was purified by flash column chromatography eluting with 10% diethyl ether in petroleum ether (40–60) to give 4-methoxyphenyl nonafluorobutanesulfonate (**1h**) as a colorless oil (1.13 g, 93%). Spectroscopic data were consistent with the literature.^25^^1^H NMR (500 MHz, CDCl_3_) *δ* 3.82 (s, 3H), 6.92 (d, *J* = 9.2 Hz, 2H), 7.21 (d, *J* = 9.2 Hz, 2H); ^13^C{^1^H} NMR (126 MHz, CDCl_3_) *δ* 55.8 (CH_3_), 115.2 (2 × CH), 122.5 (2 × CH), 143.4 (C), 159.2 (C); MS (EI) *m*/*z* 406 (M^+^, 12), 219 (5), 123 (100), 95 (14), 69 (11).

### 4-Acetamidophenyl nonafluorobutanesulfonate (**1i**)^29^

The reaction was carried out according to the previously described procedure for 4-methylphenyl nonafluorobutanesulfonate (**1a**) using 4-acetamidophenol (0.302 g, 2.00 mmol), anhydrous dichloromethane (6 mL), triethylamine (0.700 mL, 5.02 mmol), and perfluoro-1-butanesulfonyl fluoride (0.540 mL, 3.01 mmol). The reaction mixture was stirred at room temperature for 3 h. The crude material was purified by flash column chromatography eluting with 100% diethyl ether to give 4-acetamidophenyl nonafluorobutanesulfonate (**1i**) as a white solid (0.781 g, 90%). Mp 102–103 °C. Spectroscopic data were consistent with the literature.^29^^1^H NMR (400 MHz, CDCl_3_) δ 2.19 (s, 3H), 7.23 (d, *J* = 9.0 Hz, 2H), 7.41 (br s, 1H), 7.60 (d, *J* = 9.0 Hz, 2H); ^13^C{^1^H} NMR (101 MHz, CDCl_3_) δ 24.7 (CH_3_), 121.1 (2 × CH), 122.1 (2 × CH), 138.0 (C), 145.7 (C), 168.6 (C); MS (ESI) *m*/*z* 456 (M + Na^+^, 100).

### 4-Acetylphenyl nonafluorobutanesulfonate (**1j**)^25^

The reaction was carried out according to the previously described procedure for 4-methylphenyl nonafluorobutanesulfonate (**1a**) using 4-hydroxyacetophenone (0.272 g, 2.00 mmol), anhydrous dichloromethane (6 mL), triethylamine (0.700 mL, 5.02 mmol), and perfluoro-1-butanesulfonyl fluoride (0.540 mL, 3.01 mmol). The reaction mixture was stirred at room temperature for 2 h. The crude material was purified by flash column chromatography eluting with 50% diethyl ether in hexane to give 4-acetylphenyl nonafluorobutanesulfonate (**1j**) as a white solid (0.786 g, 94%). Mp 38–40 °C (lit.^25^ 38–40 °C); ^1^H NMR (500 MHz, CDCl_3_) δ 2.63 (s, 3H), 7.39 (d, *J* = 8.9 Hz, 2H), 8.06 (d, *J* = 8.9 Hz, 2H); ^13^C{^1^H} NMR (126 MHz, CDCl_3_) δ 26.8 (CH_3_), 121.8 (2 × CH), 130.7 (2 × CH), 137.0 (C), 152.9 (C), 196.3 (C); MS (EI) *m*/*z* 418 (M^+^, 30), 403 (100), 339 (38), 219 (8), 131 (11), 120 (37), 107 (38).

### 4-Nonafluorobutanesulfonyloxybenzophenone (**1k**)

The reaction was carried out according to the previously described procedure for 4-methylphenyl nonafluorobutanesulfonate (**1a**) using 4-hydroxybenzophenone (0.200 g, 1.01 mmol), anhydrous dichloromethane (5 mL), triethylamine (0.350 mL, 2.51 mmol), and perfluoro-1-butanesulfonyl fluoride (0.270 mL, 1.50 mmol). The reaction mixture was stirred at room temperature for 16 h. The crude material was purified by flash column chromatography eluting with 20% ethyl acetate in petroleum ether (40–60) to give 4-nonafluorobutanesulfonyloxybenzophenone (**1k**) as a beige solid (0.341 g, 70%). Mp 42–43 °C; IR (neat) 2980, 1740, 1651, 1424, 1227, 1202, 1138, 889, 797, 731 cm^–1^; ^1^H NMR (400 MHz, CDCl_3_) δ 7.42 (d, *J* = 8.6 Hz, 2H), 7.52 (t, *J* = 7.6 Hz, 2H), 7.63 (t, *J* = 7.6 Hz, 1H), 7.80 (d, *J* = 7.6 Hz, 2H), 7.91 (d, *J* = 8.6 Hz, 2H); ^13^C{^1^H} NMR (101 MHz, CDCl_3_) δ 121.5 (2 × CH), 128.7 (2 × CH), 130.1 (2 × CH), 132.3 (2 × CH), 133.2 (CH), 136.9 (C), 137.7 (C), 152.3 (C), 194.9 (C); MS (EI) *m*/*z* 480 (M^+^, 100), 169 (89), 105 (84), 84 (93), 63 (81); HRMS (EI) *m*/*z*: [M]^+^ calcd for C_17_H_9_F_9_O_4_S 480.0078; found 480.0083.

### Methyl 4-nonafluorobutanesulfonyloxybenzoate (**1l**)^25^

The reaction was carried out according to the previously described procedure for 4-methylphenyl nonafluorobutanesulfonate (**1a**) using methyl 4-hydroxybenzoate (0.200 g, 1.32 mmol), anhydrous dichloromethane (5 mL), triethylamine (0.460 mL, 3.30 mmol), and perfluoro-1-butanesulfonyl fluoride (0.360 mL, 2.00 mmol). The reaction mixture was stirred at room temperature for 16 h. The crude material was purified by flash column chromatography eluting with 15% ethyl acetate in petroleum ether (40–60) to give methyl 4-nonafluorobutanesulfonyloxybenzoate (**1l**) as a colorless oil (0.526 g, 92%). Spectroscopic data were consistent with the literature.^25^^1^H NMR (400 MHz, CDCl_3_) δ 3.94 (s, 3H), 7.36 (d, *J* = 8.9 Hz, 2H), 8.14 (d, *J* = 8.9 Hz, 2H); ^13^C{^1^H} NMR (101 MHz, CDCl_3_) δ 52.7 (CH_3_), 121.6 (2 × CH), 130.5 (C), 132.0 (2 × CH), 152.9 (C), 165.6 (C); MS (EI) *m*/*z* 434 (M^+^, 70), 403 (40), 339 (100), 151 (38), 123 (42).

### 4-Cyanophenyl nonafluorobutanesulfonate (**1m**)^25^

The reaction was carried out according to the previously described procedure for 4-methylphenyl nonafluorobutanesulfonate (**1a**) using 4-cyanophenol (0.200 g, 1.68 mmol), anhydrous dichloromethane (5 mL), triethylamine (0.590 mL, 4.23 mmol), and perfluoro-1-butanesulfonyl fluoride (0.460 mL, 2.56 mmol). The reaction mixture was stirred at room temperature for 2 h. The crude material was purified by flash column chromatography eluting with 20% ethyl acetate in petroleum ether (40–60) to give 4-cyanophenyl nonafluorobutanesulfonate (**1m**) as a white solid (0.609 g, 90%). Mp 109–110 °C (lit.^25^ 111–112 °C); ^1^H NMR (500 MHz, CDCl_3_) δ 7.44 (d, *J* = 9.0 Hz, 2H), 7.79 (d, *J* = 9.0 Hz, 2H); ^13^C{^1^H} NMR (101 MHz, CDCl_3_) δ 113.0 (C), 117.2 (C), 122.8 (2 × CH), 134.6 (2 × CH), 152.4 (C); MS (EI) *m*/*z* 401 (M^+^, 38), 337 (33), 219 (12), 118 (47), 102 (100), 90 (71), 77 (41), 69 (99).

### 4-(Trifluoromethyl)phenyl nonafluorobutanesulfonate (**1n**)^30^

The reaction was carried out according to the previously described procedure for 4-methylphenyl nonafluorobutanesulfonate (**1a**) using 4-hydroxybenzotrifluoride (0.200 g, 1.23 mmol), anhydrous dichloromethane (5 mL), triethylamine (0.430 mL, 3.09 mmol), and perfluoro-1-butanesulfonyl fluoride (0.330 mL, 1.84 mmol). The reaction mixture was stirred at room temperature for 16 h. The crude material was purified by flash column chromatography eluting with 20% ethyl acetate in petroleum ether (40–60) to give 4-(trifluoromethyl)phenyl nonafluorobutanesulfonate (**1n**) as a colorless oil (0.454 g, 83%). Spectroscopic data were consistent with the literature.^30^^1^H NMR (400 MHz, CDCl_3_) δ 7.43 (d, *J* = 8.8 Hz, 2H), 7.75 (d, *J* = 8.8 Hz, 2H); ^13^C{^1^H} NMR (101 MHz, CDCl_3_) δ 122.2 (2 × CH), 123.4 (q, ^1^*J*_CF_ 273.4 Hz, CF_3_), 127.9 (q, ^3^*J*_CF_ 3.6 Hz, 2 × CH), 131.0 (q, ^2^*J*_CF_ 33.4 Hz, C), 152.0 (C); MS (EI) *m*/*z* 444 (M^+^, 44), 145 (100), 133 (36), 78 (32), 69 (41).

### 2-Fluorophenyl nonafluorobutanesulfonate (**1o**)

The reaction was carried out according to the previously described procedure for 4-methylphenyl nonafluorobutanesulfonate (**1a**) using 2-fluorophenol (0.178 mL, 2.00 mmol), anhydrous dichloromethane (6 mL), triethylamine (0.700 mL, 5.02 mmol), and perfluoro-1-butanesulfonyl fluoride (0.540 mL, 3.01 mmol). The reaction mixture was stirred at room temperature for 3 h. The crude material was purified by flash column chromatography eluting with 10% diethyl ether in hexane to give 2-fluorophenyl nonafluorobutanesulfonate (**1o**) as a colorless oil (0.661 g, 84%). IR (neat) 1612, 1501, 1431, 1227, 1200, 1142, 1096, 895, 760 cm^–1^; ^1^H NMR (400 MHz, CDCl_3_) δ 7.19–7.24 (m, 1H), 7.27 (ddd, *J* = 9.8, 8.4, 1.4 Hz, 1H), 7.33–7.41 (m, 2H); ^13^C{^1^H} NMR (101 MHz, CDCl_3_) δ 117.8 (d, ^2^*J*_CF_ 18.2 Hz, CH), 123.6 (CH), 125.2 (d, ^3^*J*_CF_ 4.1 Hz, CH), 129.8 (d, ^3^*J*_CF_ 7.1 Hz, CH), 137.3 (d, ^2^*J*_CF_ 13.4 Hz, C), 153.9 (d, ^1^*J*_CF_ 254.6 Hz, C); MS (ESI) *m*/*z* 417 (M + Na^+^, 100); HRMS (ESI) *m*/*z*: [M + Na]^+^ calcd for C_10_H_4_F_10_NaO_3_S 416.9614; found 416.9614.

### 3-Chlorophenyl nonafluorobutanesulfonate (**1p**)^31^

The reaction was carried out according to the previously described procedure for 4-methylphenyl nonafluorobutanesulfonate (**1a**) using 3-chlorophenol (0.257 g, 2.00 mmol), anhydrous dichloromethane (6 mL), triethylamine (0.700 mL, 5.02 mmol), and perfluoro-1-butanesulfonyl fluoride (0.540 mL, 3.01 mmol). The reaction mixture was stirred at room temperature for 2 h. The crude material was purified by flash column chromatography eluting with 100% hexane to give 3-chlorophenyl nonafluorobutanesulfonate (**1p**) as a colorless oil (0.672 g, 82%). Spectroscopic data were consistent with the literature.^31^^1^H NMR (400 MHz, CDCl_3_) δ 7.18–7.24 (m, 1H), 7.30–7.33 (m, 1H), 7.37–7.43 (m, 2H); ^13^C{^1^H} NMR (101 MHz, CDCl_3_) δ 119.9 (CH), 122.2 (CH), 129.0 (CH), 131.1 (CH), 135.8 (C), 149.9 (C); MS (EI) *m*/*z* 410 (M^+^, 33), 348 (9), 346 (27), 127 (28), 111 (44), 99 (34), 84 (100).

### 3-Bromophenyl nonafluorobutanesulfonate (**1q**)

The reaction was carried out according to the previously described procedure for 4-methylphenyl nonafluorobutanesulfonate (**1a**) using 3-bromophenol (0.356 g, 2.06 mmol), anhydrous dichloromethane (5 mL), triethylamine (0.720 mL, 5.17 mmol), and perfluoro-1-butanesulfonyl fluoride (0.560 mL, 3.11 mmol). The reaction mixture was stirred at room temperature for 1 h. The crude material was purified by flash column chromatography eluting with 100% petroleum ether (40–60) to give 3-bromophenyl nonafluorobutanesulfonate (**1q**) as a colorless oil (0.830 g, 88%). IR (neat) 1582, 1468, 1425, 1354, 1227, 1200, 1142, 1034, 897, 785 cm^–1^; ^1^H NMR (400 MHz, CDCl_3_) δ 7.26 (ddd, *J* = 8.2, 2.0, 0.8 Hz, 1H), 7.34 (t, *J* = 8.2 Hz, 1H), 7.47 (t, *J* = 2.0 Hz, 1H), 7.55 (ddd, *J* = 8.2, 2.0, 0.8 Hz, 1H); ^13^C{^1^H} NMR (101 MHz, CDCl_3_) δ 120.3 (CH), 123.2 (C), 125.0 (CH), 131.4 (CH), 131.9 (CH), 149.9 (C); MS (EI) *m*/*z* 454 (M^+^, 42), 392 (21), 390 (22), 173 (20), 171 (20), 157 (27), 155 (28), 83 (100), 78 (50), 63 (59); HRMS (EI) *m*/*z*: [M]^+^ calcd for C_10_H_4_^79^BrF_9_O_3_S 453.8921; found 453.8920.

### Pyridin-2-yl nonafluorobutanesulfonate (**1r**)^12h^

The reaction was carried out according to the previously described procedure for 4-methylphenyl nonafluorobutanesulfonate (**1a**) using pyridin-2-ol (0.190 g, 2.00 mmol), anhydrous dichloromethane (6 mL), triethylamine (0.700 mL, 5.02 mmol), and perfluoro-1-butanesulfonyl fluoride (0.540 mL, 3.01 mmol). The reaction mixture was stirred at room temperature for 166 h. The crude material was purified by flash column chromatography eluting with 30% diethyl ether in hexane to give pyridin-2-yl nonafluorobutanesulfonate (**1r**) as a colorless oil (0.495 g, 66%). Spectroscopic data were consistent with the literature.^12h^^1^H NMR (400 MHz, CDCl_3_) δ 7.19 (br d, *J* = 8.2 Hz, 1H), 7.40 (ddd, *J* = 7.4, 4.8, 0.4 Hz, 1H), 7.90 (ddd, *J* = 8.2, 7.4, 2.0 Hz, 1H), 8.42 (dd, *J* = 4.8, 2.0 Hz, 1H); ^13^C{^1^H} NMR (101 MHz, CDCl_3_) δ 115.4 (CH), 124.4 (CH), 141.1 (CH), 148.9 (CH), 156.1 (C); MS (ESI) *m*/*z* 400 (M + Na^+^, 100).

### (4-Methylphenyl)diphenylphosphine Oxide (**2a**)^32^

#### General Procedure Using 1 equiv of Sodium Iodide

A stirrer bar and sodium iodide (0.0600 g, 0.400 mmol) were added to a microwave tube and dried in an oven at 140 °C overnight. 4-Methylphenyl nonafluorobutanesulfonate (**1a**) (0.156 g, 0.400 mmol) was dried under high vacuum for 1 h, purged with argon, and dissolved in anhydrous *N*,*N*′-dimethylformamide (2.4 mL). Diphenylphosphine oxide (0.121 g, 0.600 mmol) was dried *in vacuo* for 1 h. The oven-dried microwave tube was cooled to room temperature *in vacuo* and then purged with argon. To the tube was added diphenylphosphine oxide and palladium(II) acetate (0.00900 g, 0.0400 mmol), followed by the 4-methylphenyl nonafluorobutanesulfonate (**1a**) solution and triethylamine (0.220 mL, 1.58 mmol). The tube was sealed, heated to 120 °C, and stirred for 4 h. The reaction mixture was cooled to room temperature, diluted with ethyl acetate (15 mL), and washed with 2 M aqueous lithium chloride solution (3 × 15 mL). The organic layer was dried (MgSO_4_), filtered, and concentrated *in vacuo*. The crude material was purified by flash column chromatography eluting with 30% ethyl acetate in dichloromethane to give (4-methylphenyl)diphenylphosphine oxide (**2a**) as a white solid (0.0905 g, 78%). Mp 118–120 °C. Spectroscopic data were consistent with previously published data.^32^^1^H NMR (400 MHz, CDCl_3_) δ 2.40 (s, 3H), 7.27 (dd, *J* = 8.0, 2.8 Hz, 2H), 7.41–7.49 (m, 4H), 7.50–7.59 (m, 4H), 7.62–7.71 (m, 4H); ^13^C{^1^H} NMR (101 MHz, CDCl_3_) δ 21.8 (d, ^5^*J*_CP_ = 1.3 Hz, CH_3_), 128.6 (d, ^3^*J*_CP_ = 12.1 Hz, 4 × CH), 129.3 (d, ^1^*J*_CP_ = 107.0 Hz, C), 129.4 (d, ^3^*J*_CP_ = 12.5 Hz, 2 × CH), 131.9 (d, ^4^*J*_CP_ = 2.7 Hz, 2 × CH), 132.2 (d, ^2^*J*_CP_ = 9.8 Hz, 4 × CH), 132.3 (d, ^2^*J*_CP_ = 10.3 Hz, 2 × CH), 133.0 (d, ^1^*J*_CP_ = 104.3 Hz, 2 × C), 142.6 (d, ^4^*J*_CP_ = 2.7 Hz, C); MS (ESI) *m*/*z* 315 (M + Na^+^, 100).

#### Using 0.1 equiv of Sodium Iodide

The reaction was carried out according to the previously described procedure for (4-methylphenyl)diphenylphosphine oxide (**2a**) using sodium iodide (0.00300 g, 0.0200 mmol), 4-methylphenyl nonafluorobutanesulfonate (**1a**) (0.0780 g, 0.200 mmol), anhydrous *N*,*N*′-dimethylformamide (1.2 mL), diphenylphosphine oxide (0.0610 g, 0.302 mmol), palladium(II) acetate (0.00450 g, 0.0200 mmol), and triethylamine (0.110 mL, 0.790 mmol). The reaction mixture was stirred at 120 °C for 8 h. The crude material was purified by flash column chromatography eluting with 2% methanol in diethyl ether to give (4-methylphenyl)diphenylphosphine oxide (**2a**) as a white solid (0.0441 g, 76%). Spectroscopic data were consistent as described above.

#### Without Sodium Iodide

The reaction was carried out according to the previously described procedure for (4-methylphenyl)diphenylphosphine oxide (**2a**) using 4-methylphenyl nonafluorobutanesulfonate (**1a**) (0.0780 g, 0.200 mmol), anhydrous *N*,*N*′-dimethylformamide (1.2 mL), diphenylphosphine oxide (0.0610 g, 0.302 mmol), palladium(II) acetate (0.00450 g, 0.0200 mmol), and triethylamine (0.110 mL, 0.790 mmol). The reaction mixture was heated to 120 °C and stirred for 24 h. The crude material was purified by flash column chromatography eluting with 2% methanol in diethyl ether to give (4-methylphenyl)diphenylphosphine oxide (**2a**) as a white solid (0.0461 g, 79%). Spectroscopic data were consistent as described above.

#### Using **1** equiv of Sodium Acetate

The reaction was carried out according to the previously described procedure for (4-methylphenyl)diphenylphosphine oxide (**2a**) using sodium acetate (0.0328 g, 0.400 mmol), 4-methylphenyl nonafluorobutanesulfonate (**1a**) (0.156 g, 0.400 mmol), anhydrous *N*,*N*′-dimethylformamide (2.4 mL), diphenylphosphine oxide (0.121 g, 0.600 mmol), palladium(II) acetate (0.00900 g, 0.0400 mmol), and triethylamine (0.220 mL, 1.58 mmol). The reaction was heated to 120 °C and stirred for 22 h. The crude material was purified by flash column chromatography eluting with 2% methanol in diethyl ether to give (4-methylphenyl)diphenylphosphine oxide (**2a**) as a white solid (0.0642 g, 55%). Spectroscopic data were consistent as described above.

#### Using **1** equiv of Sodium Chloride

The reaction was carried out according to the previously described procedure for (4-methylphenyl)diphenylphosphine oxide (**2a**) using sodium chloride (0.0234 g, 0.400 mmol), 4-methylphenyl nonafluorobutanesulfonate (**1a**) (0.156 g, 0.400 mmol), anhydrous *N*,*N*′-dimethylformamide (2.4 mL), diphenylphosphine oxide (0.121 g, 0.600 mmol), palladium(II) acetate (0.00900 g, 0.0400 mmol), and triethylamine (0.220 mL, 1.58 mmol). The reaction was heated to 120 °C and stirred for 32 h. The crude material was purified by flash column chromatography eluting with 2% methanol in diethyl ether to give (4-methylphenyl)diphenylphosphine oxide (**2a**) as a white solid (0.0745 g, 64%). Spectroscopic data were consistent as described above.

### (3-Methylphenyl)diphenylphosphine Oxide (**2b**)^33^

The reaction was carried out according to the previously described general procedure for (4-methylphenyl)diphenylphosphine oxide (**2a**) using sodium iodide (0.0600 g, 0.400 mmol), 3-methylphenyl nonafluorobutanesulfonate (**1b**) (0.156 g, 0.400 mmol), anhydrous *N*,*N*′-dimethylformamide (2.4 mL), diphenylphosphine oxide (0.121 g, 0.600 mmol), palladium(II) acetate (0.00900 g, 0.0400 mmol), and triethylamine (0.220 mL, 1.58 mmol). The reaction mixture was stirred at 120 °C for 4 h. The crude material was purified by flash column chromatography eluting with 2% methanol in diethyl ether to give (3-methylphenyl)diphenylphosphine oxide (**2b**) as a pale orange solid (0.111 g, 95%). Mp 112–114 °C. Spectroscopic data were consistent with previously published data.^33^^1^H NMR (400 MHz, CDCl_3_) δ 2.36 (s, 3H), 7.29–7.41 (m, 3H), 7.42–7.49 (m, 4H), 7.54 (ttd, *J* = 7.3, 1.7, 1.6 Hz, 2H), 7.58 (br d, *J* = 12.4 Hz, 1H), 7.62–7.71 (m, 4H); ^13^C{^1^H} NMR (101 MHz, CDCl_3_) δ 21.6 (CH_3_), 128.4 (d, ^3^*J*_CP_ = 12.9 Hz, CH), 128.6 (d, ^3^*J*_CP_ = 12.1 Hz, 4 × CH), 129.3 (d, ^2^*J*_CP_ = 10.3 Hz, CH), 132.0 (d, ^4^*J*_CP_ = 2.7 Hz, 2 × CH), 132.2 (d, ^2^*J*_CP_ = 9.9 Hz, 4 × CH), 132.5 (d, ^1^*J*_CP_ = 104.5 Hz, C), 132.6 (d, ^2^*J*_CP_ = 9.5 Hz, CH), 132.8 (d, ^4^*J*_CP_ = 2.8 Hz, CH), 132.9 (d, ^1^*J*_CP_ = 104.2 Hz, 2 × C), 138.6 (d, ^3^*J*_CP_ = 12.1 Hz, C); MS (ESI) *m*/*z* 315 (M + Na^+^, 100).

### (2-Methylphenyl)diphenylphosphine Oxide (**2c**)^32^

The reaction was carried out according to the previously described general procedure for (4-methylphenyl)diphenylphosphine oxide (**2a**) using sodium iodide (0.0600 g, 0.400 mmol), 2-methylphenyl nonafluorobutanesulfonate (**1c**) (0.156 g, 0.400 mmol), anhydrous *N*,*N*′-dimethylformamide (2.4 mL), diphenylphosphine oxide (0.121 g, 0.600 mmol), palladium(II) acetate (0.00900 g, 0.0400 mmol), and triethylamine (0.220 mL, 1.58 mmol). The reaction mixture was stirred at 120 °C for 7 h. The crude material was purified by flash column chromatography eluting with 2% methanol in diethyl ether to give (2-methylphenyl)diphenylphosphine oxide (**2c**) as an off-white solid (0.0679 g, 58%). Mp 119–121 °C (lit.^32^ 121.5–122.9 °C); ^1^H NMR (400 MHz, CDCl_3_) δ 2.45 (s, 3H), 7.03 (ddd, *J* = 14.0, 7.5, 1.3 Hz, 1H), 7.13 (br td, *J* = 7.5, 2.0 Hz, 1H), 7.28 (br dd, *J* = 7.5, 4.0 Hz, 1H), 7.41 (tt, *J* = 7.5, 1.3 Hz, 1H), 7.44–7.50 (m, 4H), 7.55 (ttd, *J* = 7.4, 1.8, 1.6 Hz, 2H), 7.61–7.70 (m, 4H); ^13^C{^1^H} NMR (101 MHz, CDCl_3_) δ 21.8 (d, ^3^*J*_CP_ = 4.6 Hz, CH_3_), 125.3 (d, ^3^*J*_CP_ = 12.9 Hz, CH), 128.7 (d, ^3^*J*_CP_ = 12.1 Hz, 4 × CH), 131.0 (d, ^1^*J*_CP_ = 103.5 Hz, C), 131.9 (d, ^4^*J*_CP_ = 2.8 Hz, 2 × CH), 132.0 (d, ^3^*J*_CP_ = 10.5 Hz, CH), 132.1 (d, ^2^*J*_CP_ = 9.9 Hz, 4 × CH), 132.2 (d, ^4^*J*_CP_ = 2.6 Hz, CH), 133.0 (d, ^1^*J*_CP_ = 103.7 Hz, 2 × C), 133.6 (d, ^2^*J*_CP_ = 12.8 Hz, CH), 143.5 (d, ^2^*J*_CP_ = 8.1 Hz, C); MS (ESI) *m*/*z* 315 (M + Na^+^, 100).

### (4-*tert*-Butylphenyl)diphenylphosphine Oxide (**2d**)^34^

The reaction was carried out according to the previously described general procedure for (4-methylphenyl)diphenylphosphine oxide (**2a**) using sodium iodide (0.0600 g, 0.400 mmol), 4-*tert*-butylphenyl nonafluorobutanesulfonate (**1d**) (0.173 g, 0.400 mmol), anhydrous *N*,*N*′-dimethylformamide (2.4 mL), diphenylphosphine oxide (0.121 g, 0.600 mmol), palladium(II) acetate (0.00900 g, 0.0400 mmol), and triethylamine (0.220 mL, 1.58 mmol). The reaction mixture was stirred at 120 °C for 6 h. The crude material was purified by flash column chromatography eluting with 2% methanol in dichloromethane to give (4-*tert*-butylphenyl)diphenylphosphine oxide (**2d**) as an orange solid (0.0857 g, 64%). Mp 113–115 °C. Spectroscopic data were consistent with previously published data.^34^^1^H NMR (400 MHz, CDCl_3_) δ 1.32 (s, 9H), 7.41–7.49 (m, 6H), 7.50–7.56 (m, 2H), 7.58 (dd, *J* = 11.8, 8.6 Hz, 2H), 7.63–7.72 (m, 4H); ^13^C{^1^H} NMR (101 MHz, CDCl_3_) δ 31.2 (3 × CH_3_), 35.2 (C), 125.7 (d, ^3^*J*_CP_ = 12.4 Hz, 2 × CH), 128.6 (d, ^3^*J*_CP_ = 12.1 Hz, 4 × CH), 129.2 (d, ^1^*J*_CP_ = 106.9 Hz, C), 131.9 (d, ^4^*J*_CP_ = 2.8 Hz, 2 × CH), 132.1 (d, ^2^*J*_CP_ = 10.3 Hz, 2 × CH), 132.2 (d, ^2^*J*_CP_ = 9.9 Hz, 4 × CH), 133.0 (d, ^1^*J*_CP_ = 104.3 Hz, 2 × C), 155.5 (d, ^4^*J*_CP_ = 2.8 Hz, C); MS (ESI) *m*/*z* 357 (M + Na^+^, 100).

### Triphenylphosphine Oxide (**2e**)^20b^

The reaction was carried out according to the previously described general procedure for (4-methylphenyl)diphenylphosphine oxide (**2a**) using sodium iodide (0.0600 g, 0.400 mmol), phenyl nonafluorobutanesulfonate (**1e**) (0.150 g, 0.400 mmol), anhydrous *N*,*N*′-dimethylformamide (2.4 mL), diphenylphosphine oxide (0.121 g, 0.600 mmol), palladium(II) acetate (0.00900 g, 0.0400 mmol), and triethylamine (0.220 mL, 1.58 mmol). The reaction mixture was stirred at 120 °C for 4 h. The crude material was purified by flash column chromatography eluting with 2% methanol in diethyl ether to give triphenylphosphine oxide (**2e**) as a white solid (0.0740 g, 67%). Mp 144–146 °C (lit.^20b^ 148–149 °C); ^1^H NMR (400 MHz, CDCl_3_) δ 7.42–7.50 (m, 6H), 7.55 (ttd, *J* = 7.5, 1.6, 1.6 Hz, 3H), 7.62–7.72 (m, 6H); ^13^C{^1^H} NMR (101 MHz, CDCl_3_) *δ* 128.6 (d, ^3^*J*_CP_ = 12.1 Hz, 6 × CH), 132.1 (d, ^4^*J*_CP_ = 2.8 Hz, 3 × CH), 132.2 (d, ^2^*J*_CP_ = 10.0 Hz, 6 × CH), 132.7 (d, ^1^*J*_CP_ = 104.5 Hz, 3 × C); MS (ESI) *m*/*z* 301 (M + Na^+^, 100).

### (2-Naphthyl)diphenylphosphine Oxide (**2f**)^35^

The reaction was carried out according to the previously described general procedure for (4-methylphenyl)diphenylphosphine oxide (**2a**) using sodium iodide (0.0600 g, 0.400 mmol), 2-naphthyl nonafluorobutanesulfonate (**1f**) (0.171 g, 0.400 mmol), anhydrous *N*,*N*′-dimethylformamide (2.4 mL), diphenylphosphine oxide (0.121 g, 0.600 mmol), palladium(II) acetate (0.00900 g, 0.0400 mmol), and triethylamine (0.220 mL, 1.58 mmol). The reaction mixture was stirred at 90 °C for 3 h. The crude material was purified by flash column chromatography eluting with 30% ethyl acetate in dichloromethane to give (2-naphthyl)diphenylphosphine oxide (**2f**) as a pale yellow solid (0.122 g, 93%). Mp 106–108 °C. Spectroscopic data were consistent with previously published data.^35^^1^H NMR (400 MHz, CDCl_3_) δ 7.44–7.51 (m, 4H), 7.52–7.67 (m, 5H), 7.68–7.77 (m, 4H), 7.85–7.94 (m, 3H), 8.29 (d, *J* = 13.6 Hz, 1H); ^13^C{^1^H} NMR (101 MHz, CDCl_3_) *δ* 127.0 (d, ^3^*J*_CP_ = 10.8 Hz, CH), 127.1 (d, ^5^*J*_CP_ = 0.6 Hz, CH), 128.0 (d, ^5^*J*_CP_ = 0.8 Hz, CH), 128.3 (d, ^4^*J*_CP_ = 2.2 Hz, CH), 128.4 (d, ^2^*J*_CP_ = 9.8 Hz, CH), 128.7 (d, ^3^*J*_CP_ = 12.1 Hz, 4 × CH), 129.1 (CH), 129.8 (d, ^1^*J*_CP_ = 104.6 Hz, C), 132.1 (d, ^4^*J*_CP_ = 2.6 Hz, 2 × CH), 132.3 (d, ^2^*J*_CP_ = 10.1 Hz, 4 × CH), 132.6 (d, ^3^*J*_CP_ = 13.2 Hz, C), 132.8 (d, ^1^*J*_CP_ = 103.9 Hz, 2 × C), 134.2 (d, ^2^*J*_CP_ = 9.4 Hz, CH), 134.9 (d, ^4^*J*_CP_ = 2.4 Hz, C); MS (ESI) *m*/*z* 351 (M + Na^+^, 100).

### (1,1′-Biphenyl)-4-yldiphenylphosphine Oxide (**2g**)^35^

The reaction was carried out according to the previously described general procedure for (4-methylphenyl)diphenylphosphine oxide (**2a**) using sodium iodide (0.0300 g, 0.200 mmol), (1,1′-biphenyl)-4-yl nonafluorobutanesulfonate (**1g**) (0.0905 g, 0.200 mmol), anhydrous *N*,*N*′-dimethylformamide (1.2 mL), diphenylphosphine oxide (0.0610 g, 0.302 mmol), palladium(II) acetate (0.00450 g, 0.0200 mmol), and triethylamine (0.110 mL, 0.790 mmol). The reaction mixture was stirred at 120 °C for 4 h. The crude material was purified by flash column chromatography eluting with 1% methanol in diethyl ether to give (1,1′-biphenyl)-4-yldiphenylphosphine oxide (**2g**) as a pale brown oil (0.0680 g, 96%). Spectroscopic data were consistent with previously published data.^35^^1^H NMR (400 MHz, CDCl_3_) δ 7.39 (tt, *J* = 7.3, 1.7 Hz, 1H), 7.43–7.52 (m, 6H), 7.53–7.63 (m, 4H), 7.66–7.78 (m, 8H); ^13^C{^1^H} NMR (101 MHz, CDCl_3_) *δ* 127.3 (d, ^3^*J*_CP_ = 12.5 Hz, 2 × CH), 127.4 (2 × CH), 128.3 (CH), 128.7 (d, ^3^*J*_CP_ = 12.2 Hz, 4 × CH), 129.1 (2 × CH), 131.2 (d, ^1^*J*_CP_ = 105.6 Hz, C), 132.1 (d, ^4^*J*_CP_ = 2.7 Hz, 2 × CH), 132.2 (d, ^2^*J*_CP_ = 10.0 Hz, 4 × CH), 132.7 (d, ^2^*J*_CP_ = 10.3 Hz, 2 × CH), 132.7 (d, ^1^*J*_CP_ = 104.8 Hz, 2 × C), 140.0 (d, ^5^*J*_CP_ = 0.7 Hz, C), 144.8 (d, ^4^*J*_CP_ = 2.8 Hz, C); MS (ESI) *m*/*z* 377 (M + Na^+^, 100).

### (4-Methoxyphenyl)diphenylphosphine Oxide (**2h**)^20b^

The reaction was carried out according to the previously described general procedure for (4-methylphenyl)diphenylphosphine oxide (**2a**) using sodium iodide (0.0600 g, 0.400 mmol), 4-methoxyphenyl nonafluorobutanesulfonate (**1h**) (0.162 g, 0.400 mmol), anhydrous *N*,*N*′-dimethylformamide (2.4 mL), diphenylphosphine oxide (0.121 g, 0.600 mmol), palladium(II) acetate (0.00900 g, 0.0400 mmol), and triethylamine (0.220 mL, 1.58 mmol). The reaction mixture was stirred at 120 °C for 6 h. The crude material was purified by flash column chromatography eluting with 2% methanol in diethyl ether to give (4-methoxyphenyl)diphenylphosphine oxide (**2h**) as a pale yellow solid (0.0784 g, 64%). Mp 103–105 °C (lit.^20b^ 106–108 °C); ^1^H NMR (400 MHz, CDCl_3_) δ 3.84 (s, 3H), 6.96 (dd, *J* = 8.8, 2.4 Hz, 2H), 7.40–7.55 (m, 6H), 7.58 (dd, *J* = 11.2, 8.8 Hz, 2H), 7.62–7.71 (m, 4H); ^13^C{^1^H} NMR (101 MHz, CDCl_3_) δ 55.5 (CH_3_), 114.2 (d, ^3^*J*_CP_ = 13.1 Hz, 2 × CH), 123.8 (d, ^1^*J*_CP_ = 110.8 Hz, C), 128.6 (d, ^3^*J*_CP_ = 12.1 Hz, 4 × CH), 131.9 (d, ^4^*J*_CP_ = 2.7 Hz, 2 × CH), 132.2 (d, ^2^*J*_CP_ = 10.0 Hz, 4 × CH), 133.2 (d, ^1^*J*_CP_ = 104.7 Hz, 2 × C), 134.1 (d, ^2^*J*_CP_ = 11.2 Hz, 2 × CH), 162.6 (d, ^4^*J*_CP_ = 2.8 Hz, C); MS (ESI) *m*/*z* 331 (M + Na^+^, 100).

### (4-Acetamidophenyl)diphenylphosphine Oxide (**2i**)^36^

The reaction was carried out according to the previously described general procedure for (4-methylphenyl)diphenylphosphine oxide (**2a**) using sodium iodide (0.0300 g, 0.200 mmol), 4-acetamidophenyl nonafluorobutanesulfonate (**1i**) (0.0866 g, 0.200 mmol), anhydrous *N*,*N*′-dimethylformamide (1.2 mL), diphenylphosphine oxide (0.0610 g, 0.302 mmol), palladium(II) acetate (0.00450 g, 0.0200 mmol), and triethylamine (0.110 mL, 0.790 mmol). The reaction mixture was heated to 120 °C and stirred for 6 h. The reaction mixture was cooled to room temperature, diluted with ethyl acetate (15 mL) and washed with water (3 × 15 mL). The organic layer was dried (MgSO_4_), filtered, and concentrated *in vacuo*. The crude material was purified by flash column chromatography eluting with 8% methanol in diethyl ether to give (4-acetamidophenyl)diphenylphosphine oxide (**2i**) as a brown solid (0.0431 g, 65%). Mp 144–146 °C (lit.^36^ 150–152 °C); ^1^H NMR (400 MHz, CDCl_3_) δ 2.14 (s, 3H), 7.40–7.50 (m, 6H), 7.54 (ttd, *J* = 7.4, 1.7, 1.6 Hz, 2H), 7.58–7.69 (m, 6H), 9.28 (br s, 1H); ^13^C{^1^H} NMR (101 MHz, CDCl_3_) *δ* 24.6 (CH_3_), 119.7 (d, ^3^*J*_CP_ = 12.6 Hz, 2 × CH), 126.3 (d, ^1^*J*_CP_ = 108.7 Hz, C), 128.7 (d, ^3^*J*_CP_ = 12.2 Hz, 4 × CH), 132.1 (d, ^2^*J*_CP_ = 10.0 Hz, 4 × CH), 132.2 (d, ^4^*J*_CP_ = 2.6 Hz, 2 × CH), 132.4 (d, ^1^*J*_CP_ = 105.2 Hz, 2 × C), 133.1 (d, ^2^*J*_CP_ = 10.9 Hz, 2 × CH), 142.4 (d, ^4^*J*_CP_ = 3.0 Hz, C), 169.6 (C); MS (ESI) *m*/*z* 358 (M + Na^+^, 100).

### (4-Acetylphenyl)diphenylphosphine Oxide (**2j**)^37^

The reaction was carried out according to the previously described general procedure for (4-methylphenyl)diphenylphosphine oxide (**2a**) using sodium iodide (0.0300 g, 0.200 mmol), 4-acetylphenyl nonafluorobutanesulfonate (**1i**) (0.0836 g, 0.200 mmol), anhydrous *N*,*N*′-dimethylformamide (1.2 mL), diphenylphosphine oxide (0.0610 g, 0.302 mmol), palladium(II) acetate (0.00450 g, 0.0200 mmol), and triethylamine (0.110 mL, 0.790 mmol). The reaction mixture was stirred at 120 °C for 4 h. The crude material was purified by flash column chromatography eluting with 2% methanol in diethyl ether to give (4-acetylphenyl)diphenylphosphine oxide (**2i**) as a pale yellow solid (0.0615 g, 96%). Mp 116–118 °C (lit.^37^ 120.0–120.5 °C); ^1^H NMR (400 MHz, CDCl_3_) δ 2.63 (s, 3H), 7.42–7.52 (m, 4H), 7.53–7.60 (m, 2H), 7.61–7.71 (m, 4H), 7.79 (dd, *J* = 10.8, 8.4 Hz, 2H), 8.02 (dd, *J* = 8.4, 2.0 Hz, 2H); ^13^C{^1^H} NMR (101 MHz, CDCl_3_) δ 27.0 (CH_3_), 128.2 (d, ^3^*J*_CP_ = 12.1 Hz, 2 × CH), 128.8 (d, ^3^*J*_CP_ = 12.3 Hz, 4 × CH), 132.0 (d, ^1^*J*_CP_ = 105.2 Hz, 2 × C), 132.2 (d, ^2^*J*_CP_ = 10.0 Hz, 4 × CH), 132.4 (d, ^4^*J*_CP_ = 2.7 Hz, 2 × CH), 132.6 (d, ^2^*J*_CP_ = 10.1 Hz, 2 × CH), 137.9 (d, ^1^*J*_CP_ = 101.0 Hz, C), 139.6 (d, ^4^*J*_CP_ = 2.7 Hz, C), 197.7 (d, ^5^*J*_CP_ = 0.8 Hz, C); MS (ESI) *m*/*z* 343 (M + Na^+^, 100).

### 4-(Diphenylphosphoryl)benzophenone (**2k**)

The reaction was carried out according to the previously described general procedure for (4-methylphenyl)diphenylphosphine oxide (**2a**) using sodium iodide (0.0300 g, 0.200 mmol), 4-nonafluorobutanesulfonyloxybenzophenone (**1k**) (0.0960 g, 0.200 mmol), anhydrous *N*,*N*′-dimethylformamide (1.2 mL), diphenylphosphine oxide (0.0610 g, 0.302 mmol), palladium(II) acetate (0.00450 g, 0.0200 mmol), and triethylamine (0.110 mL, 0.790 mmol). The reaction mixture was stirred at 120 °C for 4 h. The crude material was purified by flash column chromatography eluting with 2% methanol in diethyl ether to give 4-(diphenylphosphoryl)benzophenone (**2k**) as a pale yellow solid (0.0624 g, 82%). Mp 138–140 °C; IR (neat) 3028, 1659, 1435, 1285, 1196, 1111, 926, 717 cm^–1^; ^1^H NMR (400 MHz, CDCl_3_) δ 7.45–7.54 (m, 6H), 7.55–7.64 (m, 3H), 7.65–7.74 (m, 4H), 7.76–7.89 (m, 6H); ^13^C{^1^H} NMR (101 MHz, CDCl_3_) δ 128.6 (2 × CH), 128.8 (d, ^3^*J*_CP_ = 12.2 Hz, 4 × CH), 129.8 (d, ^3^*J*_CP_ = 12.1 Hz, 2 × CH), 130.3 (2 × CH), 132.0 (d, ^1^*J*_CP_ = 105.1 Hz, 2 × C), 132.2 (d, ^2^*J*_CP_ = 10.0 Hz, 6 × CH), 132.4 (d, ^4^*J*_CP_ = 2.8 Hz, 2 × CH), 133.2 (CH), 136.9 (C), 137.1 (d, ^1^*J*_CP_ = 101.3 Hz, C), 140.7 (d, ^4^*J*_CP_ = 2.8 Hz, C), 196.2 (d, ^5^*J*_CP_ = 0.8 Hz, C); MS (ESI) *m*/*z* 405 (M + Na^+^, 100); HRMS (ESI) *m*/*z*: [M + Na]^+^ calcd for C_25_H_19_NaO_2_P 405.1015; found 405.1017.

### (4-Methoxycarbonylphenyl)diphenylphosphine Oxide (**2l**)^38^

The reaction was carried out according to the previously described general procedure for (4-methylphenyl)diphenylphosphine oxide (**2a**) using sodium iodide (0.0300 g, 0.200 mmol), methyl 4-nonafluorobutanesulfonyloxybenzoate (**1l**) (0.0870 g, 0.200 mmol), anhydrous *N*,*N*′-dimethylformamide (1.2 mL), diphenylphosphine oxide (0.0610 g, 0.302 mmol), palladium(II) acetate (0.00450 g, 0.0200 mmol), and triethylamine (0.110 mL, 0.790 mmol). The reaction mixture was stirred at 120 °C for 4 h. The reaction mixture was cooled to room temperature and diluted with ethyl acetate (10 mL). The organic layer was washed with 2 M aqueous lithium chloride solution (3 × 10 mL) and then water (2 × 10 mL). The organic layer was dried (MgSO_4_), filtered, and concentrated *in vacuo*. The crude material was purified by flash column chromatography eluting with 2% methanol in diethyl ether to give (4-methoxycarbonylphenyl)diphenylphosphine oxide (**2l**) as an off-white solid (0.0518 g, 77%). Mp 101–103 °C (lit.^38^ 104–105 °C); ^1^H NMR (400 MHz, CDCl_3_) δ 3.93 (s, 3H), 7.42–7.51 (m, 4H), 7.56 (ttd, *J* = 7.5, 1.6, 1.5 Hz, 2H), 7.61–7.70 (m, 4H), 7.76 (dd, *J* = 11.6, 8.4 Hz, 2H), 8.11 (dd, *J* = 8.4, 2.4 Hz, 2H); ^13^C{^1^H} NMR (101 MHz, CDCl_3_) δ 52.6 (CH_3_), 128.8 (d, ^3^*J*_CP_ = 12.2 Hz, 4 × CH), 129.5 (d, ^3^*J*_CP_ = 12.1 Hz, 2 × CH), 132.0 (d, ^1^*J*_CP_ = 105.1 Hz, 2 × C), 132.2 (d, ^2^*J*_CP_ = 10.2 Hz, 4 × CH), 132.3 (d, ^2^*J*_CP_ = 10.8 Hz, 2 × CH), 132.3 (d, ^4^*J*_CP_ = 2.7 Hz, 2 × CH), 133.3 (d, ^4^*J*_CP_ = 2.7 Hz, C), 137.8 (d, ^1^*J*_CP_ = 101.2 Hz, C), 166.4 (d, ^5^*J*_CP_ = 0.8 Hz, C); MS (ESI) *m*/*z* 359 (M + Na^+^, 100).

### (4-Cyanophenyl)diphenylphosphine Oxide (**2m**)^37^

The reaction was carried out according to the previously described general procedure for (4-methylphenyl)diphenylphosphine oxide (**2a**) using sodium iodide (0.0300 g, 0.200 mmol), 4-cyanophenyl nonafluorobutanesulfonate (**1m**) (0.0802 g, 0.200 mmol), anhydrous *N*,*N*′-dimethylformamide (1.2 mL), diphenylphosphine oxide (0.0610 g, 0.302 mmol), palladium(II) acetate (0.00450 g, 0.0200 mmol), and triethylamine (0.110 mL, 0.790 mmol). The reaction mixture was stirred at 120 °C for 4 h. The reaction mixture was cooled to room temperature and diluted with ethyl acetate (10 mL). The organic layer was washed with 2 M aqueous lithium chloride solution (3 × 10 mL) and then water (2 × 10 mL). The organic layer was dried (MgSO_4_), filtered, and concentrated *in vacuo*. The crude material was purified by flash column chromatography eluting with 2% methanol in diethyl ether to give (4-cyanophenyl)diphenylphosphine oxide (**2m**) as a pale brown oil (0.0536 g, 89%). Spectroscopic data were consistent with previously published data.^37^^1^H NMR (400 MHz, CDCl_3_) δ 7.44–7.54 (m, 4H), 7.59 (ttd, *J* = 7.5, 1.7, 1.6 Hz, 2H), 7.61–7.70 (m, 4H), 7.71–7.84 (m, 4H); ^13^C{^1^H} NMR (101 MHz, CDCl_3_) δ 115.7 (d, ^4^*J*_CP_ = 3.2 Hz, C), 118.0 (d, ^5^*J*_CP_ = 1.6 Hz, C), 128.9 (d, ^3^*J*_CP_ = 12.4 Hz, 4 × CH), 131.3 (d, ^1^*J*_CP_ = 105.7 Hz, 2 × C), 132.1 (d, ^3^*J*_CP_ = 11.4 Hz, 2 × CH), 132.1 (d, ^2^*J*_CP_ = 10.3 Hz, 4 × CH), 132.7 (d, ^4^*J*_CP_ = 3.1 Hz, 2 × CH), 132.7 (d, ^2^*J*_CP_ = 10.1 Hz, 2 × CH), 138.6 (d, ^1^*J*_CP_ = 99.5 Hz, C); MS (ESI) *m*/*z* 326 (M + Na^+^, 100).

### [4-(Trifluoromethyl)phenyl]diphenylphosphine Oxide (**2n**)^37^

The reaction was carried out according to the previously described general procedure for (4-methylphenyl)diphenylphosphine oxide (**2a**) using sodium iodide (0.0300 g, 0.200 mmol), 4-(trifluoromethyl)phenyl nonafluorobutanesulfonate (**1n**) (0.0890 g, 0.200 mmol), anhydrous *N*,*N*′-dimethylformamide (1.2 mL), diphenylphosphine oxide (0.0610 g, 0.302 mmol), palladium(II) acetate (0.00450 g, 0.0200 mmol), and triethylamine (0.110 mL, 0.790 mmol). The reaction mixture was stirred at 120 °C for 4 h. The reaction mixture was cooled to room temperature and diluted with ethyl acetate (10 mL). The organic layer was washed with 2 M aqueous lithium chloride solution (3 × 10 mL) and then water (2 × 10 mL). The organic layer was dried (MgSO_4_), filtered, and concentrated *in vacuo*. The crude material was purified by flash column chromatography eluting with 1% methanol in diethyl ether to give [4-(trifluoromethyl)phenyl]diphenylphosphine oxide (**2n**) as a pale brown oil (0.0604 g, 87%). Spectroscopic data were consistent with previously published data.^37^^1^H NMR (400 MHz, CDCl_3_) δ 7.49 (td, *J* = 7.4, 2.4 Hz, 4H), 7.58 (t, *J* = 7.4 Hz, 2H), 7.66 (dd, *J* = 12.4, 7.4 Hz, 4H), 7.72 (dd, *J* = 8.4, 2.4 Hz, 2H), 7.82 (dd, *J* = 11.2, 8.4 Hz, 2H); ^13^C{^1^H} NMR (101 MHz, CDCl_3_) δ 123.7 (q, ^1^*J*_CF_ = 273.6 Hz, CF_3_), 125.5 (dq, ^3^*J*_CP_ = 12.0 Hz, ^3^*J*_CF_ = 3.8 Hz, 2 × CH), 128.9 (d, ^3^*J*_CP_ = 12.3 Hz, 4 × CH), 131.8 (d, ^1^*J*_CP_ = 105.4 Hz, 2 × C), 132.2 (d, ^2^*J*_CP_ = 10.1 Hz, 4 × CH), 132.5 (d, ^4^*J*_CP_ = 2.8 Hz, 2 × CH), 132.7 (d, ^2^*J*_CP_ = 10.1 Hz, 2 × CH), 133.8 (qd, ^2^*J*_CF_ = 32.9 Hz, ^4^*J*_CP_ = 2.9 Hz, C), 137.3 (d, ^1^*J*_CP_ = 101.2 Hz, C); MS (ESI) *m*/*z* 369 (M + Na^+^, 100).

### (2-Fluorophenyl)diphenylphosphine Oxide (**2o**)

The reaction was carried out according to the previously described general procedure for (4-methylphenyl)diphenylphosphine oxide (**2a**) using sodium iodide (0.0300 g, 0.200 mmol), 2-fluorophenyl nonafluorobutanesulfonate (**1o**) (0.0790 g, 0.200 mmol), anhydrous *N*,*N*′-dimethylformamide (1.2 mL), diphenylphosphine oxide (0.0610 g, 0.302 mmol), palladium(II) acetate (0.00450 g, 0.0200 mmol), and triethylamine (0.110 mL, 0.790 mmol). The reaction mixture was stirred at 120 °C for 4 h. The reaction mixture was cooled to room temperature and diluted with ethyl acetate (10 mL). The organic layer was washed with 2 M aqueous lithium chloride solution (3 × 10 mL) and then water (2 × 10 mL). The organic layer was dried (MgSO_4_), filtered, and concentrated *in vacuo*. The crude material was purified by flash column chromatography eluting with 2% methanol in diethyl ether to give (2-fluorophenyl)diphenylphosphine oxide (**2o**) as a pale yellow solid (0.0303 g, 51%). Mp 119–121 °C; IR (neat) 3010, 1601, 1437, 1273, 1191, 1119, 823, 757 cm^–1^; ^1^H NMR (400 MHz, CDCl_3_) δ 7.05–7.13 (m, 1H), 7.31 (br t, *J* = 7.6 Hz, 1H), 7.47 (td, *J* = 7.4, 3.2 Hz, 4H), 7.52–7.61 (m, 3H), 7.73 (dd, *J* = 12.6, 7.4 Hz, 4H), 7.83–7.93 (m, 1H); ^13^C{^1^H} NMR (101 MHz, CDCl_3_) *δ* 116.2 (dd, ^2^*J*_CF_ = 22.8 Hz, ^3^*J*_CP_ = 5.6 Hz, CH), 120.4 (dd, ^1^*J*_CP_ = 100.6 Hz, ^2^*J*_CF_ = 18.6 Hz, C), 124.7 (dd, ^3^*J*_CP_ = 10.6, ^4^*J*_CF_ = 3.4 Hz, CH), 128.6 (d, ^3^*J*_CP_ = 12.6 Hz, 4 × CH), 131.9 (dd, ^2^*J*_CP_ = 10.6 Hz, ^5^*J*_CF_ = 2.0 Hz, 4 × CH), 132.2 (d, ^4^*J*_CP_ = 2.9 Hz, 2 × CH), 132.3 (d, ^1^*J*_CP_ = 108.6 Hz, 2 × C), 134.8–135.0 (m, 2 × CH), 163.1 (dd, ^1^*J*_CF_ = 251.3 Hz, ^2^*J*_CP_ = 2.1 Hz, C); MS (ESI) *m*/*z* 319 (M + Na^+^, 100); HRMS (ESI) *m*/*z*: [M + Na]^+^ calcd for C_18_H_14_FNaOP 319.0659; found 319.0654.

### (3-Chlorophenyl)diphenylphosphine Oxide (**2p**)

The reaction was carried out according to the previously described general procedure for (4-methylphenyl)diphenylphosphine oxide (**2a**) using sodium iodide (0.0300 g, 0.200 mmol), 3-chlorophenyl nonafluorobutanesulfonate (**1p**) (0.0820 g, 0.200 mmol), anhydrous *N*,*N*′-dimethylformamide (1.2 mL), diphenylphosphine oxide (0.0610 g, 0.302 mmol), palladium(II) acetate (0.00450 g, 0.0200 mmol), and triethylamine (0.110 mL, 0.790 mmol). The reaction mixture was stirred at 120 °C for 4 h. The reaction mixture was cooled to room temperature and diluted with ethyl acetate (10 mL). The organic layer was washed with 2 M aqueous lithium chloride solution (3 × 10 mL) and then water (2 × 10 mL). The organic layer was dried (MgSO_4_), filtered, and concentrated *in vacuo*. The crude material was purified by flash column chromatography eluting with 2% methanol in diethyl ether to give (3-chlorophenyl)diphenylphosphine oxide (**2p**) as an off-white solid (0.0544 g, 87%). Mp 99–101 °C; IR (neat) 3055, 1439, 1400, 1188, 1119, 1076, 795, 752, 721 cm^–1^; ^1^H NMR (400 MHz, CDCl_3_) δ 7.40 (td, *J* = 7.8, 3.3 Hz, 1H), 7.44–7.60 (m, 8H), 7.61–7.71 (m, 5H); ^13^C{^1^H} NMR (101 MHz, CDCl_3_) δ 128.8 (d, ^3^*J*_CP_ = 12.3 Hz, 4 × CH), 130.1 (d, ^3^*J*_CP_ = 13.0 Hz, CH), 130.3 (d, ^2^*J*_CP_ = 9.4 Hz, CH), 132.0 (d, ^1^*J*_CP_ = 105.4 Hz, 2 × C), 132.0 (d, ^2^*J*_CP_ = 10.7 Hz, CH), 132.2 (d, ^2^*J*_CP_ = 10.0 Hz, 4 × CH), 132.2 (d, ^4^*J*_CP_ = 1.8 Hz, CH), 132.4 (d, ^4^*J*_CP_ = 2.9 Hz, 2 × CH), 135.1 (d, ^3^*J*_CP_ = 15.7 Hz, C), 135.3 (d, ^1^*J*_CP_ = 101.6 Hz, C); MS (ESI) *m*/*z* 335 (M + Na^+^, 100); HRMS (ESI) *m*/*z*: [M + Na]^+^ calcd for C_18_H_14_^35^ClNaOP 335.0363; found 335.0365.

### 1,3-Bis(diphenylphosphoryl)benzene (**2q**)^39^

The reaction was carried out according to the previously described general procedure for (4-methylphenyl)diphenylphosphine oxide (**2a**) using sodium iodide (0.0300 g, 0.200 mmol), 3-bromophenyl nonafluorobutanesulfonate (**1q**) (0.0910 g, 0.200 mmol), anhydrous *N*,*N*′-dimethylformamide (1.2 mL), diphenylphosphine oxide (0.121 g, 0.600 mmol), palladium(II) acetate (0.00450 g, 0.0200 mmol), and triethylamine (0.110 mL, 0.790 mmol). The reaction mixture was stirred at 120 °C for 5 h. The reaction mixture was cooled to room temperature and diluted with ethyl acetate (10 mL). The organic layer was washed with 2 M aqueous lithium chloride solution (3 × 10 mL) and then water (2 × 10 mL). The organic layer was dried (MgSO_4_), filtered, and concentrated *in vacuo*. The crude material was purified by flash column chromatography eluting with 3% methanol in dichloromethane to give 1,3-bis(diphenylphosphoryl)benzene (**2q**) as a colorless oil (0.0545 g, 57%). Spectroscopic data were consistent with previously published data.^39^^1^H NMR (400 MHz, CDCl_3_) δ 7.40 (td, *J* = 7.6, 2.8 Hz, 8H), 7.52 (ttd, *J* = 7.4, 1.5, 1.3 Hz, 4H), 7.53–7.60 (m, 8H), 7.61 (t, *J* = 7.6 Hz, 1H), 7.68 (tt, *J* = 11.7, 1.2 Hz, 1H), 7.90–8.00 (m, 2H); ^13^C{^1^H} NMR (101 MHz, CDCl_3_) δ 128.7 (d, ^3^*J*_CP_ = 12.7 Hz, 8 × CH), 129.1 (t, ^3^*J*_CP_ = 11.3 Hz, CH), 131.8 (d, ^1^*J*_CP_ = 105.3 Hz, 4 × C), 132.1 (d, ^2^*J*_CP_ = 10.1 Hz, 8 × CH), 132.3 (d, ^4^*J*_CP_ = 2.8 Hz, 4 × CH), 133.8 (dd, ^1^*J*_CP_ = 102.1 Hz, ^3^*J*_CP_ 10.9 Hz, 2 × C), 135.5 (t, ^2^*J*_CP_ = 11.2 Hz, CH), 135.6 (dd, ^2^*J*_CP_ = 10.2 Hz, ^4^*J*_CP_ 3.3 Hz, 2 × CH); MS (ESI) *m*/*z* 501 (M + Na^+^, 100).

### Pyridin-2-yldiphenylphosphine Oxide (**2r**)^40^

The reaction was carried out according to the previously described general procedure for (4-methylphenyl)diphenylphosphine oxide (**2a**) using sodium iodide (0.0600 g, 0.400 mmol), pyridin-2-yl nonafluorobutanesulfonate (**1r**) (0.151 g, 0.400 mmol), anhydrous *N*,*N*′-dimethylformamide (2.4 mL), diphenylphosphine oxide (0.121 g, 0.600 mmol), palladium(II) acetate (0.00900 g, 0.0400 mmol), and triethylamine (0.220 mL, 1.58 mmol). The reaction mixture was stirred at 120 °C for 4 h. The reaction mixture was cooled to room temperature and diluted with ethyl acetate (10 mL). The organic layer was washed with 2 M aqueous lithium chloride solution (3 × 10 mL) and then water (2 × 10 mL). The organic layer was dried (MgSO_4_), filtered, and concentrated *in vacuo*. The crude material was purified by flash column chromatography eluting with 2% methanol in diethyl ether to give pyridin-2-yldiphenylphosphine oxide (**2r**) as a white solid (0.0673 g, 60%). Mp 101–103 °C (lit.^40^ 106–107 °C); ^1^H NMR (400 MHz, CDCl_3_) δ 7.34–7.55 (m, 7H), 7.80–7.86 (m, 1H), 7.88 (dd, *J* = 12.0, 8.0 Hz, 4H), 8.30 (t, *J* = 6.8 Hz, 1H), 8.72–8.81 (m, 1H); ^13^C{^1^H} NMR (101 MHz, CDCl_3_) δ 125.4 (d, ^4^*J*_CP_ = 3.2 Hz, CH), 128.5 (d, ^3^*J*_CP_ = 12.3 Hz, 4 × CH), 128.5 (d, ^3^*J*_CP_ = 19.0 Hz, CH), 132.0 (d, ^4^*J*_CP_ = 2.9 Hz, 2 × CH), 132.2 (d, ^2^*J*_CP_ = 9.5 Hz, 4 × CH), 132.3 (d, ^1^*J*_CP_ = 104.5 Hz, 2 × C), 136.3 (d, ^2^*J*_CP_ = 9.2 Hz, CH), 150.3 (d, ^3^*J*_CP_ = 19.2 Hz, CH), 156.5 (d, ^1^*J*_CP_ = 132.2 Hz, C); MS (ESI) *m*/*z* 302 (M + Na^+^, 100).

### Tris(*p*-tolyl)phosphine Oxide (**3a**)^41^

The reaction was carried out according to the previously described general procedure for (4-methylphenyl)diphenylphosphine oxide (**2a**) using sodium iodide (0.0600 g, 0.400 mmol), 4-methylphenyl nonafluorobutanesulfonate (**1a**) (0.156 g, 0.400 mmol), anhydrous *N*,*N*′-dimethylformamide (2.4 mL), bis(*p*-tolyl)phosphine oxide (0.138 g, 0.600 mmol), and palladium(II) acetate (0.00900 g, 0.0400 mmol). The reaction was heated to 120 °C and stirred for 4 h. The crude material was purified by flash column chromatography eluting with 2% methanol in diethyl ether to give tris(*p*-tolyl)phosphine oxide (**3a**) as a white solid (0.0810 g, 63%). Mp 135–137 °C (lit.^41^ 140 °C); ^1^H NMR (400 MHz, CDCl_3_) δ 2.39 (s, 9H), 7.24 (dd, *J* = 8.0, 2.4 Hz, 6H), 7.54 (dd, *J* = 12.0, 8.0 Hz, 4H); ^13^C{^1^H} NMR (101 MHz, CDCl_3_) δ 21.7 (d, ^5^*J*_CP_ = 1.4 Hz, 3 × CH_3_), 129.3 (d, ^3^*J*_CP_ = 12.4 Hz, 6 × CH), 129.9 (d, ^1^*J*_CP_ = 106.7 Hz, 3 × C), 132.2 (d, ^2^*J*_CP_ = 10.3 Hz, 6 × CH), 142.3 (d, ^4^*J*_CP_ = 2.8 Hz, 3 × C); MS (ESI) *m*/*z* 343 (M + Na^+^, 100).

### (4-Methylphenyl)di(*n*-butyl)phosphine Oxide (**3b**)

The reaction was carried out according to the previously described general procedure for (4-methylphenyl)diphenylphosphine oxide (**2a**) using sodium iodide (0.0600 g, 0.400 mmol), 4-methylphenyl nonafluorobutanesulfonate (**1a**) (0.156 g, 0.400 mmol), anhydrous *N*,*N*′-dimethylformamide (2.4 mL), di(*n*-butyl)phosphine oxide (0.0973 g, 0.600 mmol), tetrakis(triphenylphosphine)palladium(0) (0.0462 g, 0.0400 mmol), and triethylamine (0.220 mL, 1.58 mmol). The reaction mixture was heated to 120 °C and stirred for 5 h. The reaction mixture was cooled to room temperature, diluted with ethyl acetate (30 mL), and washed with water (3 × 30 mL). The organic layer was dried (MgSO_4_), filtered, and concentrated *in vacuo*. The crude material was purified by flash column chromatography eluting with 0–2% gradient of methanol in diethyl ether to give (4-methylphenyl)di(*n*-butyl)phosphine oxide (**3b**) as a white solid (0.0587 g, 58%). Mp 51–53 °C; IR (neat) 2926, 2865, 1462, 1163, 1109, 1054, 900, 808, 757 cm^–1^; ^1^H NMR (400 MHz, CDCl_3_) δ 0.85 (t, *J* = 7.2 Hz, 6H), 1.30–1.65 (m, 8H), 1.75–2.00 (m, 4H), 2.39 (s, 3H), 7.27 (dd, *J* = 8.2, 2.4 Hz, 2H), 7.56 (dd, *J* = 10.6, 8.2 Hz, 2H); ^13^C{^1^H} NMR (101 MHz, CDCl_3_) δ 13.7 (2 × CH_3_), 21.6 (d, ^5^*J*_CP_ = 1.2 Hz, CH_3_), 23.7 (d, ^2^*J*_CP_ = 4.1 Hz, 2 × CH_2_), 24.2 (d, ^3^*J*_CP_ = 14.4 Hz, 2 × CH_2_), 29.9 (d, ^1^*J*_CP_ = 68.9 Hz, 2 × CH_2_), 129.4 (d, ^3^*J*_CP_ = 11.4 Hz, 2 × CH), 129.5 (d, ^1^*J*_CP_ = 94.5 Hz, C), 130.5 (d, ^2^*J*_CP_ = 9.0 Hz, 2 × CH), 141.8 (d, ^4^*J*_CP_ = 2.6 Hz, C); MS (ESI) *m*/*z* 275 (M + Na^+^, 100); HRMS (ESI) *m*/*z*: [M + Na]^+^ calcd for C_15_H_25_NaOP 275.1535; found 275.1534.

### Ethyl (4-methylphenyl)phenylphosphinate (**3c**)^42^

The reaction was carried out according to the previously described general procedure for (4-methylphenyl)diphenylphosphine oxide (**2a**) using sodium iodide (0.0300 g, 0.200 mmol), 4-methylphenyl nonafluorobutanesulfonate (**1a**) (0.0780 g, 0.200 mmol), anhydrous *N*,*N*′-dimethylformamide (1.2 mL), tetrakis(triphenylphosphine)palladium(0) (0.0231 g, 0.0200 mmol), ethyl phenylphosphinate (0.0450 mL, 0.299 mmol), and triethylamine (0.110 mL, 0.790 mmol). The reaction was heated to 80 °C and stirred for 4 h. The reaction mixture was cooled to room temperature, diluted with ethyl acetate (15 mL) and washed with water (3 × 15 mL). The organic layer was dried (MgSO_4_), filtered, and concentrated *in vacuo*. The crude material was purified by flash column chromatography eluting with 1% methanol in diethyl ether to give ethyl (4-methylphenyl)phenylphosphinate (**3c**) as a colorless oil (0.0381 g, 73%). Spectroscopic data were consistent with previously published data.^42^^1^H NMR (400 MHz, CDCl_3_) δ 1.36 (t, *J* = 7.2 Hz, 3H), 2.38 (s, 3H), 4.09 (quin., *J* = 7.2 Hz, 2H), 7.25 (dd, *J* = 8.0, 3.2 Hz, 2H), 7.39–7.47 (m, 2H), 7.50 (ttd, *J* = 7.4, 1.9, 1.3 Hz, 1H), 7.70 (dd, *J* = 12.0, 8.0 Hz, 2H), 7.76–7.84 (m, 2H); ^13^C{^1^H} NMR (101 MHz, CDCl_3_) δ 16.7 (d, ^3^*J*_CP_ = 6.7 Hz, CH_3_), 21.8 (d, ^5^*J*_CP_ = 1.3 Hz, CH_3_), 61.1 (d, ^2^*J*_CP_ = 5.9 Hz, CH_2_), 128.6 (d, ^1^*J*_CP_ = 139.8 Hz, C), 128.6 (d, ^3^*J*_CP_ = 13.2 Hz, 2 × CH), 129.4 (d, ^3^*J*_CP_ = 13.5 Hz, 2 × CH), 131.7 (d, ^2^*J*_CP_ = 10.0 Hz, 2 × CH), 131.9 (d, ^2^*J*_CP_ = 10.6 Hz, 2 × CH), 132.1 (d, ^4^*J*_CP_ = 2.8 Hz, CH), 132.2 (d, ^1^*J*_CP_ = 137.6 Hz, C), 142.7 (d, ^4^*J*_CP_ = 2.9 Hz, C); MS (ESI) *m*/*z* 283 (M + Na^+^, 100).

### Diethyl (4-methylphenyl)phosphonate (**3d**)^42^

The reaction was carried out according to the previously described general procedure for (4-methylphenyl)diphenylphosphine oxide (**2a**) using sodium iodide (0.105 g, 0.700 mmol), 4-methylphenyl nonafluorobutanesulfonate (**1a**) (0.273 g, 0.700 mmol), anhydrous *N*,*N*′-dimethylformamide (4 mL), tetrakis(triphenylphosphine)palladium(0) (0.0809 g, 0.0700 mmol), diethyl phosphite (0.136 mL, 1.06 mmol), and triethylamine (0.390 mL, 2.80 mmol). The reaction mixture was stirred at 80 °C for 6 h. The crude material was purified by flash column chromatography eluting with 1% methanol in diethyl ether to give diethyl (4-methylphenyl)phosphonate (**3d**) as a yellow oil (0.107 g, 67%). Spectroscopic data were consistent with previously published data.^42^^1^H NMR (400 MHz, CDCl_3_) δ 1.31 (t, *J* = 7.2 Hz, 6H), 2.39 (s, 3H), 3.99–4.18 (m, 4H), 7.26 (ddd, *J* = 8.0, 4.0, 0.4 Hz, 2H), 7.69 (dd, *J* = 13.2, 8.0 Hz, 2H); ^13^C{^1^H} NMR (101 MHz, CDCl_3_) δ 16.5 (d, ^3^*J*_CP_ = 6.6 Hz, 2 × CH_3_), 21.8 (d, ^5^*J*_CP_ = 1.4 Hz, CH_3_), 62.1 (d, ^2^*J*_CP_ = 5.4 Hz, 2 × CH_2_), 125.2 (d, ^1^*J*_CP_ = 190.8 Hz, C), 129.3 (d, ^3^*J*_CP_ = 15.5 Hz, 2 × CH), 132.0 (d, ^2^*J*_CP_ = 10.4 Hz, 2 × CH), 143.0 (d, ^4^*J*_CP_ = 3.1 Hz, C); MS (ESI) *m*/*z* 251 (M + Na^+^, 100).

### 1-Pyrenyl nonafluorobutanesulfonate (**5**)

The reaction was carried out according to the previously described procedure for 4-methylphenyl nonafluorobutanesulfonate (**1a**) using pyren-1-ol (**4**) (0.327 g, 1.50 mmol), anhydrous dichloromethane (5 mL), triethylamine (0.530 mL, 3.80 mmol) and perfluoro-1-butanesulfonyl fluoride (0.410 mL, 2.28 mmol). The reaction mixture was stirred at room temperature for 3 h. The crude material was purified by flash column chromatography eluting with 5% diethyl ether in hexane to give 1-pyrenyl nonafluorobutanesulfonate (**5**) as a white solid (0.654 g, 87%). Mp 122–124 °C; IR (neat) 3049, 1598, 1416, 1236, 1193, 1135, 1031, 904, 844 cm^–1^; ^1^H NMR (400 MHz, CDCl_3_) δ 7.96 (d, *J* = 8.4 Hz, 1H), 8.01–8.12 (m, 3H), 8.15 (d, *J* = 8.4 Hz, 1H), 8.19–8.30 (m, 4H); ^13^C{^1^H} NMR (101 MHz, CDCl_3_) δ 118.7 (CH), 119.5 (CH), 123.9 (C), 124.1 (C), 125.1 (CH), 125.8 (C), 126.3 (CH), 126.6 (CH), 126.8 (CH), 127.0 (CH), 128.6 (CH), 129.9 (CH), 130.8 (C), 131.0 (C), 131.1 (C), 142.9 (C); MS (ESI) *m*/*z* 523 (M + Na^+^, 100); HRMS (ESI) *m*/*z*: [M + Na]^+^ calcd for C_20_H_9_F_9_NaO_3_S 523.0021; found 523.0024.

### (1-Pyrenyl)diphenylphosphine Oxide (**6**)^18^

The reaction was carried out according to the previously described general procedure for (4-methylphenyl)diphenylphosphine oxide (**2a**) using sodium iodide (0.0300 g, 0.200 mmol), 1-pyrenyl nonafluorobutanesulfonate (**5**) (0.100 g, 0.200 mmol), diphenylphosphine oxide (0.0610 g, 0.302 mmol), palladium(II) acetate (0.00450 g, 0.0200 mmol), anhydrous *N*,*N*′-dimethylformamide (1.2 mL), and triethylamine (0.110 mL, 0.790 mmol). The reaction mixture was heated to 120 °C and stirred for 4 h. The reaction mixture was cooled to room temperature, diluted with ethyl acetate (15 mL), and washed with water (3 × 15 mL). The organic layer was dried (MgSO_4_), filtered, and concentrated *in vacuo*. The crude material was purified by flash column chromatography eluting with 2% methanol in diethyl ether to give (1-pyrenyl)diphenylphosphine oxide (**6**) as an off-white solid (0.0588 g, 73%). Mp 233–235 °C. Spectroscopic data were consistent with previously published data.^18^^1^H NMR (400 MHz, CDCl_3_) δ 7.47 (td, *J* = 7.4, 2.9 Hz, 4H), 7.57 (ttd, *J* = 7.4, 1.7, 1.3 Hz, 2H), 7.68–7.80 (m, 5H), 8.00–8.11 (m, 4H), 8.19 (d, *J* = 9.2 Hz, 1H), 8.24 (t, *J* = 7.6 Hz, 2H), 8.94 (d, *J* = 9.2 Hz, 1H); ^13^C{^1^H} NMR (101 MHz, CDCl_3_) δ 123.7 (d, ^3^*J*_CP_ = 13.7 Hz, CH), 124.3 (d, ^5^*J*_CP_ = 0.9 Hz, C), 125.3 (d, ^1^*J*_CP_ = 103.6 Hz, C), 125.3 (d, ^3^*J*_CP_ = 10.3 Hz, C), 126.3 (CH), 126.5 (d, ^2^*J*_CP_ = 6.5 Hz, CH), 126.6 (2 × CH), 127.3 (d, ^5^*J*_CP_ = 0.8 Hz, CH), 128.8 (d, ^3^*J*_CP_ = 12.2 Hz, 4 × CH), 129.0 (CH), 130.0 (CH), 130.6 (C), 131.2 (d, ^5^*J*_CP_ = 0.8 Hz, C), 131.3 (d, ^3^*J*_CP_ = 12.2 Hz, CH), 132.0 (d, ^4^*J*_CP_ = 2.8 Hz, 2 × CH), 132.4 (d, ^2^*J*_CP_ = 9.9 Hz, 4 × CH), 133.5 (d, ^1^*J*_CP_ = 104.7 Hz, 2 × C), 134.3 (d, ^2^*J*_CP_ = 8.2 Hz, C), 134.4 (d, ^4^*J*_CP_ = 2.6 Hz, C); MS (ESI) *m*/*z* 425 (M + Na^+^, 100).

### Methyl (2*S*)-2-[(benzyloxycarbonyl)amino]-3-[(phenylnonafluorobutanesulfonate)-4′-yl]propanoate (**8**)

The reaction was carried out according to the previously described procedure for 4-methylphenyl nonafluorobutanesulfonate (**1a**) using methyl (2*S*)-2-[(benzyloxycarbonyl)amino]-3-(4-hydroxyphenyl)propanoate (**7**) (0.988 g, 3.00 mmol), anhydrous dichloromethane (10 mL), triethylamine (1.05 mL, 7.53 mmol), and perfluoro-1-butanesulfonyl fluoride (0.810 mL, 4.50 mmol). The reaction mixture was stirred at room temperature for 2 h. The crude material was purified by flash column chromatography eluting with 50% diethyl ether in hexane to give methyl (2*S*)-2-[(benzyloxycarbonyl)amino]-3-[(phenylnonafluorobutanesulfonate)-4′-yl]propanoate (**8**) as a colorless oil which solidified upon standing (1.72 g, 94%). Mp 46–48 °C; IR (neat) 3341, 2959, 1717, 1501, 1423, 1200, 1142, 1015, 891 cm^–1^; [α]_D_^17^ −14.6 (*c* 0.5, MeOH); ^1^H NMR (400 MHz, CDCl_3_) δ 3.09 (dd, *J* = 14.0, 6.4 Hz, 1H), 3.19 (dd, *J* = 14.0, 5.6 Hz, 1H), 3.72 (s, 3H), 4.60–4.72 (m, 1H), 5.07 (d, *J* = 12.2 Hz, 1H), 5.12 (d, *J* = 12.2 Hz, 1H), 5.26 (d, *J* = 8.0 Hz, 1H), 7.18 (br s, 4H), 7.28–7.42 (m, 5H); ^13^C{^1^H} NMR (101 MHz, CDCl_3_) δ 37.8 (CH_2_), 52.6 (CH_3_), 54.8 (CH), 67.3 (CH_2_), 121.6 (2 × CH), 128.3 (2 × CH), 128.5 (CH), 128.7 (2 × CH), 131.2 (2 × CH), 136.2 (C), 136.7 (C), 149.0 (C), 155.6 (C), 171.6 (C); MS (ESI) *m*/*z* 634 (M + Na^+^, 100); HRMS (ESI) *m*/*z*: [M + Na]^+^ calcd for C_22_H_18_F_9_NNaO_7_S 634.0552; found 634.0549.

### Methyl (2*S*)-2-[(benzyloxycarbonyl)amino]-3-[(diethylphenylphosphonate)-4′-yl]propanoate (**9**) Using Pd(PPh_3_)_4_ (10 mol %)

The reaction was carried out according to the previously described general procedure for (4-methylphenyl)diphenylphosphine oxide (**2a**) using sodium iodide (0.150 g, 1.00 mmol), methyl (2*S*)-2-[(benzyloxycarbonyl)amino]-3-[(phenylnonafluorobutanesulfonate)-4′-yl]propanoate (**8**) (0.611 g, 1.00 mmol), anhydrous *N*,*N*′-dimethylformamide (6 mL), diethyl phosphite (0.193 mL, 1.50 mmol), tetrakis(triphenylphosphine)palladium(0) (0.116 g, 0.100 mmol), and triethylamine (0.557 mL, 4.00 mmol). The reaction mixture was heated to 80 °C and stirred for 6 h. The reaction mixture was cooled to room temperature, diluted with ethyl acetate (50 mL), and washed with water (3 × 50 mL). The organic layer was dried (MgSO_4_), filtered, and concentrated *in vacuo*. The crude material was purified by flash column chromatography eluting with 2% methanol and 2% toluene in diethyl ether to give methyl (2*S*)-2-[(benzyloxycarbonyl)amino]-3-[(diethylphenylphosphonate)-4′-yl]propanoate (**9**) as a colorless oil (0.326 g, 72%). IR (neat) 3248, 2983, 1714, 1533, 1225, 1017, 961, 745 cm^–1^; [α]_D_^23^ +50.1 (*c* 0.1, CHCl_3_); ^1^H NMR (400 MHz, CDCl_3_) δ 1.31 (t, *J* = 7.0 Hz, 6H), 3.11 (dd, *J* = 14.0, 6.0 Hz, 1H), 3.20 (dd, *J* = 14.0, 5.6 Hz, 1H), 3.71 (s, 3H), 4.00–4.20 (m, 4H), 4.61–4.75 (m, 1H), 5.07 (d, *J* = 12.4 Hz, 1H), 5.11 (d, *J* = 12.4 Hz, 1H), 5.28 (d, *J* = 8.0 Hz, 1H), 7.20 (dd, *J* = 8.2, 3.6 Hz, 2H), 7.27–7.42 (m, 5H), 7.71 (dd, *J* = 13.2, 8.2 Hz, 2H); ^13^C{^1^H} NMR (101 MHz, CDCl_3_) δ 16.5 (d, ^3^*J*_CP_ = 6.6 Hz, 2 × CH_3_), 38.3 (CH_2_), 52.6 (CH_3_), 54.7 (CH), 62.2 (d, ^2^*J*_CP_ = 5.6 Hz, 2 × CH_2_), 67.2 (CH_2_), 127.3 (d, ^1^*J*_CP_ = 190.3 Hz, C), 128.2 (2 × CH), 128.4 (CH), 128.7 (2 × CH), 129.6 (d, ^3^*J*_CP_ = 15.4 Hz, 2 × CH), 132.1 (d, ^2^*J*_CP_ = 10.3 Hz, 2 × CH), 136.2 (C), 140.7 (d, ^4^*J*_CP_ = 2.8 Hz, C), 155.7 (C), 171.7 (C); MS (ESI) *m*/*z* 472 (M + Na^+^, 100); HRMS (ESI) *m*/*z*: [M + Na]^+^ calcd for C_22_H_28_NNaO_7_P 472.1496; found 472.1498.

### Methyl (2*S*)-2-[(benzyloxycarbonyl)amino]-3-[(diethylphenylphosphonate)-4′-yl]propanoate (**9**) Using Pd(PPh_3_)_4_ (5 mol %)

The reaction was carried out according to the previously described general procedure for (4-methylphenyl)diphenylphosphine oxide (**2a**) using sodium iodide (0.150 g, 1.00 mmol), methyl (2*S*)-2-[(benzyloxycarbonyl)amino]-3-[(phenylnonafluorobutanesulfonate)-4′-yl]propanoate (**8**) (0.611 g, 1.00 mmol), anhydrous *N*,*N*′-dimethylformamide (6 mL), diethyl phosphite (0.193 mL, 1.50 mmol), tetrakis(triphenylphosphine)palladium(0) (0.058 g, 0.05 mmol), and triethylamine (0.557 mL, 4.00 mmol). The reaction mixture was heated to 80 °C and stirred for 7 h. The reaction mixture was cooled to room temperature, diluted with ethyl acetate (50 mL), and washed with water (3 × 50 mL). The organic layer was dried (MgSO_4_), filtered, and concentrated *in vacuo*. The crude material was purified by flash column chromatography eluting with 2% methanol and 2% toluene in diethyl ether to give methyl (2*S*)-2-[(benzyloxycarbonyl)amino]-3-[(diethylphenylphosphonate)-4′-yl]propanoate (**9**) as a colorless oil (0.294 g, 65%). Spectroscopic data were consistent as described above.

### (2*S*)-2-Amino-3-[(phenylphosphonate)-4′-yl]propanoic Hydrochloride (**10**)

Methyl (2*S*)-2-[(benzyloxycarbonyl)amino]-3-[(diethylphenylphosphonate)-4′-yl]propanoate (**9**) (0.209 g, 0.465 mmol) was suspended in 6 M aqueous hydrochloric acid solution (1.70 mL, 10.2 mmol) and stirred under reflux for 6 h. The reaction mixture was cooled to room temperature and concentrated *in vacuo*. The crude material was purified by trituration with diethyl ether to give (2*S*)-2-amino-3-[(phenylphosphonate)-4′-yl]propanoic hydrochloride (**10**) as a white solid (0.115 g, 88%). Mp 218–220 °C; IR (neat) 2745, 1729, 1605, 1501, 1407, 1135, 921 cm^–1^; [α]_D_^25^ +3.5 (*c* 0.1, H_2_O); ^1^H NMR (400 MHz, D_2_O) δ 3.25 (dd, *J* = 14.6, 7.6 Hz, 1H), 3.41 (dd, *J* = 14.6, 5.6 Hz, 1H), 4.32 (dd, *J* = 7.6, 5.6 Hz, 1H), 7.43 (dd, *J* = 8.0, 3.2 Hz, 2H), 7.71 (dd, *J* = 12.8, 8.0 Hz, 2H); ^13^C{^1^H} NMR (101 MHz, D_2_O) δ 38.6 (CH_2_), 57.3 (CH), 132.3 (d, ^3^*J*_CP_ = 14.5 Hz, 2 × CH), 134.0 (d, ^2^*J*_CP_ = 10.2 Hz, 2 × CH), 136.2 (d, ^1^*J*_CP_ = 180.5 Hz, C), 140.3 (d, ^4^*J*_CP_ = 3.1 Hz, C), 174.7 (C); MS (ESI) *m*/*z* 246 (M + Na^+^, 100); HRMS (ESI) *m*/*z*: [M + Na]^+^ calcd for C_9_H_13_NO_5_P 246.0526; found 246.0527.

### (4-Methylphenyl)diphenylphosphine Oxide (**2a**) Using *p*-Tolyl Iodide (**11**)^32^

The reaction was carried out according to the previously described procedure for (4-methylphenyl)diphenylphosphine oxide (**2a**) using *p*-tolyl iodide (**11**) (0.0436 g, 0.200 mmol), anhydrous *N*,*N*′-dimethylformamide (1.2 mL), diphenylphosphine oxide (0.0610 g, 0.302 mmol), palladium(II) acetate (0.00450 g, 0.0200 mmol), and triethylamine (0.110 mL, 0.790 mmol). The reaction mixture was heated to 120 °C and stirred for 1.5 h. The crude material was purified by flash column chromatography eluting with 2% methanol in diethyl ether to give (4-methylphenyl)diphenylphosphine oxide (**2a**) as a white solid (0.0199 g, 34%). Spectroscopic data were consistent as described above.

## Supporting Information Available

^1^H and ^13^C NMR spectra of all compounds (PDF)
