# Supplementary material for: Palladium-Catalyzed C–P Bond-Forming Reactions of Aryl Nonaflates Accelerated by Iodide
Source: J Org Chem. 2021 Nov 2;86(23):17036–49. doi: 10.1021/acs.joc.1c02172 (PMC8650017; doi:10.1021/acs.joc.1c02172)
Supplement: Supplementary file 1 — jo1c02172_si_001.pdf [file jo1c02172_si_001.pdf]

Supporting Information for:

Palladium-Catalyzed C–P Bond Forming Reactions of Aryl Nonaflates Accelerated by Iodide

Holly McErlain, Leanne M. Riley and Andrew Sutherland*

School of Chemistry, The Joseph Black Building, University of Glasgow, Glasgow G12 8QQ, UK. Email: Andrew.Sutherland@glasgow.ac.uk.

Table of Contents

1. ^{1}H and $^{13}C\{^{1}H\}$ NMR Spectra for all Compounds S2–S91

1. ^{1}H and $^{13}C\{^{1}H\}$ NMR Spectra of All Compounds

$CDCl_3$
400 MHz

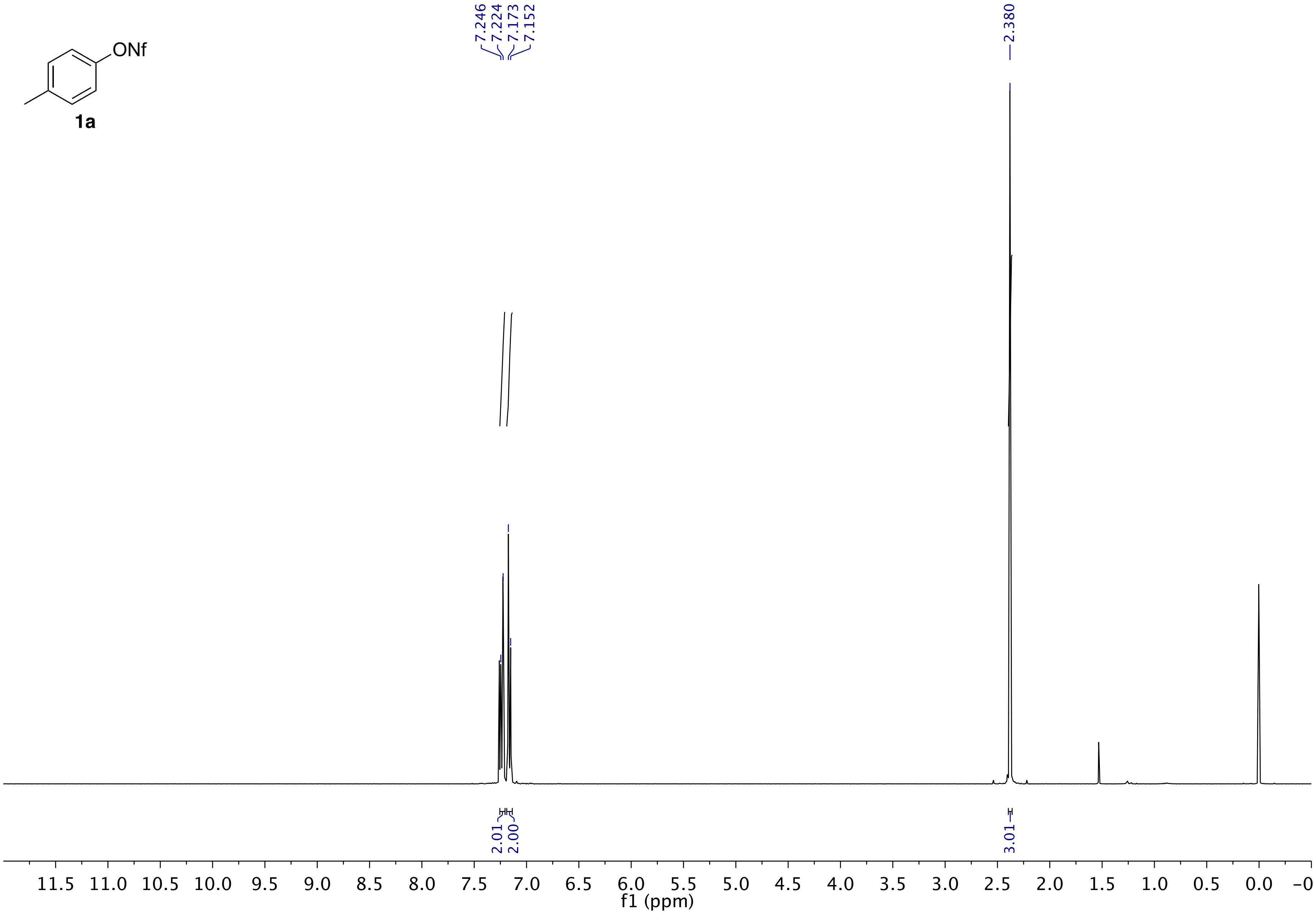

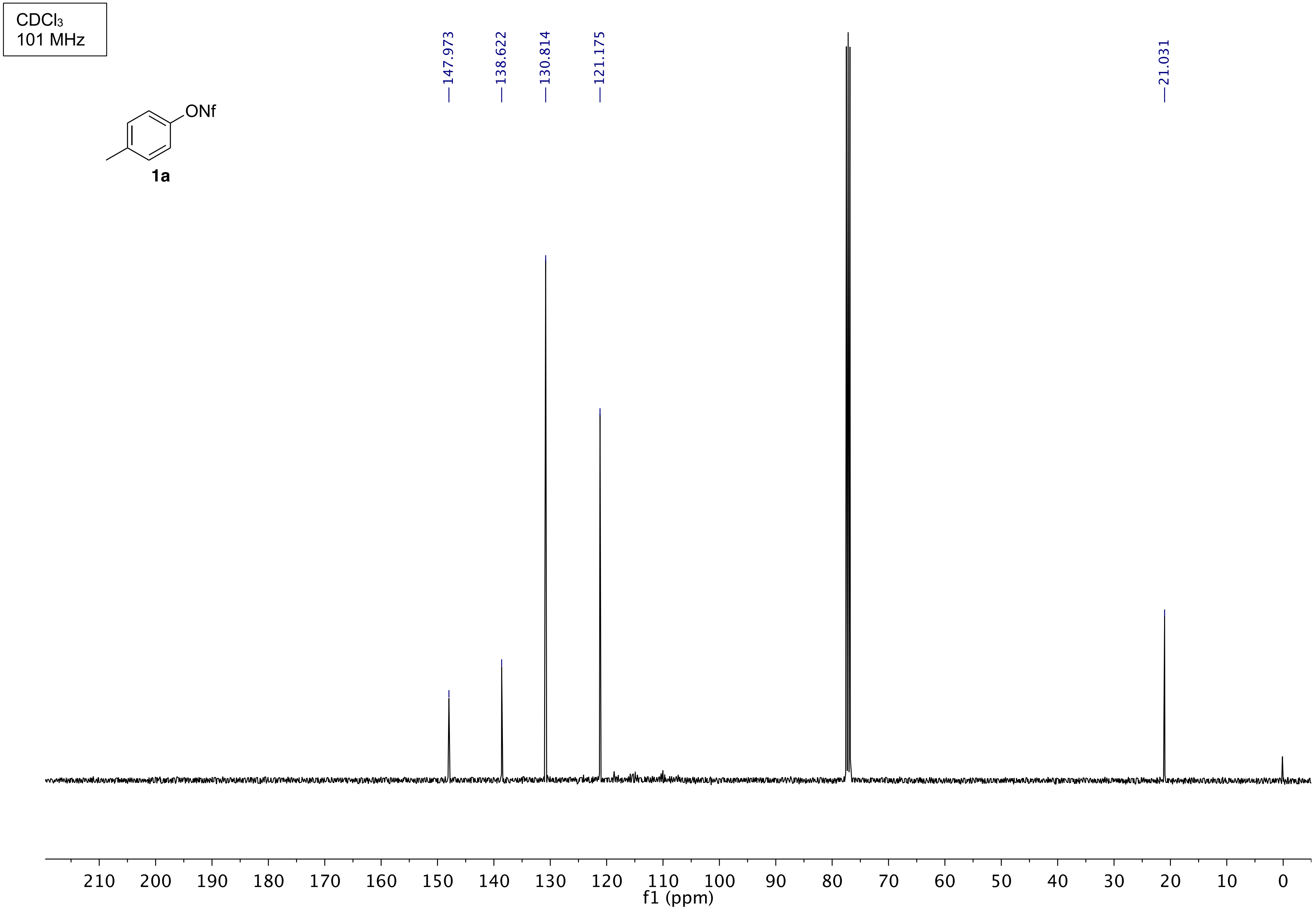

CDCl3
101 MHz
ONf
1a
147.973
138.622
130.814
121.175
21.031
210 200 190 180 170 160 150 140 130 120 110 100 90 80 70 60 50 40 30 20 10 0
f1 (ppm)

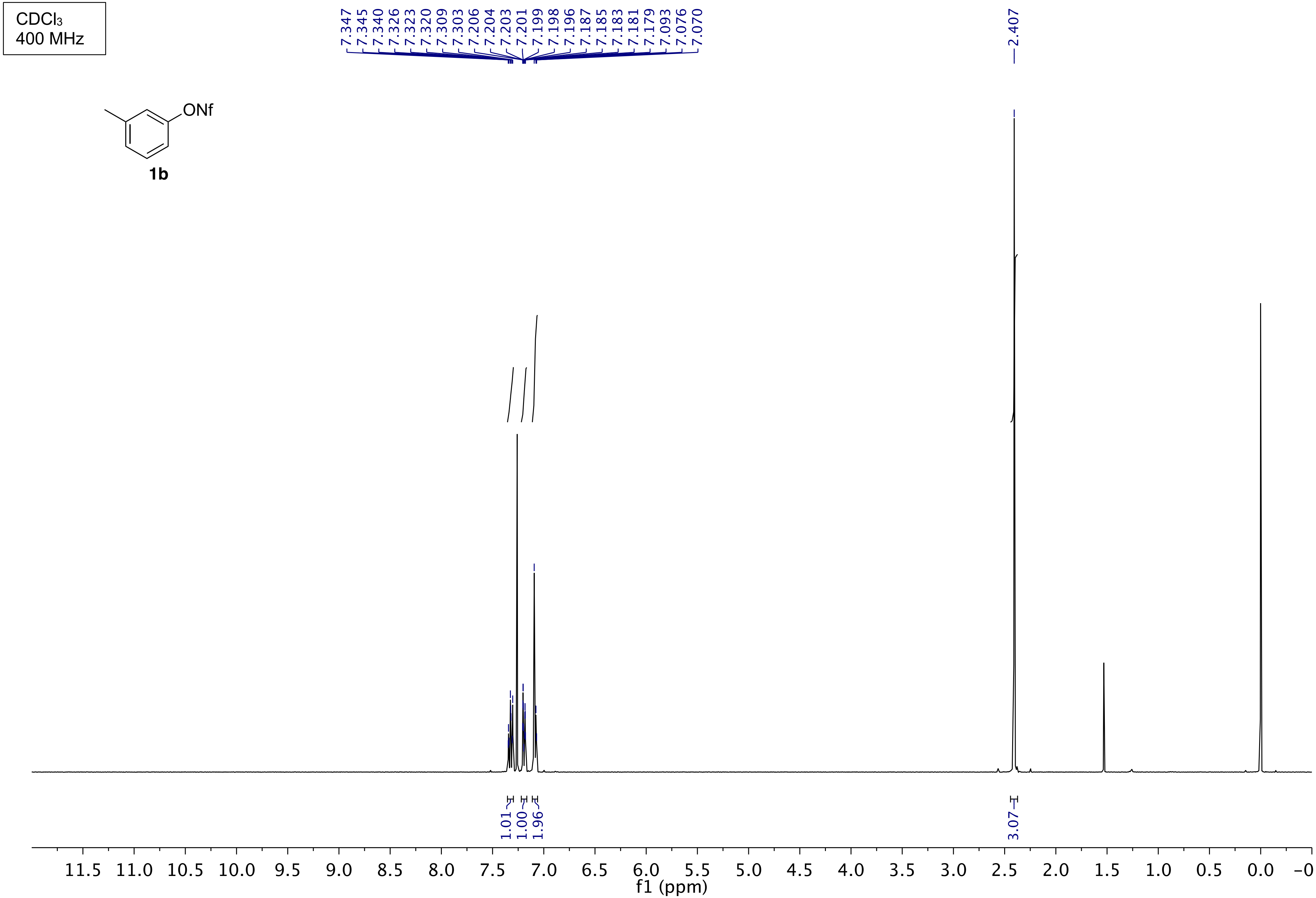

CDCl3
400 MHz
ONf
1b
7.347
7.345
7.340
7.326
7.323
7.320
7.309
7.303
7.206
7.204
7.203
7.201
7.199
7.198
7.196
7.187
7.185
7.183
7.181
7.179
7.093
7.076
7.070
2.407
1.01
1.00
1.96
3.07
11.5 11.0 10.5 10.0 9.5 9.0 8.5 8.0 7.5 7.0 6.5 6.0 5.5 5.0 4.5 4.0 3.5 3.0 2.5 2.0 1.5 1.0 0.5 0.0 -0
f1 (ppm)

CDCl3
101 MHz

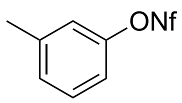

1b

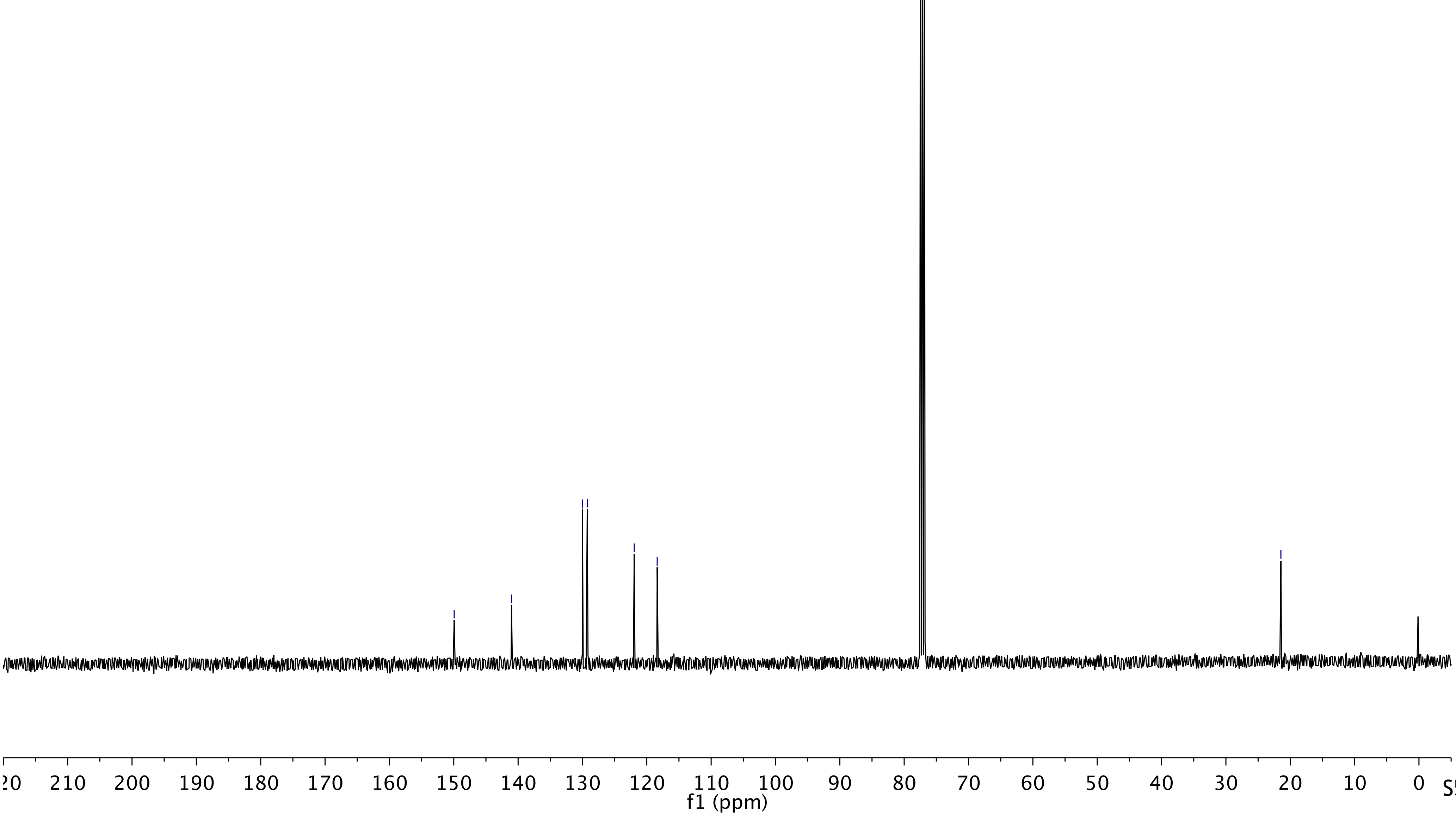

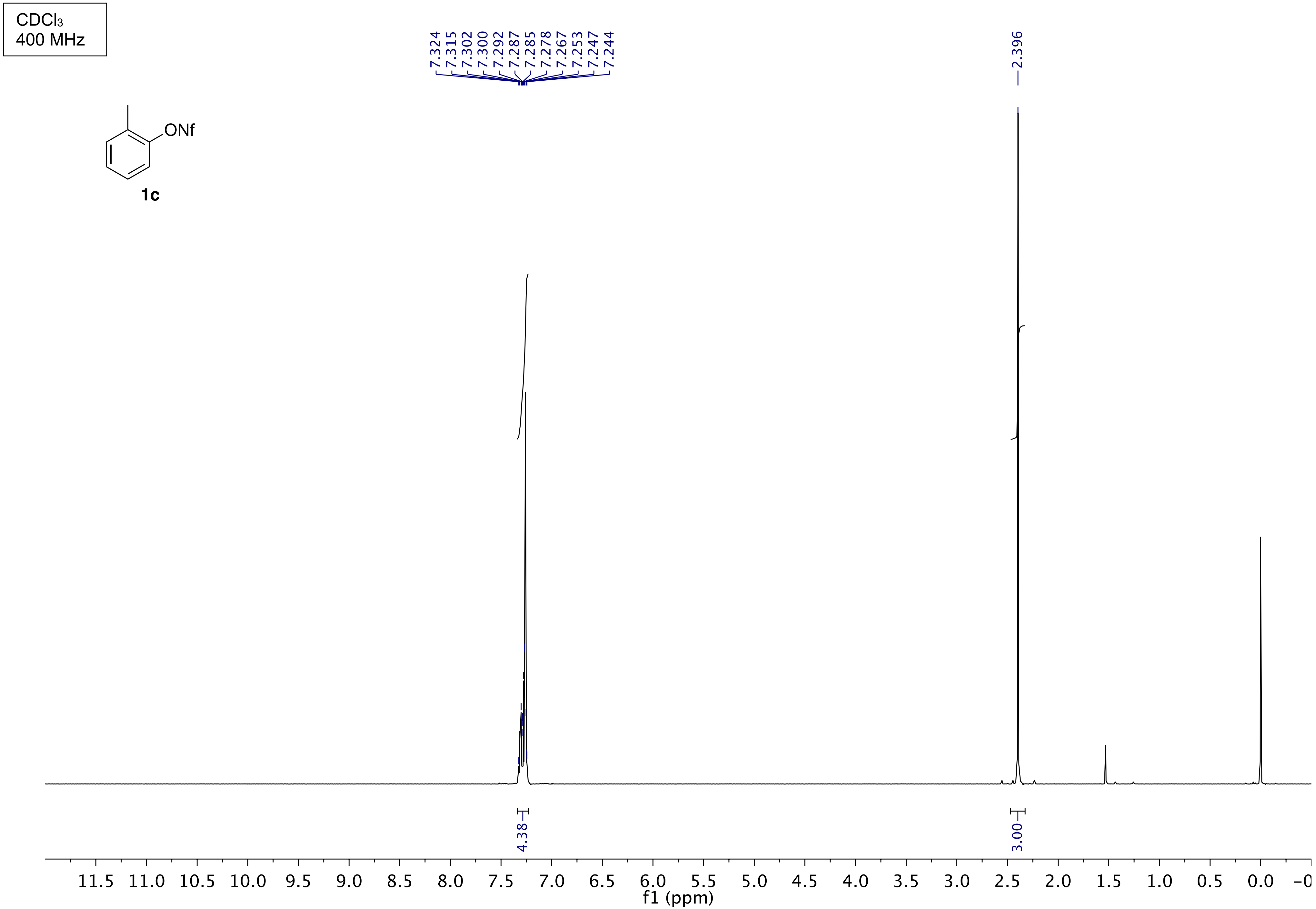

CDCl3
400 MHz
ONf
1c
7.324
7.315
7.302
7.300
7.292
7.287
7.285
7.278
7.267
7.253
7.247
7.244
2.396
4.38
3.00
11.5 11.0 10.5 10.0 9.5 9.0 8.5 8.0 7.5 7.0 6.5 6.0 5.5 5.0 4.5 4.0 3.5 3.0 2.5 2.0 1.5 1.0 0.5 0.0
f1 (ppm)

CDCl3
101 MHz

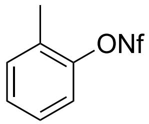

1c

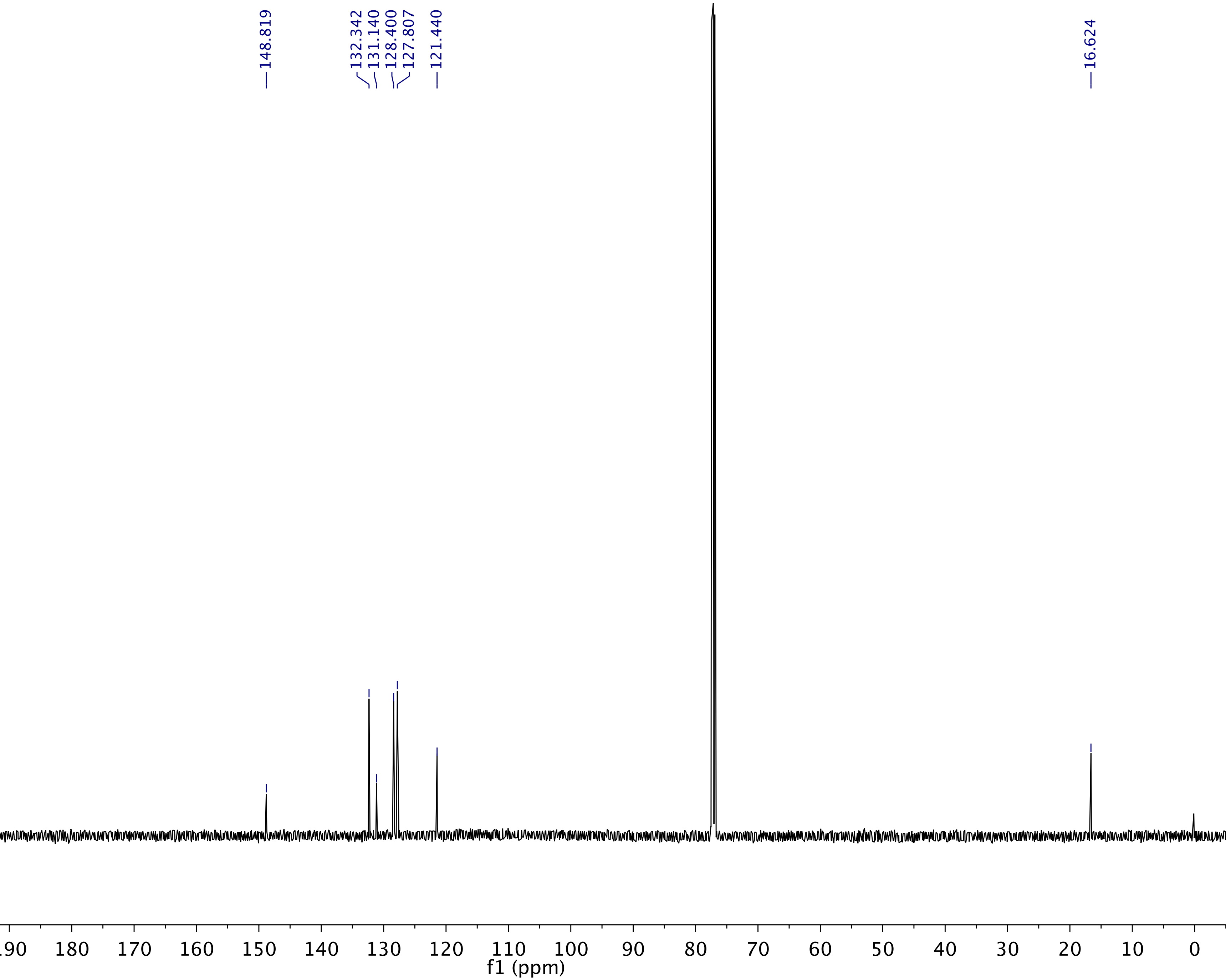

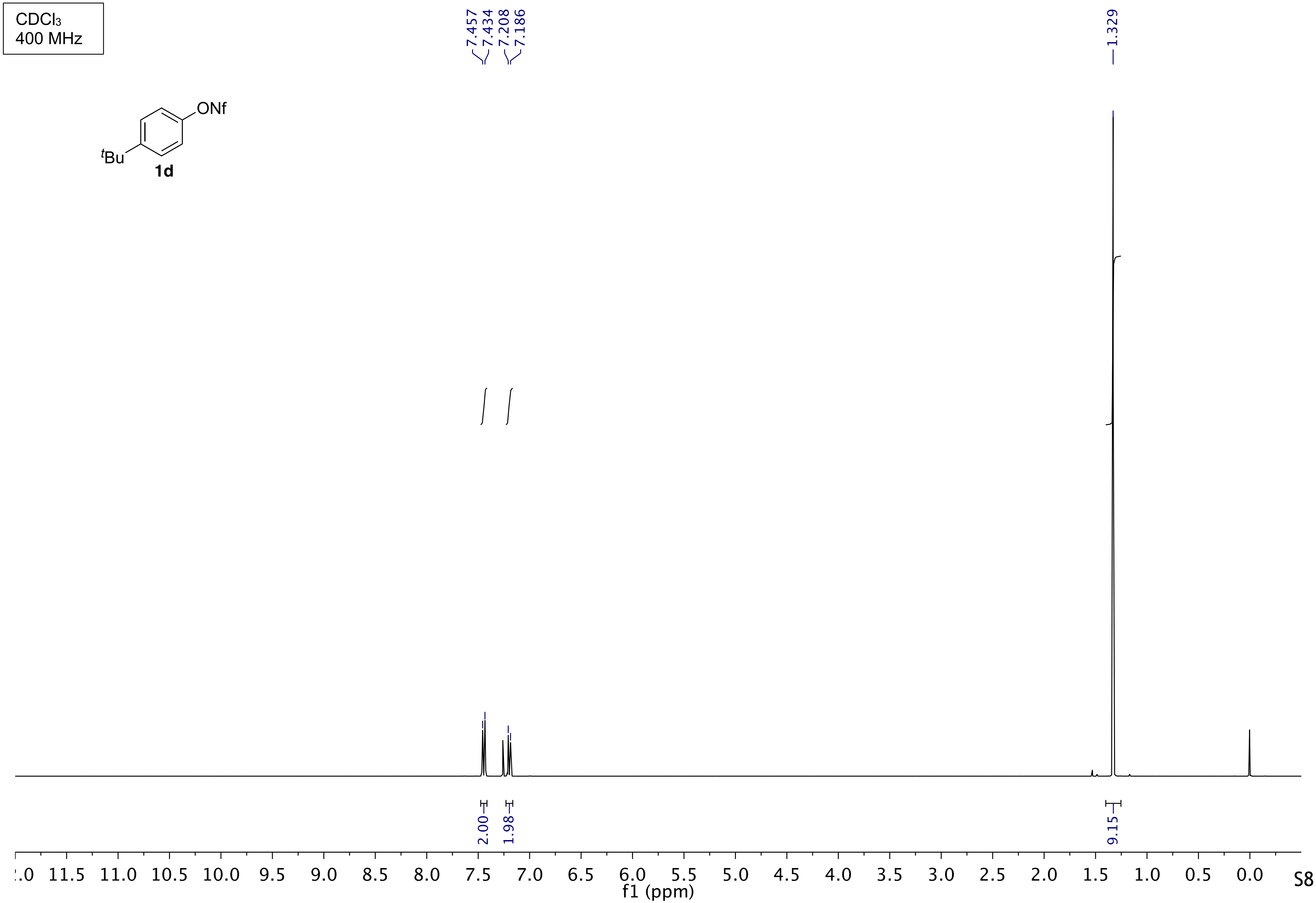
CDCl$_3$
400 MHz
ONf
^{t}Bu
1d
7.457
7.434
7.208
7.186
1.329
2.00
1.98
9.15
f1 (ppm)

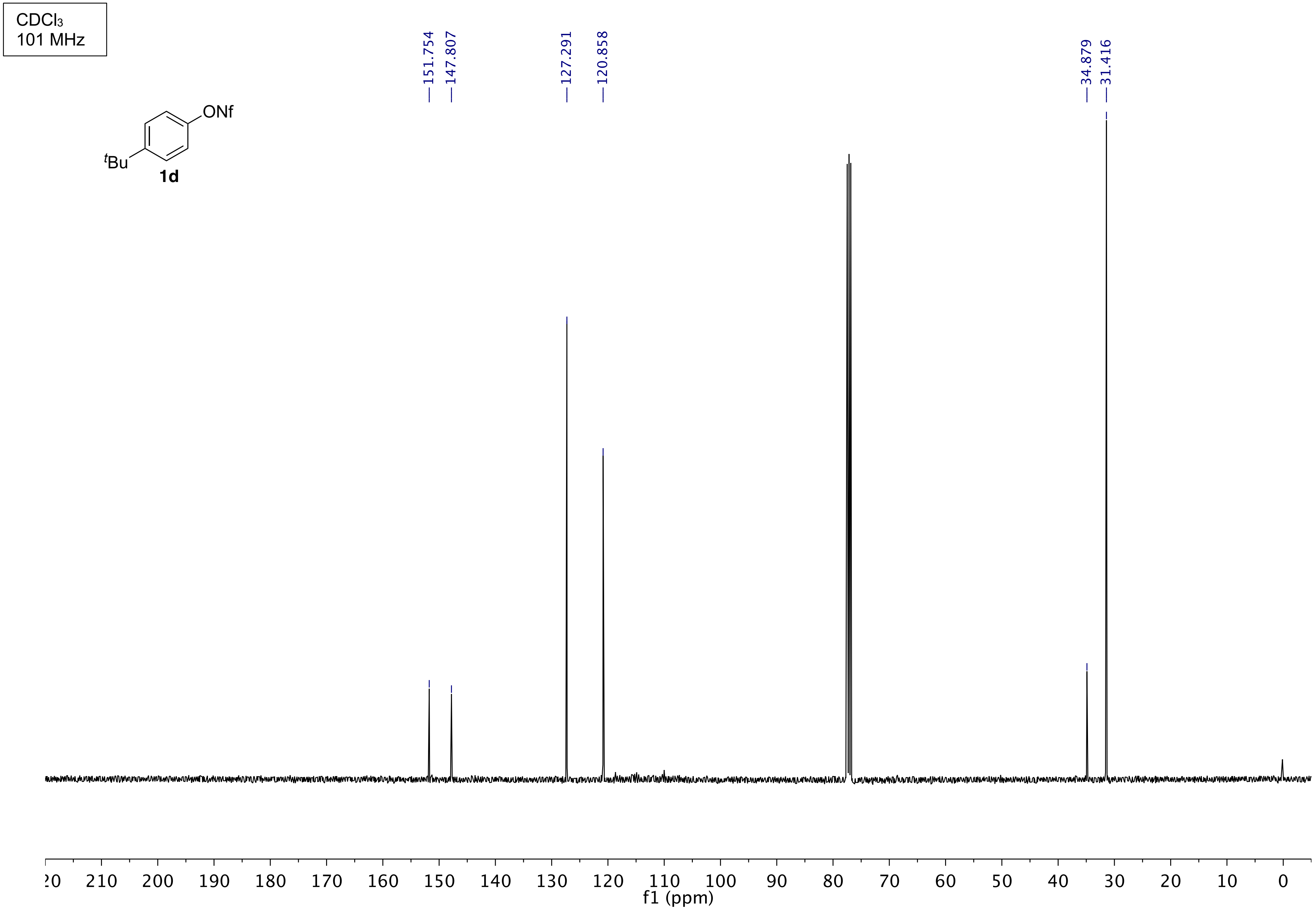
CDCl$_3$
101 MHz
ONf
^{t}Bu
1d
151.754
147.807
127.291
120.858
34.879
31.416
20 210 200 190 180 170 160 150 140 130 120 110 100 90 80 70 60 50 40 30 20 10 0
f1 (ppm)

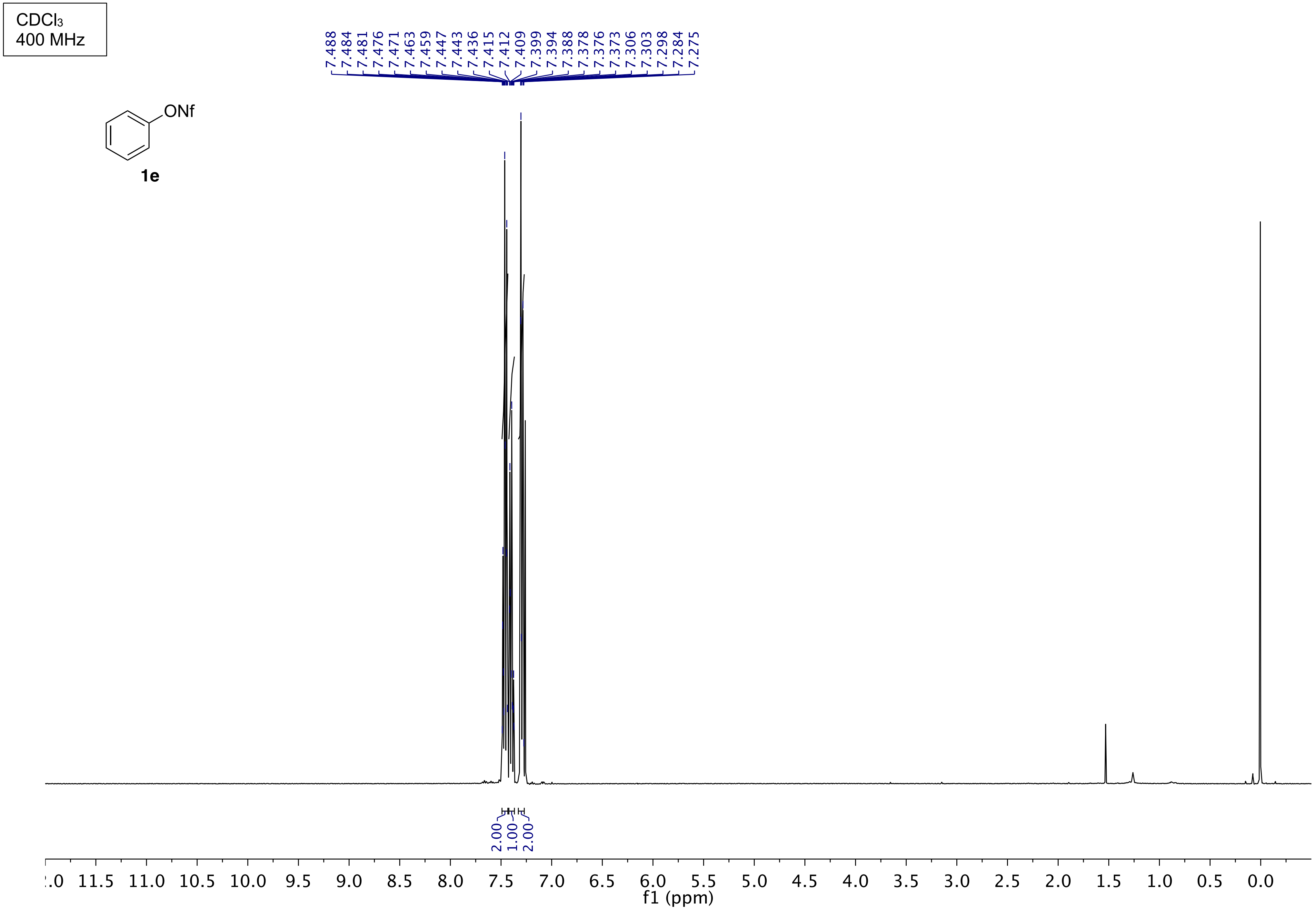
CDCl3
400 MHz
ONf
1e
7.488
7.484
7.481
7.476
7.471
7.463
7.459
7.447
7.443
7.436
7.415
7.412
7.409
7.399
7.394
7.388
7.378
7.376
7.373
7.306
7.303
7.298
7.284
7.275
2.00
1.00
2.00
f1 (ppm)

CDCl$_3$
101 MHz

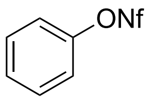

1e

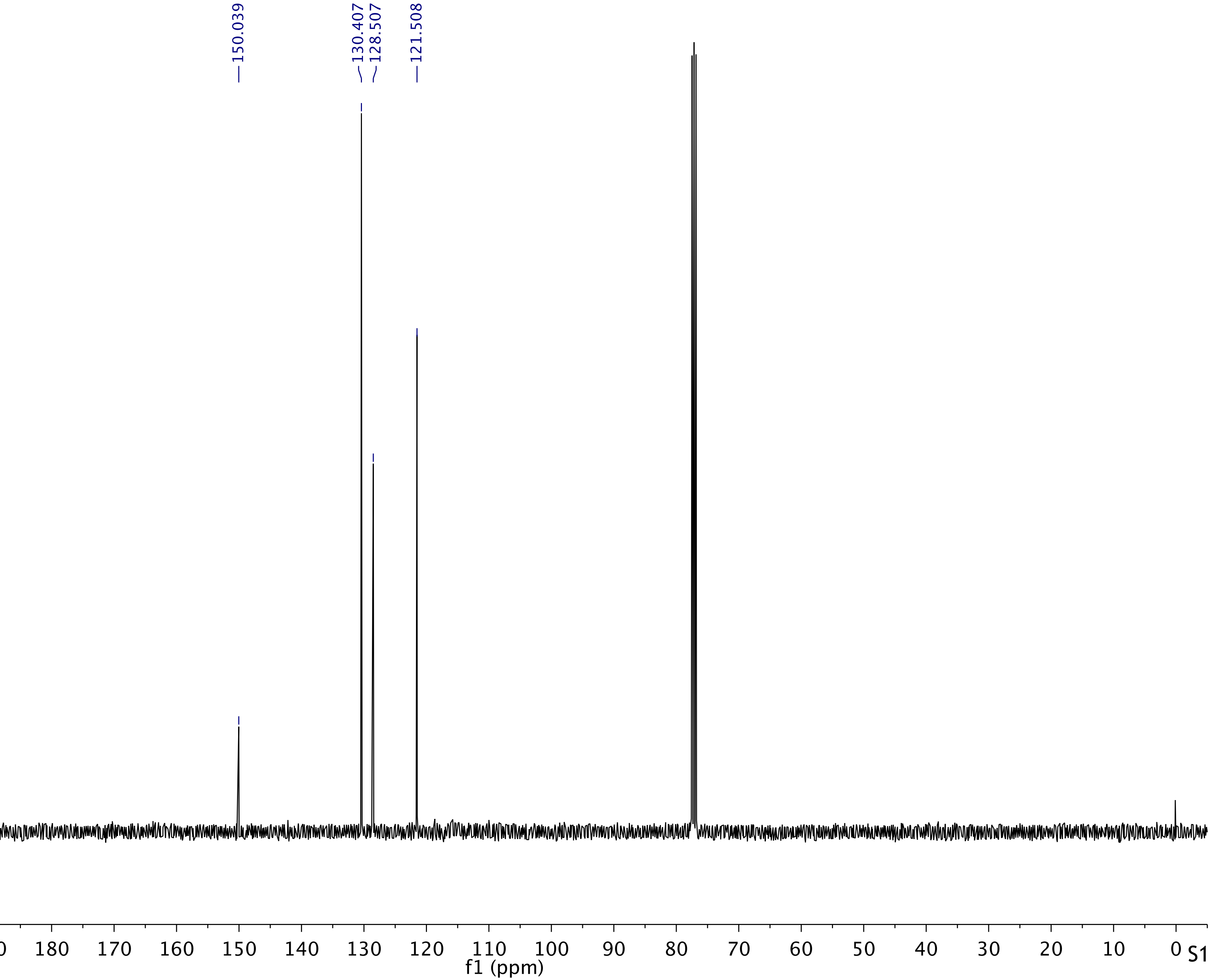

CDCl3
400 MHz

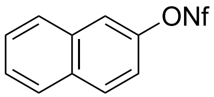

1f

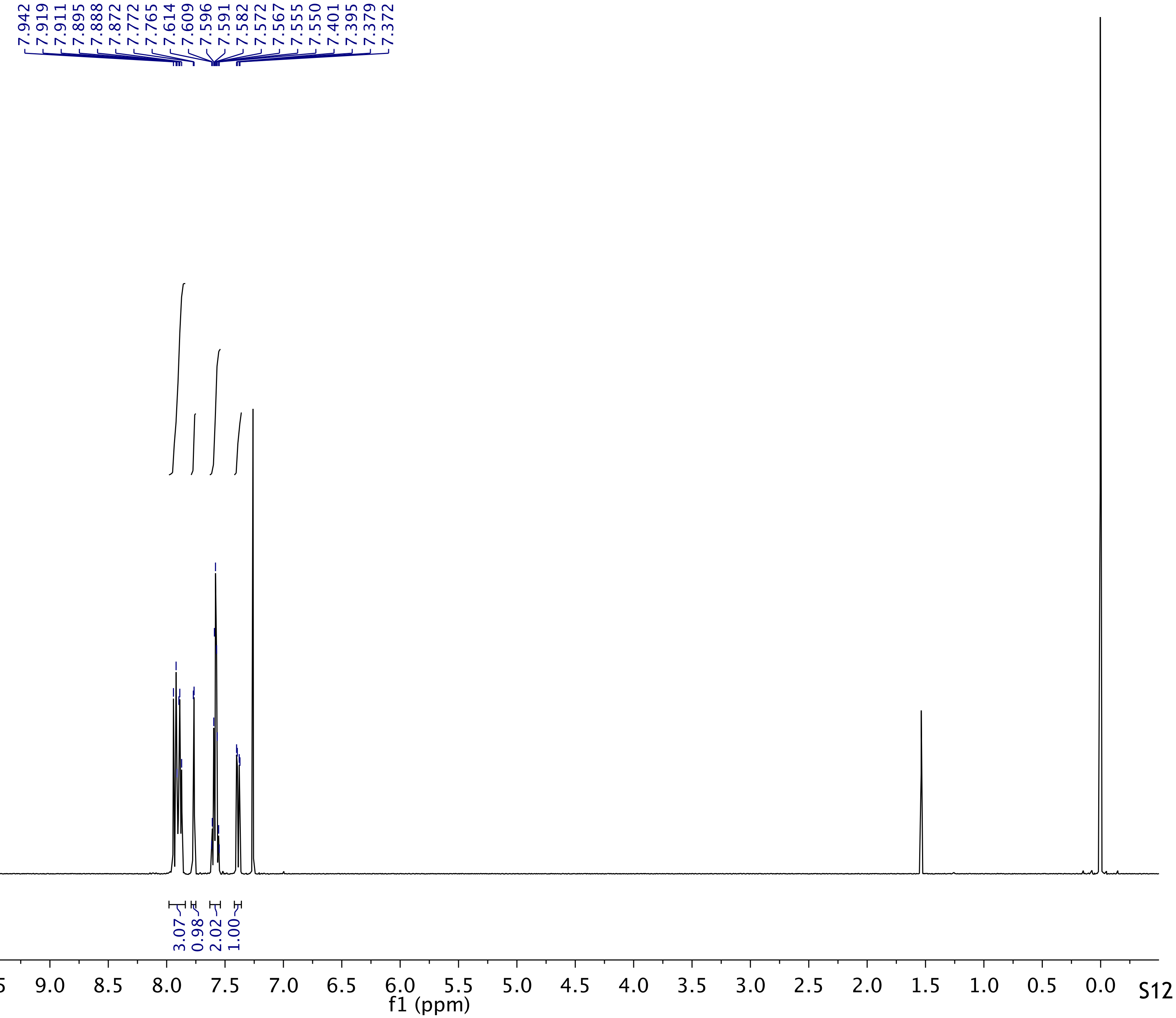

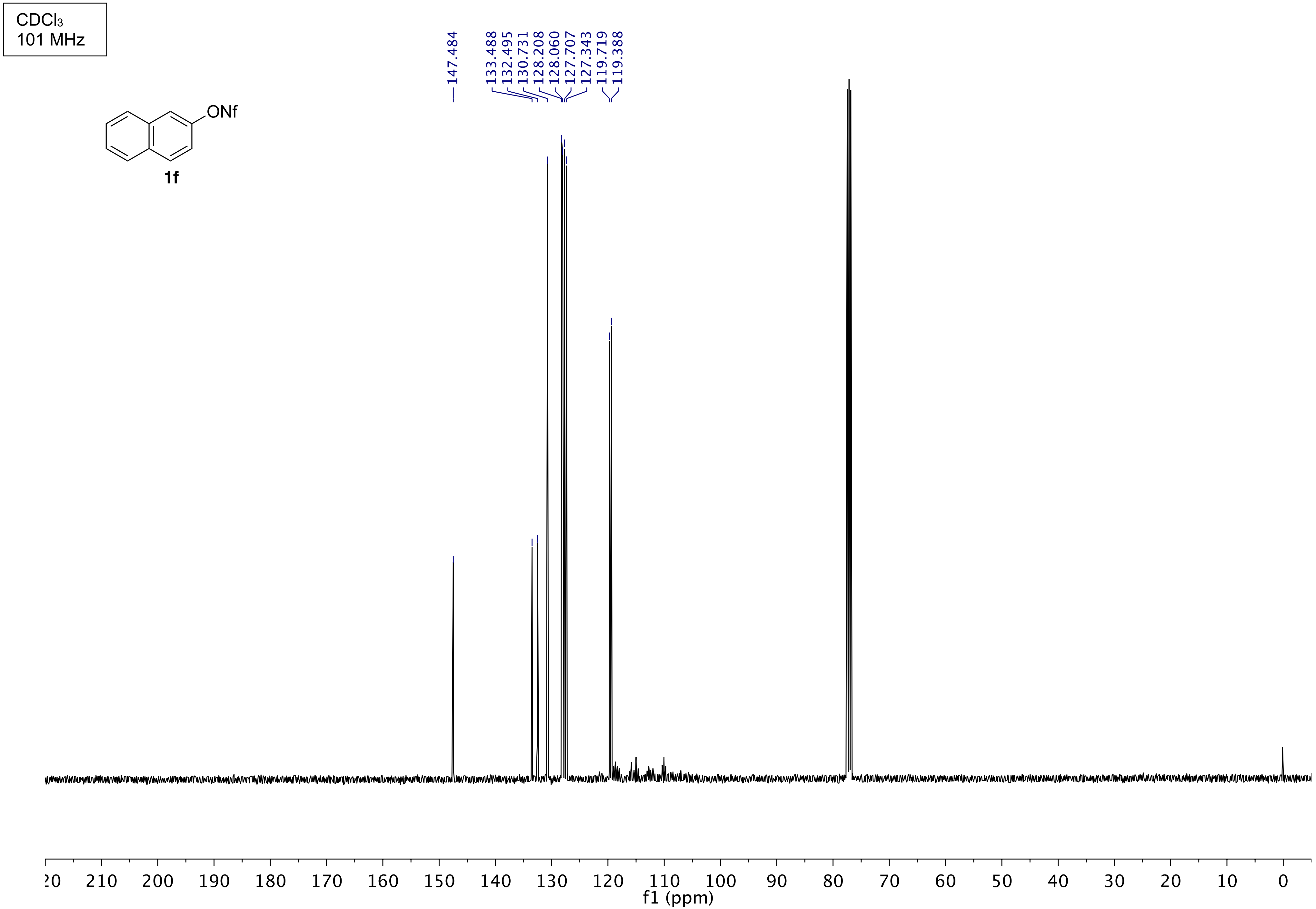
CDCl3
101 MHz
ONf
1f
147.484
133.488
132.495
130.731
128.208
128.060
127.707
127.343
119.719
119.388
f1 (ppm)

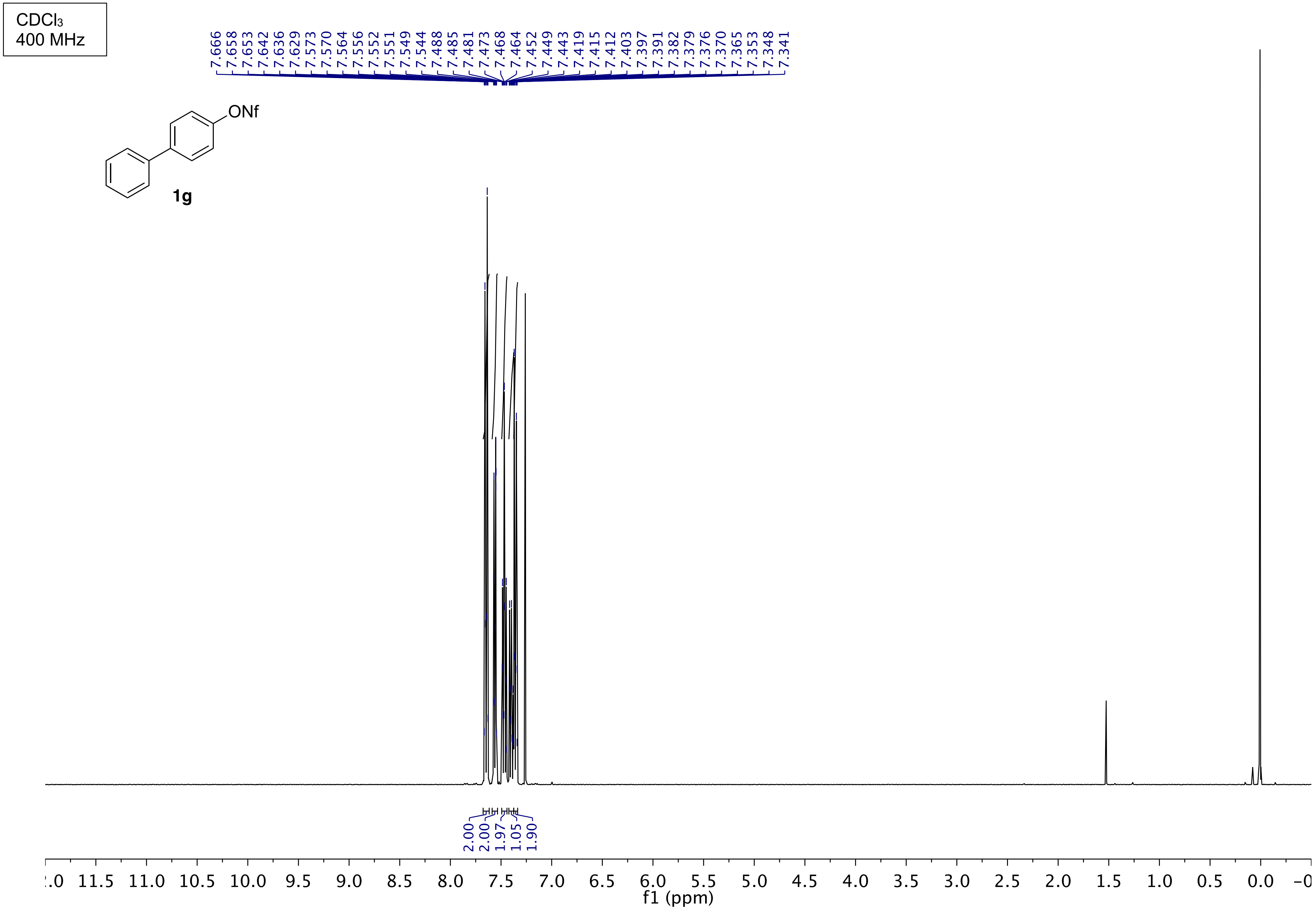
CDCl3
400 MHz
7.666 7.658 7.653 7.642 7.636 7.629 7.573 7.570 7.564 7.556 7.552 7.551 7.549 7.544 7.488 7.485 7.481 7.473 7.468 7.464 7.452 7.449 7.443 7.419 7.415 7.412 7.403 7.397 7.391 7.382 7.379 7.376 7.370 7.365 7.353 7.348 7.341
ONf
1g
2.00 2.00 1.97 1.05 1.90
11.5 11.0 10.5 10.0 9.5 9.0 8.5 8.0 7.5 7.0 6.5 6.0 5.5 5.0 4.5 4.0 3.5 3.0 2.5 2.0 1.5 1.0 0.5 0.0
f1 (ppm)

CDCl3
101 MHz

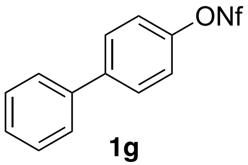

1g

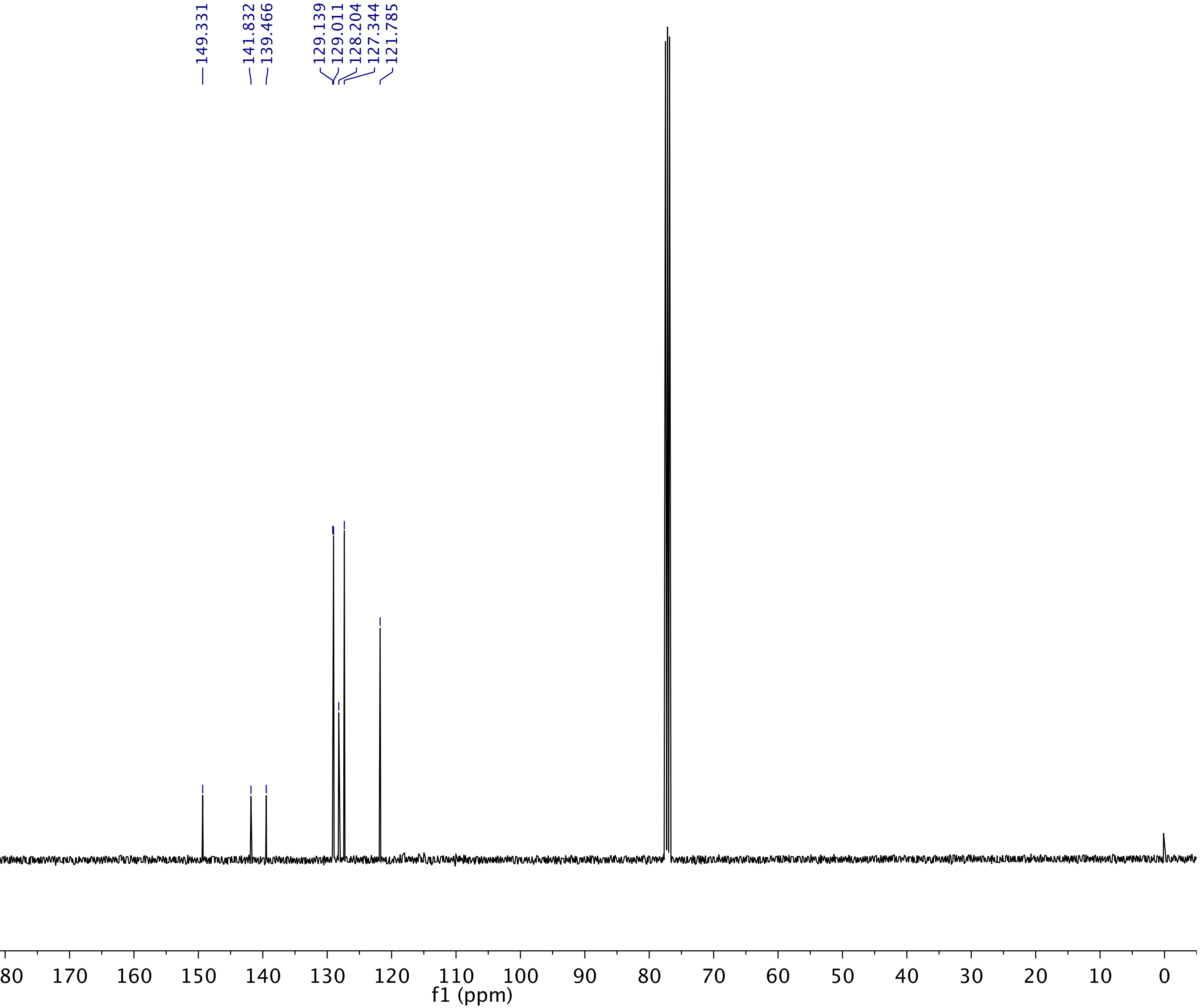

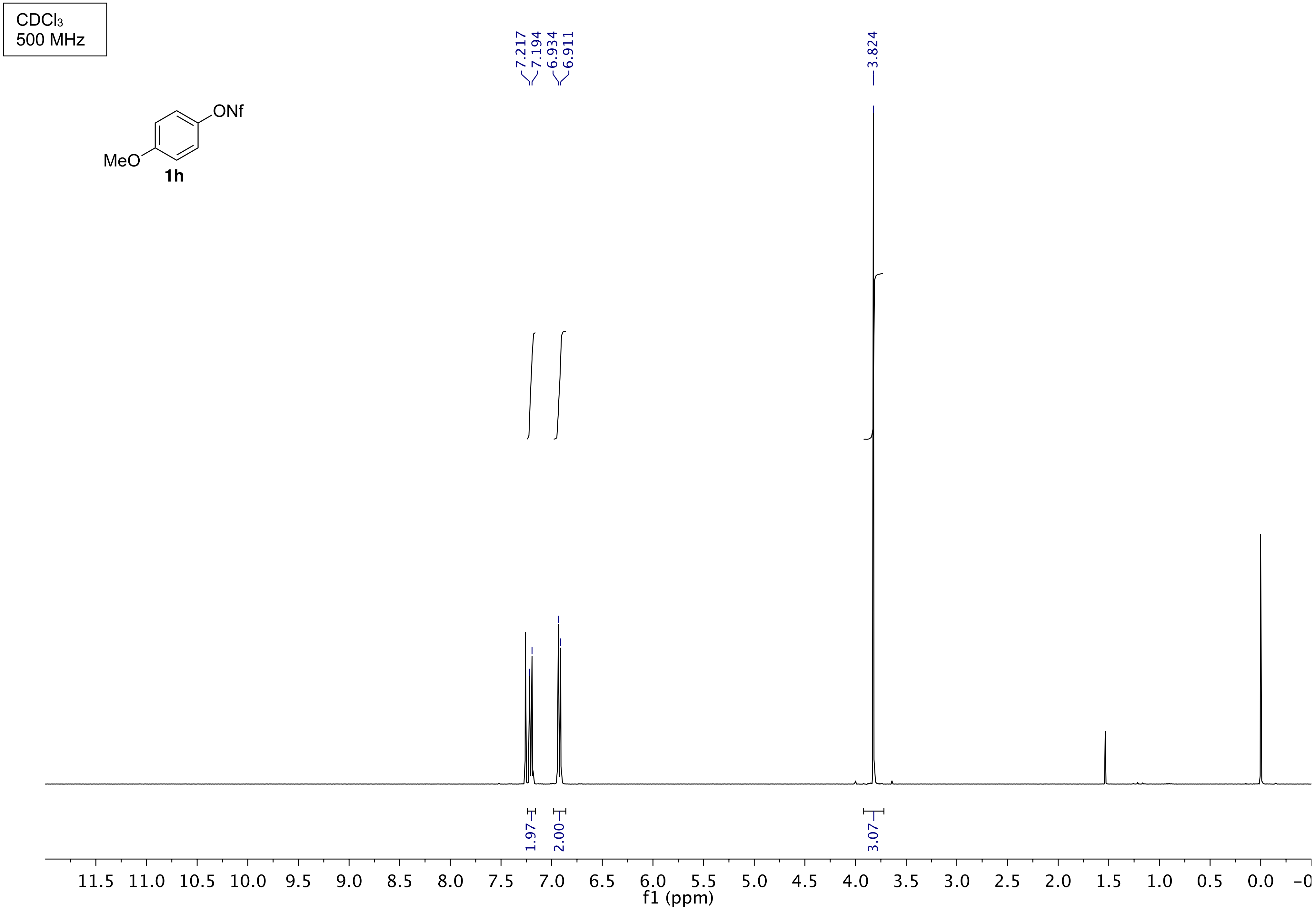
CDCl3
500 MHz
ONf
MeO
1h
7.217
7.194
6.934
6.911
3.824
1.97
2.00
3.07
11.5 11.0 10.5 10.0 9.5 9.0 8.5 8.0 7.5 7.0 6.5 6.0 5.5 5.0 4.5 4.0 3.5 3.0 2.5 2.0 1.5 1.0 0.5 0.0
f1 (ppm)

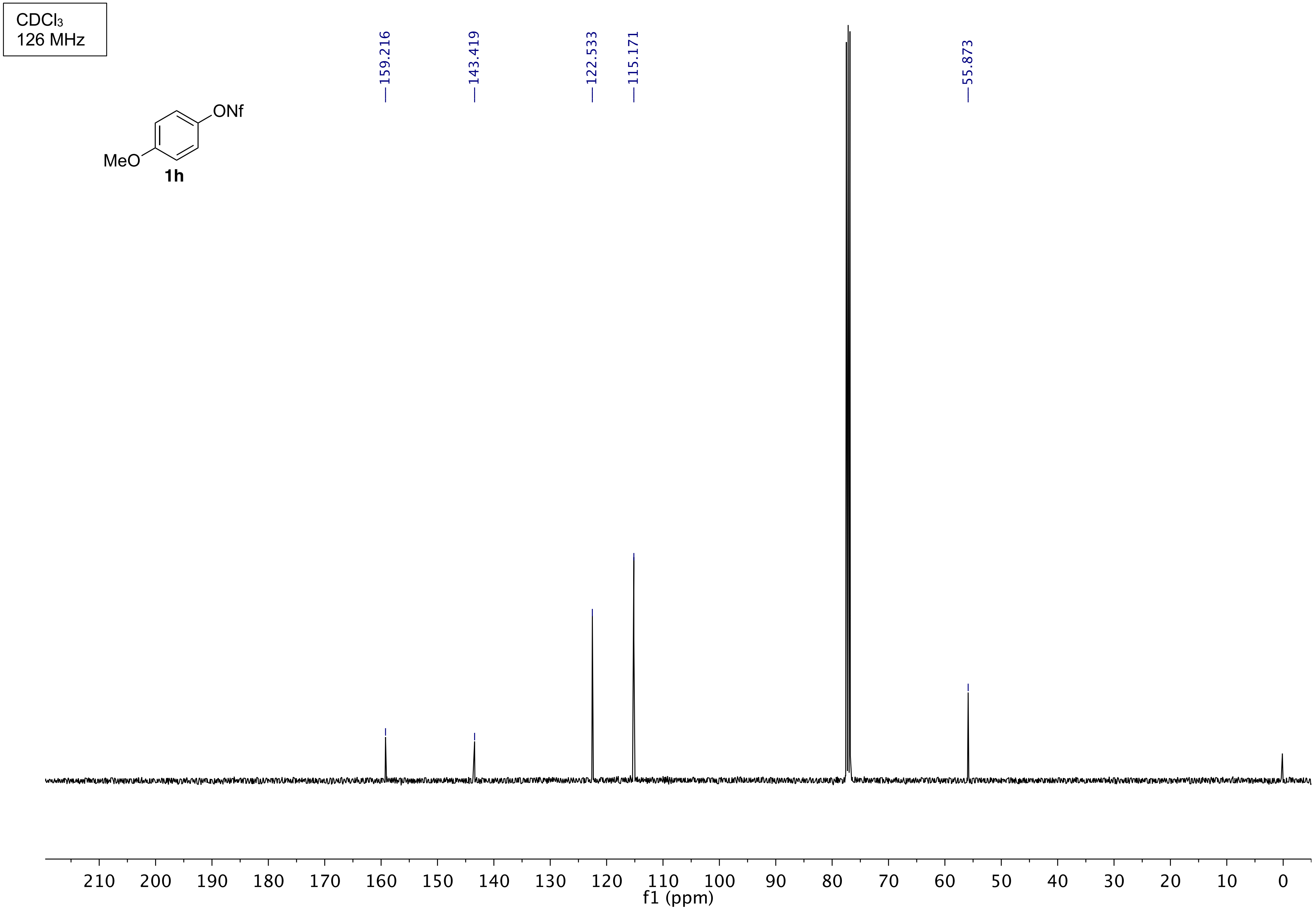
CDCl3
126 MHz
159.216
143.419
122.533
115.171
55.873
ONf
MeO
1h
210 200 190 180 170 160 150 140 130 120 110 100 90 80 70 60 50 40 30 20 10 0
f1 (ppm)

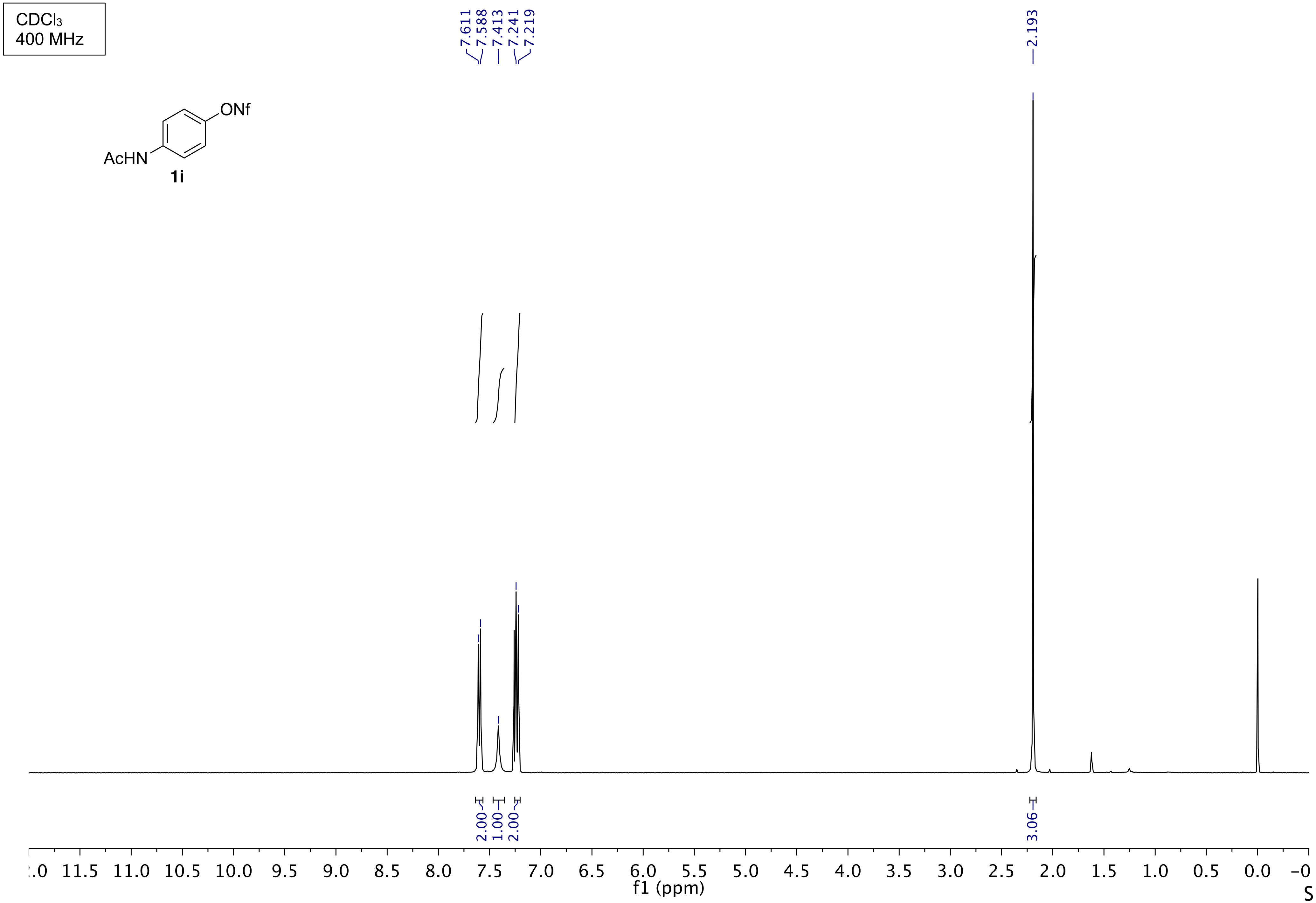
CDCl3
400 MHz
ONf
AcHN
1i
7.611
7.588
7.413
7.241
7.219
2.193
2.00
1.00
2.00
3.06
f1 (ppm)

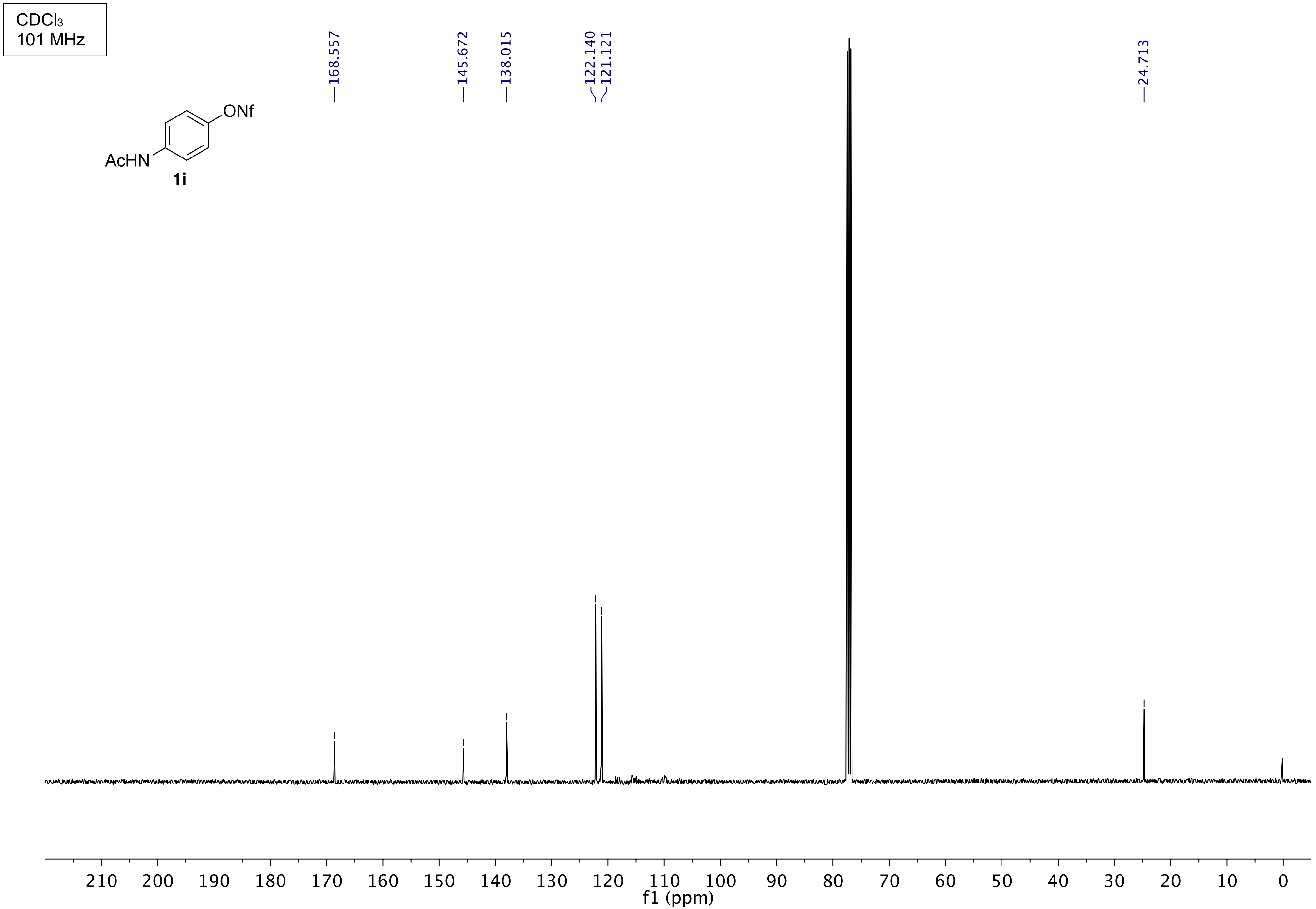
CDCl3
101 MHz
ONf
AcHN
1i
168.557
145.672
138.015
122.140
121.121
24.713
210 200 190 180 170 160 150 140 130 120 110 100 90 80 70 60 50 40 30 20 10 0
f1 (ppm)

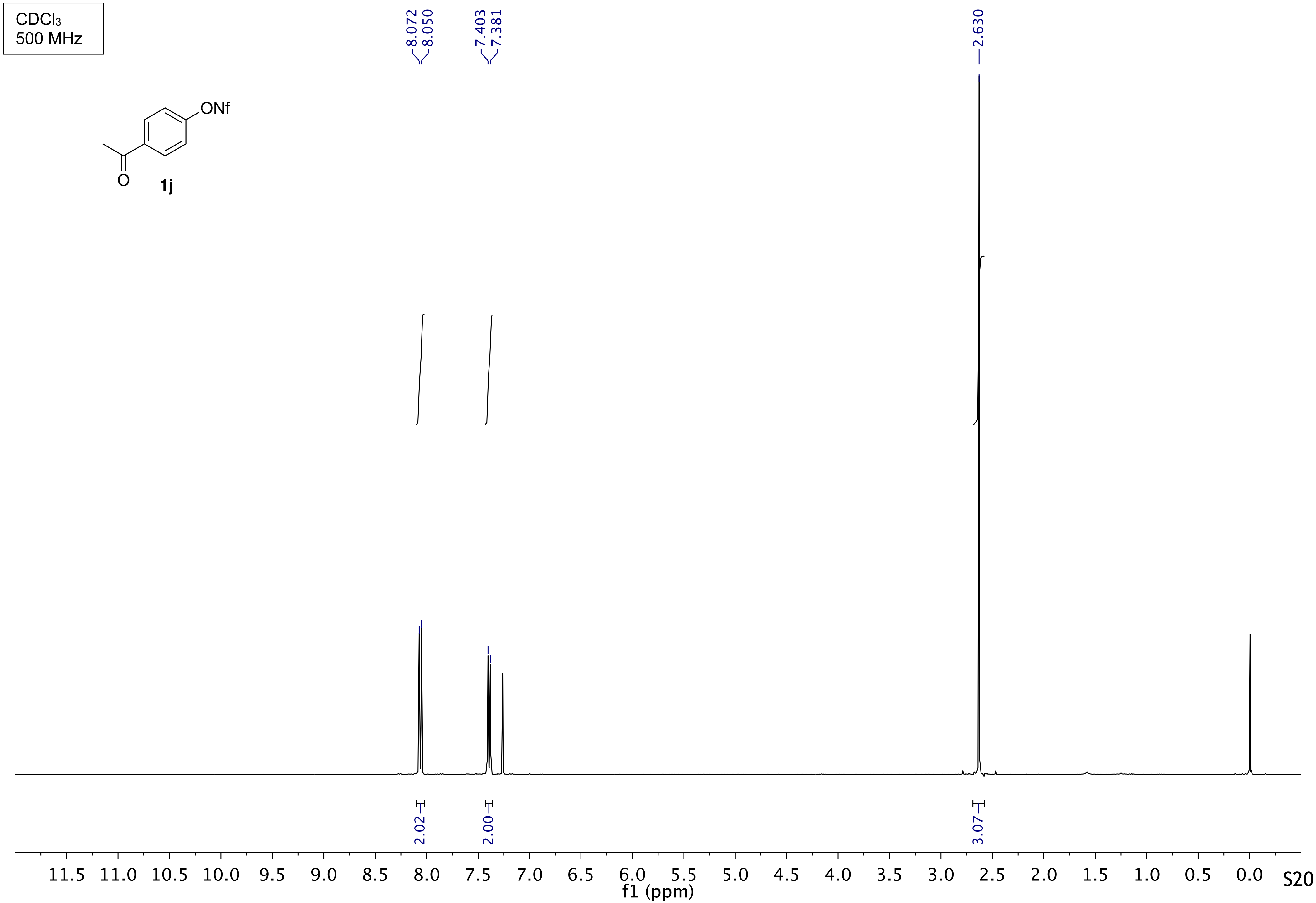
CDCl3
500 MHz
ONf
O
1j
8.072
8.050
7.403
7.381
2.630
2.02
2.00
3.07
11.5 11.0 10.5 10.0 9.5 9.0 8.5 8.0 7.5 7.0 6.5 6.0 5.5 5.0 4.5 4.0 3.5 3.0 2.5 2.0 1.5 1.0 0.5 0.0
f1 (ppm)

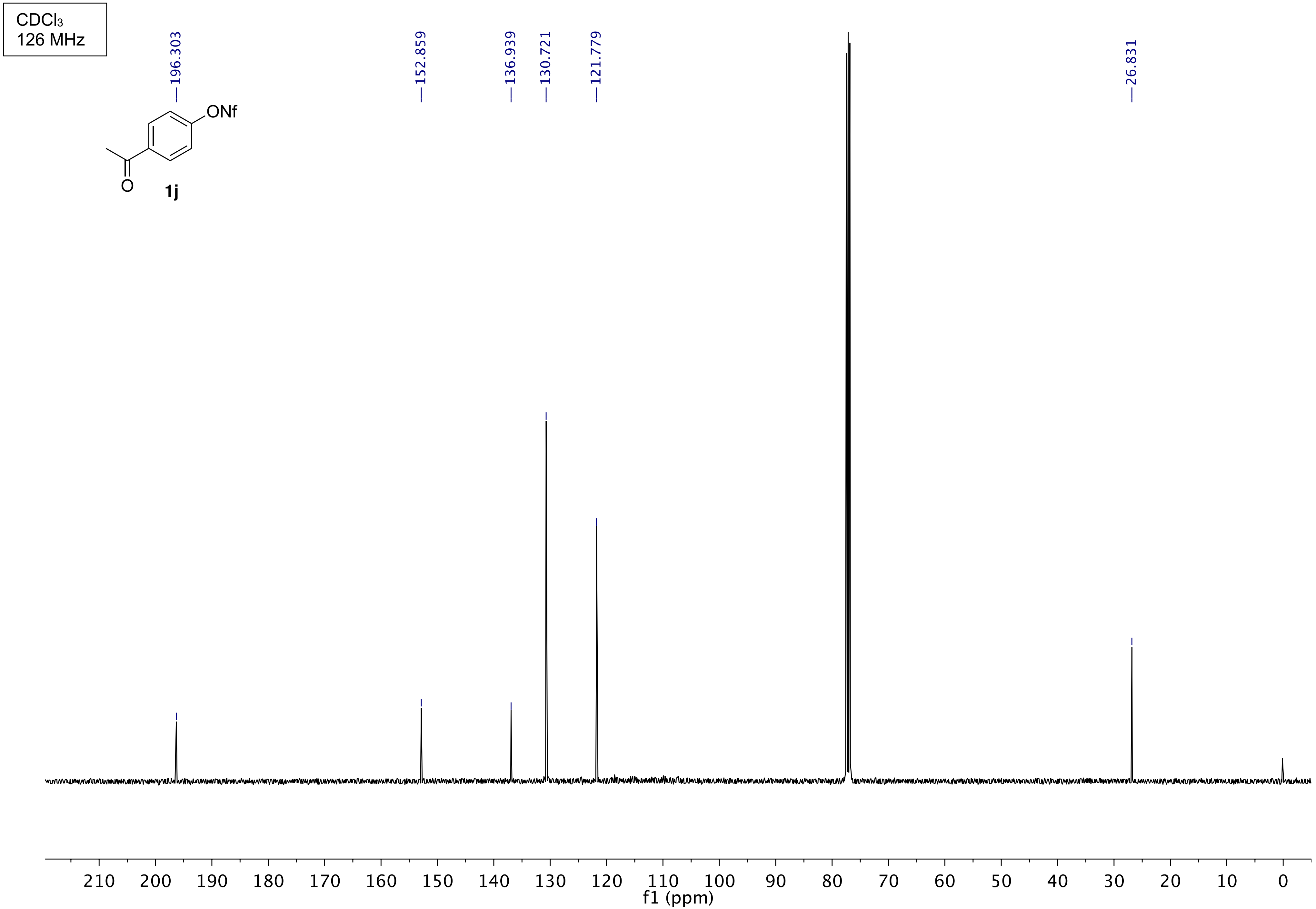
CDCl$_3$
126 MHz
196.303
152.859
136.939
130.721
121.779
26.831
ONf
O
1j
210 200 190 180 170 160 150 140 130 120 110 100 90 80 70 60 50 40 30 20 10 0
f1 (ppm)

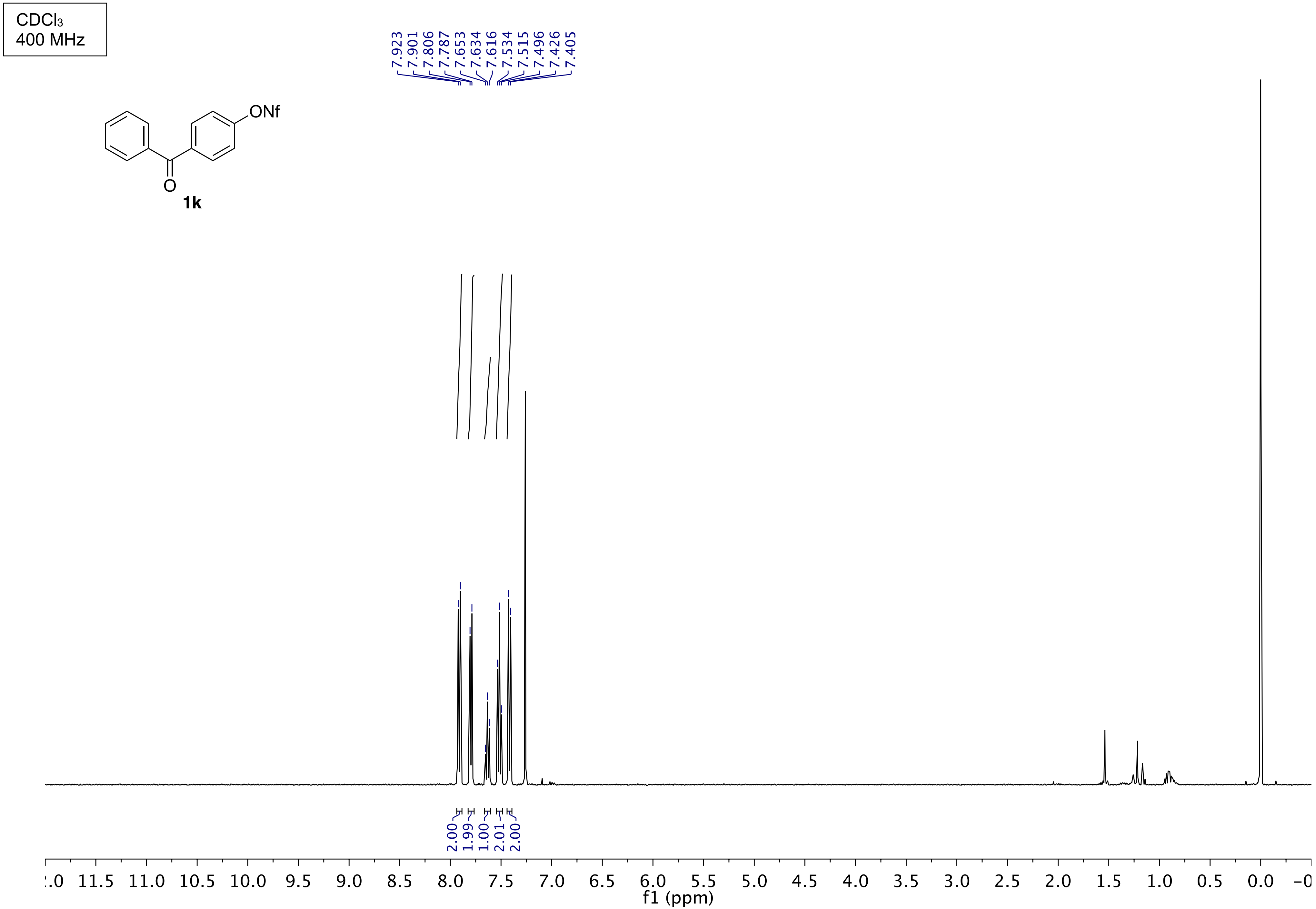
CDCl3
400 MHz
ONf
O
1k
7.923
7.901
7.806
7.787
7.653
7.634
7.616
7.534
7.515
7.496
7.426
7.405
2.00
1.99
1.00
2.01
2.00
11.5 11.0 10.5 10.0 9.5 9.0 8.5 8.0 7.5 7.0 6.5 6.0 5.5 5.0 4.5 4.0 3.5 3.0 2.5 2.0 1.5 1.0 0.5 0.0
f1 (ppm)

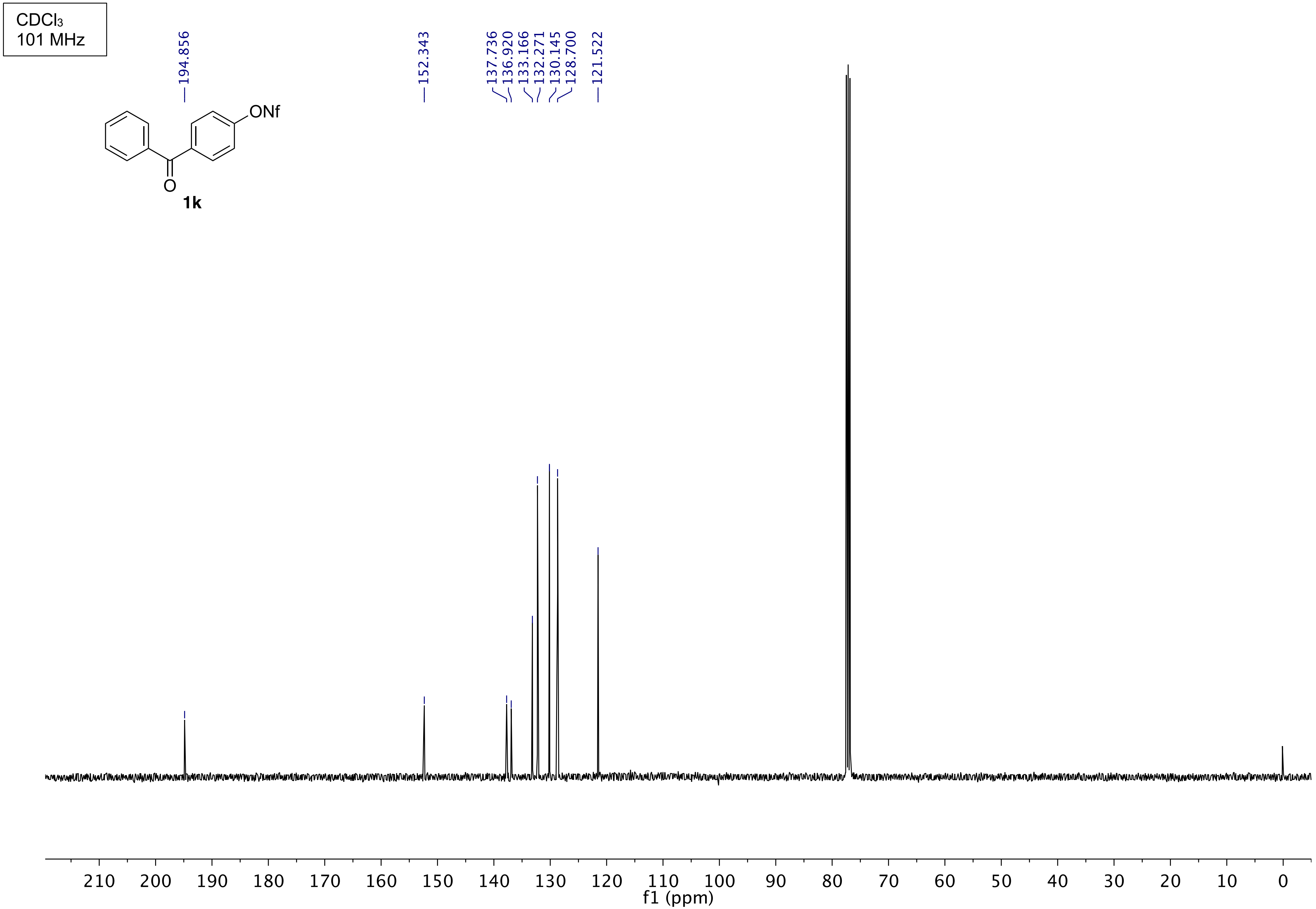

CDCl$_3$
101 MHz
194.856
152.343
137.736
136.920
133.166
132.271
130.145
128.700
121.522
ONf
O
1k
210 200 190 180 170 160 150 140 130 120 110 100 90 80 70 60 50 40 30 20 10 0
f1 (ppm)

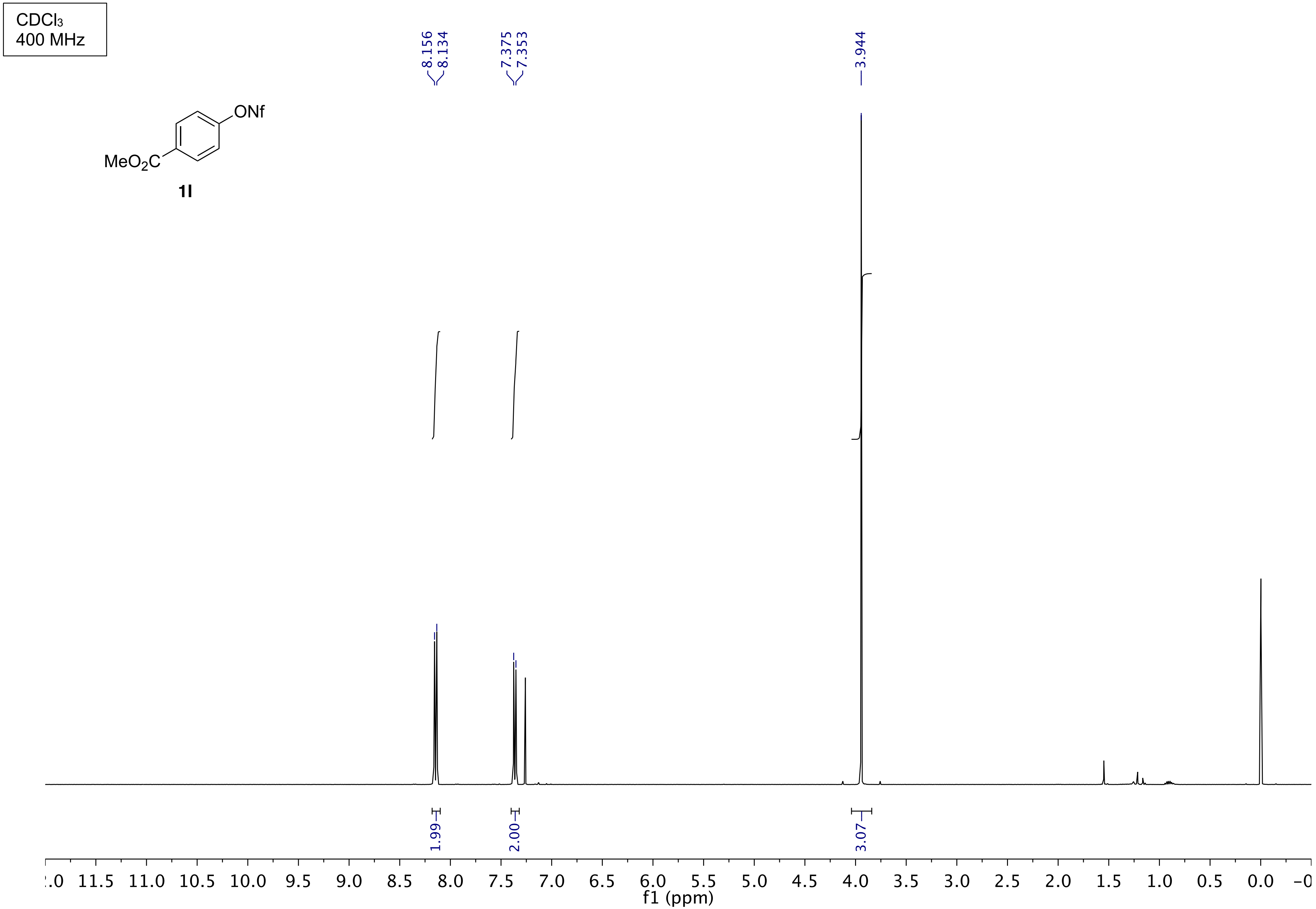

CDCl3
400 MHz
ONf
MeO2C
1l
8.156
8.134
7.375
7.353
3.944
1.99
2.00
3.07
12.0 11.5 11.0 10.5 10.0 9.5 9.0 8.5 8.0 7.5 7.0 6.5 6.0 5.5 5.0 4.5 4.0 3.5 3.0 2.5 2.0 1.5 1.0 0.5 0.0 -0
f1 (ppm)

CDCl3
101 MHz

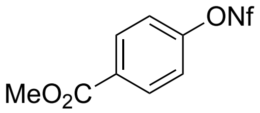

1l

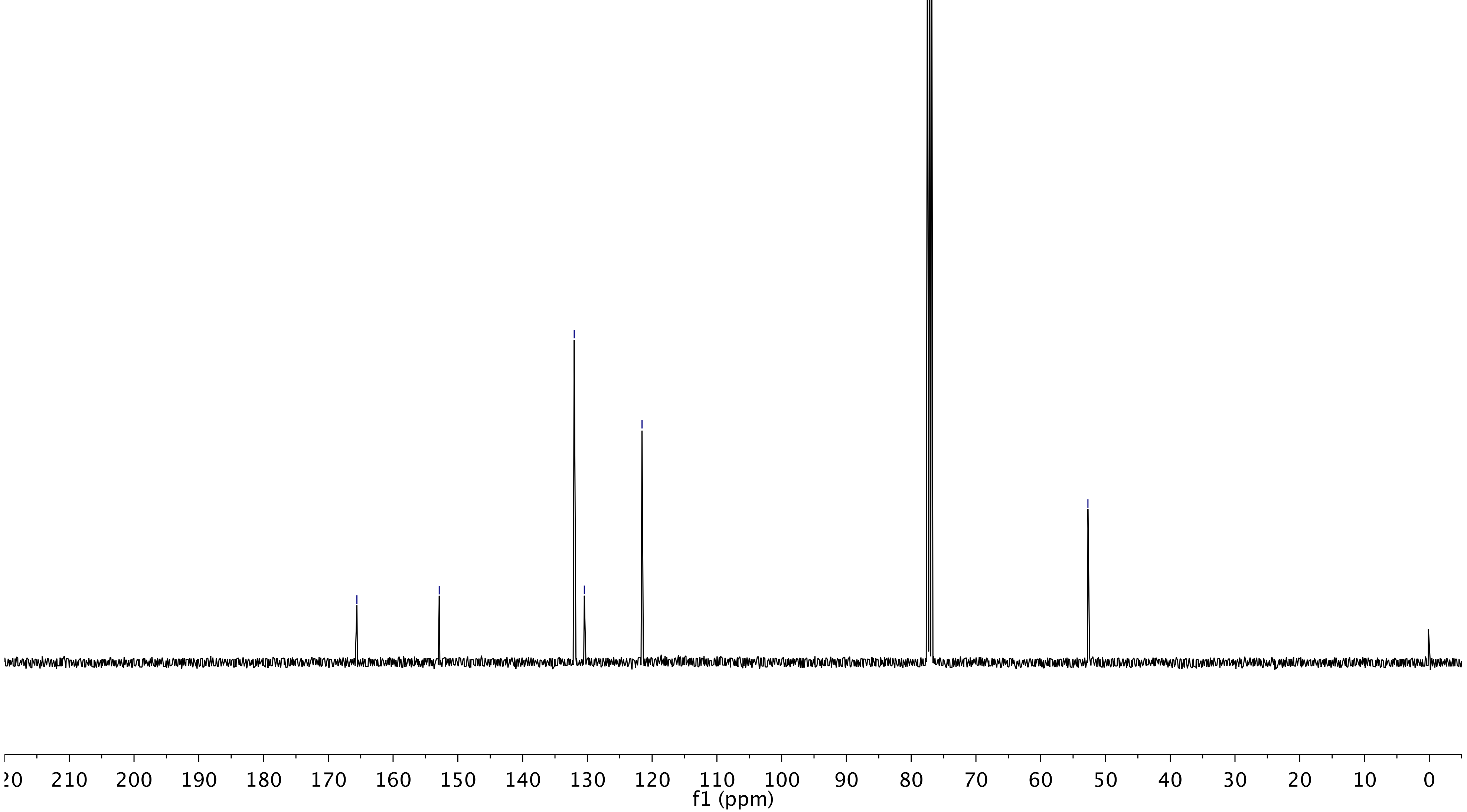

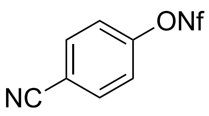

1m

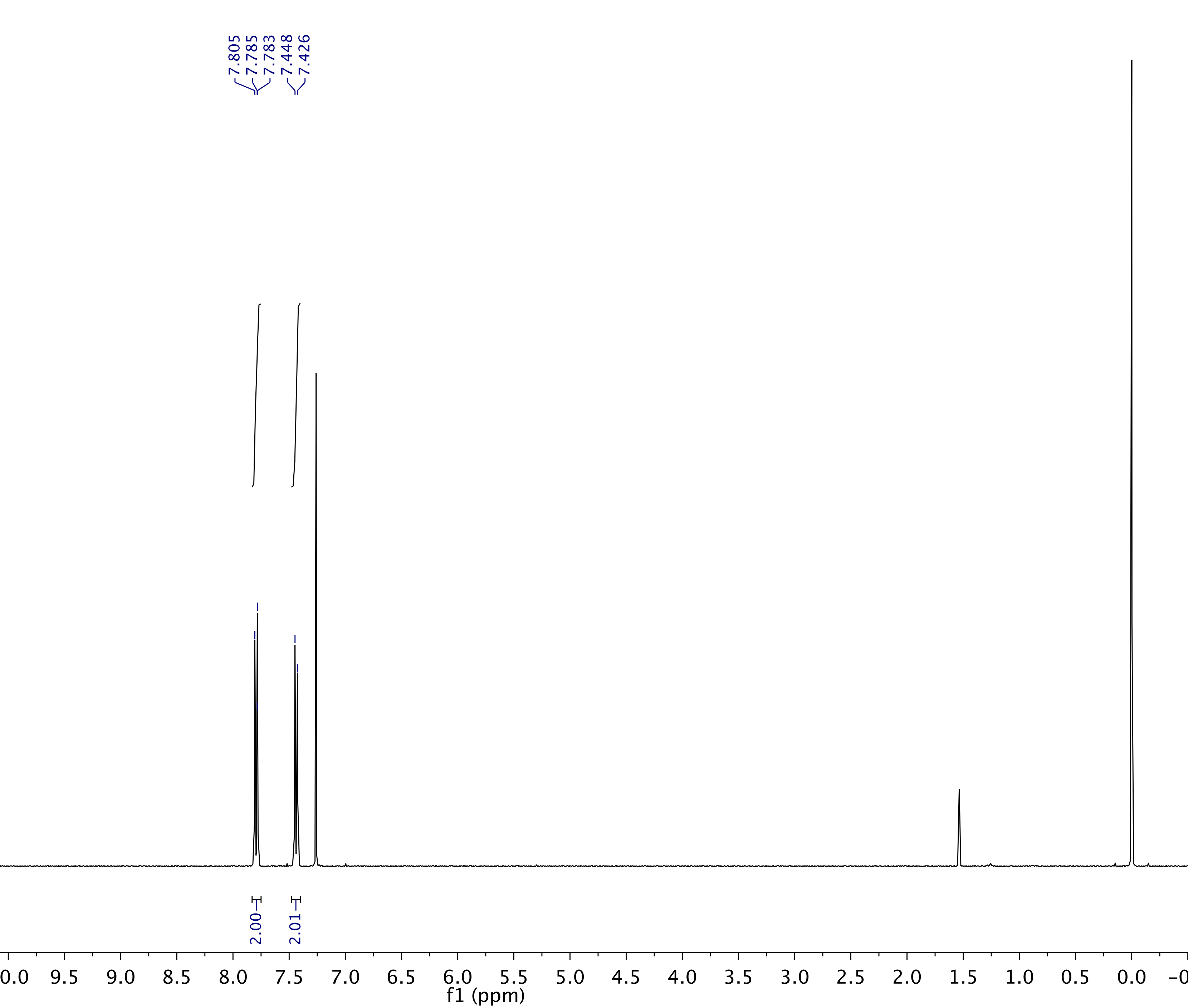

CDCl3
500 MHz
7.805
7.785
7.783
7.448
7.426
2.00
2.01
12.0 11.5 11.0 10.5 10.0 9.5 9.0 8.5 8.0 7.5 7.0 6.5 6.0 5.5 5.0 4.5 4.0 3.5 3.0 2.5 2.0 1.5 1.0 0.5 0.0 -0
f1 (ppm)

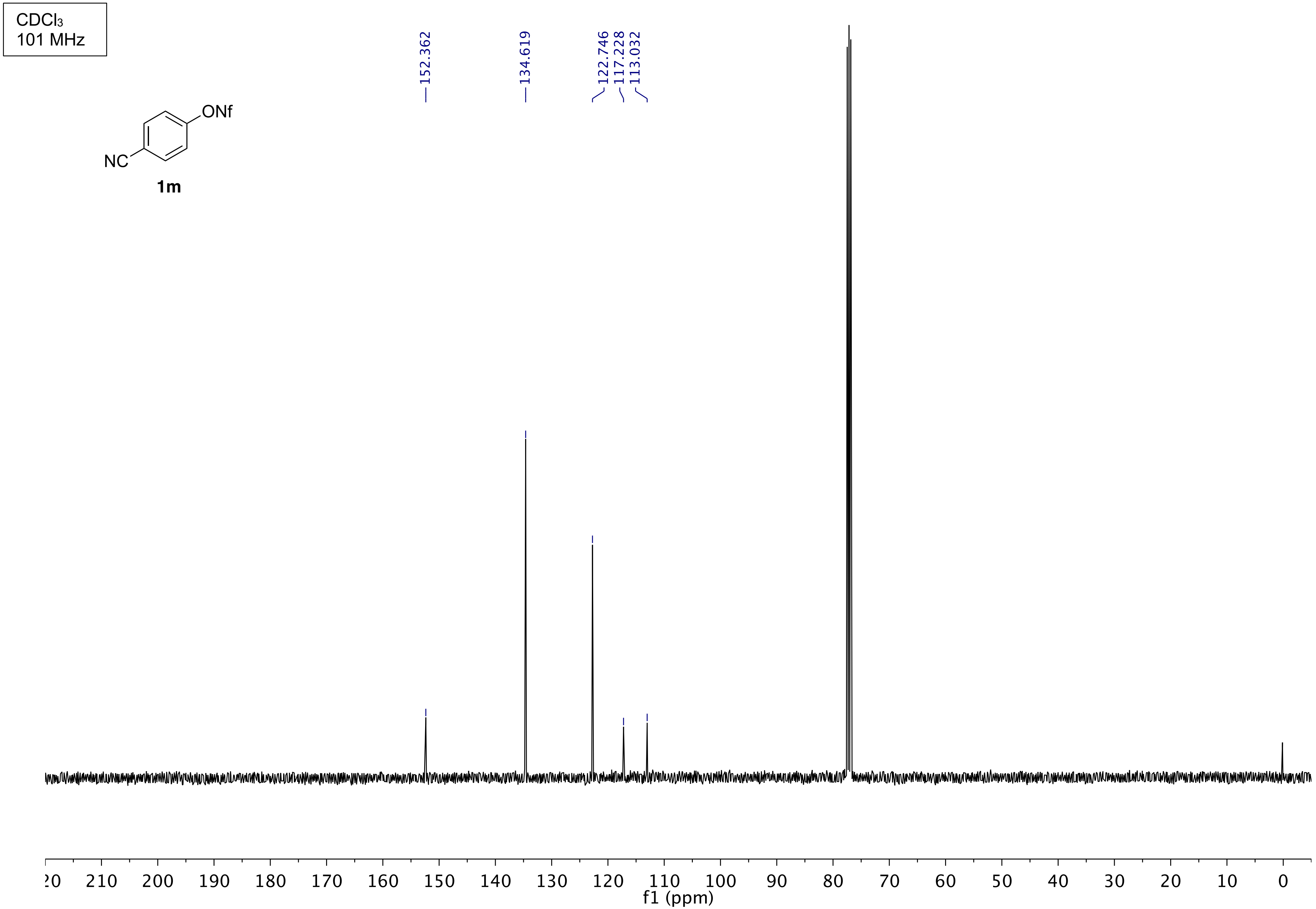

CDCl3
101 MHz
ONf
NC
1m
152.362
134.619
122.746
117.228
113.032
f1 (ppm)

CDCl3
400 MHz

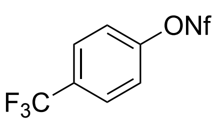

1n

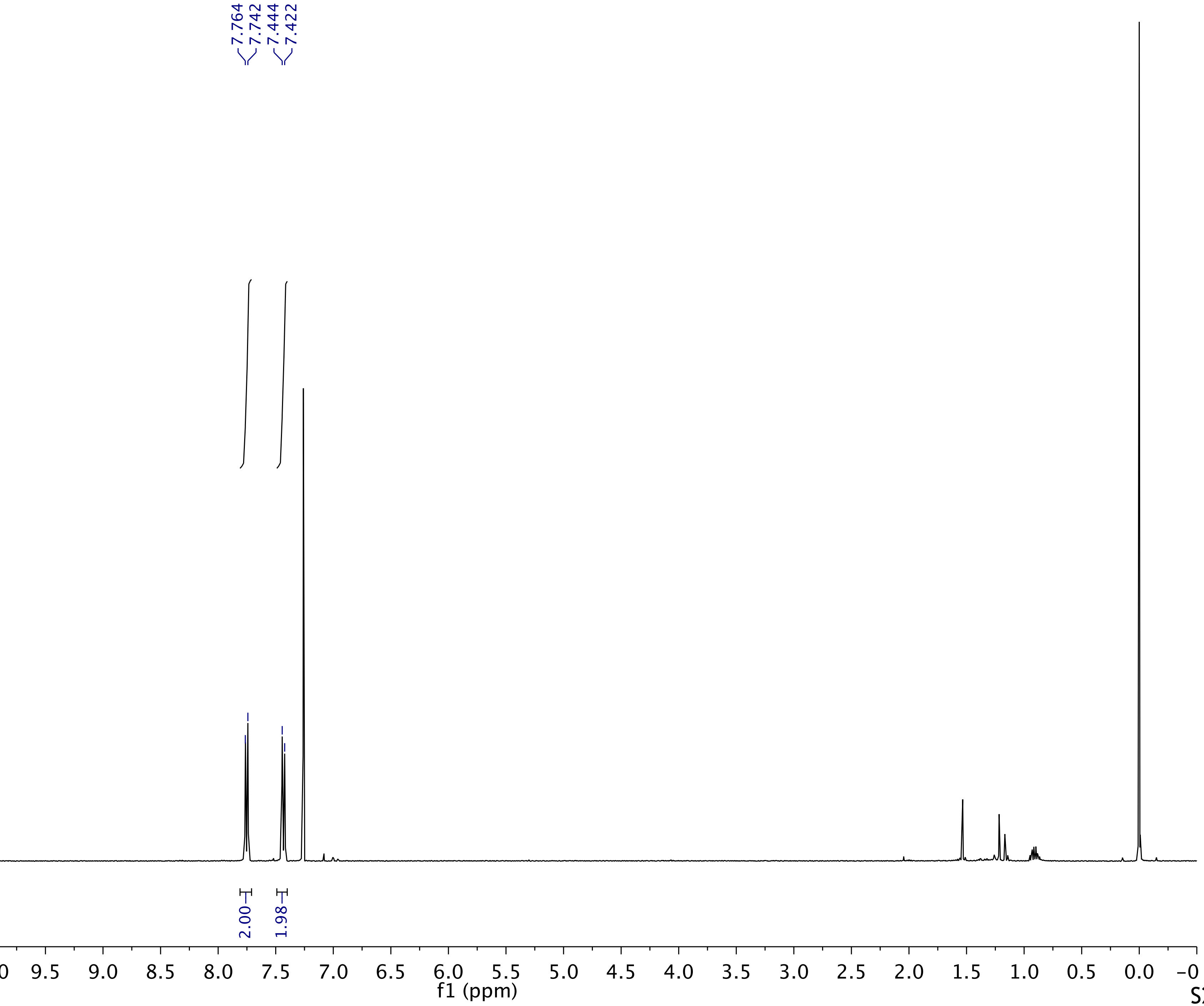

CDCl3
101 MHz

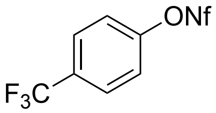

1n

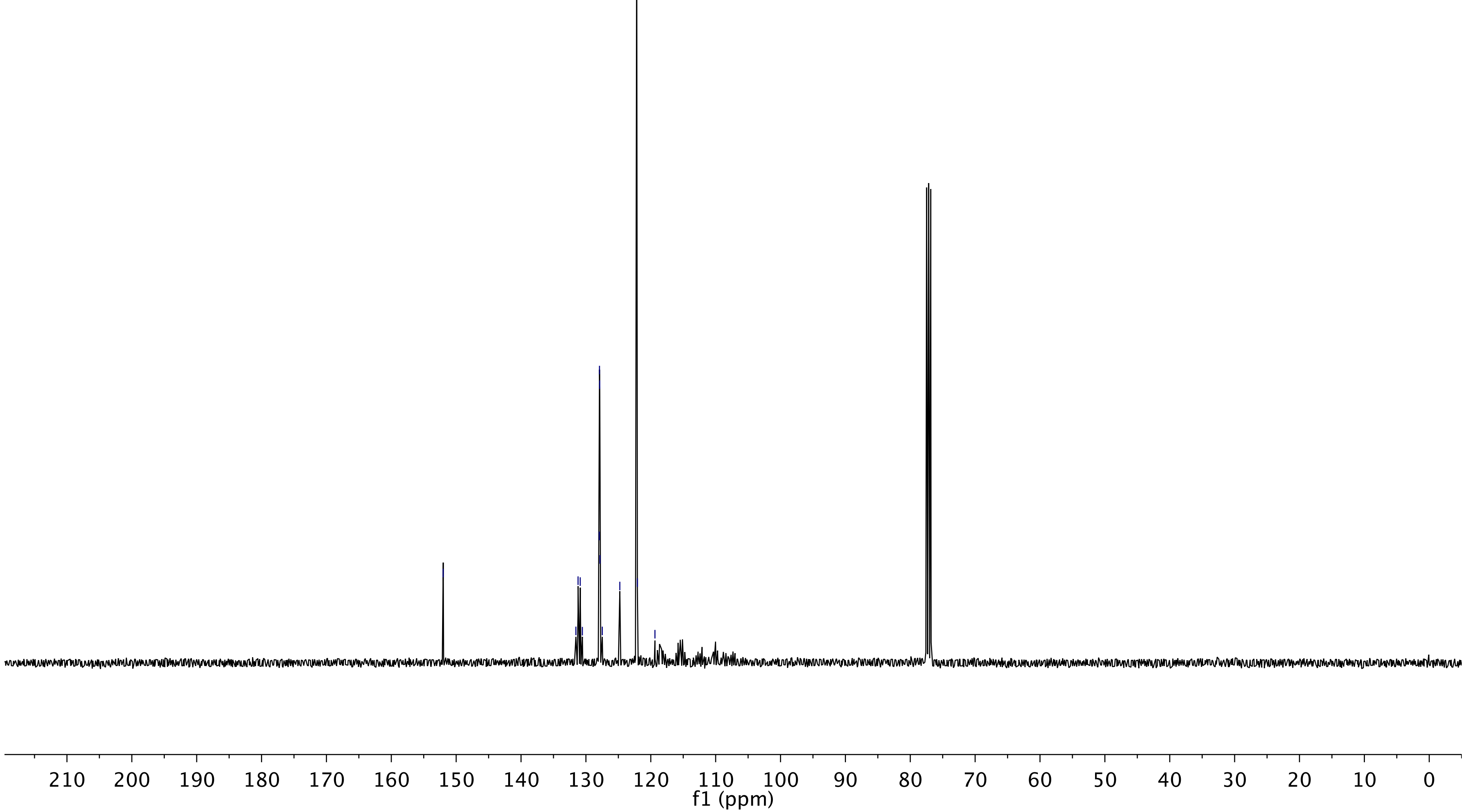

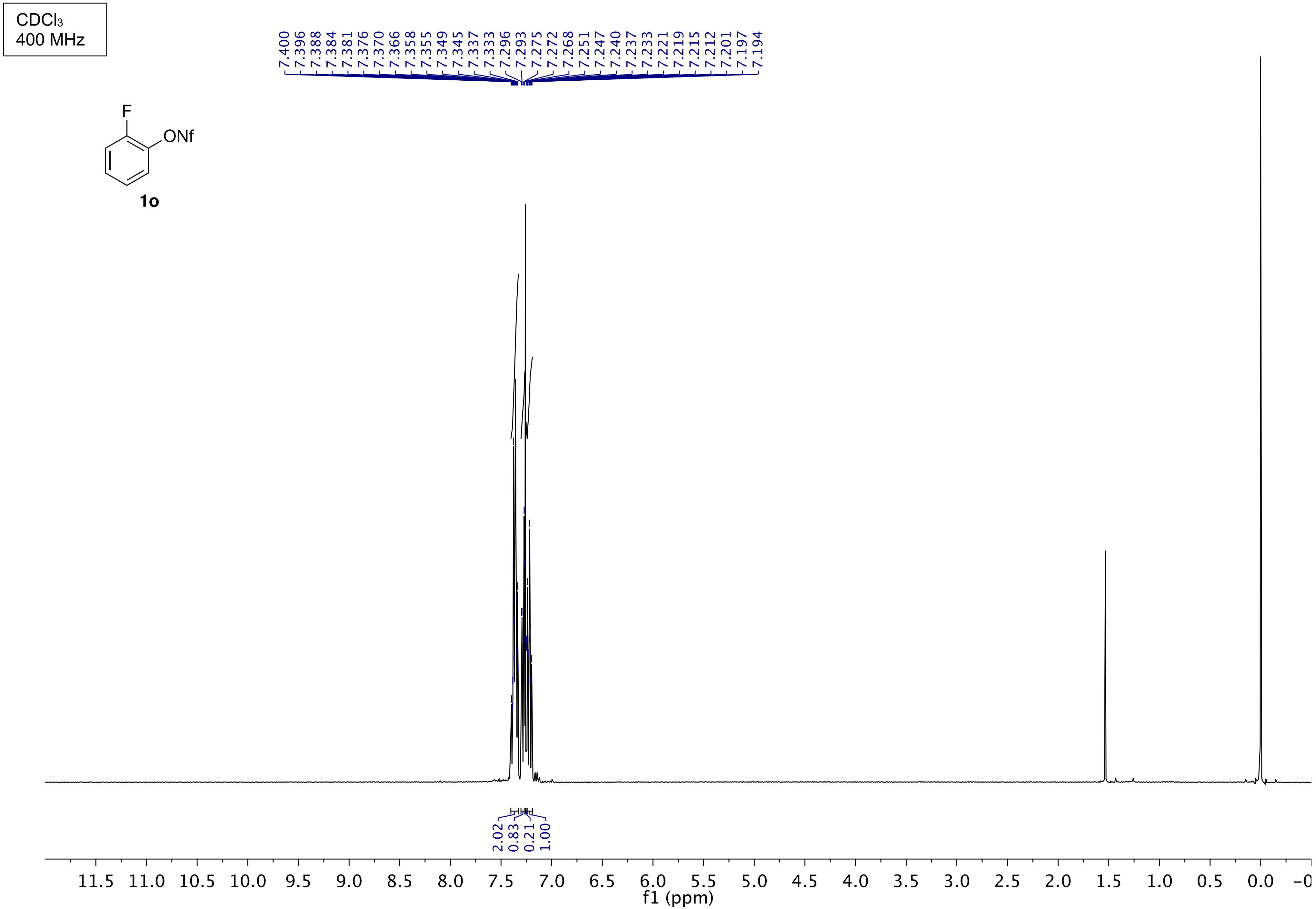
CDCl3
400 MHz
F
ONf
1o
7.400
7.396
7.388
7.384
7.381
7.376
7.370
7.366
7.358
7.355
7.349
7.345
7.337
7.333
7.296
7.293
7.275
7.272
7.268
7.251
7.247
7.240
7.237
7.233
7.221
7.219
7.215
7.212
7.201
7.197
7.194
2.02
0.83
0.21
1.00
11.5 11.0 10.5 10.0 9.5 9.0 8.5 8.0 7.5 7.0 6.5 6.0 5.5 5.0 4.5 4.0 3.5 3.0 2.5 2.0 1.5 1.0 0.5 0.0
f1 (ppm)

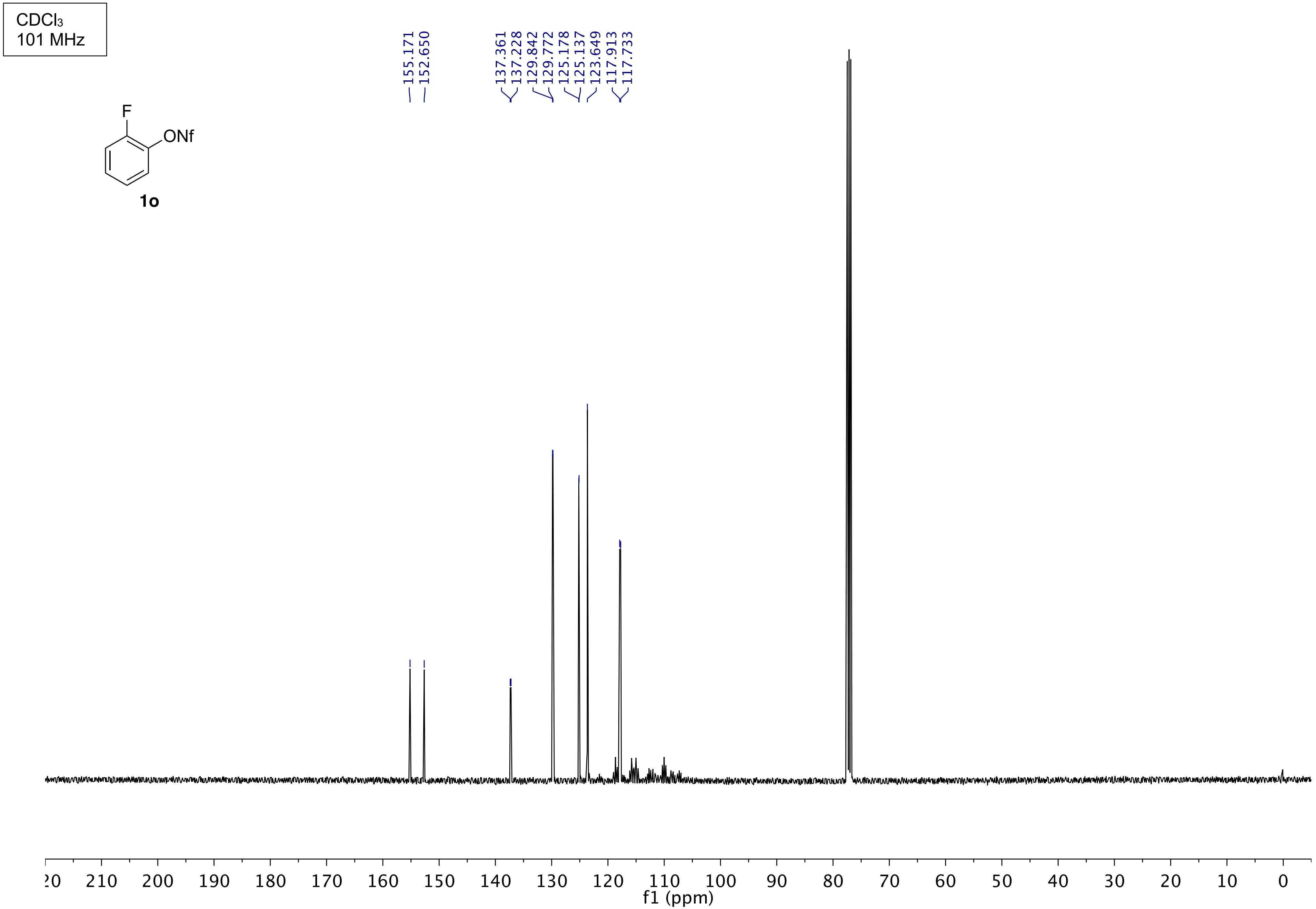

CDCl3
101 MHz
F
ONf
1o
155.171
152.650
137.361
137.228
129.842
129.772
125.178
125.137
123.649
117.913
117.733
20 210 200 190 180 170 160 150 140 130 120 110 100 90 80 70 60 50 40 30 20 10 0
f1 (ppm)

CDCl3
400 MHz

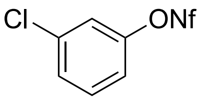

1p

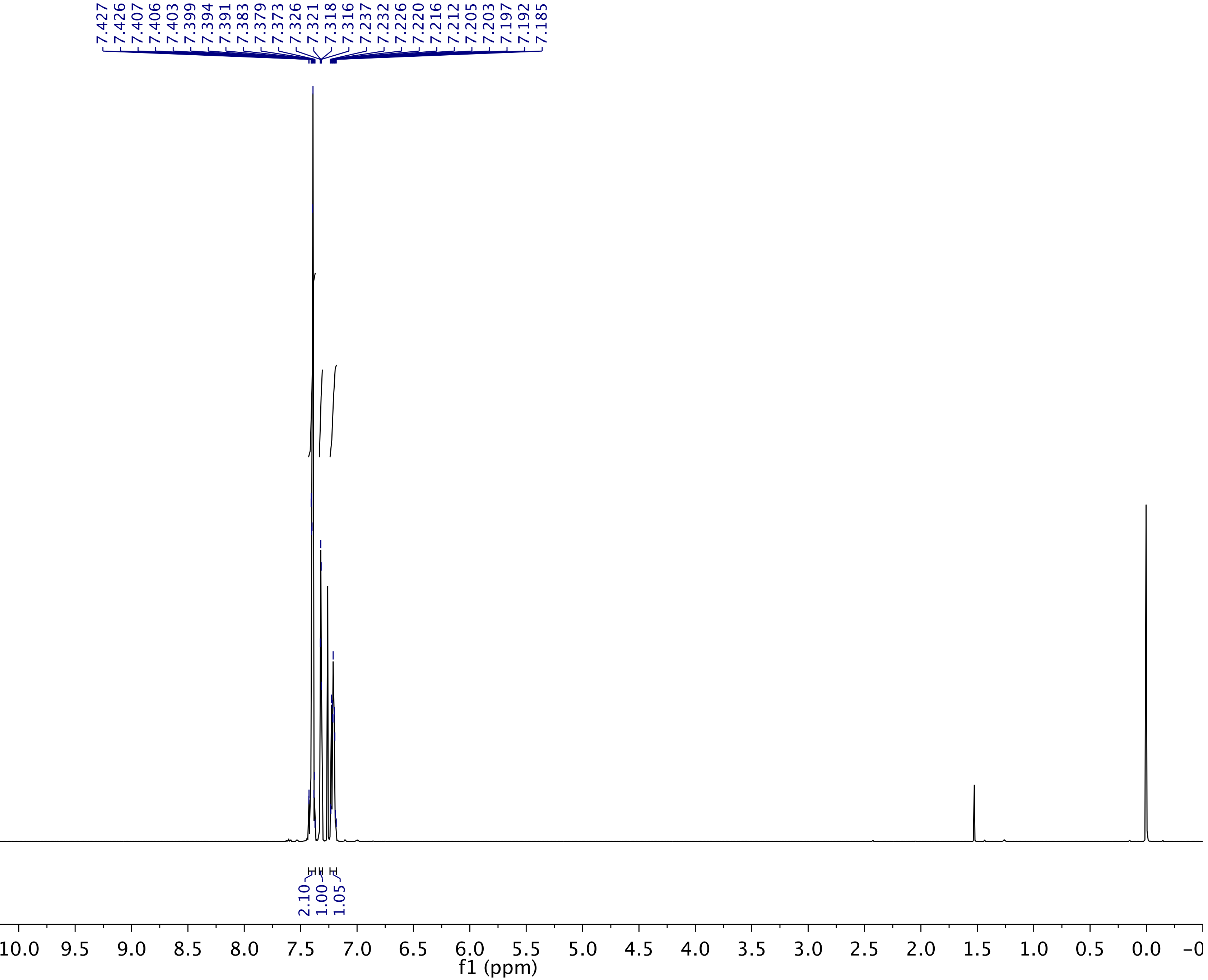

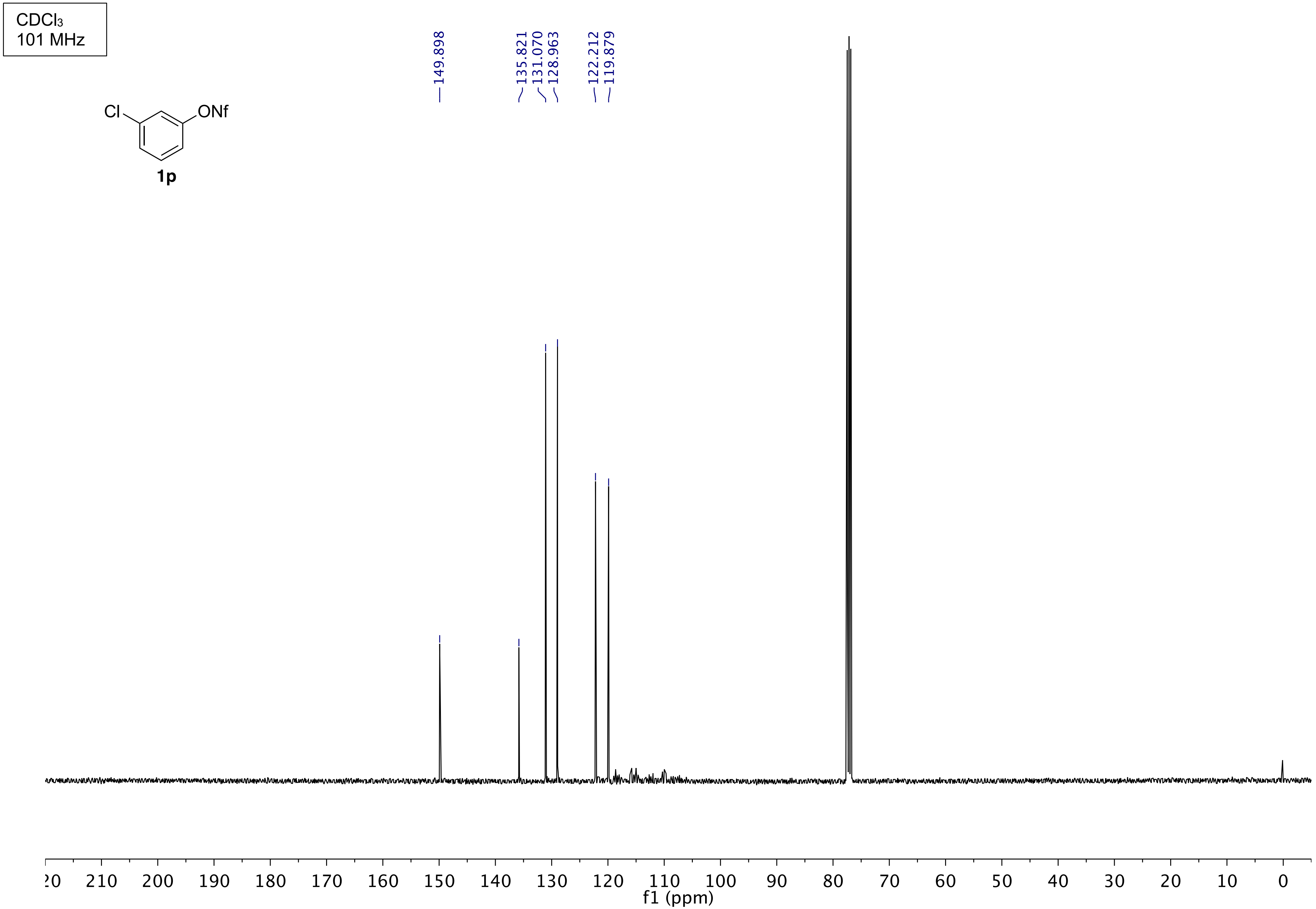
CDCl3
101 MHz
149.898
135.821
131.070
128.963
122.212
119.879
Cl
ONf
1p
220 210 200 190 180 170 160 150 140 130 120 110 100 90 80 70 60 50 40 30 20 10 0
f1 (ppm)

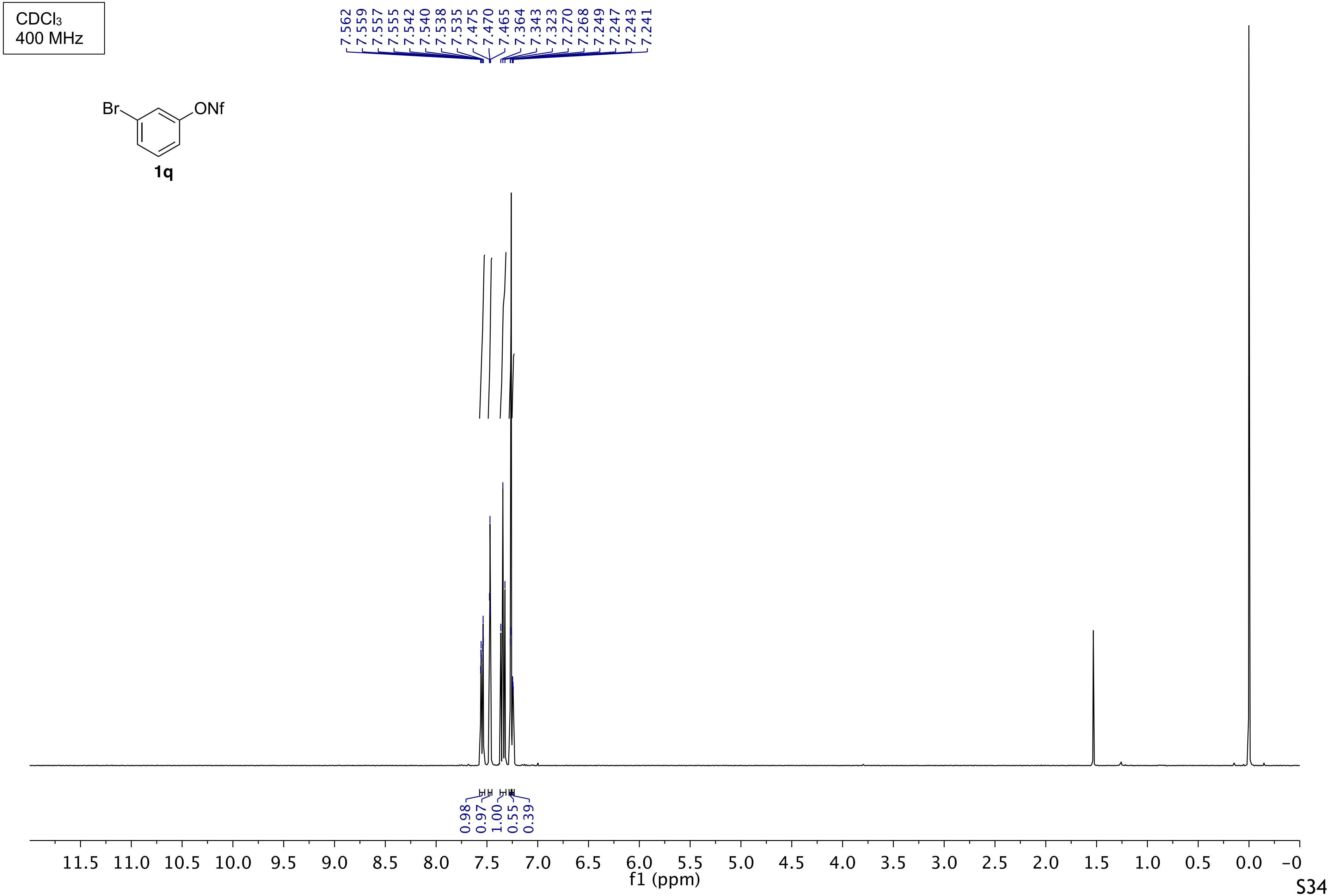

CDCl$_3$
400 MHz
7.562
7.559
7.557
7.555
7.542
7.540
7.538
7.535
7.475
7.470
7.465
7.364
7.343
7.323
7.270
7.268
7.249
7.247
7.243
7.241
Br
ONf
1q
0.98
0.97
1.00
0.55
0.39
11.5 11.0 10.5 10.0 9.5 9.0 8.5 8.0 7.5 7.0 6.5 6.0 5.5 5.0 4.5 4.0 3.5 3.0 2.5 2.0 1.5 1.0 0.5 0.0 –0
f1 (ppm)

CDCl$_3$
101 MHz

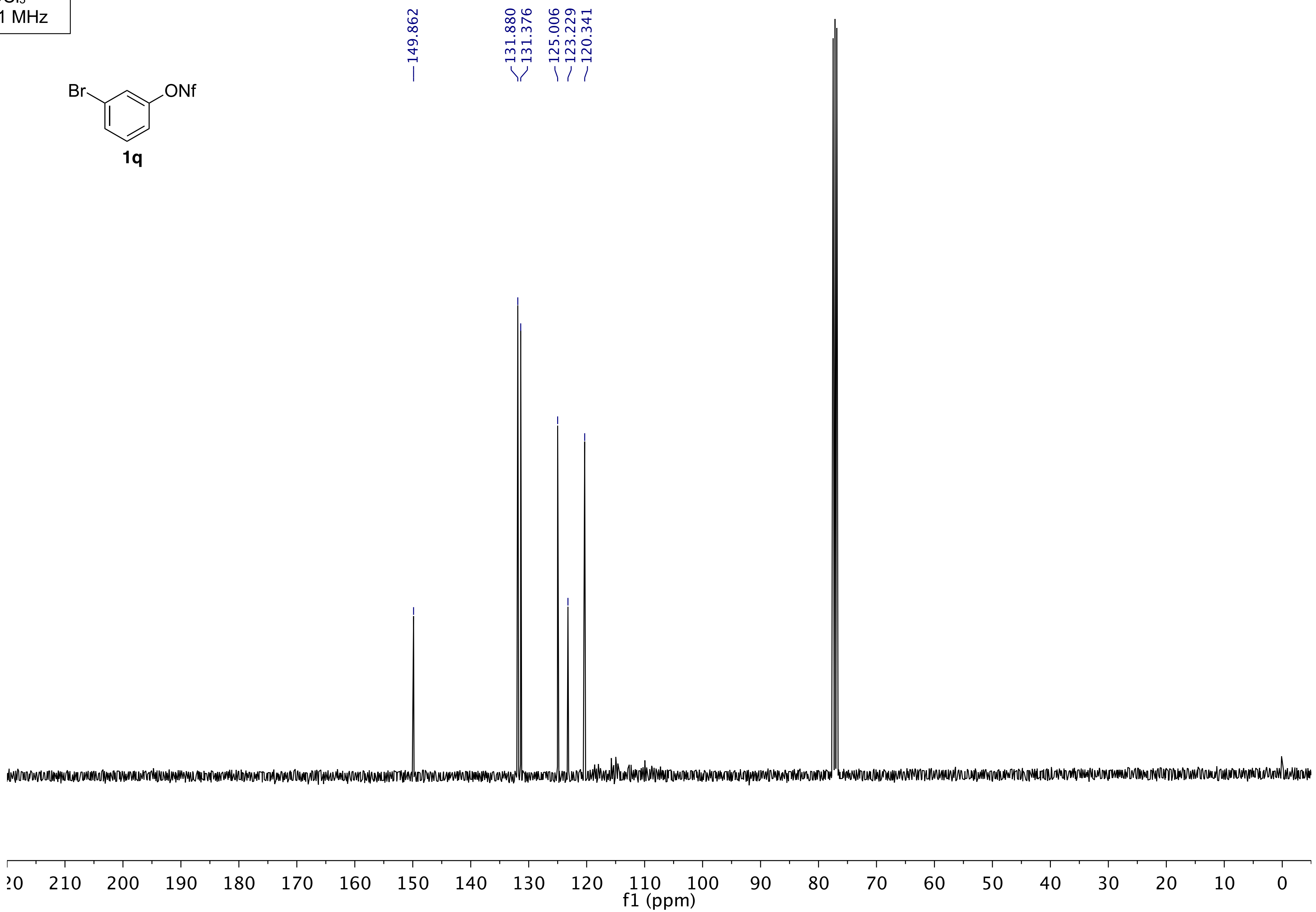

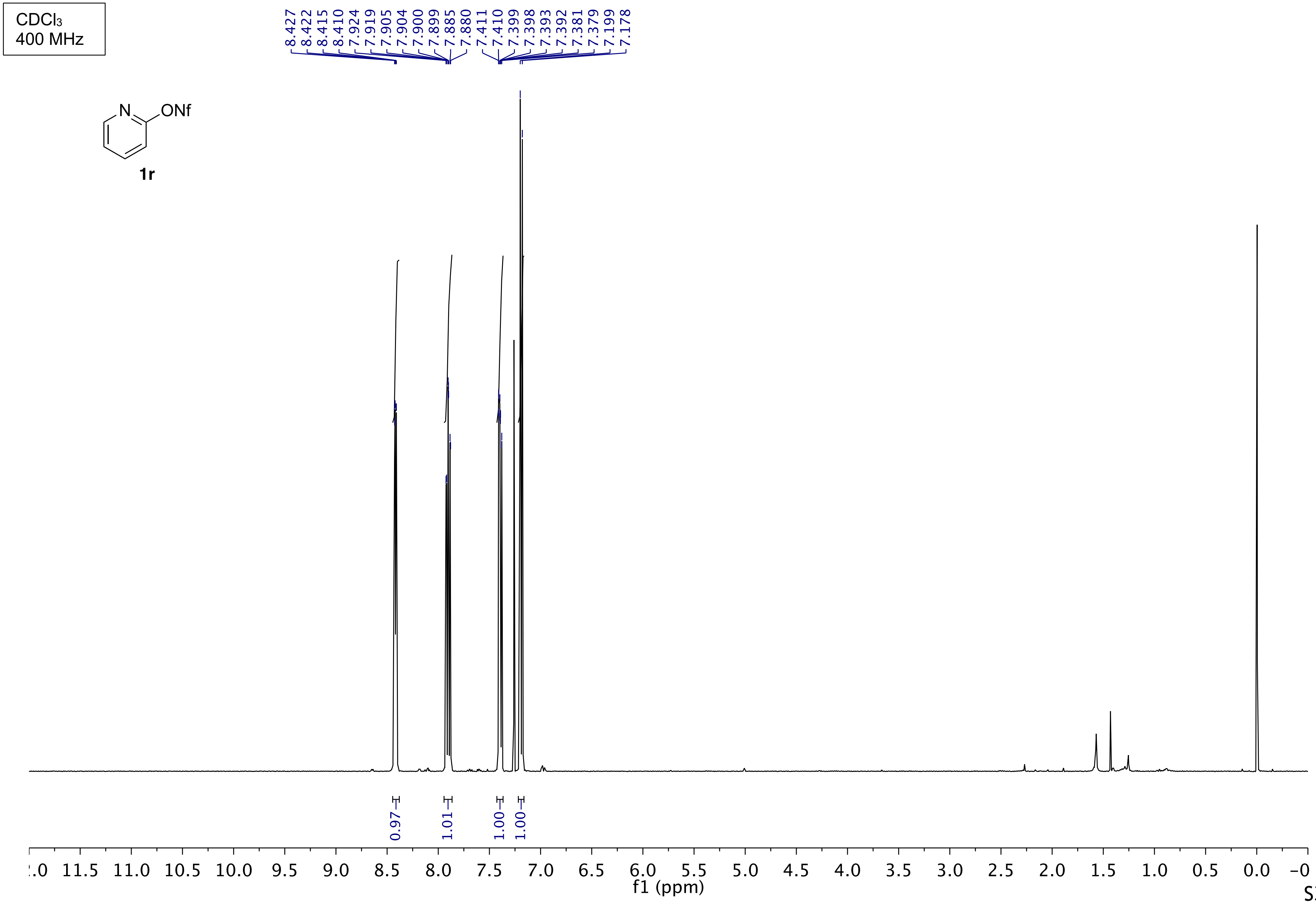
CDCl3
400 MHz
8.427
8.422
8.415
8.410
7.924
7.919
7.905
7.904
7.900
7.899
7.885
7.880
7.411
7.410
7.399
7.398
7.393
7.392
7.381
7.379
7.199
7.178
N
ONf
1r
0.97
1.01
1.00
1.00
11.5 11.0 10.5 10.0 9.5 9.0 8.5 8.0 7.5 7.0 6.5 6.0 5.5 5.0 4.5 4.0 3.5 3.0 2.5 2.0 1.5 1.0 0.5 0.0
f1 (ppm)

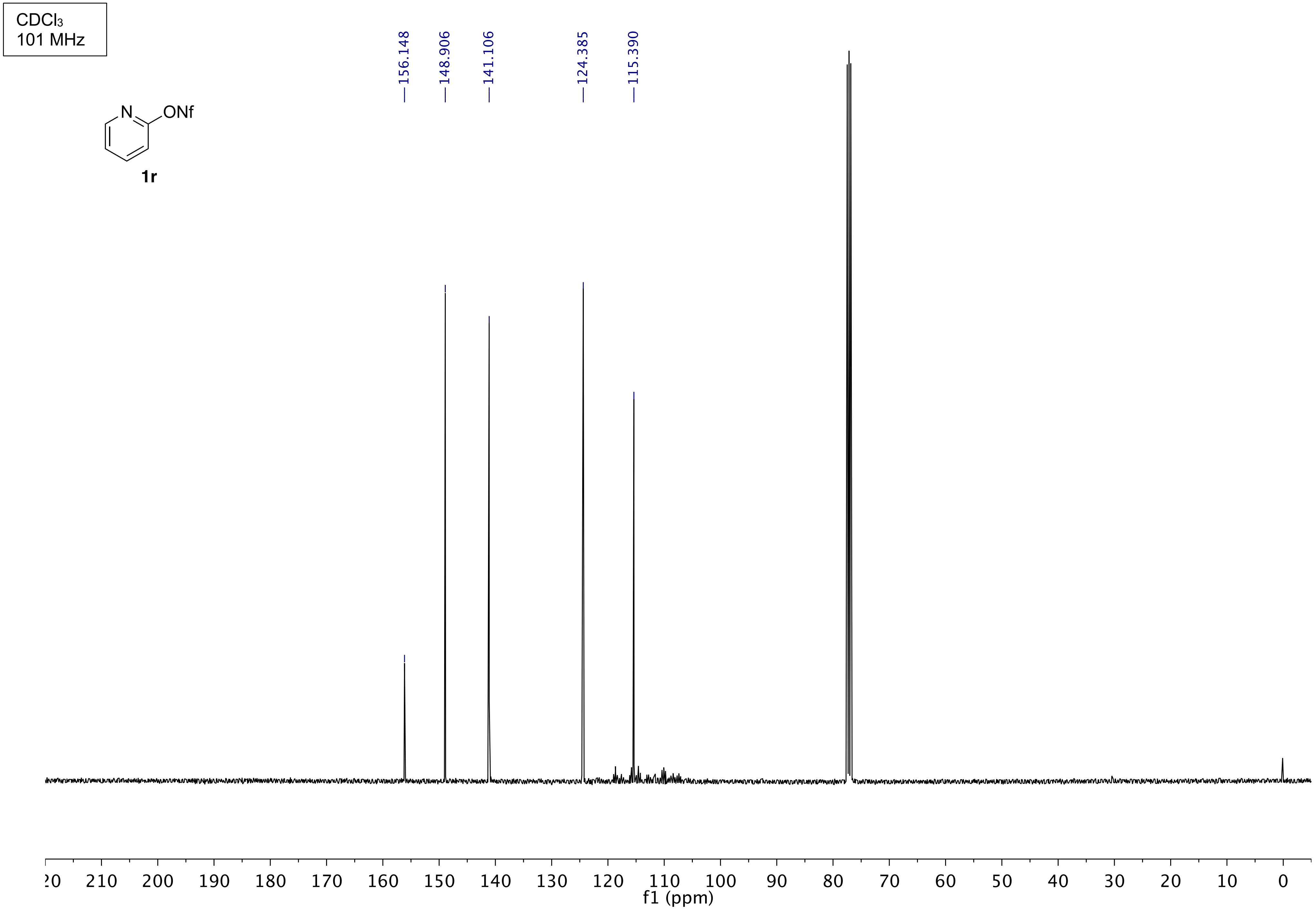

CDCl$_3$
101 MHz
N
ONf
1r
156.148
148.906
141.106
124.385
115.390
20 210 200 190 180 170 160 150 140 130 120 110 100 90 80 70 60 50 40 30 20 10 0
f1 (ppm)

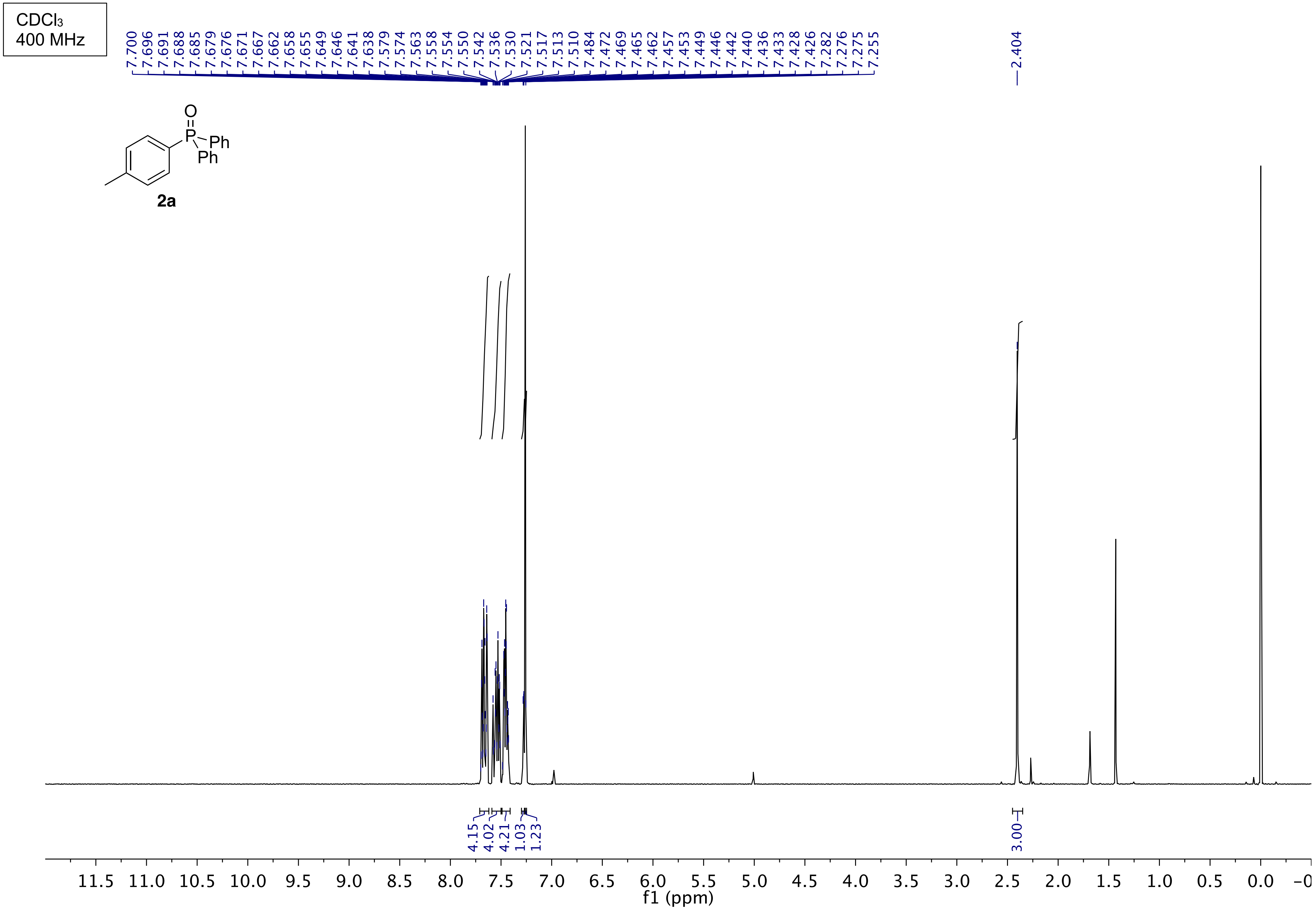
CDCl3
400 MHz
7.700
7.696
7.691
7.688
7.685
7.679
7.676
7.671
7.667
7.662
7.658
7.655
7.649
7.646
7.641
7.638
7.579
7.574
7.563
7.558
7.554
7.550
7.542
7.536
7.530
7.521
7.517
7.513
7.510
7.484
7.472
7.469
7.465
7.462
7.457
7.453
7.449
7.446
7.442
7.440
7.436
7.433
7.428
7.426
7.282
7.276
7.275
7.255
2.404
O
P
Ph
Ph
2a
4.15
4.02
4.21
1.03
1.23
3.00
11.5 11.0 10.5 10.0 9.5 9.0 8.5 8.0 7.5 7.0 6.5 6.0 5.5 5.0 4.5 4.0 3.5 3.0 2.5 2.0 1.5 1.0 0.5 0.0
f1 (ppm)

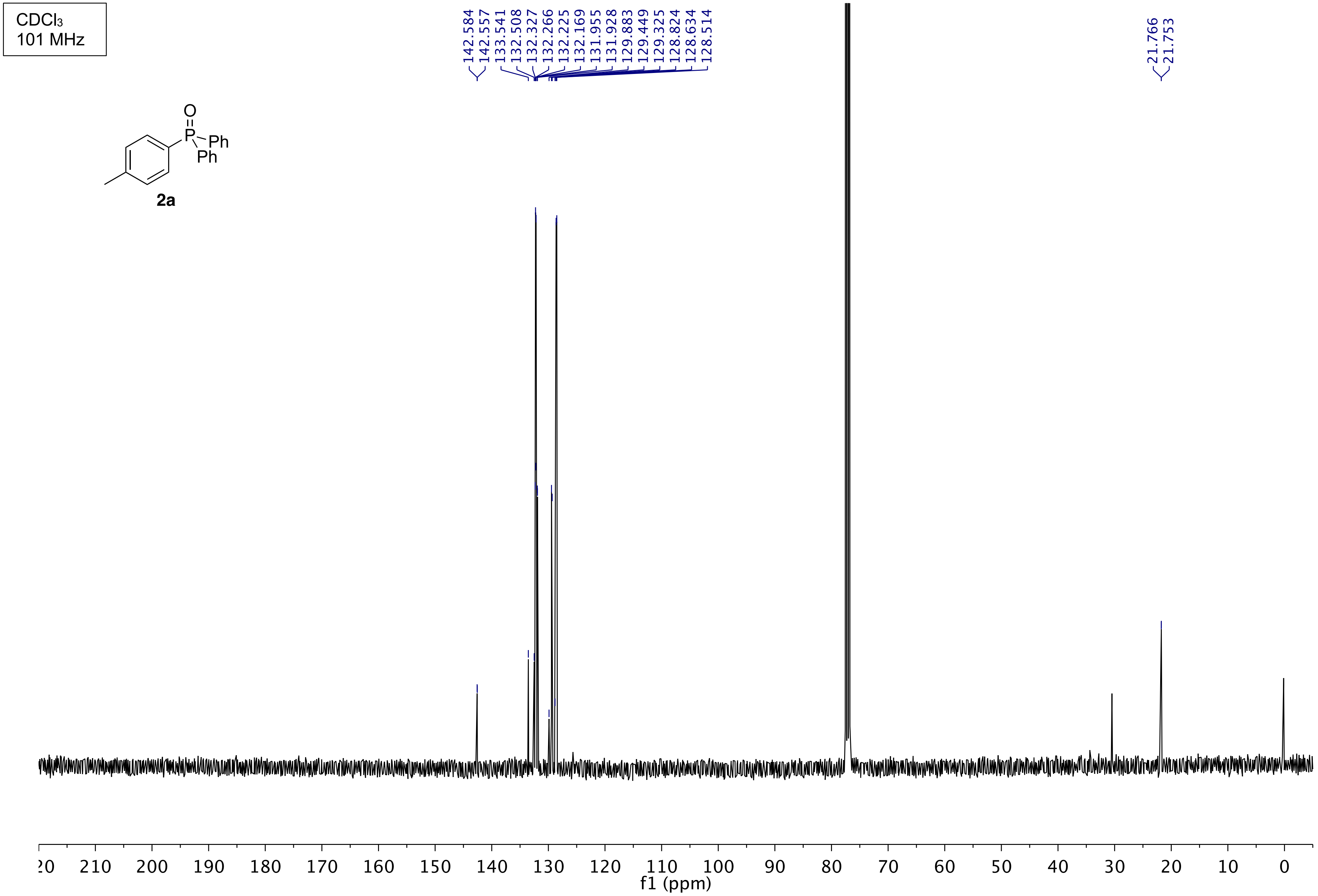

CDCl$_3$
101 MHz
O
P
Ph
Ph
2a
142.584
142.557
133.541
132.508
132.327
132.266
132.225
132.169
131.955
131.928
129.883
129.449
129.325
128.824
128.634
128.514
21.766
21.753
220 210 200 190 180 170 160 150 140 130 120 110 100 90 80 70 60 50 40 30 20 10 0
f1 (ppm)

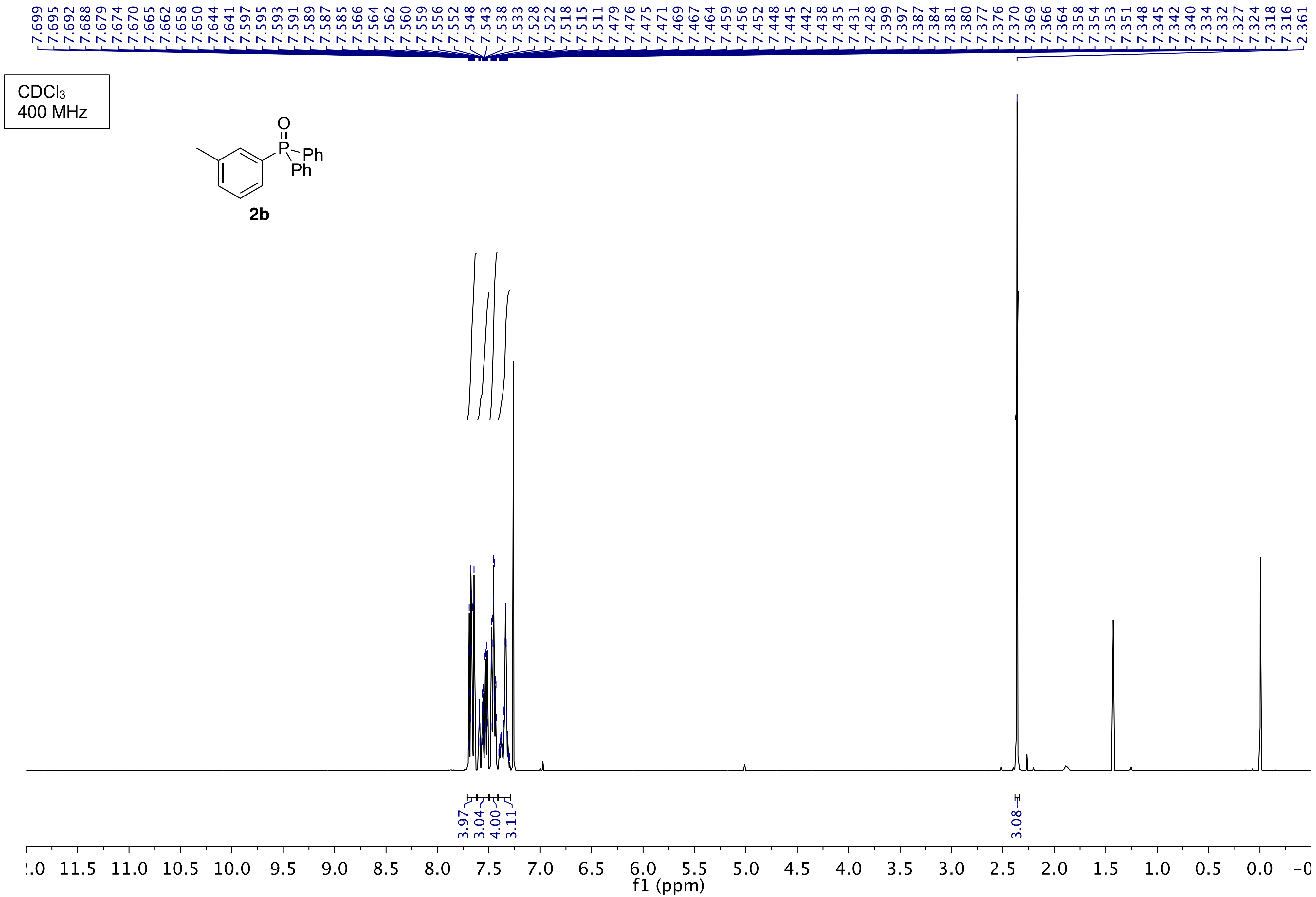

7.699 7.695 7.692 7.688 7.679 7.674 7.670 7.665 7.662 7.658 7.650 7.644 7.641 7.597 7.595 7.593 7.591 7.589 7.587 7.585 7.566 7.564 7.562 7.560 7.559 7.556 7.552 7.548 7.543 7.538 7.533 7.528 7.522 7.518 7.515 7.511 7.479 7.476 7.475 7.471 7.469 7.467 7.464 7.459 7.456 7.452 7.448 7.445 7.442 7.438 7.435 7.431 7.428 7.399 7.397 7.387 7.384 7.381 7.380 7.377 7.376 7.370 7.369 7.366 7.364 7.358 7.354 7.353 7.351 7.348 7.345 7.342 7.340 7.334 7.332 7.327 7.324 7.318 7.316 2.361
CDCl3
400 MHz
O
P
Ph
Ph
2b
3.97
3.04
4.00
3.11
3.08
11.5 11.0 10.5 10.0 9.5 9.0 8.5 8.0 7.5 7.0 6.5 6.0 5.5 5.0 4.5 4.0 3.5 3.0 2.5 2.0 1.5 1.0 0.5 0.0
f1 (ppm)

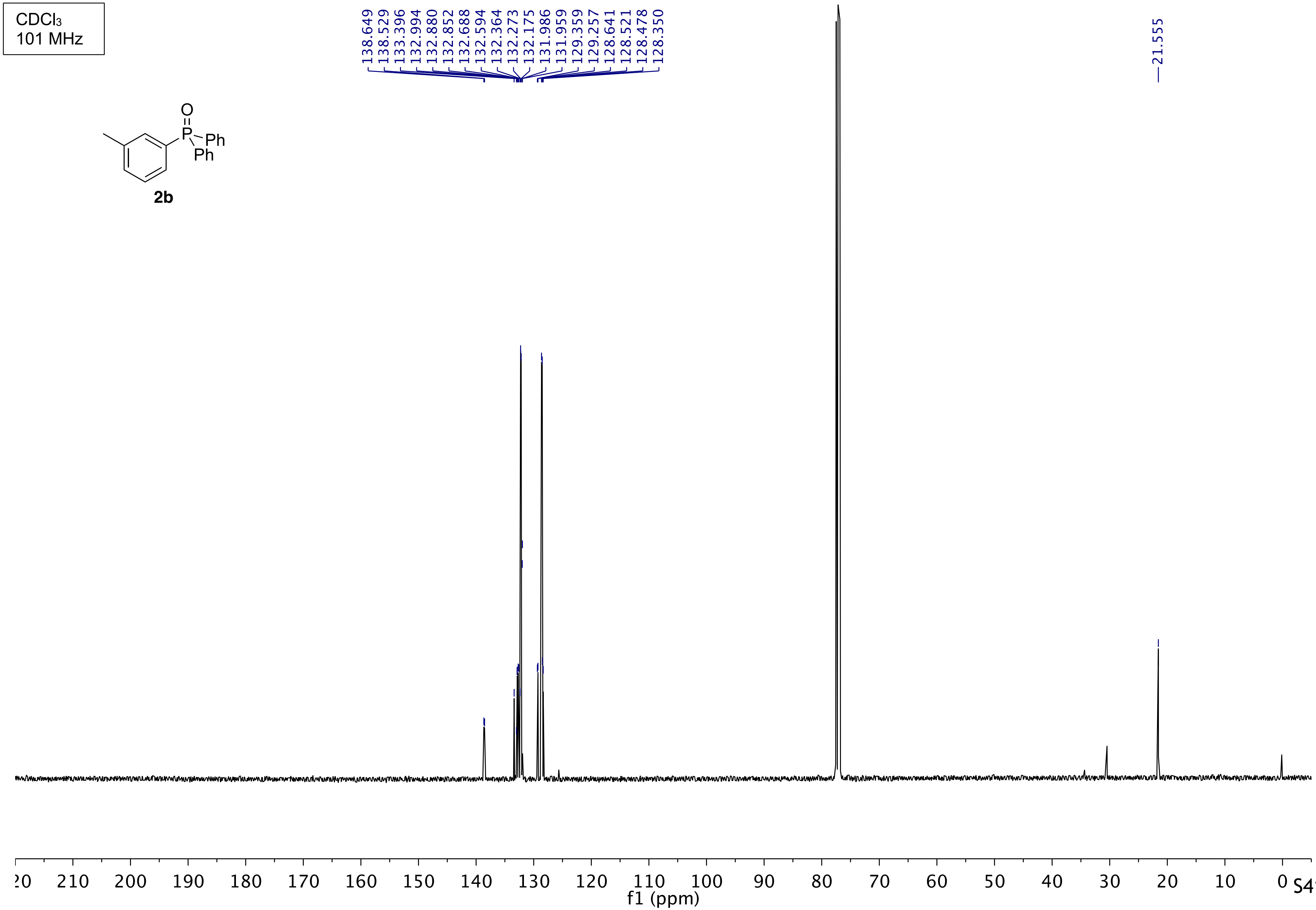
CDCl3
101 MHz
2b
138.649
138.529
133.396
132.994
132.880
132.852
132.688
132.594
132.364
132.273
132.175
131.986
131.959
129.359
129.257
128.641
128.521
128.478
128.350
21.555
f1 (ppm)

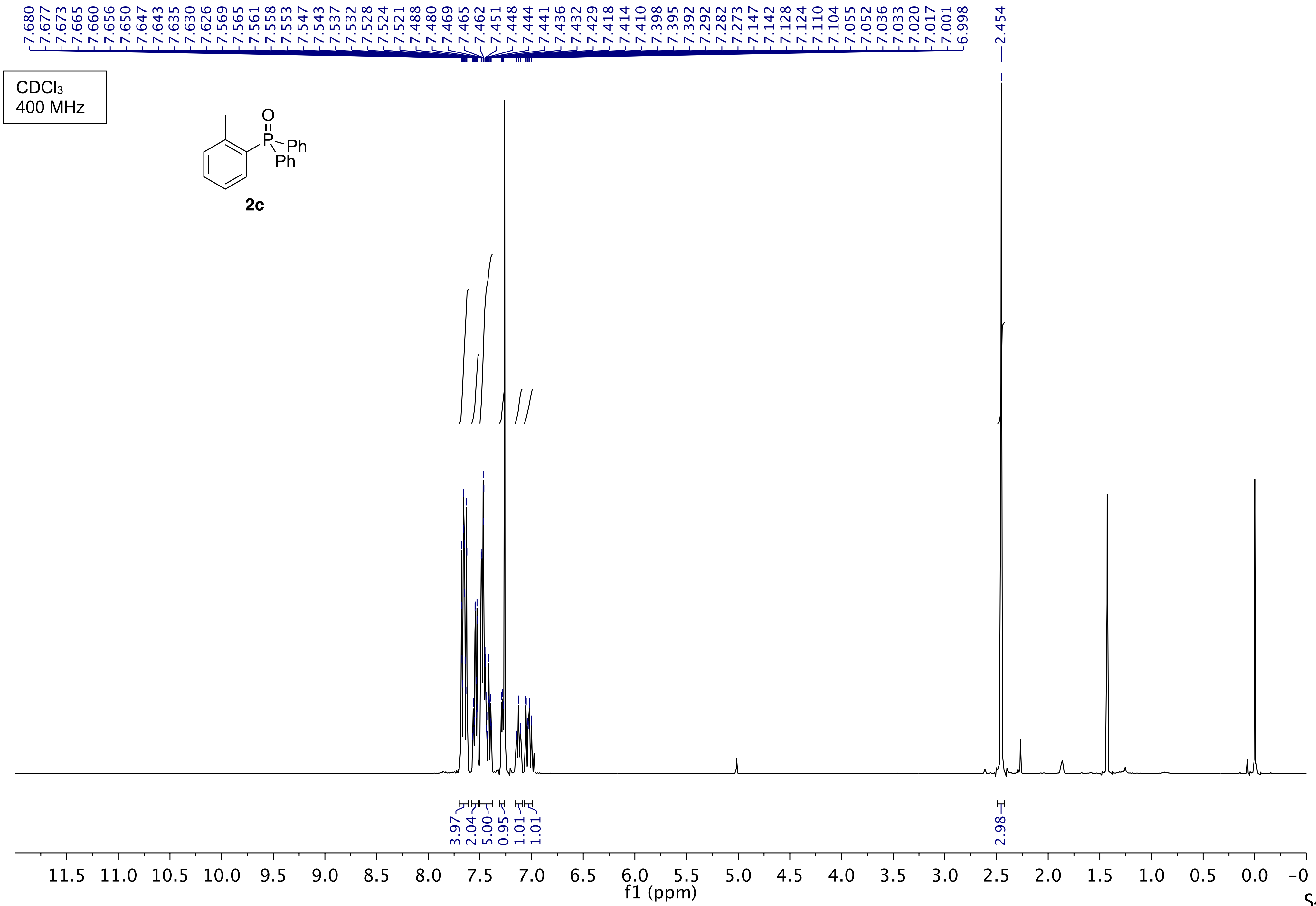
7.680 7.677 7.673 7.665 7.660 7.656 7.650 7.647 7.643 7.635 7.630 7.626 7.569 7.565 7.561 7.558 7.553 7.547 7.543 7.537 7.532 7.528 7.524 7.521 7.488 7.480 7.469 7.465 7.462 7.451 7.448 7.444 7.441 7.436 7.432 7.429 7.418 7.414 7.410 7.398 7.395 7.392 7.292 7.282 7.273 7.147 7.142 7.128 7.124 7.110 7.104 7.055 7.052 7.036 7.033 7.020 7.017 7.001 6.998
2.454
CDCl3
400 MHz
O
P
Ph
Ph
2c
3.97
2.04
5.00
0.95
1.01
1.01
2.98
11.5 11.0 10.5 10.0 9.5 9.0 8.5 8.0 7.5 7.0 6.5 6.0 5.5 5.0 4.5 4.0 3.5 3.0 2.5 2.0 1.5 1.0 0.5 0.0 -0
f1 (ppm)

$CDCl_3$
101 MHz

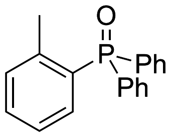

2c

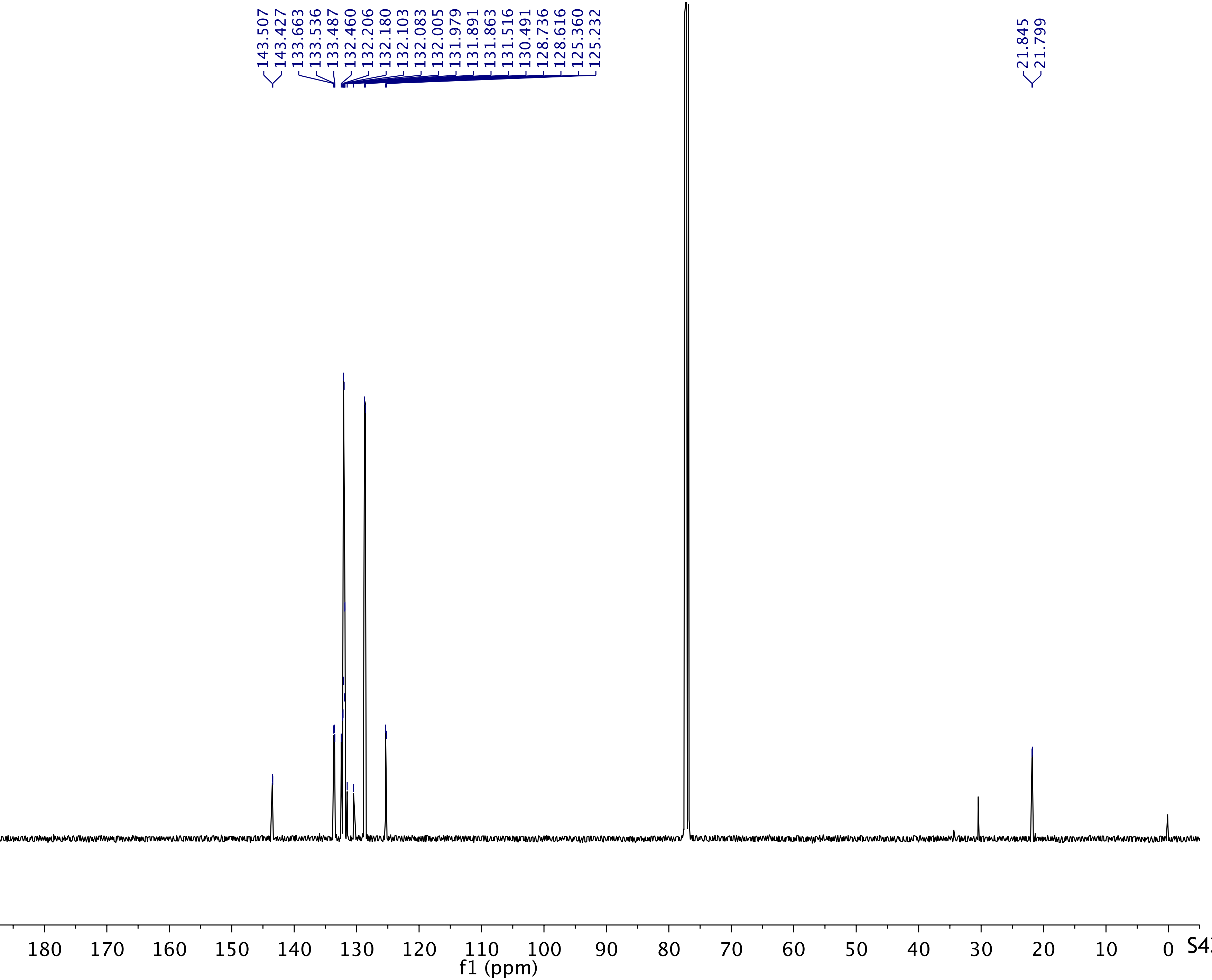

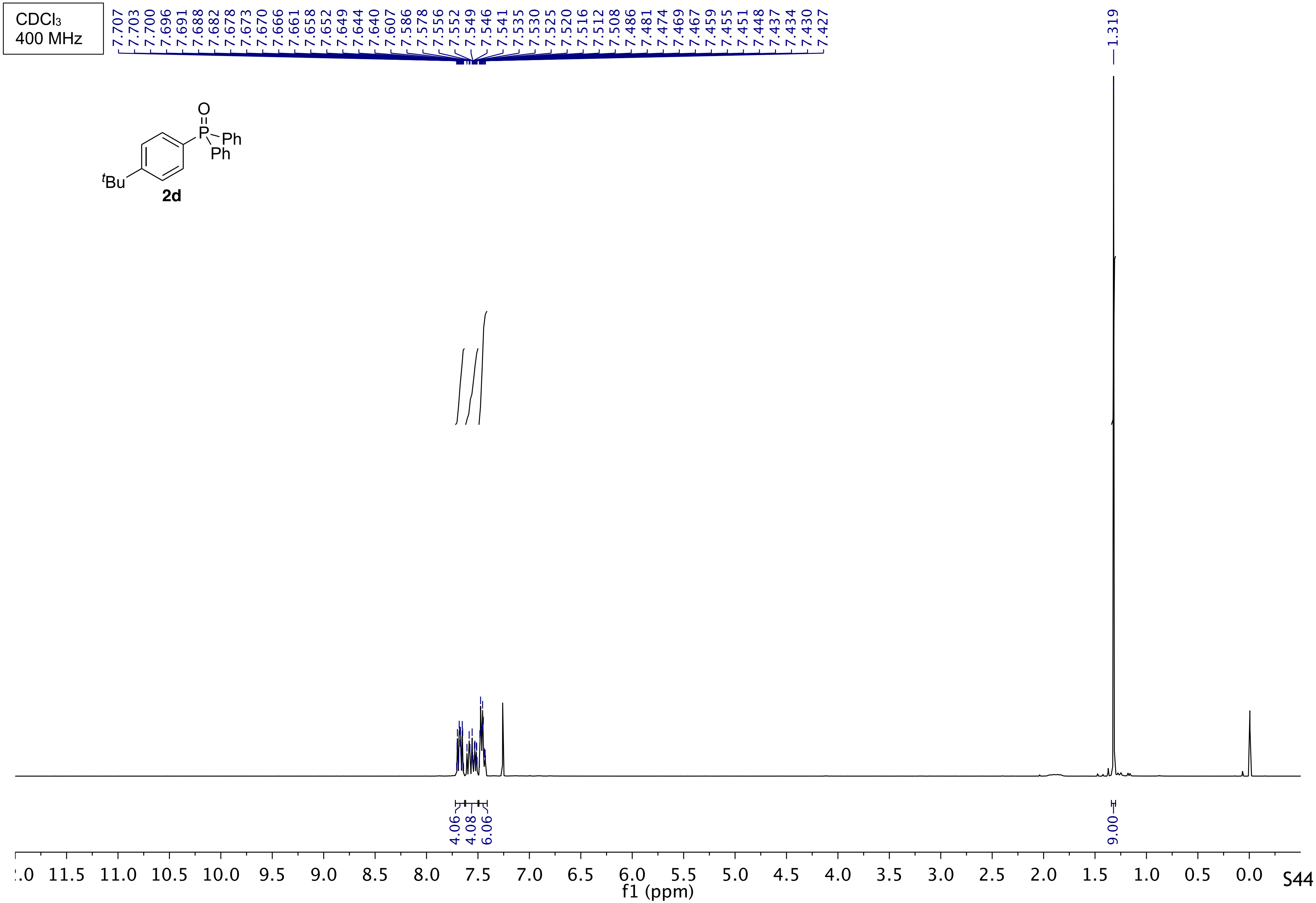
CDCl3
400 MHz
7.707
7.703
7.700
7.696
7.691
7.688
7.682
7.678
7.673
7.670
7.666
7.661
7.658
7.652
7.649
7.644
7.640
7.607
7.586
7.578
7.556
7.552
7.549
7.546
7.541
7.535
7.530
7.525
7.520
7.516
7.512
7.508
7.486
7.481
7.474
7.469
7.467
7.459
7.455
7.451
7.448
7.437
7.434
7.430
7.427
1.319
O
P
Ph
Ph
tBu
2d
4.06
4.08
6.06
9.00
.0 11.5 11.0 10.5 10.0 9.5 9.0 8.5 8.0 7.5 7.0 6.5 6.0 5.5 5.0 4.5 4.0 3.5 3.0 2.5 2.0 1.5 1.0 0.5 0.0
f1 (ppm)

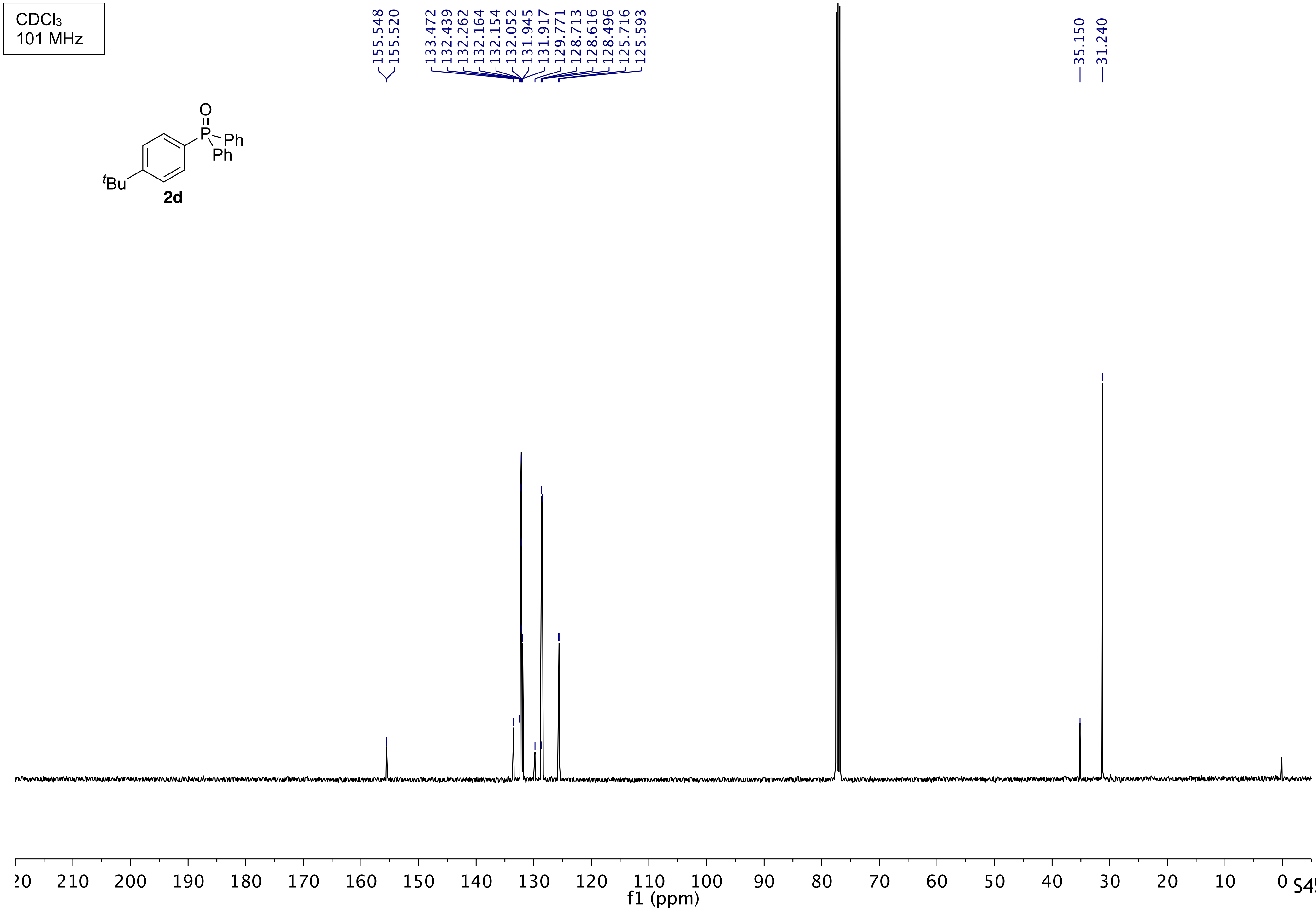

CDCl3
101 MHz
O
P
Ph
Ph
tBu
2d
155.548
155.520
133.472
132.439
132.262
132.164
132.154
132.052
131.945
131.917
129.771
128.713
128.616
128.496
125.716
125.593
35.150
31.240
20 210 200 190 180 170 160 150 140 130 120 110 100 90 80 70 60 50 40 30 20 10 0
f1 (ppm)

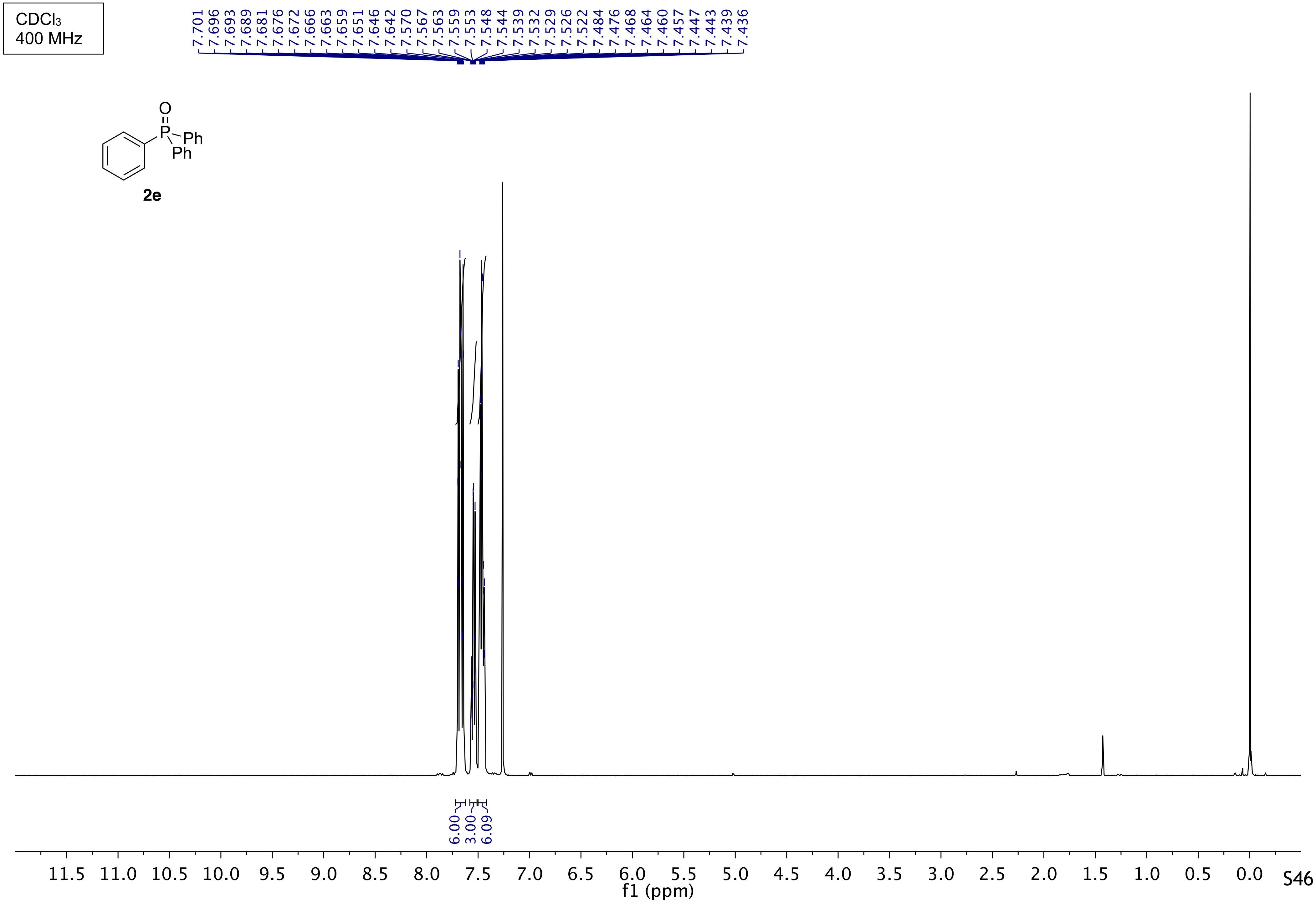

CDCl3
400 MHz
7.701 7.696 7.693 7.689 7.681 7.676 7.672 7.666 7.663 7.659 7.651 7.646 7.642 7.570 7.567 7.563 7.559 7.553 7.548 7.544 7.539 7.532 7.529 7.526 7.522 7.484 7.476 7.468 7.464 7.460 7.457 7.447 7.443 7.439 7.436
O
P
Ph
Ph
2e
6.00
3.00
6.09
11.5 11.0 10.5 10.0 9.5 9.0 8.5 8.0 7.5 7.0 6.5 6.0 5.5 5.0 4.5 4.0 3.5 3.0 2.5 2.0 1.5 1.0 0.5 0.0
f1 (ppm)

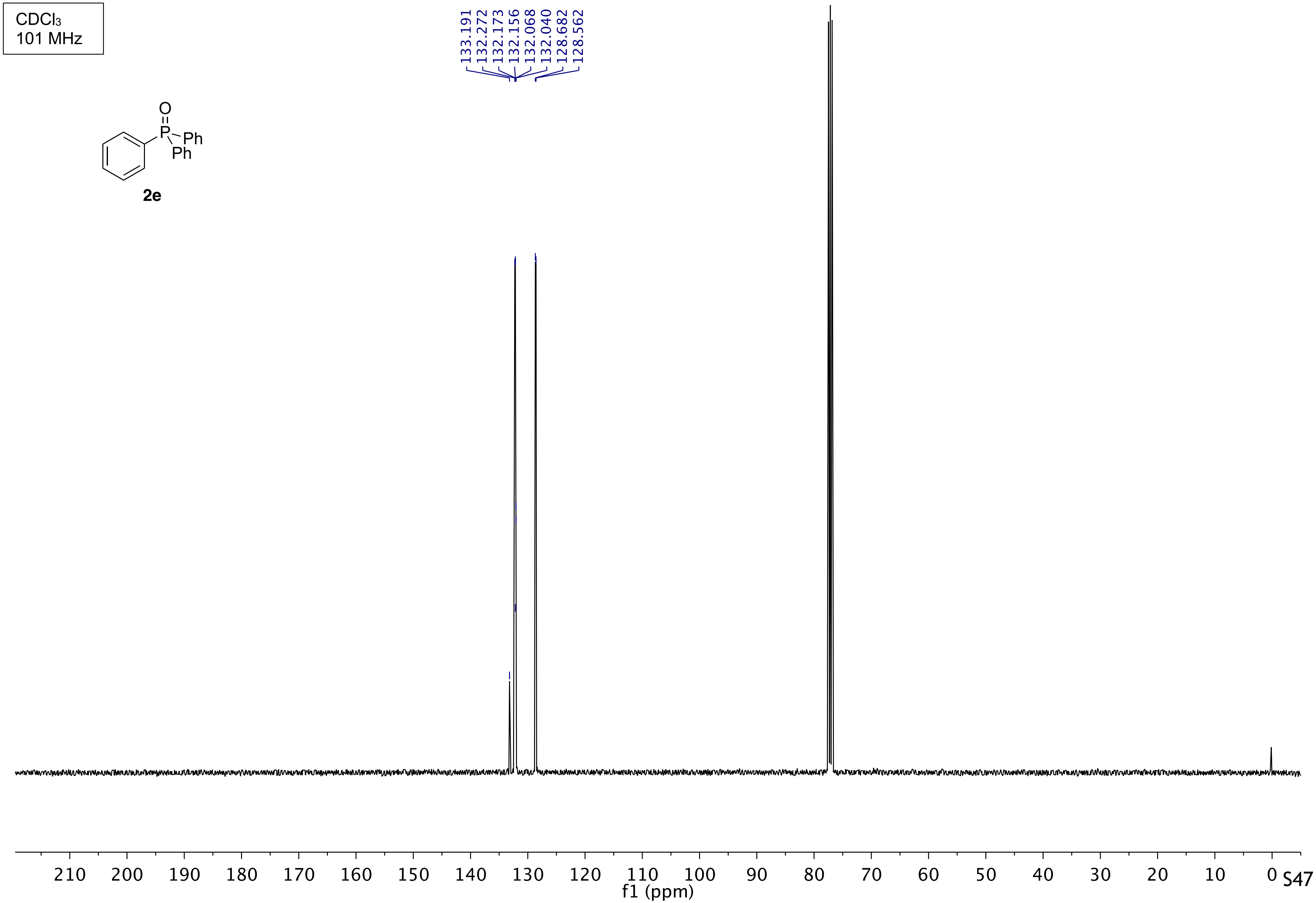
CDCl3
101 MHz
133.191
132.272
132.173
132.156
132.068
132.040
128.682
128.562
O
P
Ph
Ph
2e
210
200
190
180
170
160
150
140
130
120
110
100
90
80
70
60
50
40
30
20
10
0
f1 (ppm)

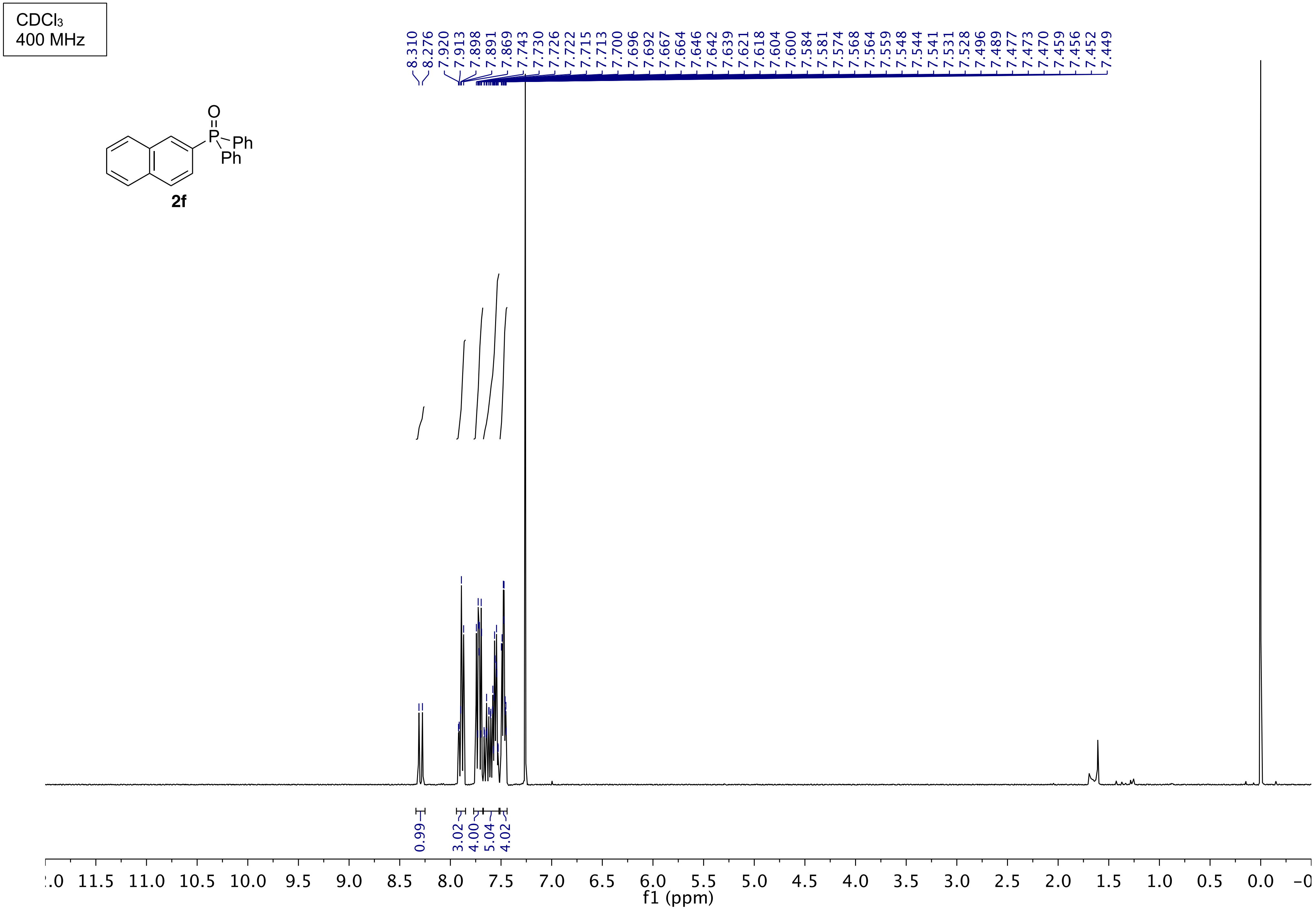
CDCl3
400 MHz
O
P
Ph
Ph
2f
8.310
8.276
7.920
7.913
7.898
7.891
7.869
7.743
7.730
7.726
7.722
7.715
7.713
7.700
7.696
7.692
7.667
7.664
7.646
7.642
7.639
7.621
7.618
7.604
7.600
7.584
7.581
7.574
7.568
7.564
7.559
7.548
7.544
7.541
7.531
7.528
7.496
7.489
7.477
7.473
7.470
7.459
7.456
7.452
7.449
0.99
3.02
4.00
5.04
4.02
11.5 11.0 10.5 10.0 9.5 9.0 8.5 8.0 7.5 7.0 6.5 6.0 5.5 5.0 4.5 4.0 3.5 3.0 2.5 2.0 1.5 1.0 0.5 0.0
f1 (ppm)

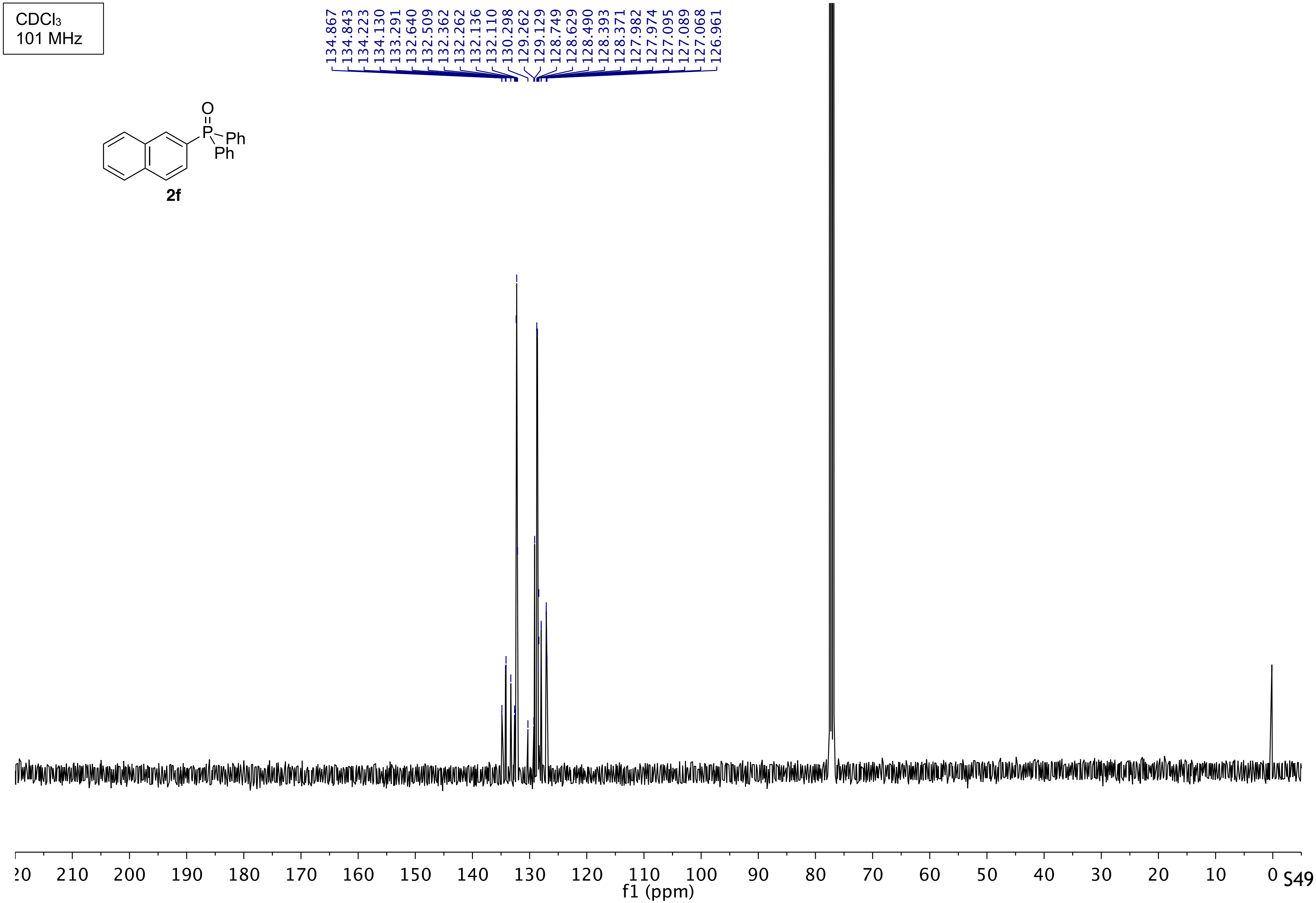
CDCl3
101 MHz
134.867
134.843
134.223
134.130
133.291
132.640
132.509
132.362
132.262
132.136
132.110
130.298
129.262
129.129
128.749
128.629
128.490
128.393
128.371
127.982
127.974
127.095
127.089
127.068
126.961
O
P
Ph
Ph
2f
20
210
200
190
180
170
160
150
140
130
120
110
100
90
80
70
60
50
40
30
20
10
0
f1 (ppm)

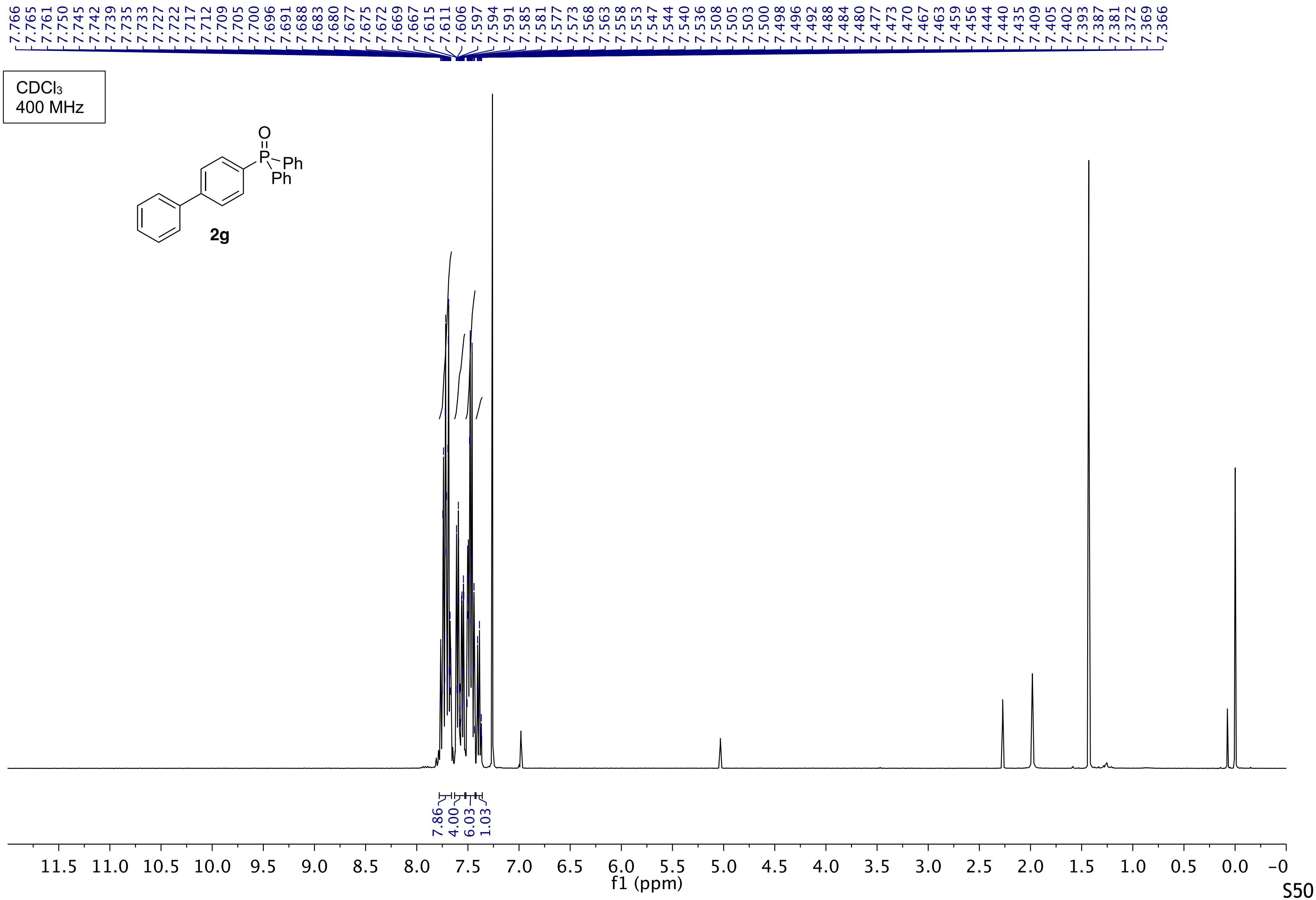

7.766 7.765 7.761 7.750 7.745 7.742 7.739 7.735 7.733 7.727 7.722 7.717 7.712 7.709 7.705 7.700 7.696 7.691 7.688 7.683 7.680 7.677 7.675 7.672 7.669 7.667 7.615 7.611 7.606 7.597 7.594 7.591 7.585 7.581 7.577 7.573 7.568 7.563 7.558 7.553 7.547 7.544 7.540 7.536 7.508 7.505 7.503 7.500 7.498 7.496 7.492 7.488 7.484 7.480 7.477 7.473 7.470 7.467 7.463 7.459 7.456 7.444 7.440 7.435 7.409 7.405 7.402 7.393 7.387 7.381 7.372 7.369 7.366
CDCl3
400 MHz
O
P
Ph
Ph
2g
7.86
4.00
6.03
1.03
11.5 11.0 10.5 10.0 9.5 9.0 8.5 8.0 7.5 7.0 6.5 6.0 5.5 5.0 4.5 4.0 3.5 3.0 2.5 2.0 1.5 1.0 0.5 0.0 –0
f1 (ppm)

CDCl3
101 MHz

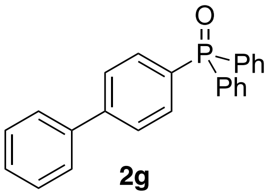

2g

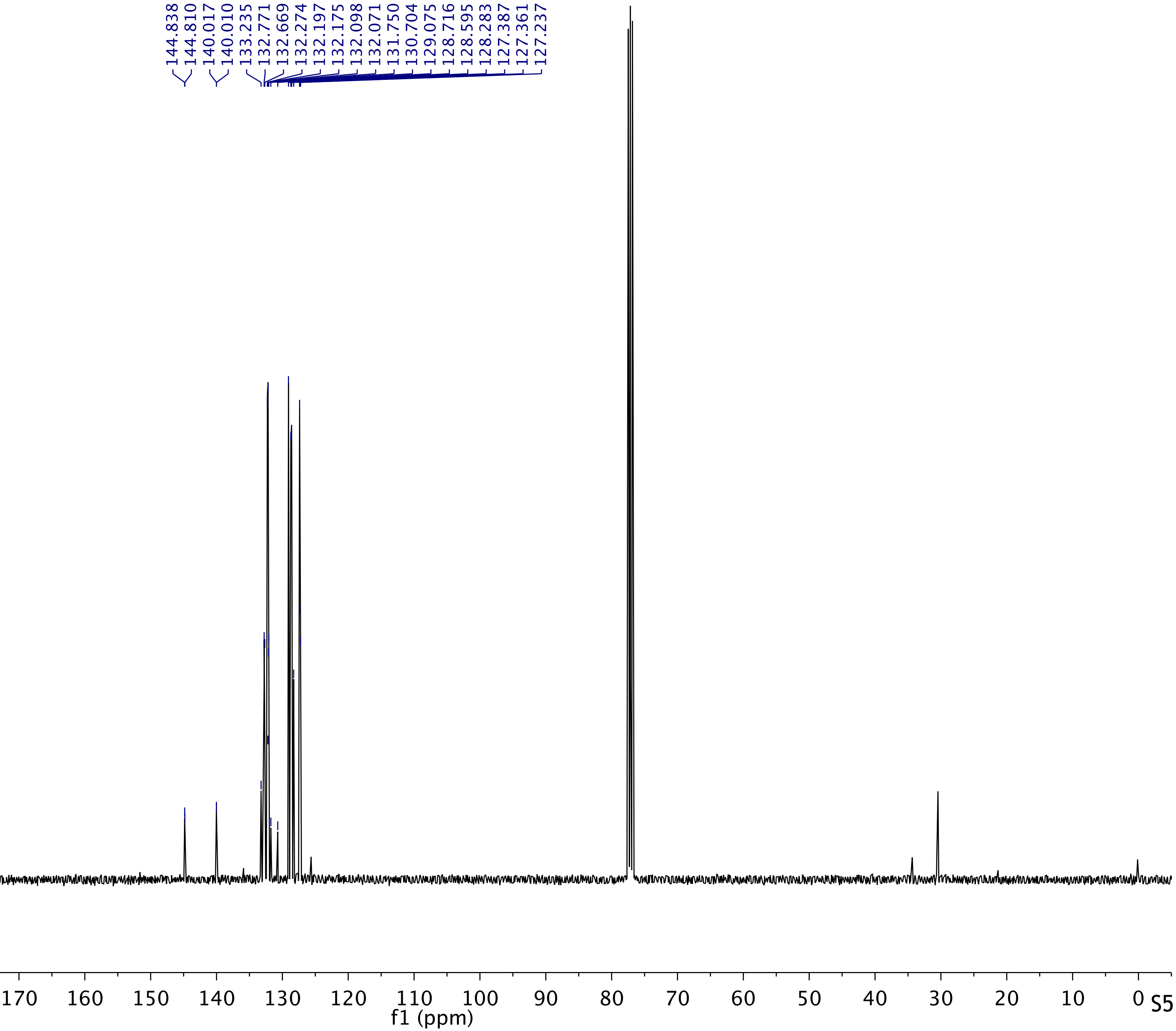

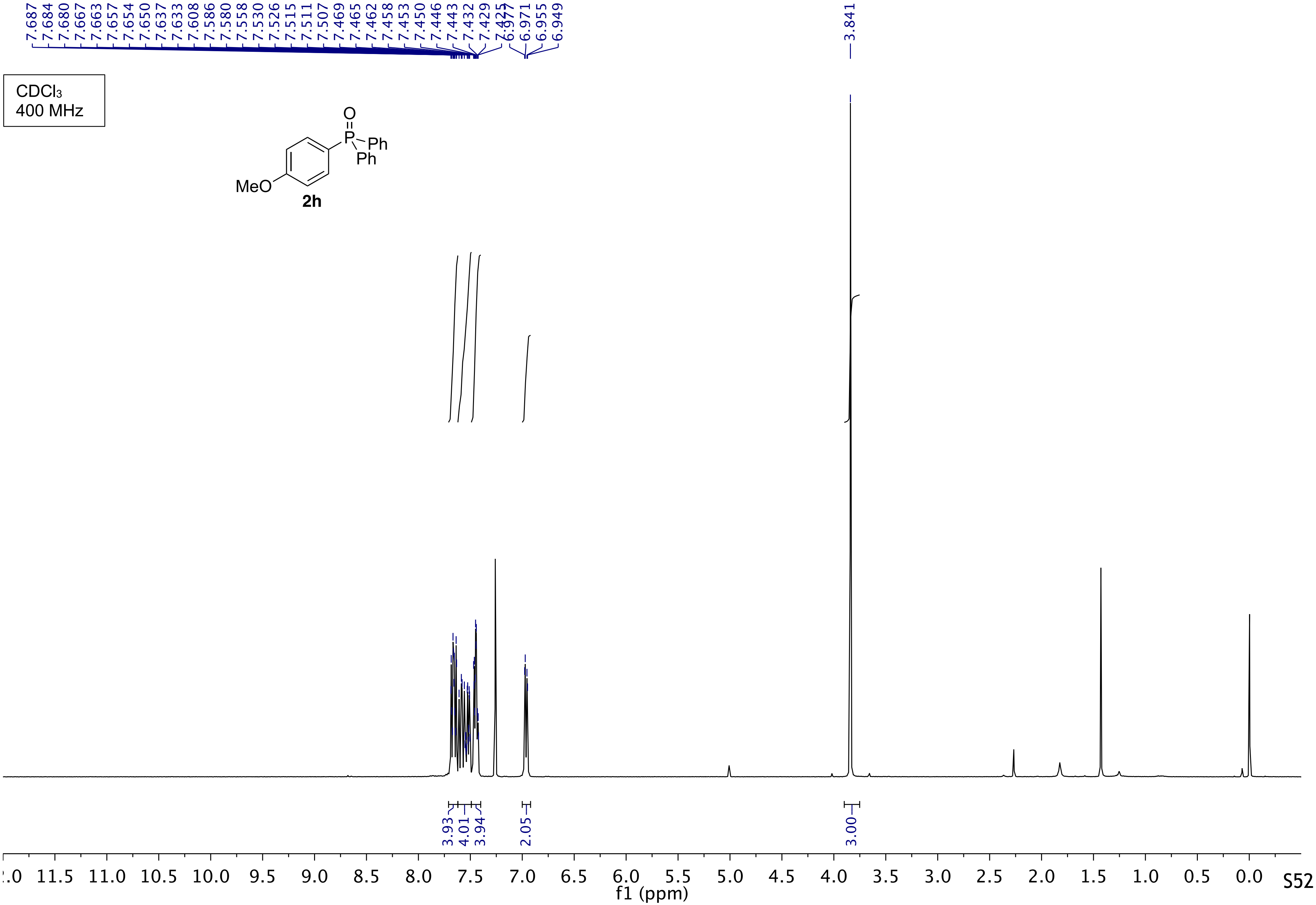
7.687
7.684
7.680
7.667
7.663
7.657
7.654
7.650
7.637
7.633
7.608
7.586
7.580
7.558
7.530
7.526
7.515
7.511
7.507
7.469
7.465
7.462
7.458
7.453
7.450
7.446
7.443
7.432
7.429
7.425
6.977
6.971
6.955
6.949
3.841
CDCl3
400 MHz
O
P
Ph
Ph
MeO
2h
3.93
4.01
3.94
2.05
3.00
f1 (ppm)

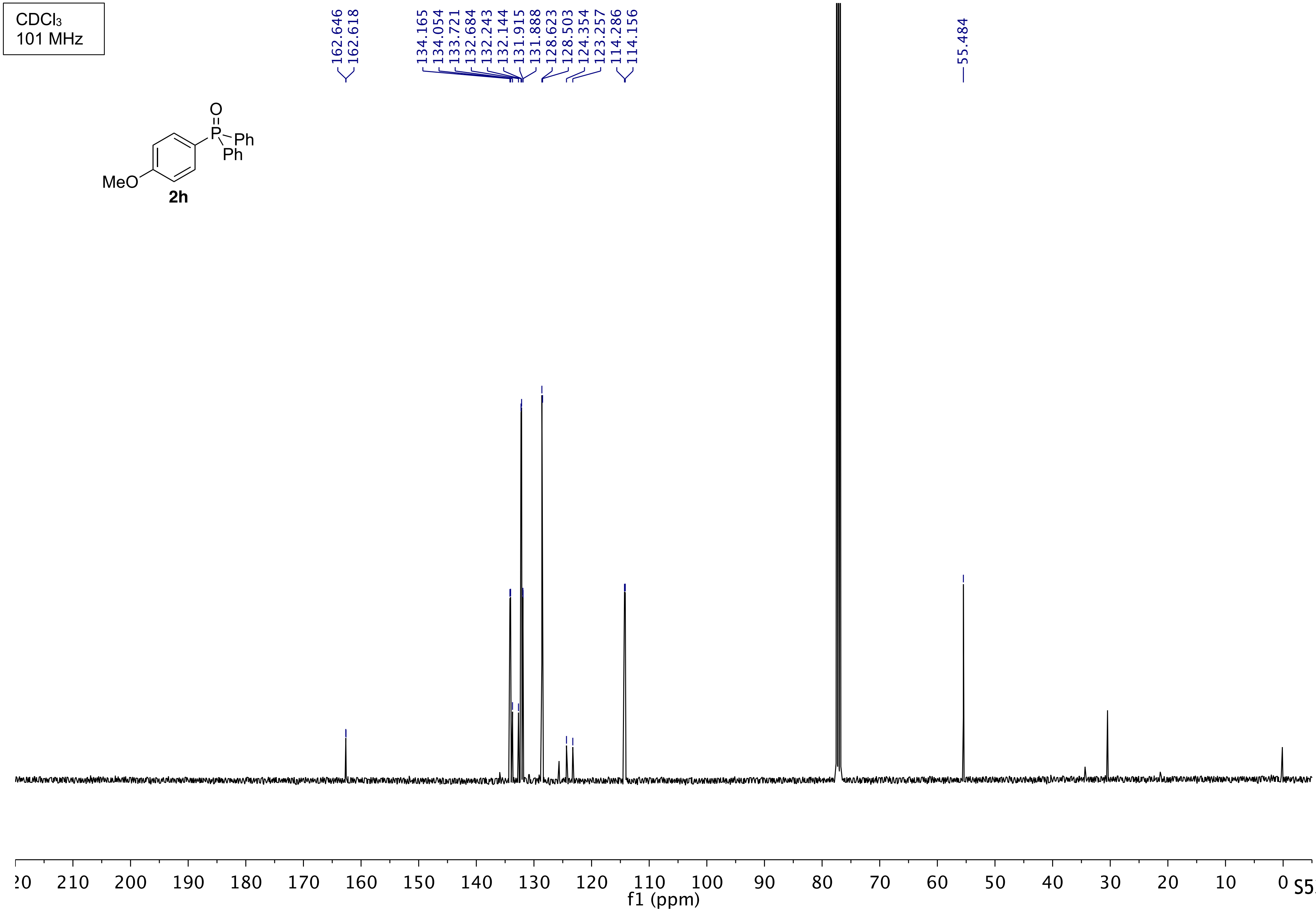

CDCl3
101 MHz
162.646
162.618
134.165
134.054
133.721
132.684
132.243
132.144
131.915
131.888
128.623
128.503
124.354
123.257
114.286
114.156
55.484
O
P
Ph
Ph
MeO
2h
f1 (ppm)

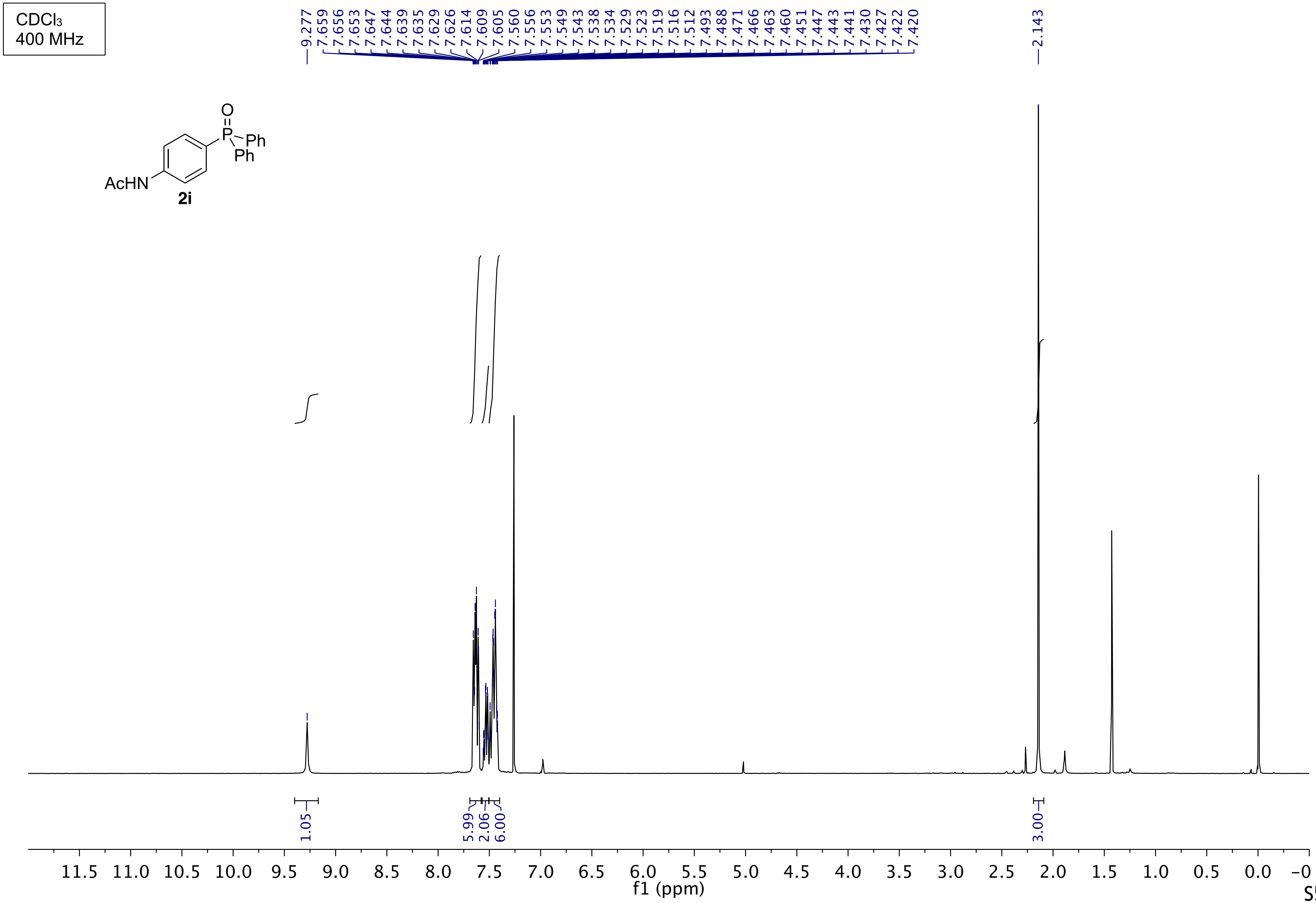
CDCl3
400 MHz
AcHN
P
O
Ph
Ph
2i
9.277
7.659
7.656
7.653
7.647
7.644
7.639
7.635
7.629
7.626
7.614
7.609
7.605
7.560
7.556
7.553
7.549
7.543
7.538
7.534
7.529
7.523
7.519
7.516
7.512
7.493
7.488
7.471
7.466
7.463
7.460
7.451
7.447
7.443
7.441
7.430
7.427
7.422
7.420
2.143
1.05
5.99
2.06
6.00
3.00
11.5 11.0 10.5 10.0 9.5 9.0 8.5 8.0 7.5 7.0 6.5 6.0 5.5 5.0 4.5 4.0 3.5 3.0 2.5 2.0 1.5 1.0 0.5 0.0 –0
f1 (ppm)

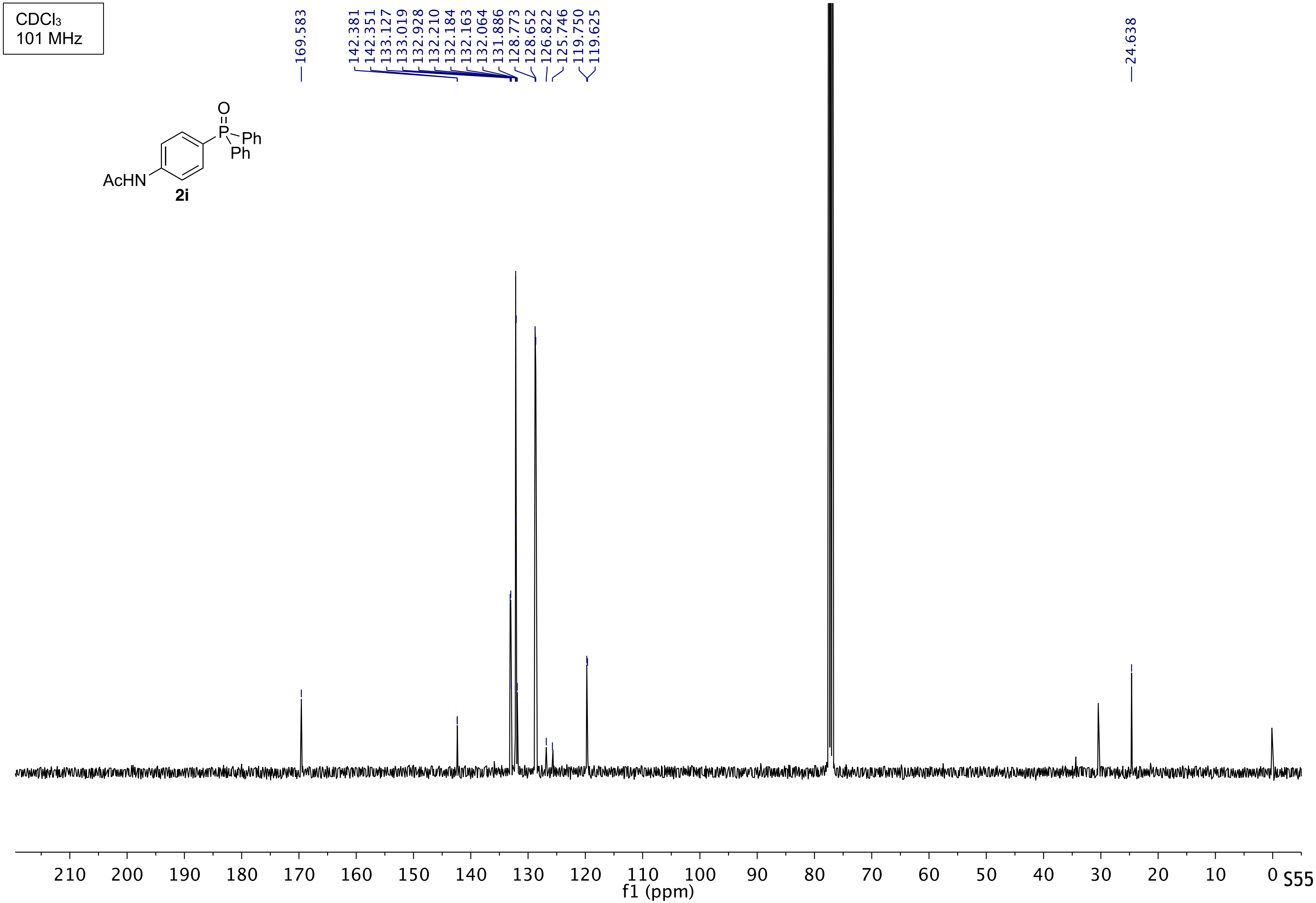
CDCl3
101 MHz
169.583
142.381
142.351
133.127
133.019
132.928
132.210
132.184
132.163
132.064
131.886
128.773
128.652
126.822
125.746
119.750
119.625
24.638
O
P
Ph
Ph
AcHN
2i
210 200 190 180 170 160 150 140 130 120 110 100 90 80 70 60 50 40 30 20 10 0
f1 (ppm)

CDCl3
400 MHz

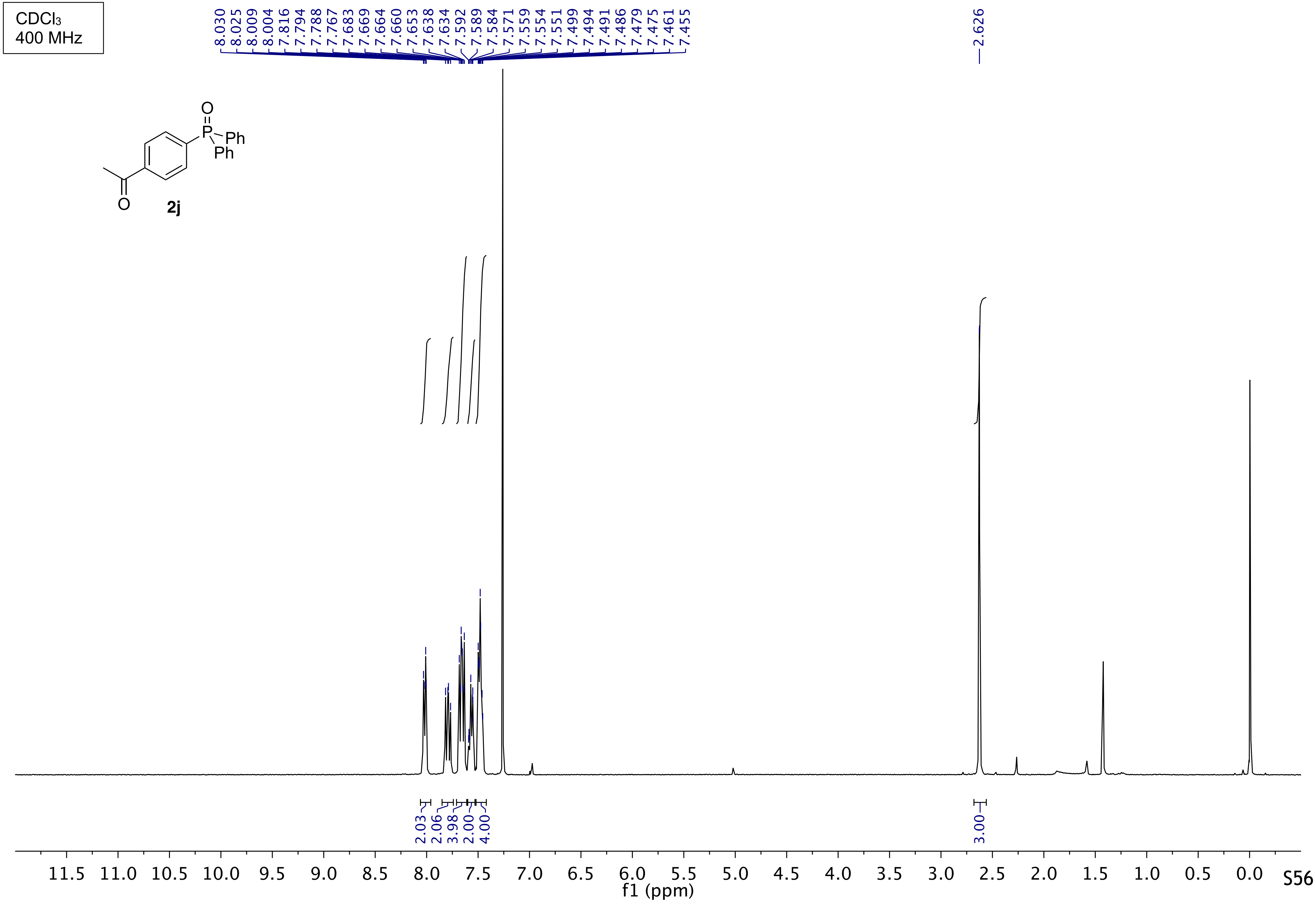

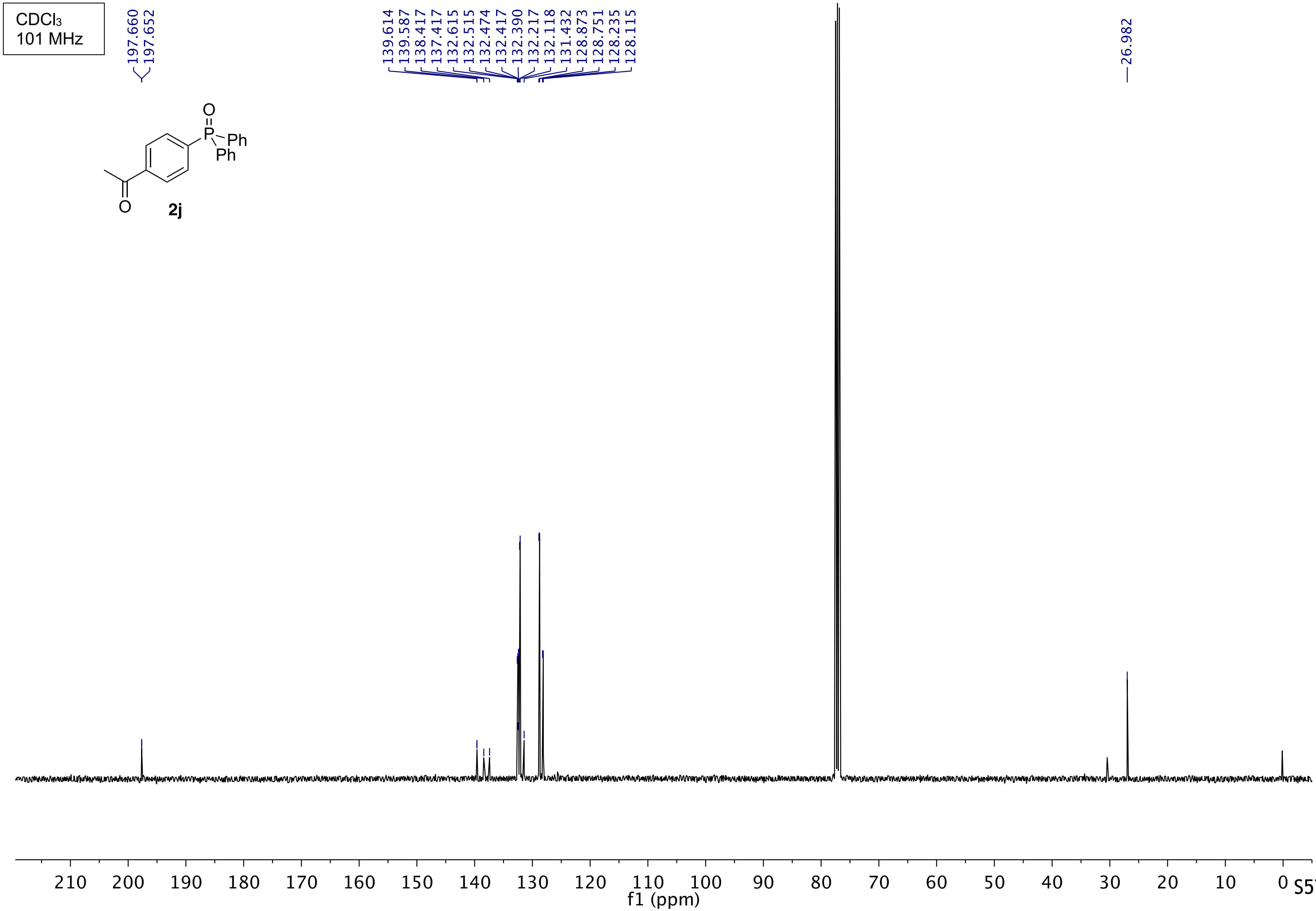
CDCl3
101 MHz
197.660
197.652
139.614
139.587
138.417
137.417
132.615
132.515
132.474
132.417
132.390
132.217
132.118
131.432
128.873
128.751
128.235
128.115
26.982
O
P
Ph
Ph
O
2j
210 200 190 180 170 160 150 140 130 120 110 100 90 80 70 60 50 40 30 20 10 0
f1 (ppm)

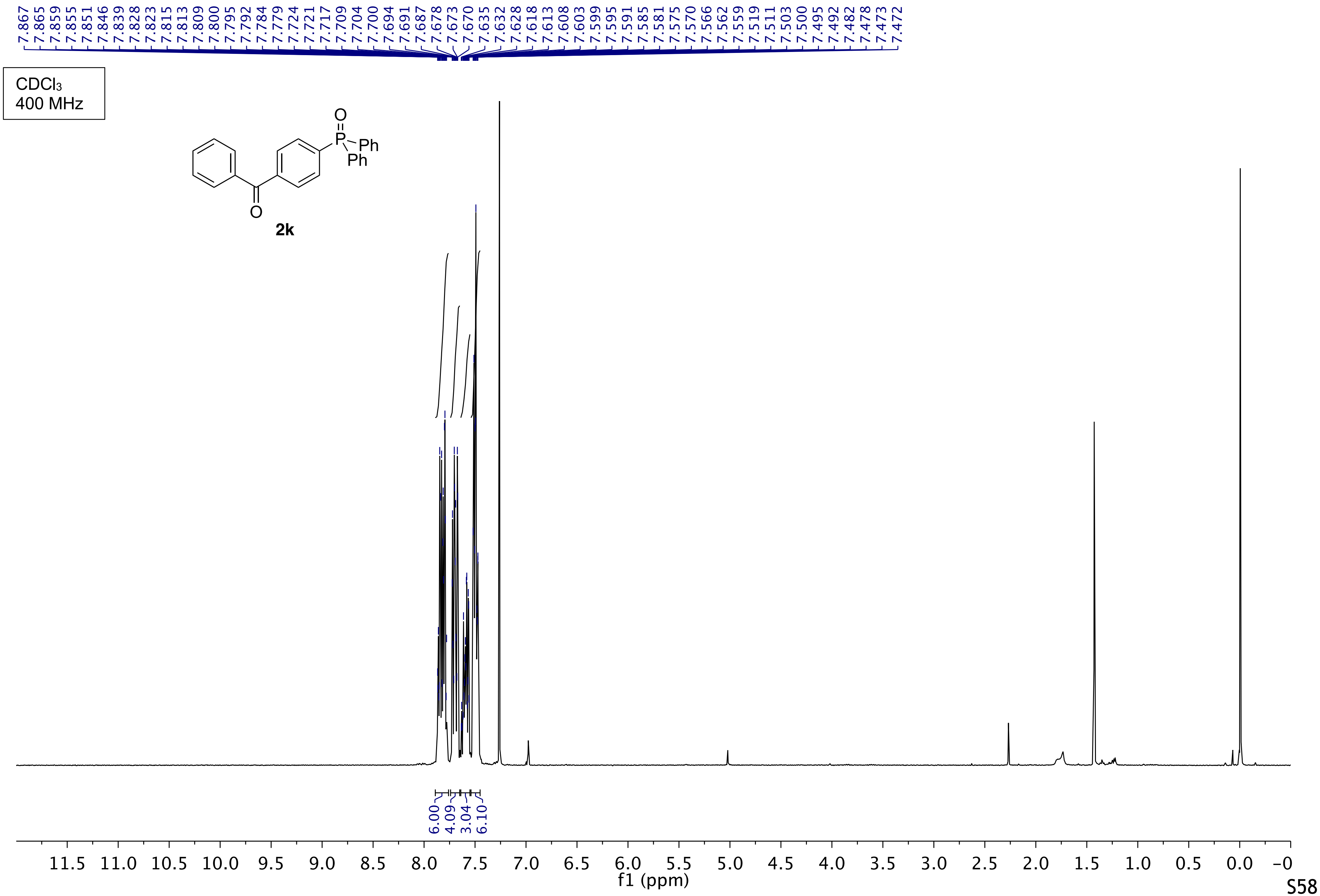
CDCl3
400 MHz
2k
7.867 7.865 7.859 7.855 7.851 7.846 7.839 7.828 7.823 7.815 7.813 7.809 7.800 7.795 7.792 7.784 7.779 7.724 7.721 7.717 7.709 7.704 7.700 7.694 7.691 7.687 7.678 7.673 7.670 7.635 7.632 7.628 7.618 7.613 7.608 7.603 7.599 7.595 7.591 7.585 7.581 7.575 7.570 7.566 7.562 7.559 7.519 7.511 7.503 7.500 7.495 7.492 7.482 7.478 7.473 7.472
6.00
4.09
3.04
6.10
f1 (ppm)

CDCl3
101 MHz

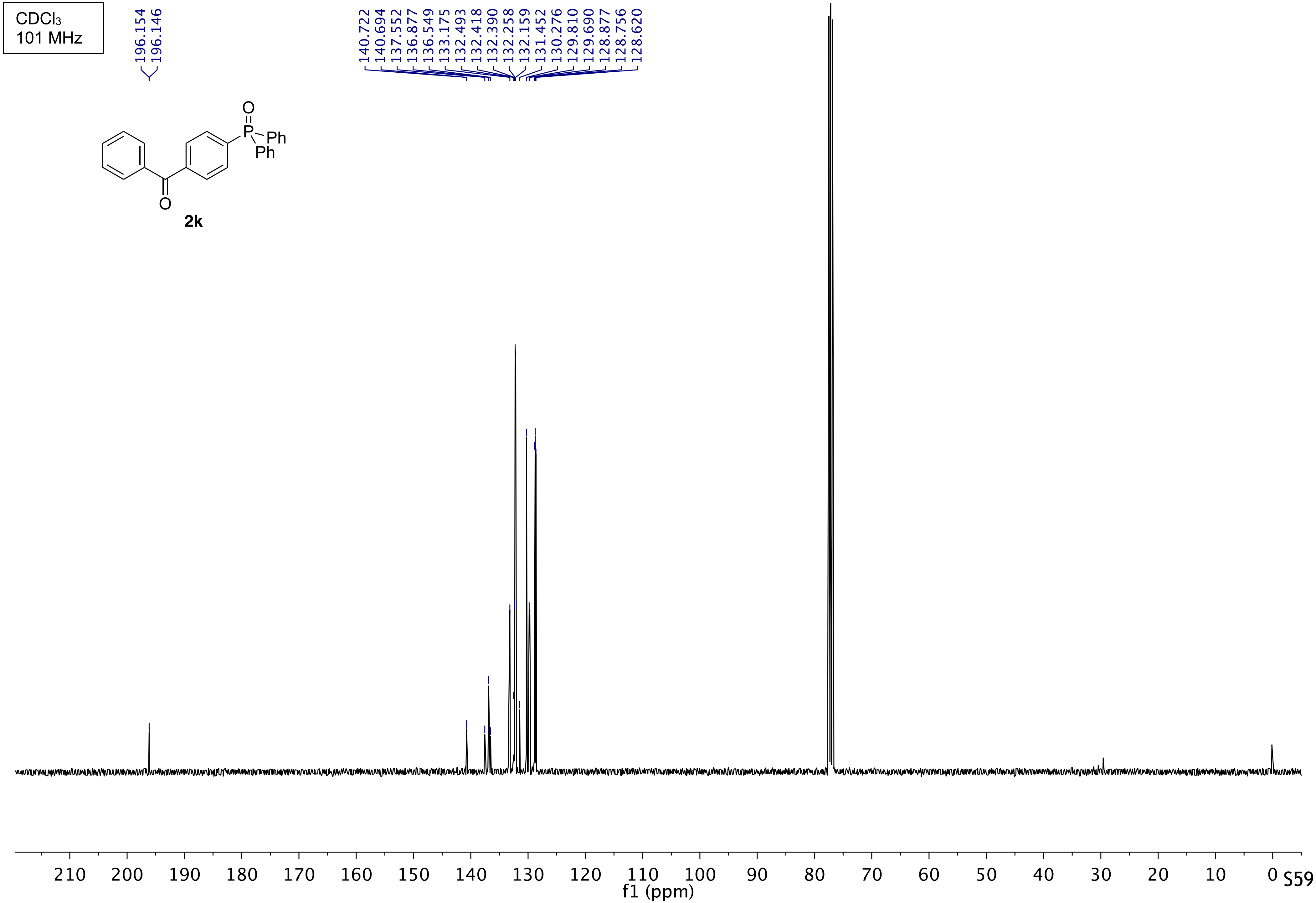

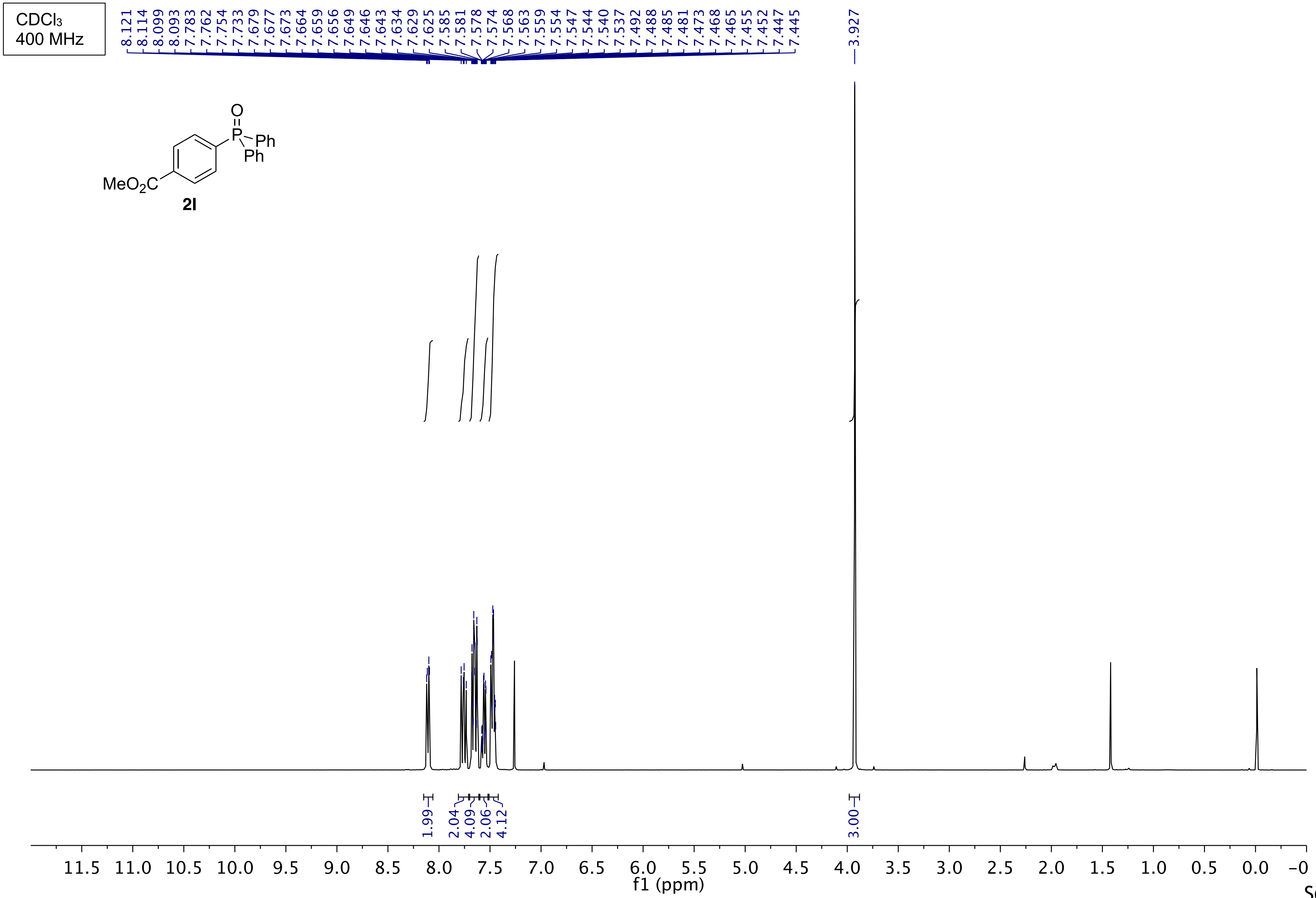

CDCl3
400 MHz
8.121
8.114
8.099
8.093
7.783
7.762
7.754
7.733
7.679
7.677
7.673
7.664
7.659
7.656
7.649
7.646
7.643
7.634
7.629
7.625
7.585
7.581
7.578
7.574
7.568
7.563
7.559
7.554
7.547
7.544
7.540
7.537
7.492
7.488
7.485
7.481
7.473
7.468
7.465
7.455
7.452
7.447
7.445
3.927
O
P
Ph
Ph
MeO2C
2l
1.99
2.04
4.09
2.06
4.12
3.00
11.5 11.0 10.5 10.0 9.5 9.0 8.5 8.0 7.5 7.0 6.5 6.0 5.5 5.0 4.5 4.0 3.5 3.0 2.5 2.0 1.5 1.0 0.5 0.0 −0
f1 (ppm)

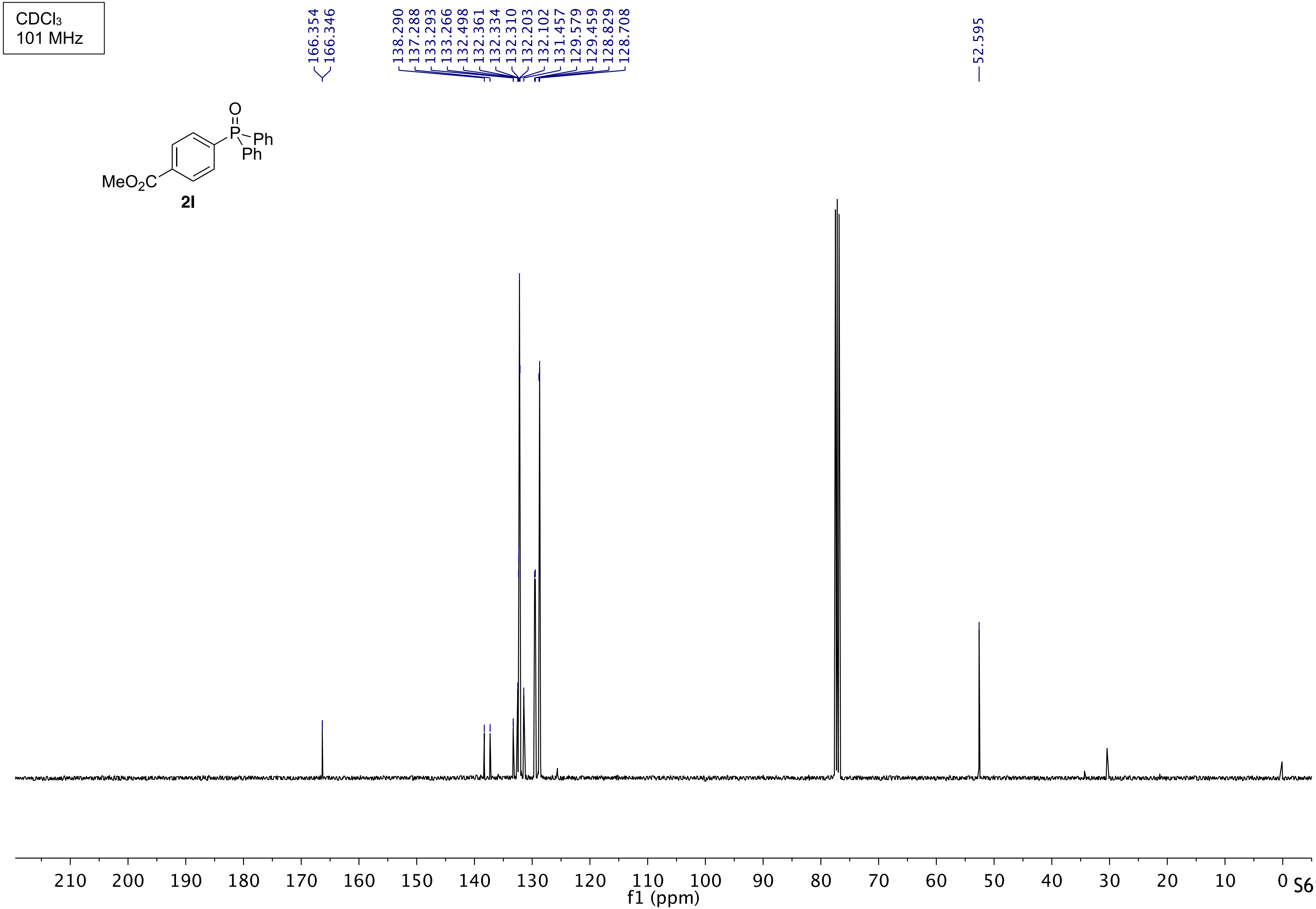
CDCl3
101 MHz
166.354
166.346
138.290
137.288
133.293
133.266
132.498
132.361
132.334
132.310
132.203
132.102
131.457
129.579
129.459
128.829
128.708
52.595
O
P
Ph
Ph
MeO2C
2l
210
200
190
180
170
160
150
140
130
120
110
100
90
80
70
60
50
40
30
20
10
0
f1 (ppm)

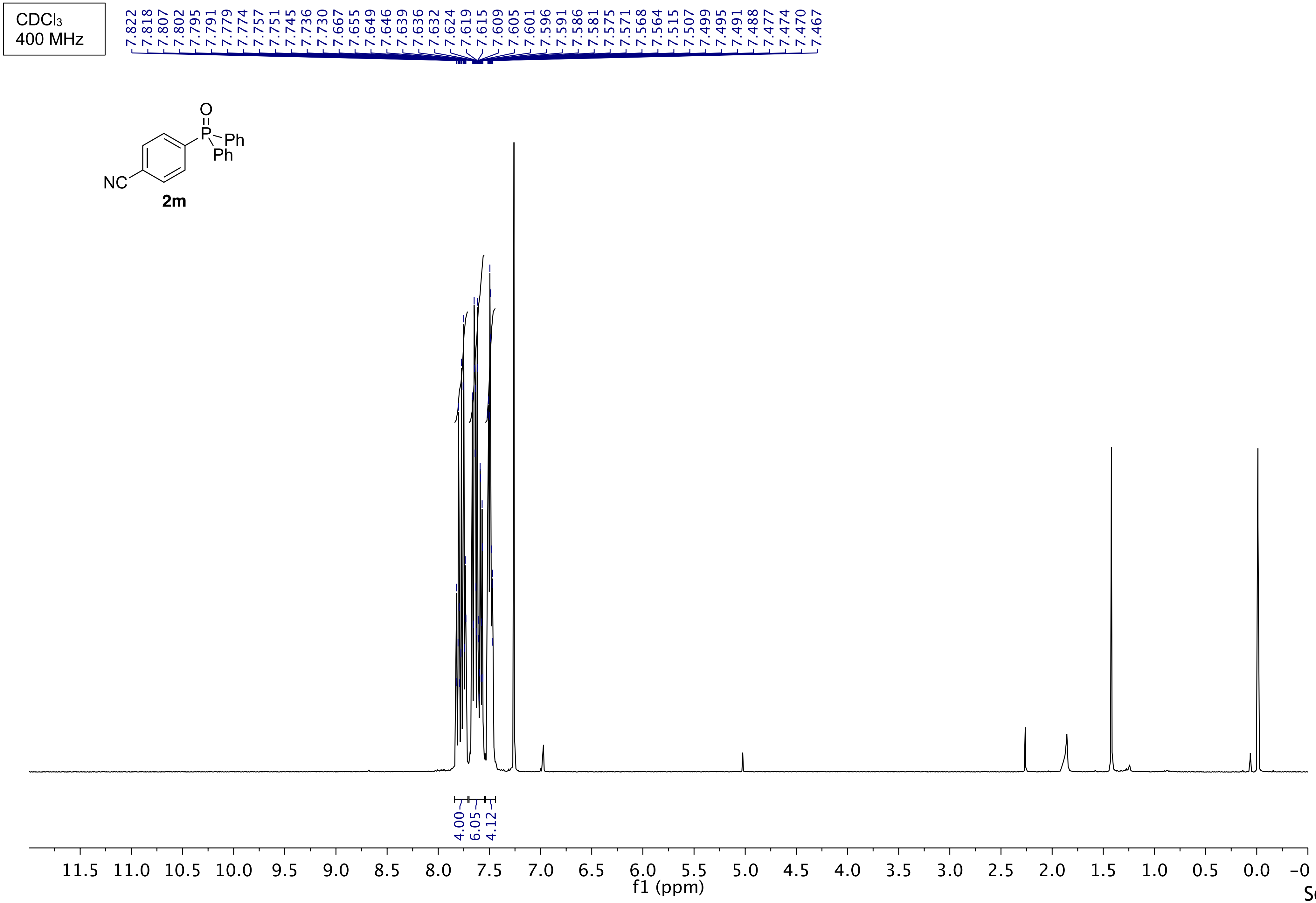
CDCl3
400 MHz
7.822
7.818
7.807
7.802
7.795
7.791
7.779
7.774
7.757
7.751
7.745
7.736
7.730
7.667
7.655
7.649
7.646
7.639
7.636
7.632
7.624
7.619
7.615
7.609
7.605
7.601
7.596
7.591
7.586
7.581
7.575
7.571
7.568
7.564
7.515
7.507
7.499
7.495
7.491
7.488
7.477
7.474
7.470
7.467
O
P
Ph
Ph
NC
2m
4.00
6.05
4.12
11.5 11.0 10.5 10.0 9.5 9.0 8.5 8.0 7.5 7.0 6.5 6.0 5.5 5.0 4.5 4.0 3.5 3.0 2.5 2.0 1.5 1.0 0.5 0.0 –0
f1 (ppm)

CDCl$_3$
101 MHz

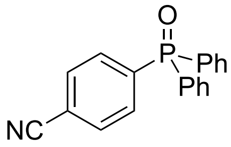

2m

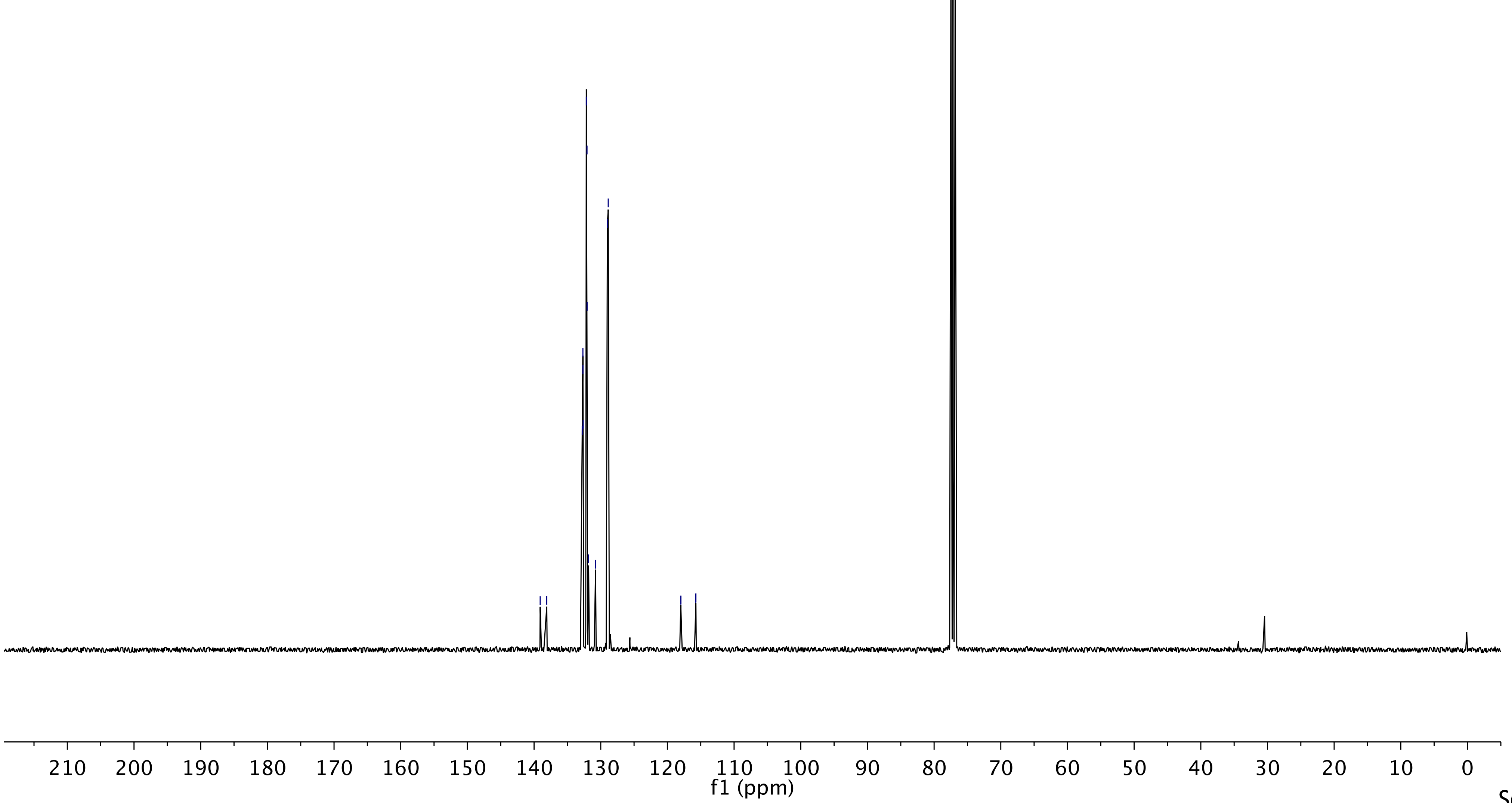

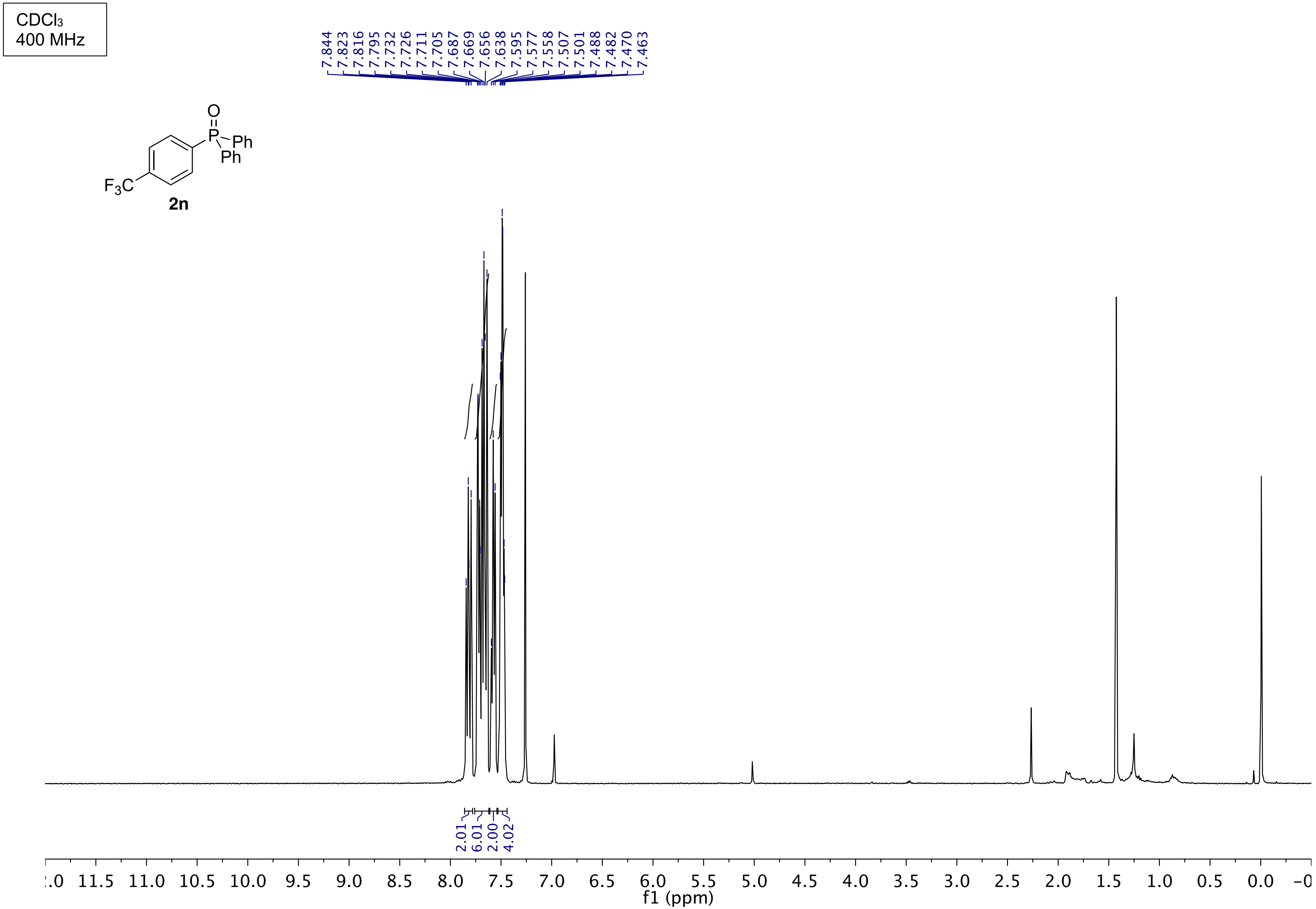
CDCl3
400 MHz
7.844
7.823
7.816
7.795
7.732
7.726
7.711
7.705
7.687
7.669
7.656
7.638
7.595
7.577
7.558
7.507
7.501
7.488
7.482
7.470
7.463
O
P
Ph
Ph
F3C
2n
2.01
6.01
2.00
4.02
12.0 11.5 11.0 10.5 10.0 9.5 9.0 8.5 8.0 7.5 7.0 6.5 6.0 5.5 5.0 4.5 4.0 3.5 3.0 2.5 2.0 1.5 1.0 0.5 0.0 -0
f1 (ppm)

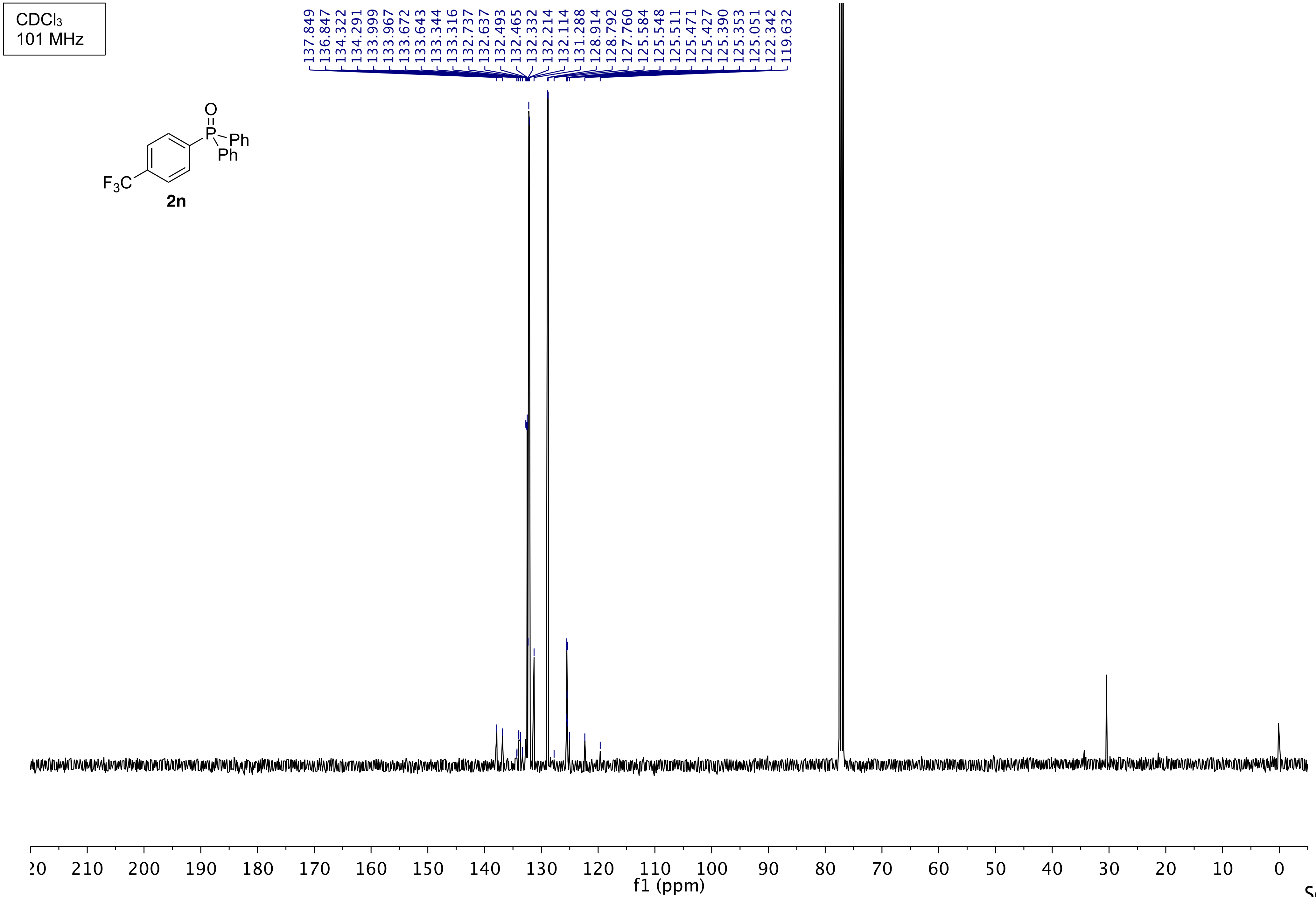
CDCl3
101 MHz
137.849
136.847
134.322
134.291
133.999
133.967
133.672
133.643
133.344
133.316
132.737
132.637
132.493
132.465
132.332
132.214
132.114
131.288
128.914
128.792
127.760
125.584
125.548
125.511
125.471
125.427
125.390
125.353
125.051
122.342
119.632
O
P
Ph
Ph
F3C
2n
210 200 190 180 170 160 150 140 130 120 110 100 90 80 70 60 50 40 30 20 10 0
f1 (ppm)

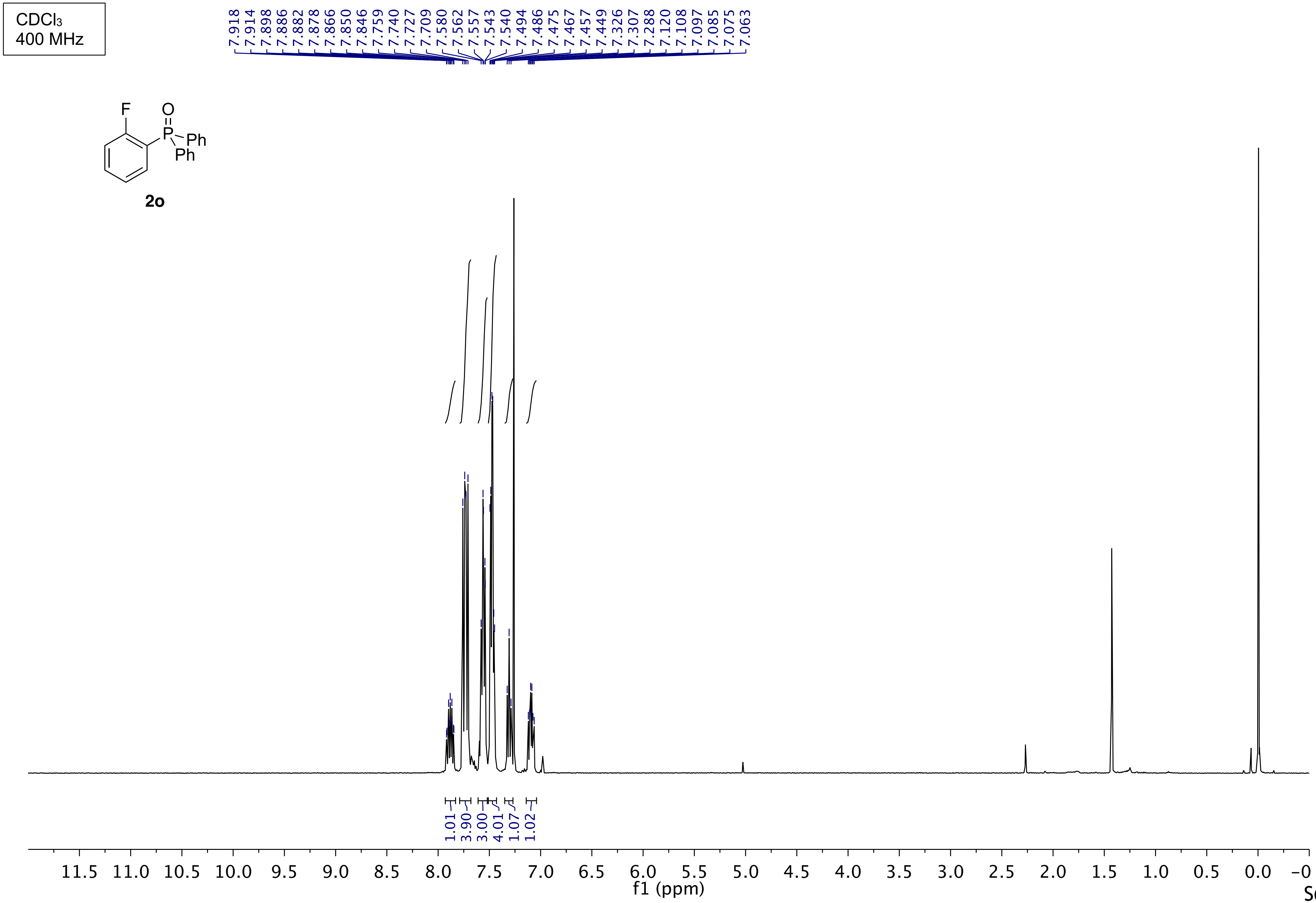
CDCl3
400 MHz
F
O
P
Ph
Ph
2o
7.918
7.914
7.898
7.886
7.882
7.878
7.866
7.850
7.846
7.759
7.740
7.727
7.709
7.580
7.562
7.557
7.543
7.540
7.494
7.486
7.475
7.467
7.457
7.449
7.326
7.307
7.288
7.120
7.108
7.097
7.085
7.075
7.063
1.01
3.90
3.00
4.01
1.07
1.02
11.5 11.0 10.5 10.0 9.5 9.0 8.5 8.0 7.5 7.0 6.5 6.0 5.5 5.0 4.5 4.0 3.5 3.0 2.5 2.0 1.5 1.0 0.5 0.0 –0
f1 (ppm)

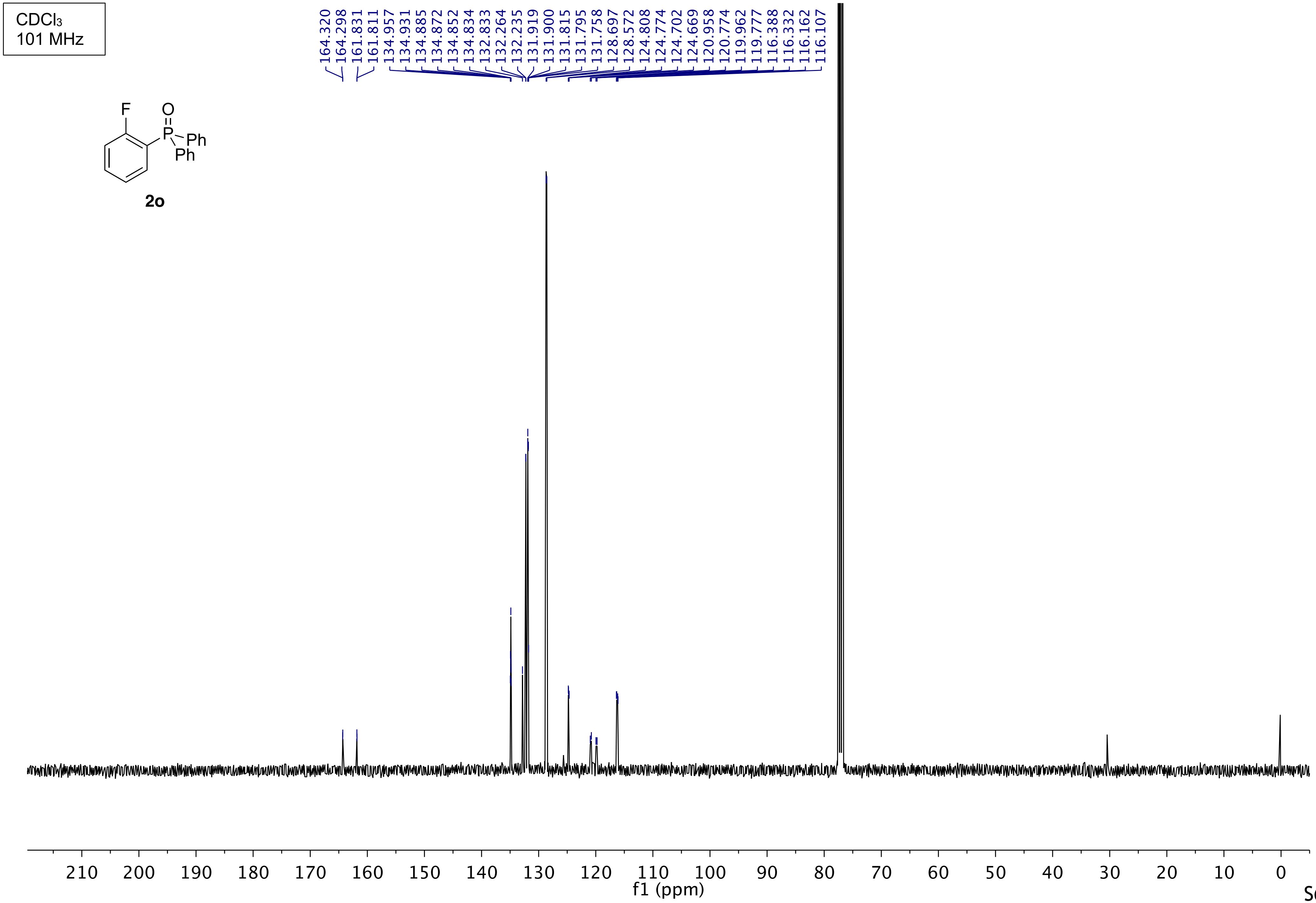
CDCl3
101 MHz
2o
164.320
164.298
161.831
161.811
134.957
134.931
134.885
134.872
134.852
134.834
132.833
132.264
132.235
131.919
131.900
131.815
131.795
131.758
128.697
128.572
124.808
124.774
124.702
124.669
120.958
120.774
119.962
119.777
116.388
116.332
116.162
116.107
210 200 190 180 170 160 150 140 130 120 110 100 90 80 70 60 50 40 30 20 10 0
f1 (ppm)

CDCl$_3$
400 MHz

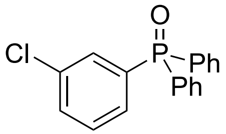

2p

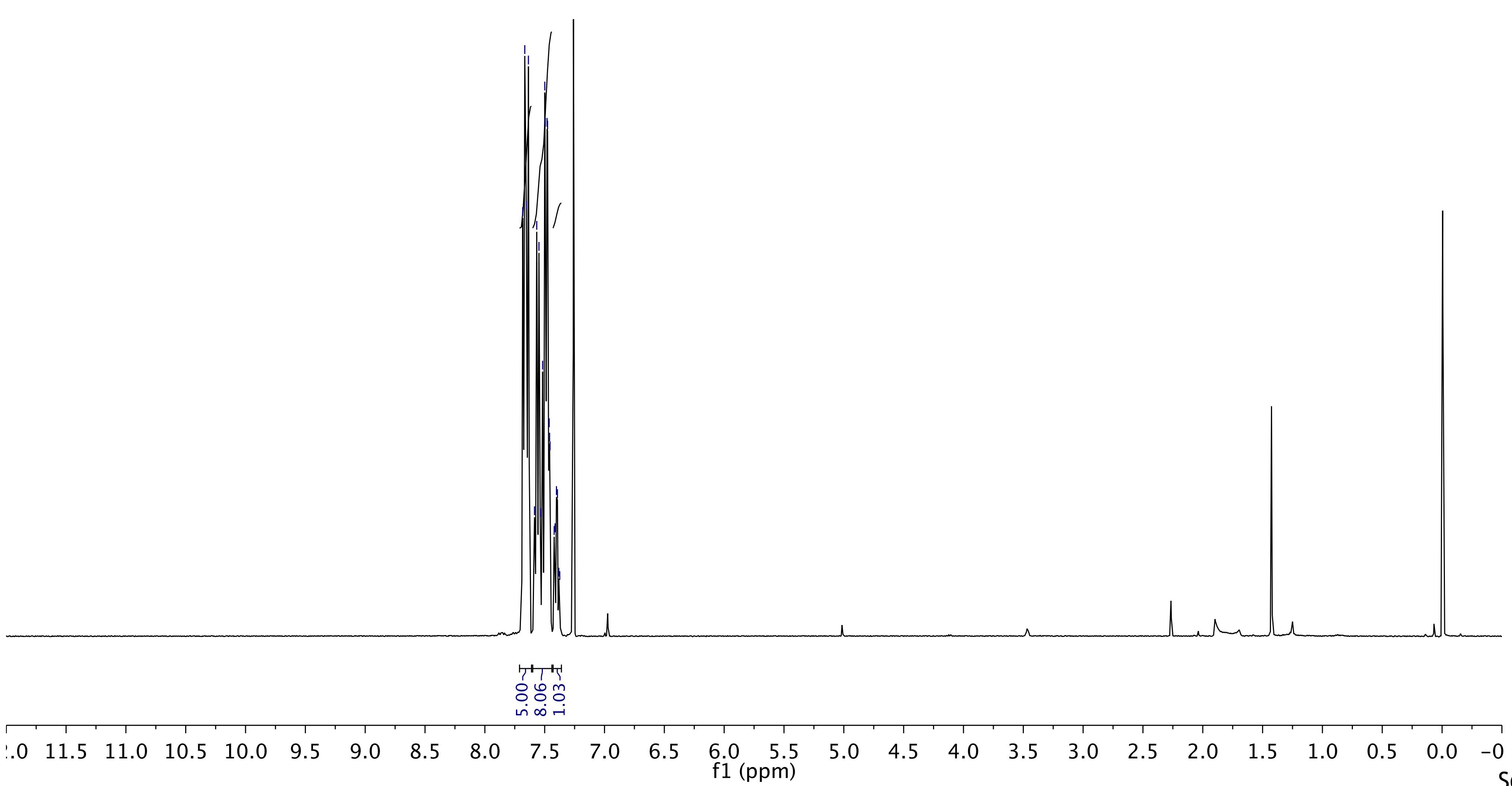

CDCl3
101 MHz

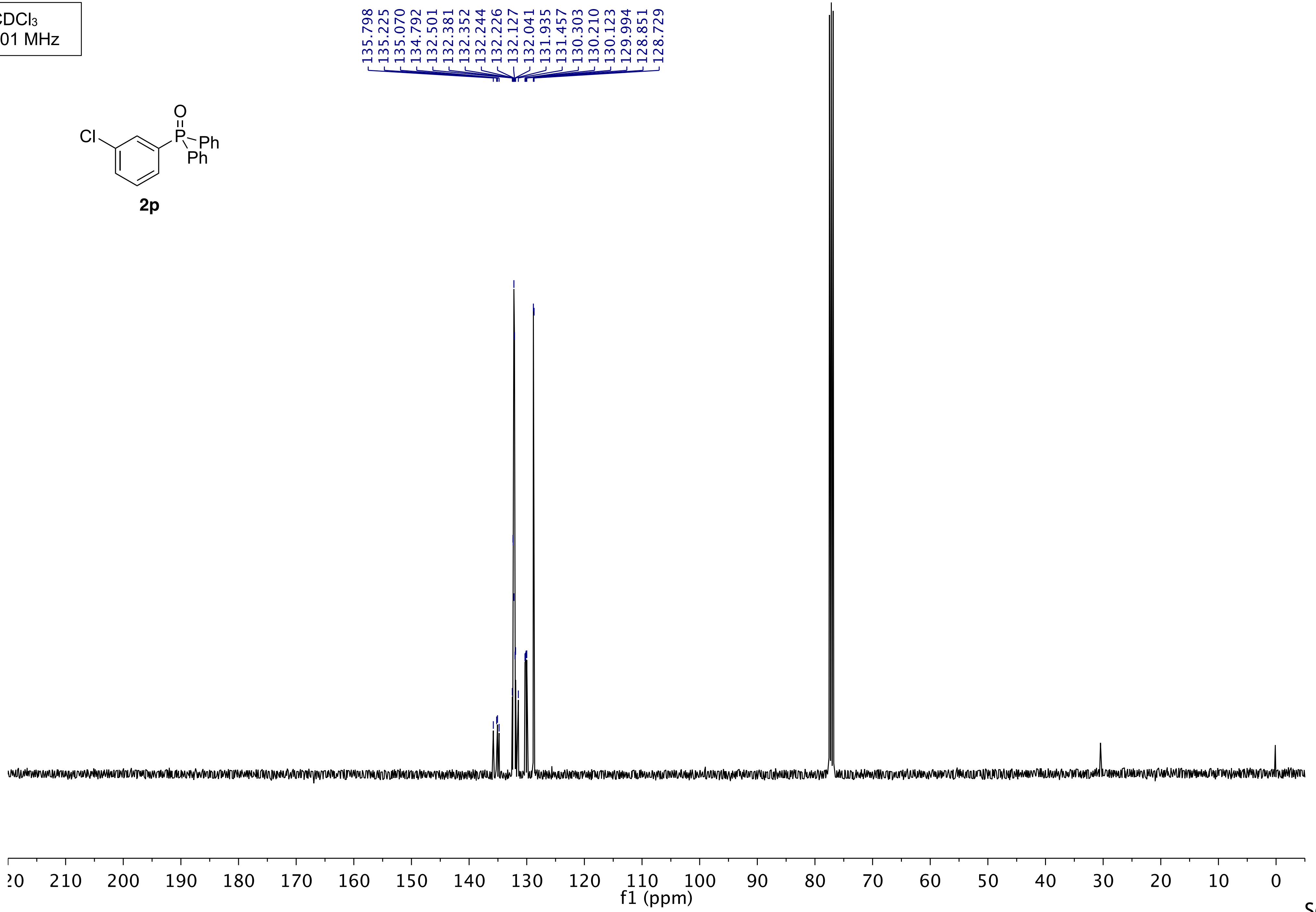

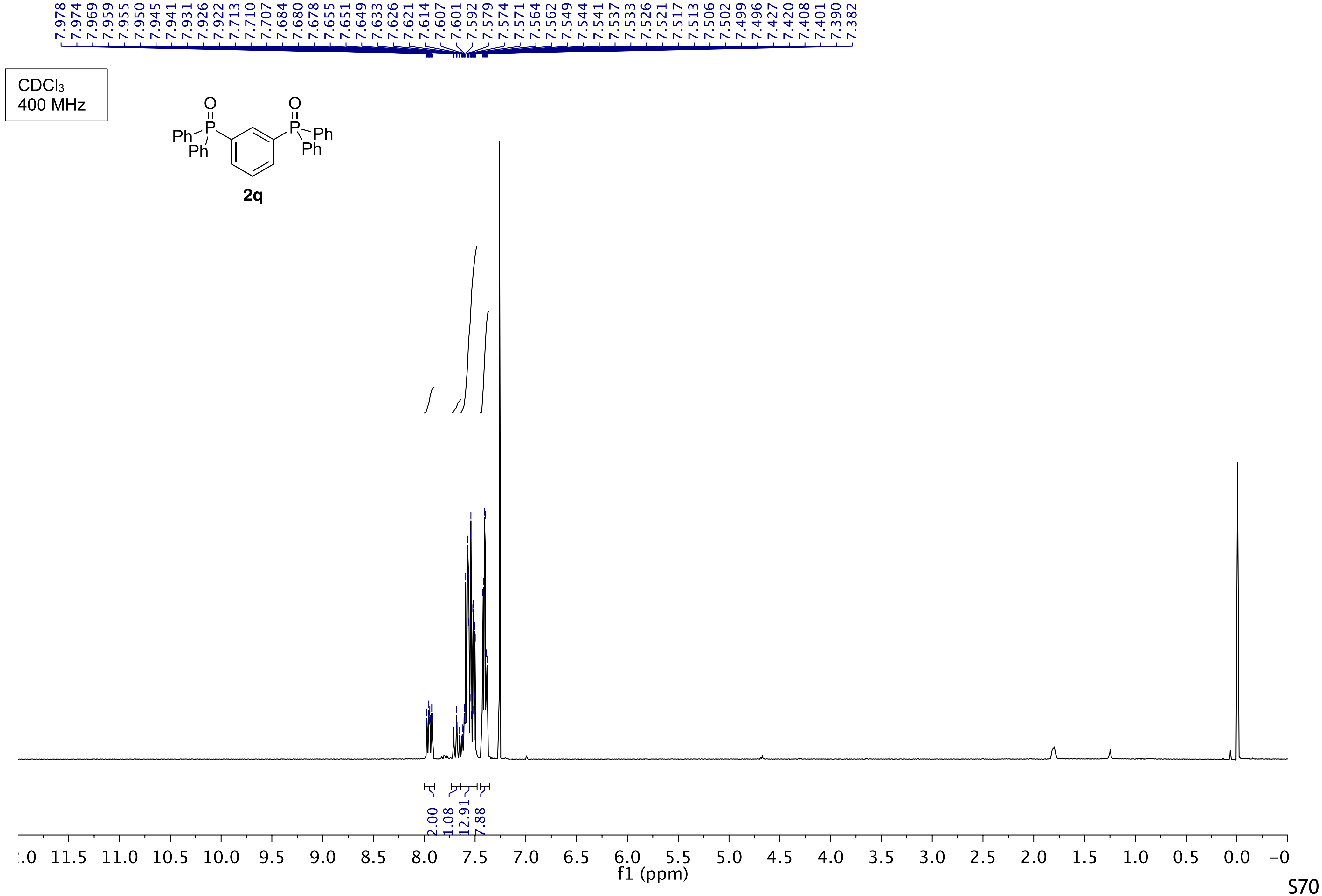
7.978 7.974 7.969 7.959 7.955 7.950 7.945 7.941 7.931 7.926 7.922 7.713 7.710 7.707 7.684 7.680 7.678 7.655 7.651 7.649 7.633 7.626 7.621 7.614 7.607 7.601 7.592 7.579 7.574 7.571 7.564 7.562 7.549 7.544 7.541 7.537 7.533 7.526 7.521 7.517 7.513 7.506 7.502 7.499 7.496 7.427 7.420 7.408 7.401 7.390 7.382
CDCl3
400 MHz
O
P
Ph
Ph
2q
2.00
1.08
12.91
7.88
f1 (ppm)

CDCl$_3$
101 MHz

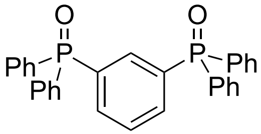

2q

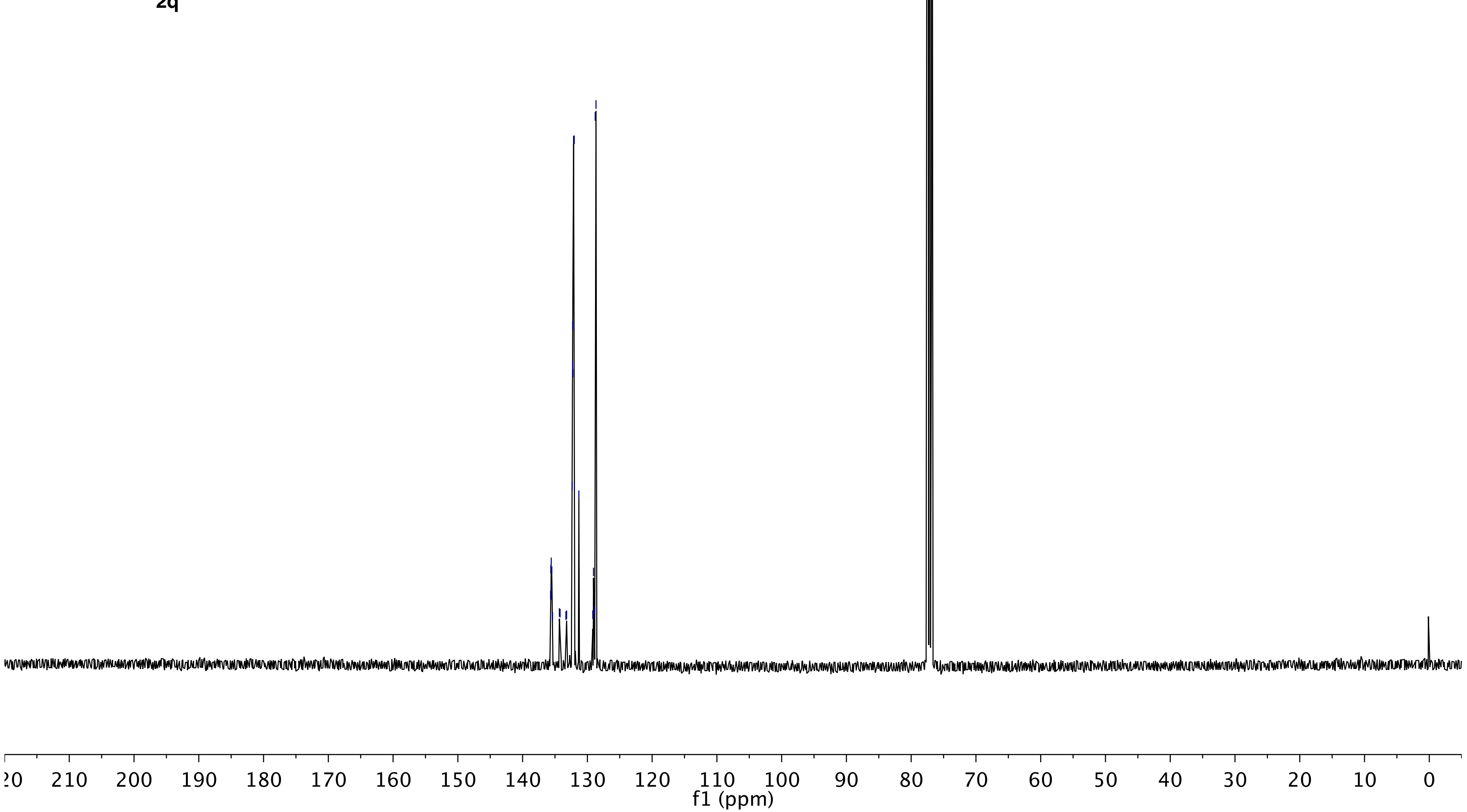

CDCl3
400 MHz

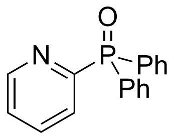

2r

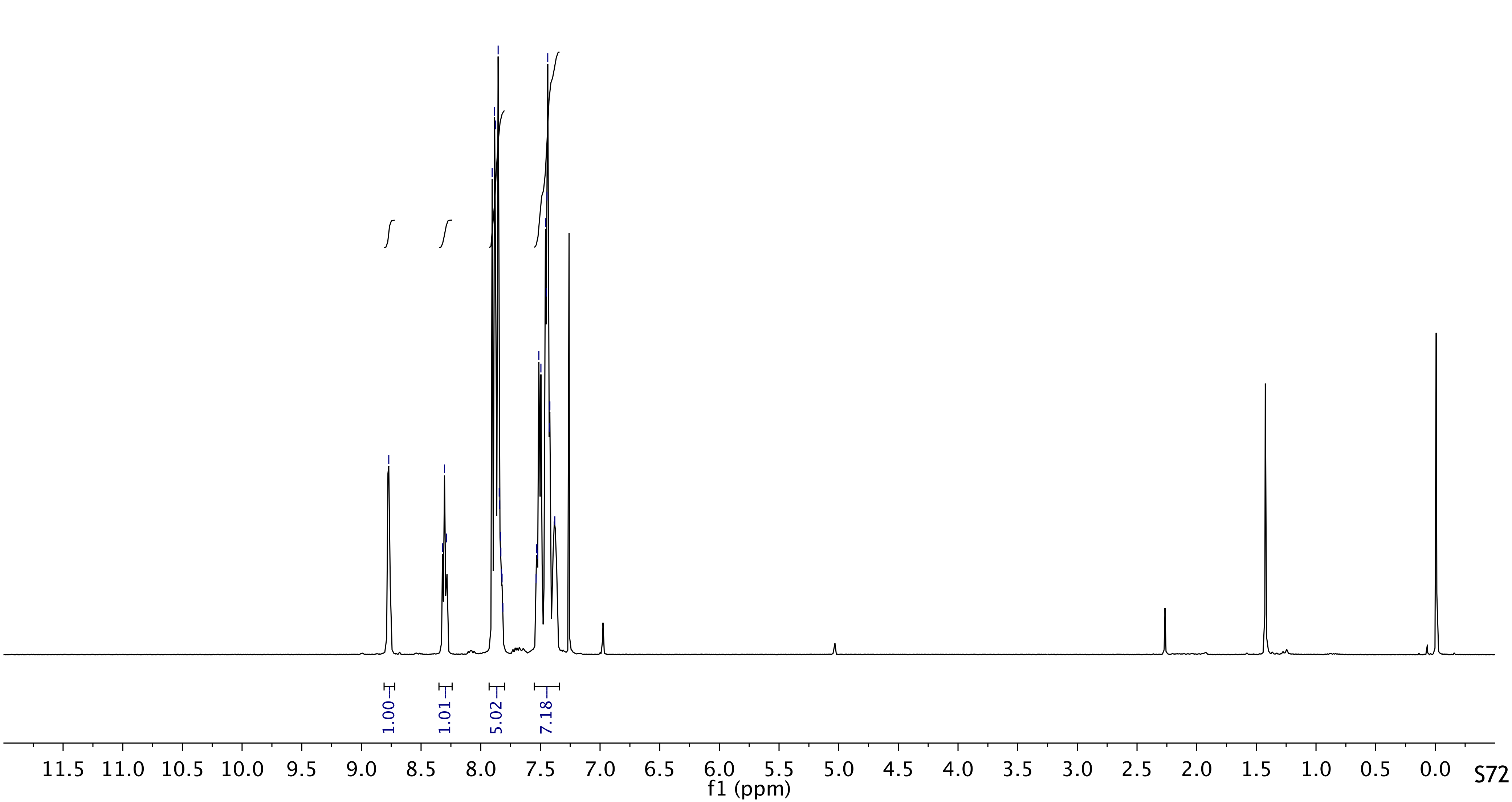

CDCl3
101 MHz

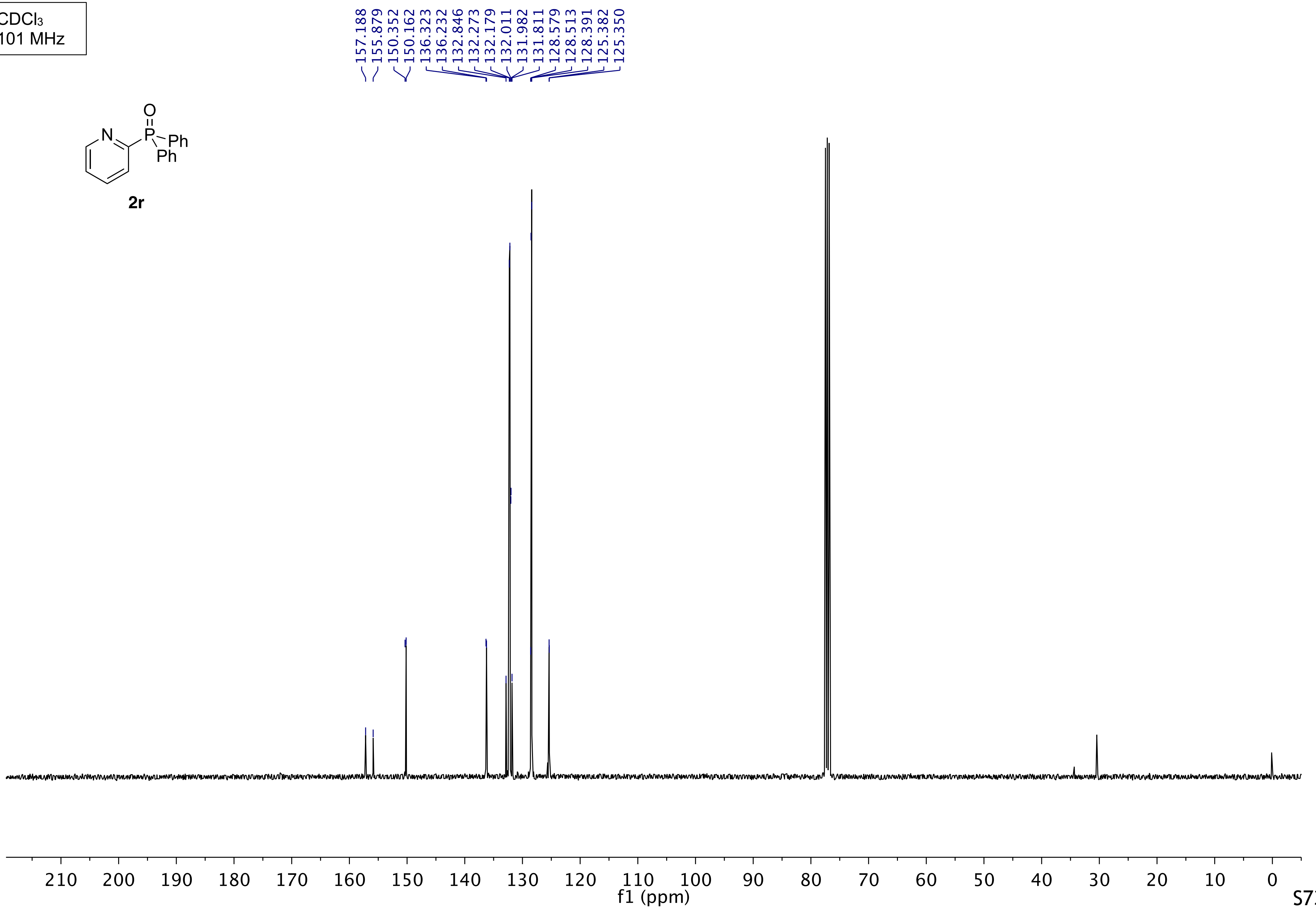

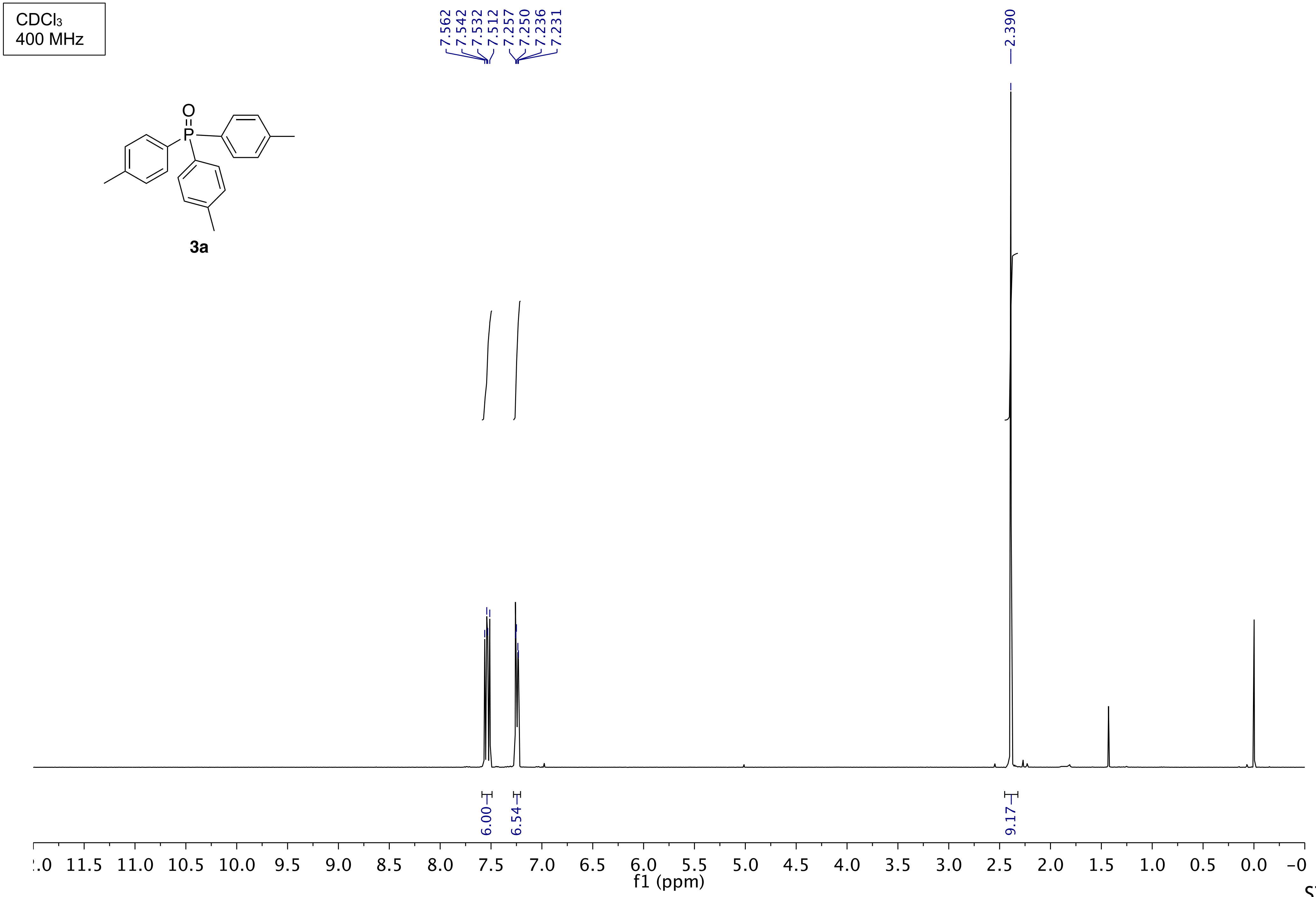
CDCl3
400 MHz
3a
7.562
7.542
7.532
7.512
7.257
7.250
7.236
7.231
2.390
6.00
6.54
9.17
f1 (ppm)

$CDCl_3$
101 MHz

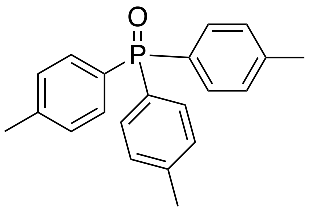

3a

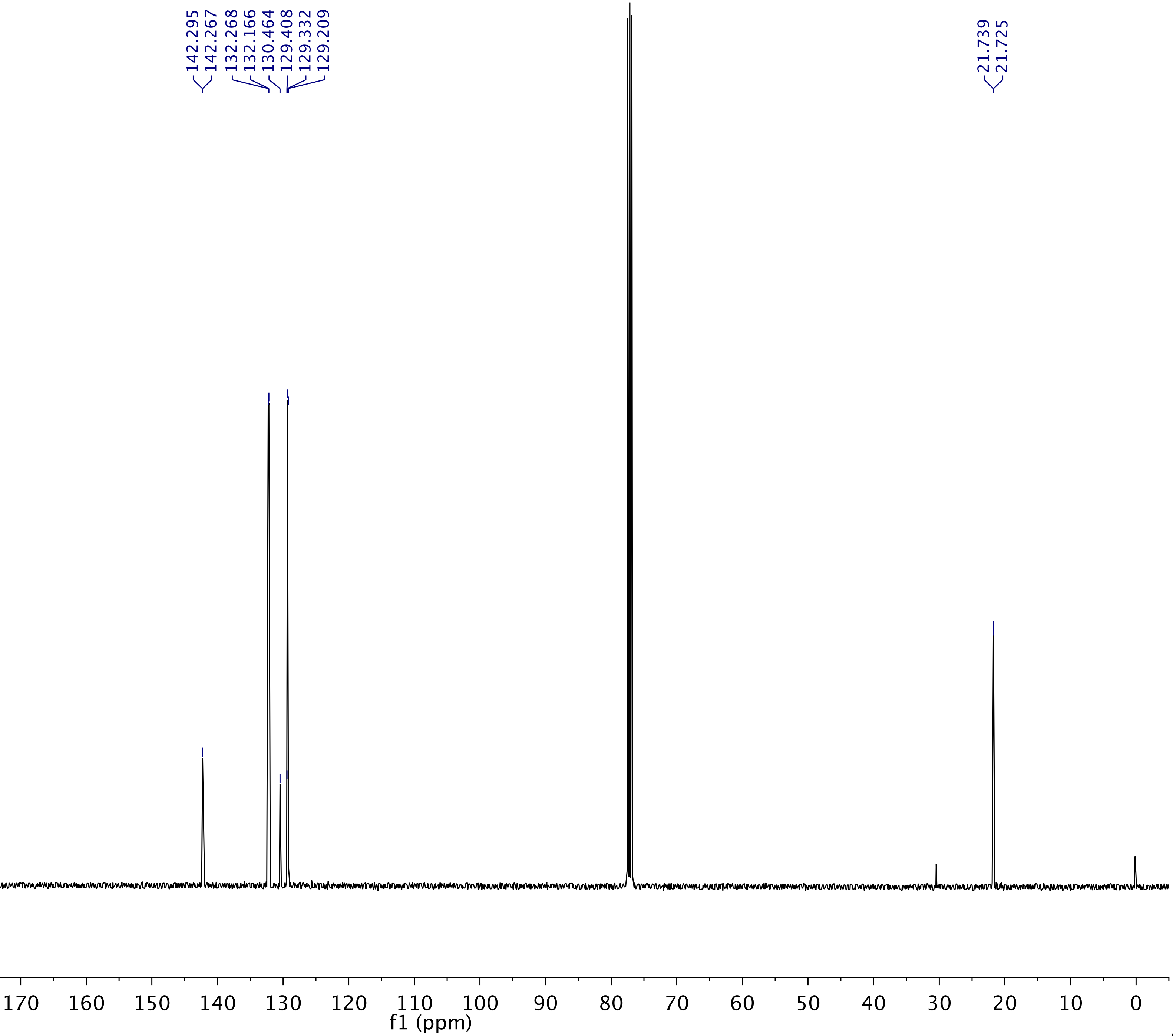

CDCl3
400 MHz

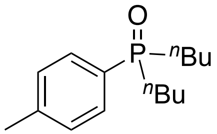

3b

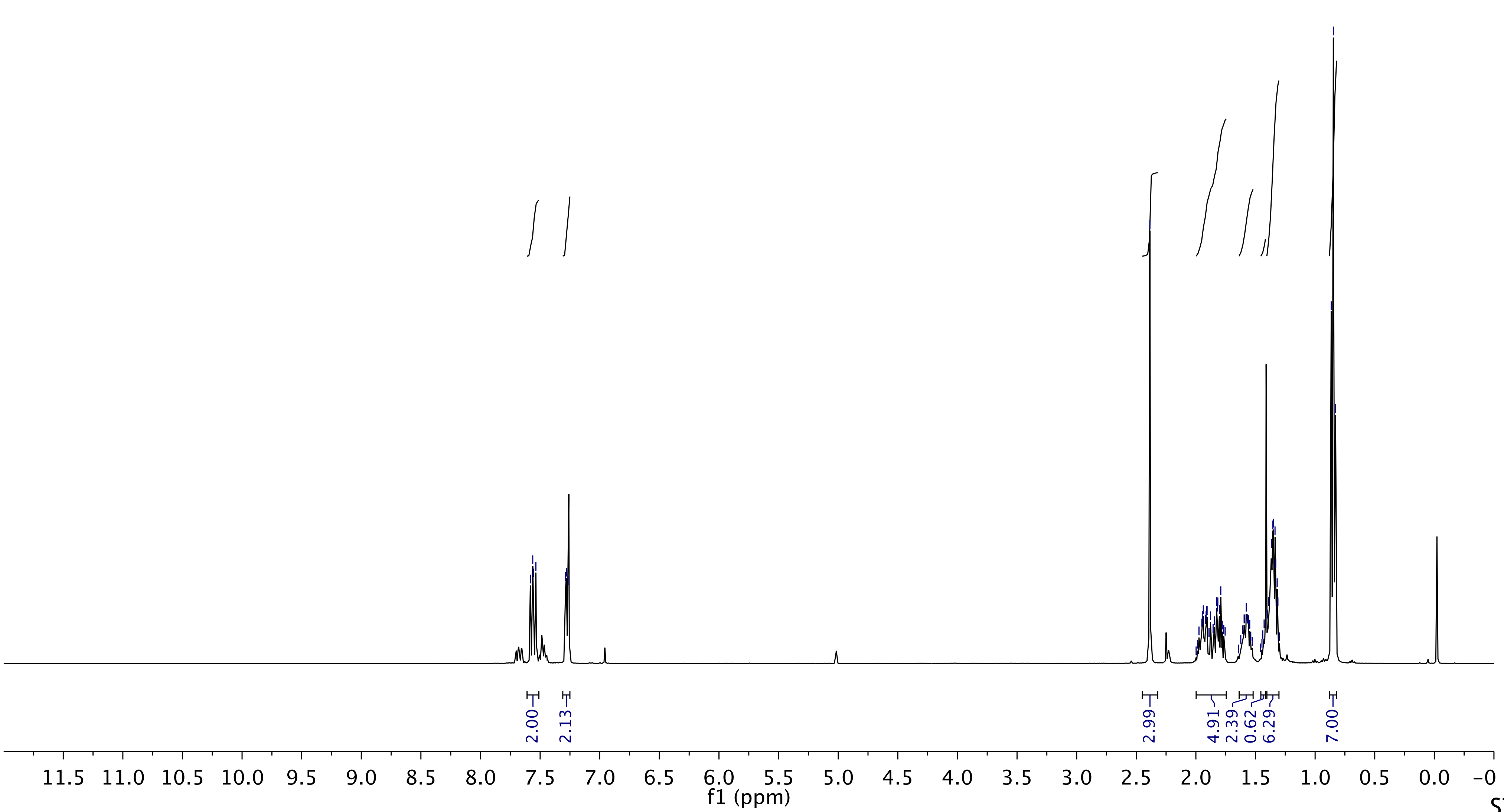

CDCl3
101 MHz

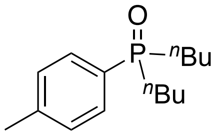

3b

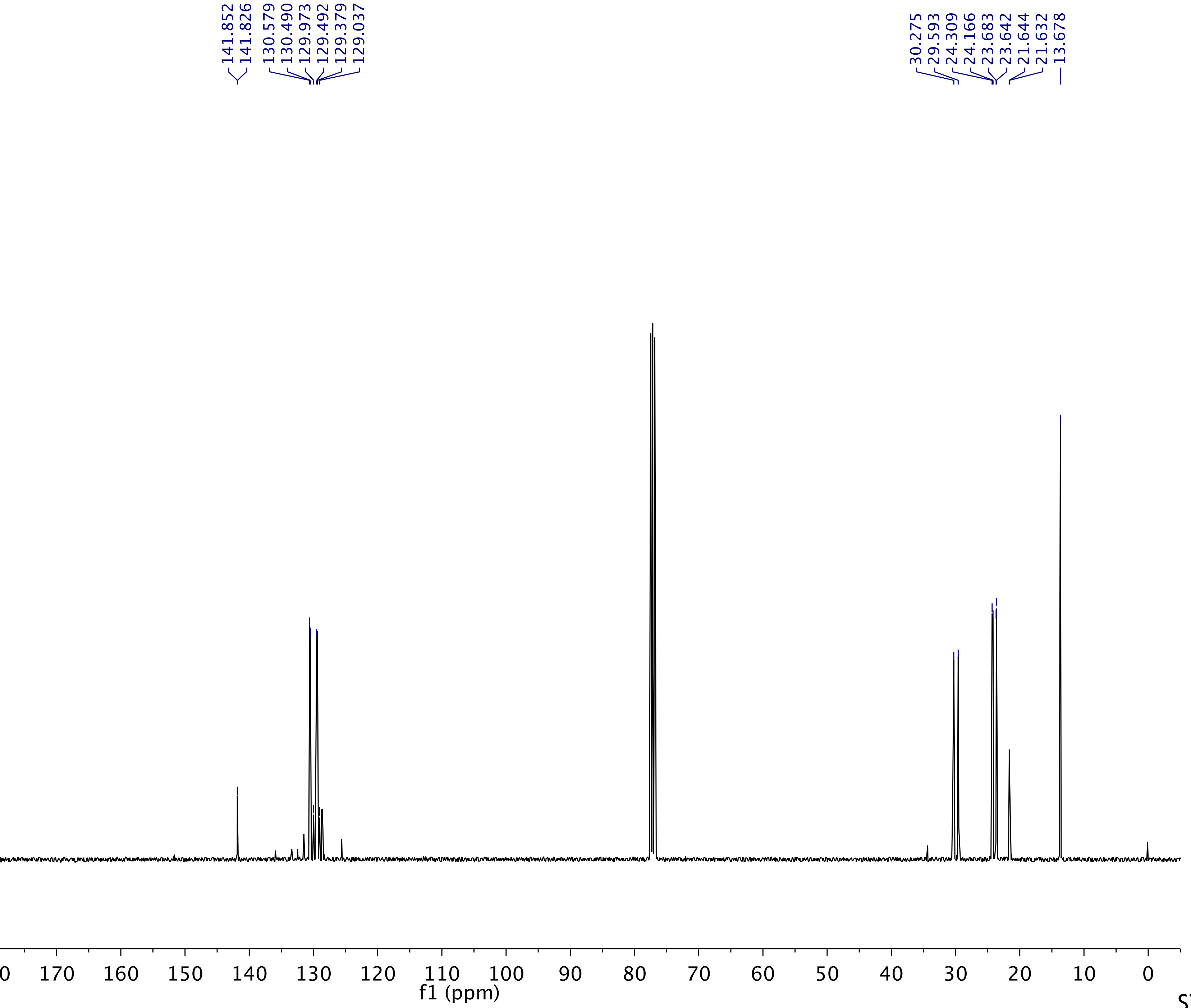

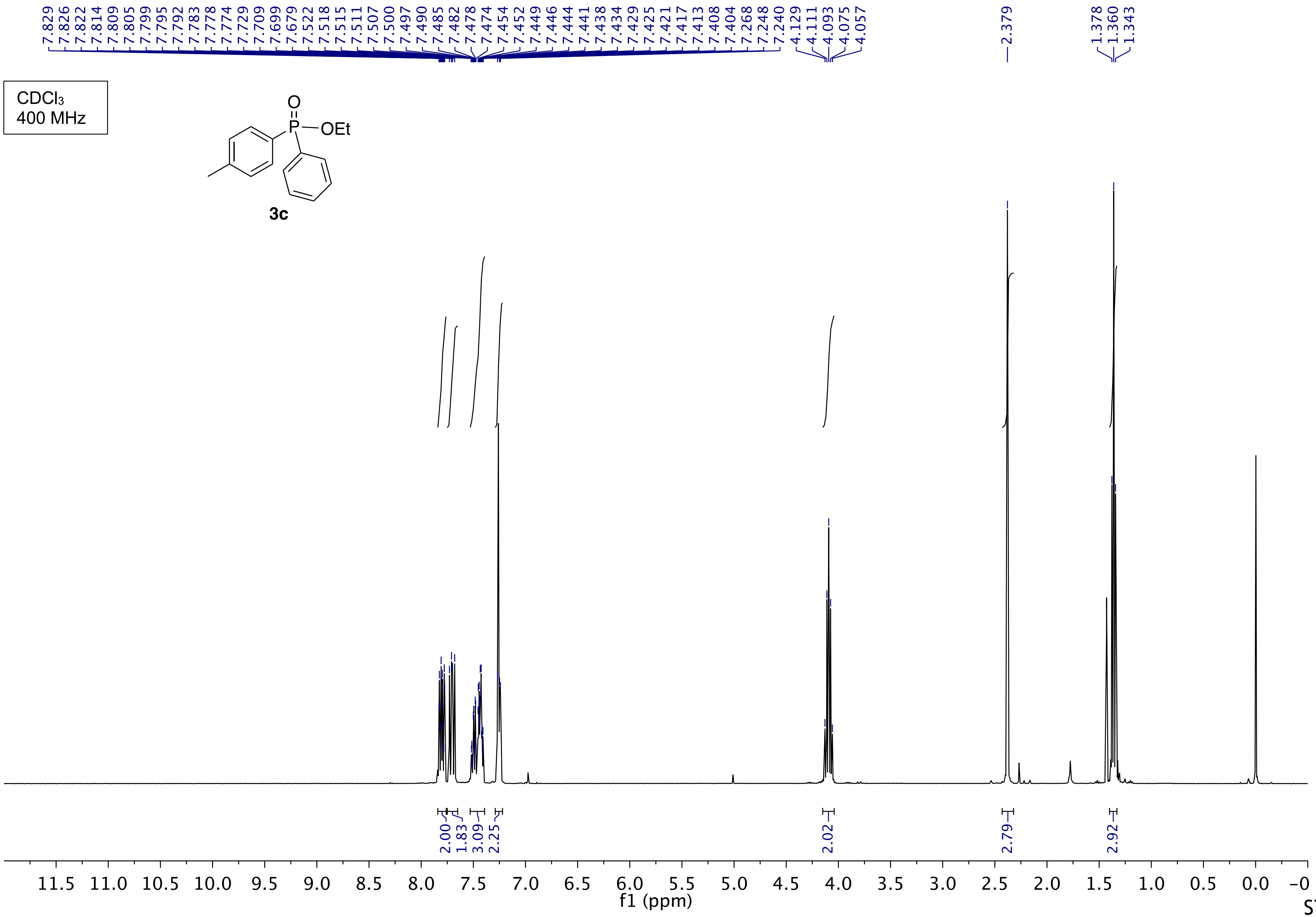

7.829 7.826 7.822 7.814 7.809 7.805 7.799 7.795 7.792 7.783 7.778 7.774 7.729 7.709 7.699 7.679 7.522 7.518 7.515 7.511 7.507 7.500 7.497 7.490 7.485 7.482 7.478 7.474 7.454 7.452 7.449 7.446 7.444 7.441 7.438 7.434 7.429 7.425 7.421 7.417 7.413 7.408 7.404 7.268 7.248 7.240 4.129 4.111 4.093 4.075 4.057
2.379
1.378 1.360 1.343
CDCl3
400 MHz
O
P
OEt
3c
2.00
1.83
3.09
2.25
2.02
2.79
2.92
11.5 11.0 10.5 10.0 9.5 9.0 8.5 8.0 7.5 7.0 6.5 6.0 5.5 5.0 4.5 4.0 3.5 3.0 2.5 2.0 1.5 1.0 0.5 0.0 –0
f1 (ppm)

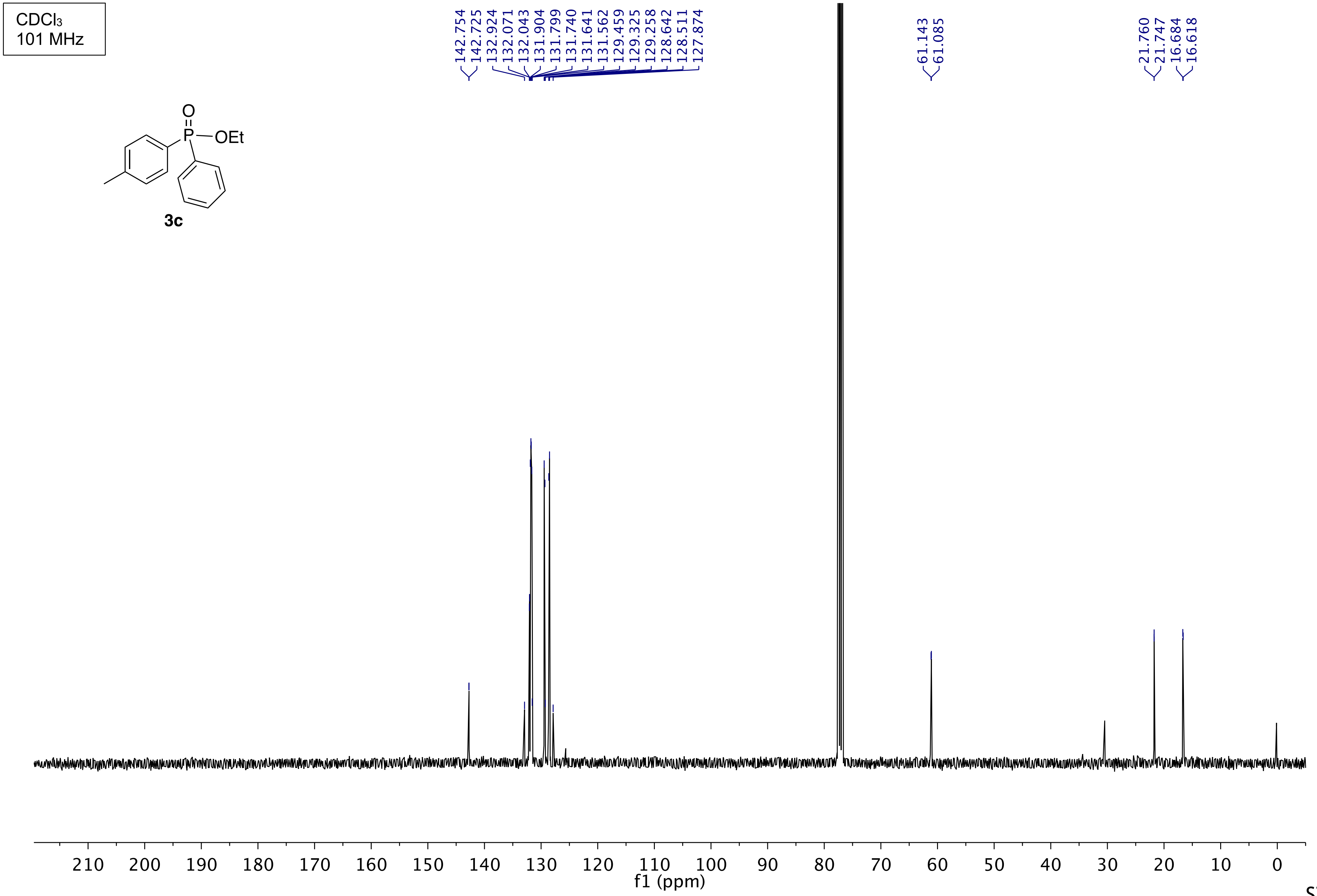
CDCl3
101 MHz
O
P
OEt
3c
142.754
142.725
132.924
132.071
132.043
131.904
131.799
131.740
131.641
131.562
129.459
129.325
129.258
128.642
128.511
127.874
61.143
61.085
21.760
21.747
16.684
16.618
210 200 190 180 170 160 150 140 130 120 110 100 90 80 70 60 50 40 30 20 10 0
f1 (ppm)

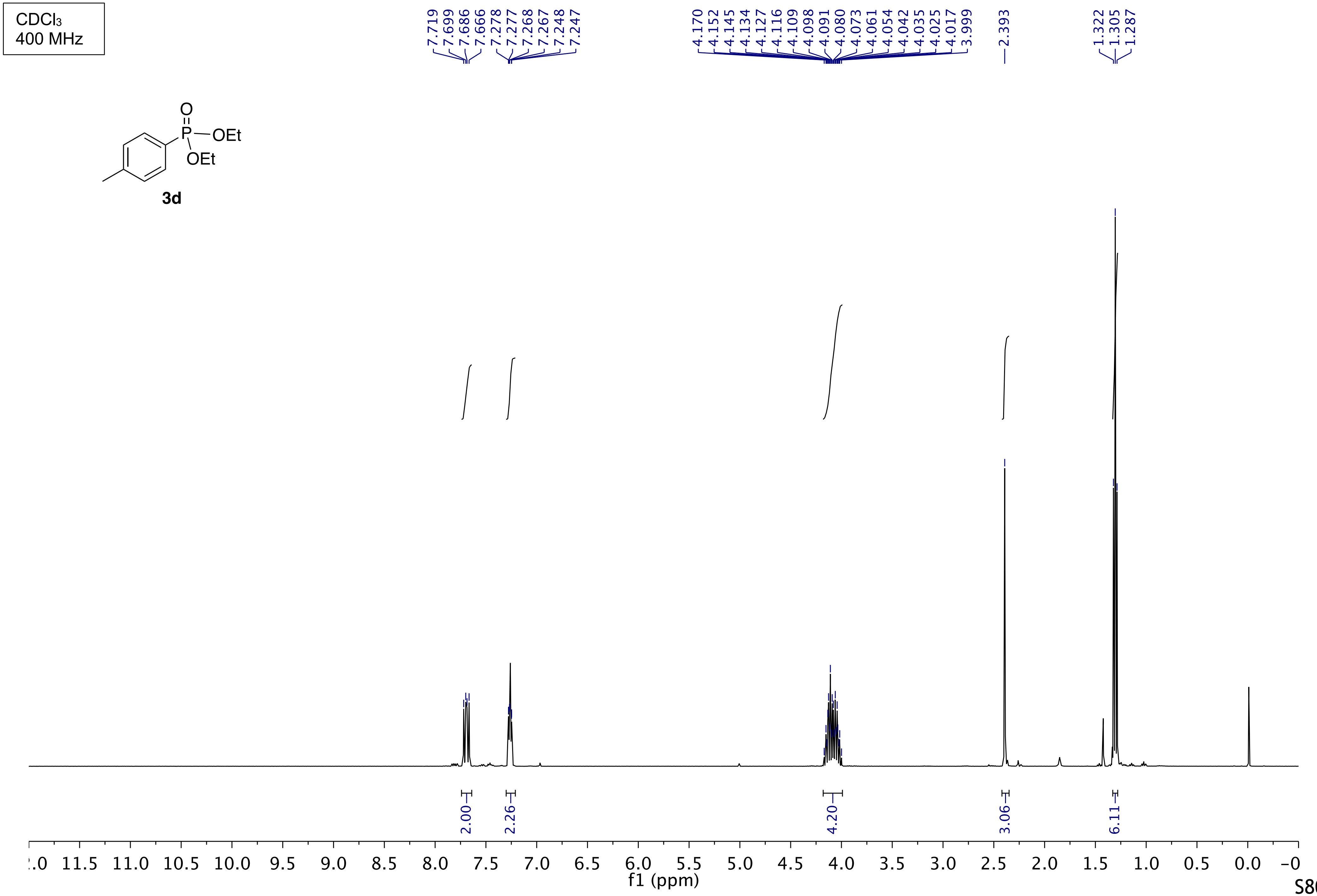
CDCl3
400 MHz
7.719
7.699
7.686
7.666
7.278
7.277
7.268
7.267
7.248
7.247
4.170
4.152
4.145
4.134
4.127
4.116
4.109
4.098
4.091
4.080
4.073
4.061
4.054
4.042
4.035
4.025
4.017
3.999
2.393
1.322
1.305
1.287
O
P
OEt
OEt
3d
2.00
2.26
4.20
3.06
6.11
11.5 11.0 10.5 10.0 9.5 9.0 8.5 8.0 7.5 7.0 6.5 6.0 5.5 5.0 4.5 4.0 3.5 3.0 2.5 2.0 1.5 1.0 0.5 0.0
f1 (ppm)

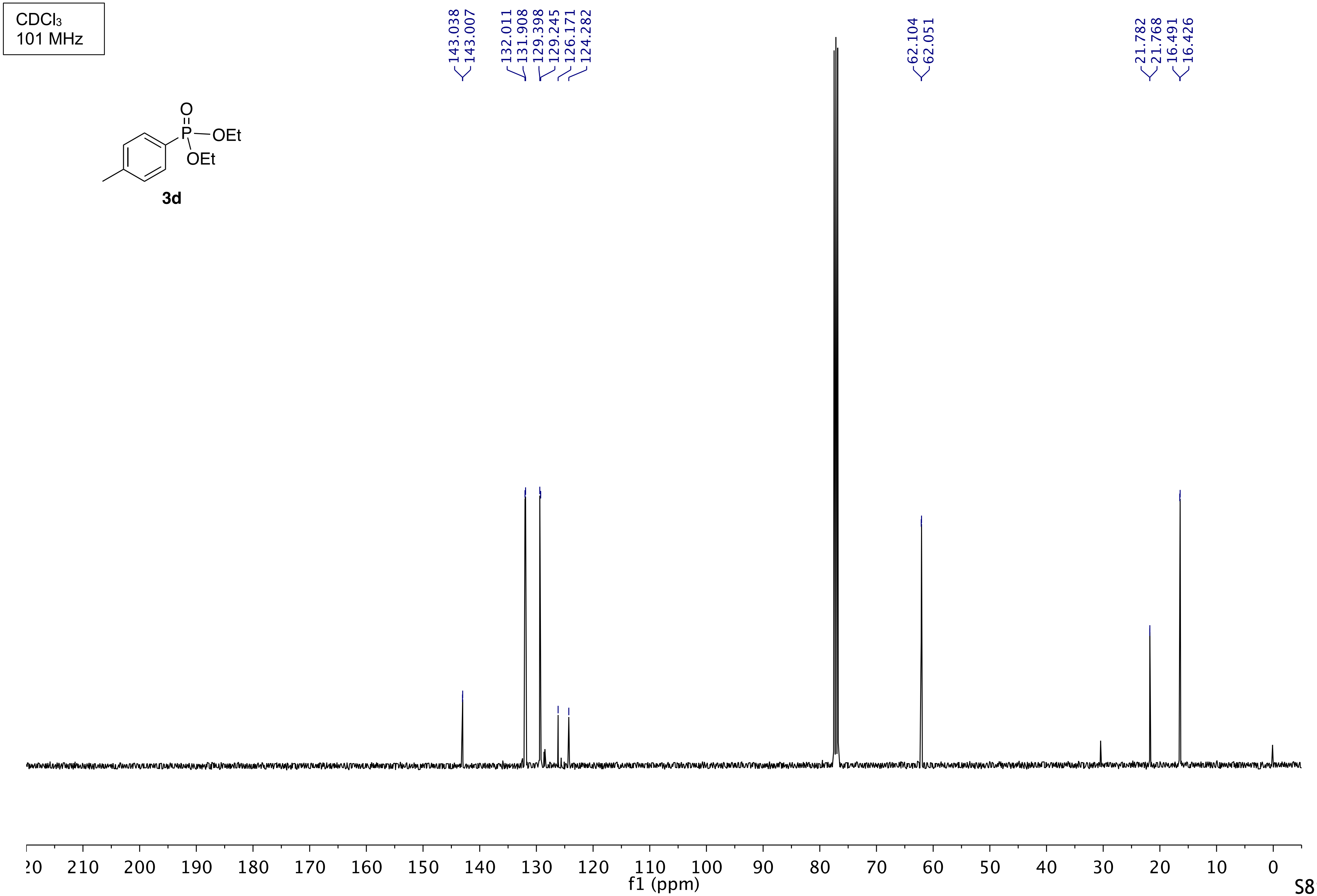
CDCl3
101 MHz
O
P
OEt
OEt
3d
143.038
143.007
132.011
131.908
129.398
129.245
126.171
124.282
62.104
62.051
21.782
21.768
16.491
16.426
f1 (ppm)

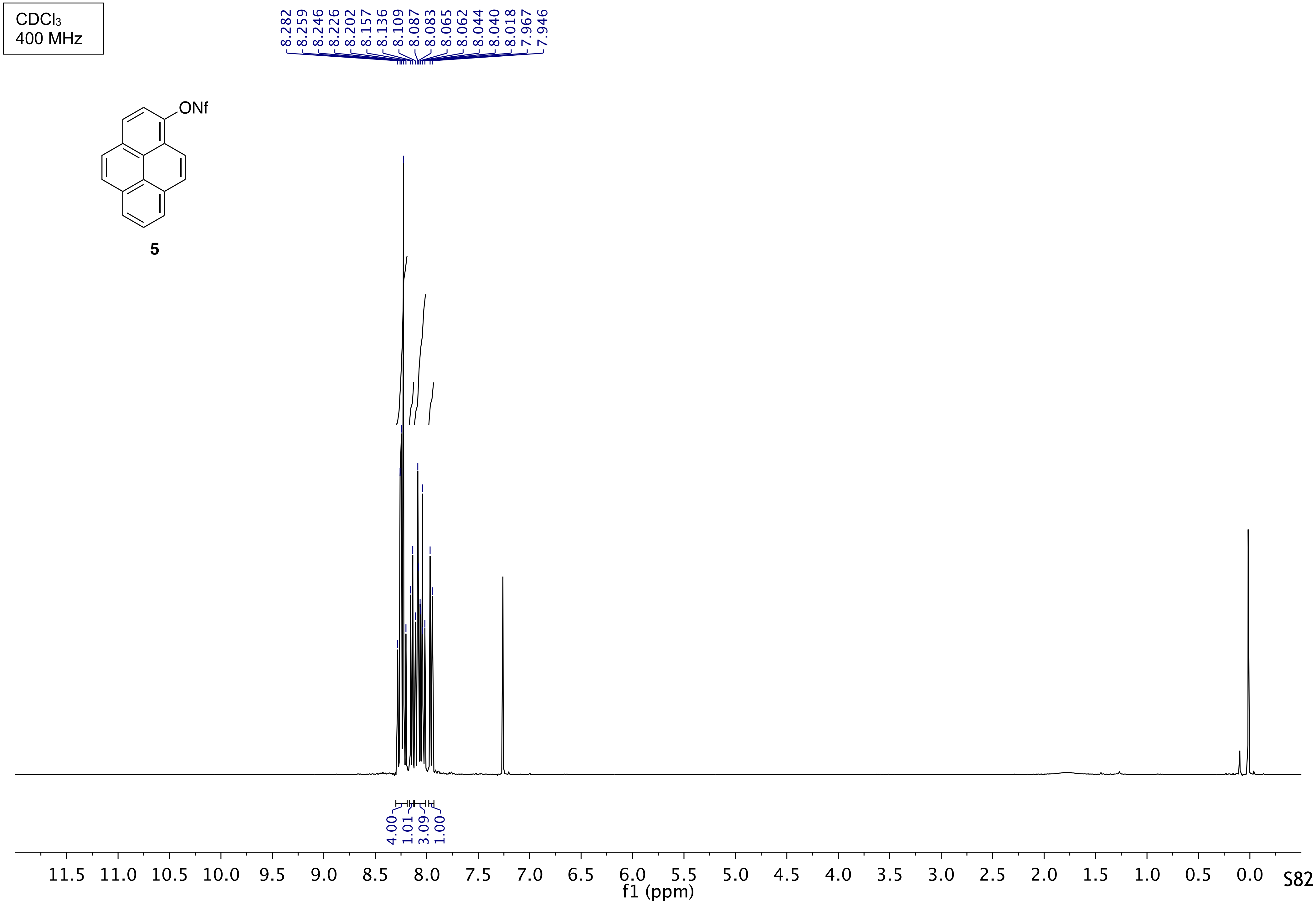
CDCl$_3$
400 MHz
ONf
5
8.282
8.259
8.246
8.226
8.202
8.157
8.136
8.109
8.087
8.083
8.065
8.062
8.044
8.040
8.018
7.967
7.946
4.00
1.01
3.09
1.00
11.5 11.0 10.5 10.0 9.5 9.0 8.5 8.0 7.5 7.0 6.5 6.0 5.5 5.0 4.5 4.0 3.5 3.0 2.5 2.0 1.5 1.0 0.5 0.0
f1 (ppm)

CDCl3
101 MHz

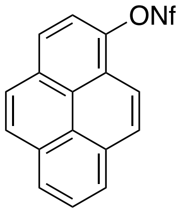

5

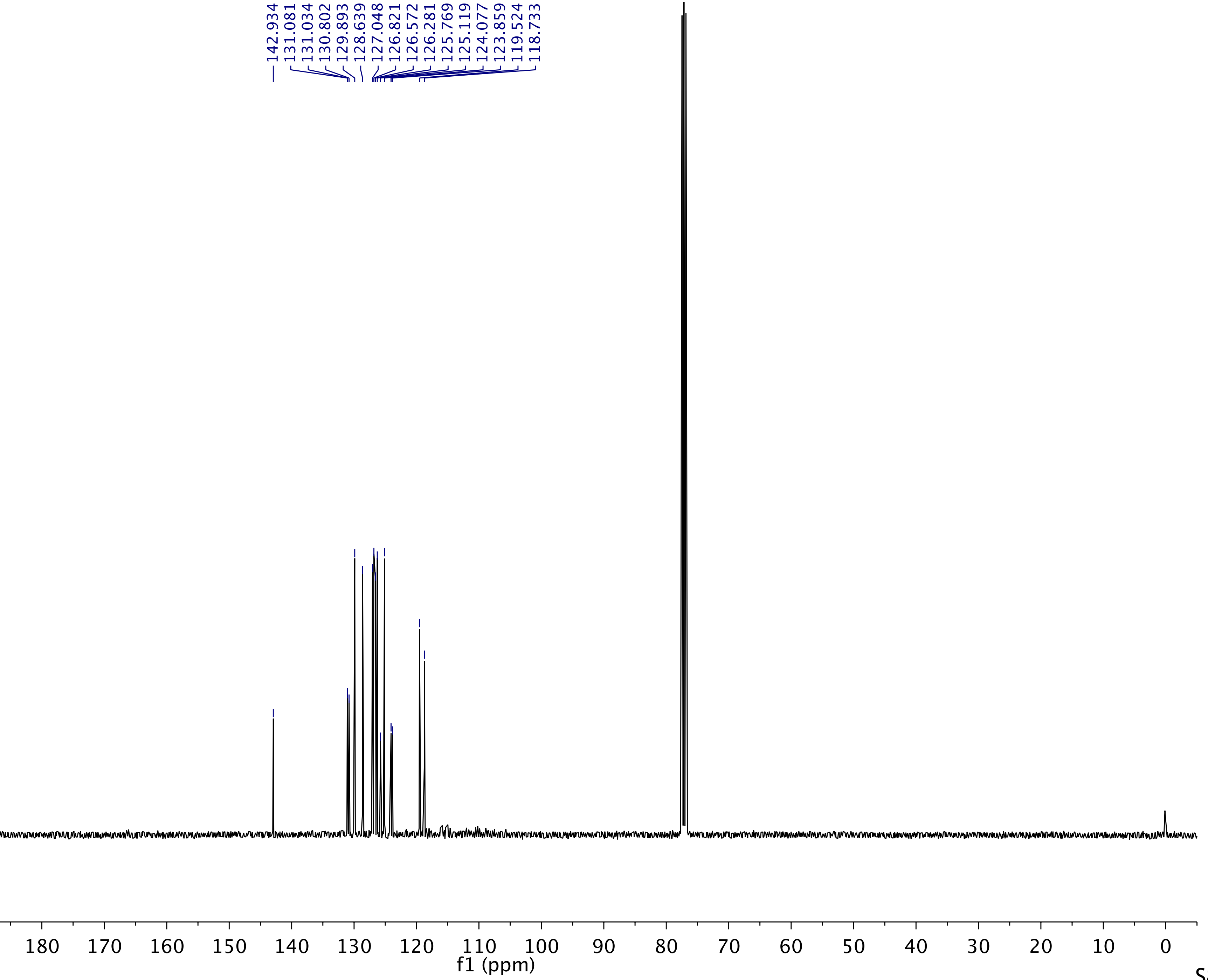

CDCl$_3$
400 MHz

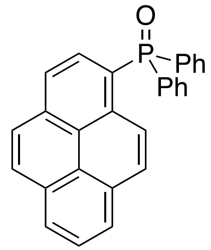

6

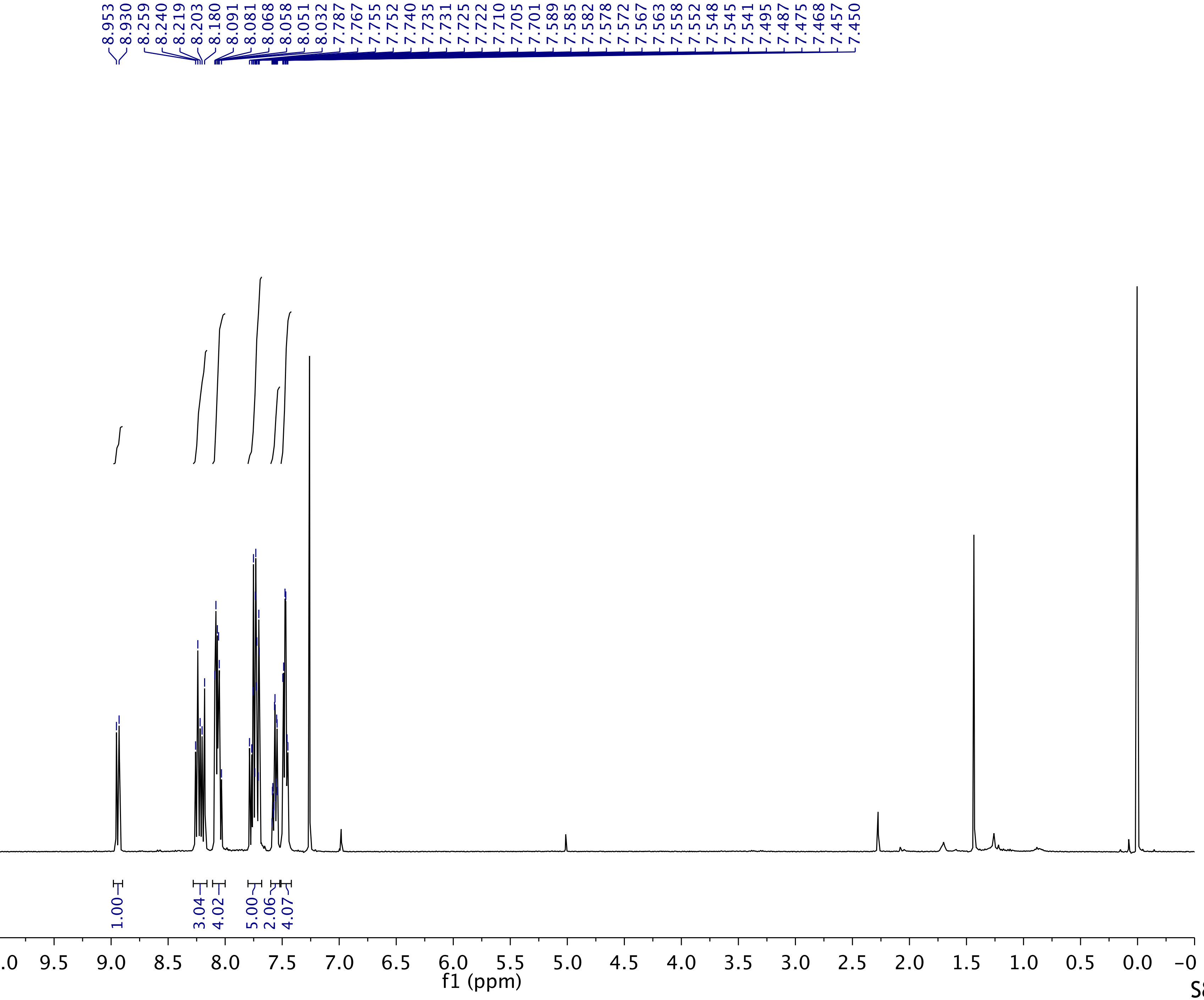

$CDCl_3$
101 MHz

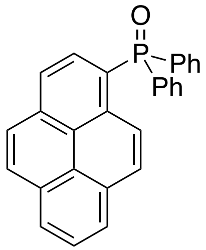

6

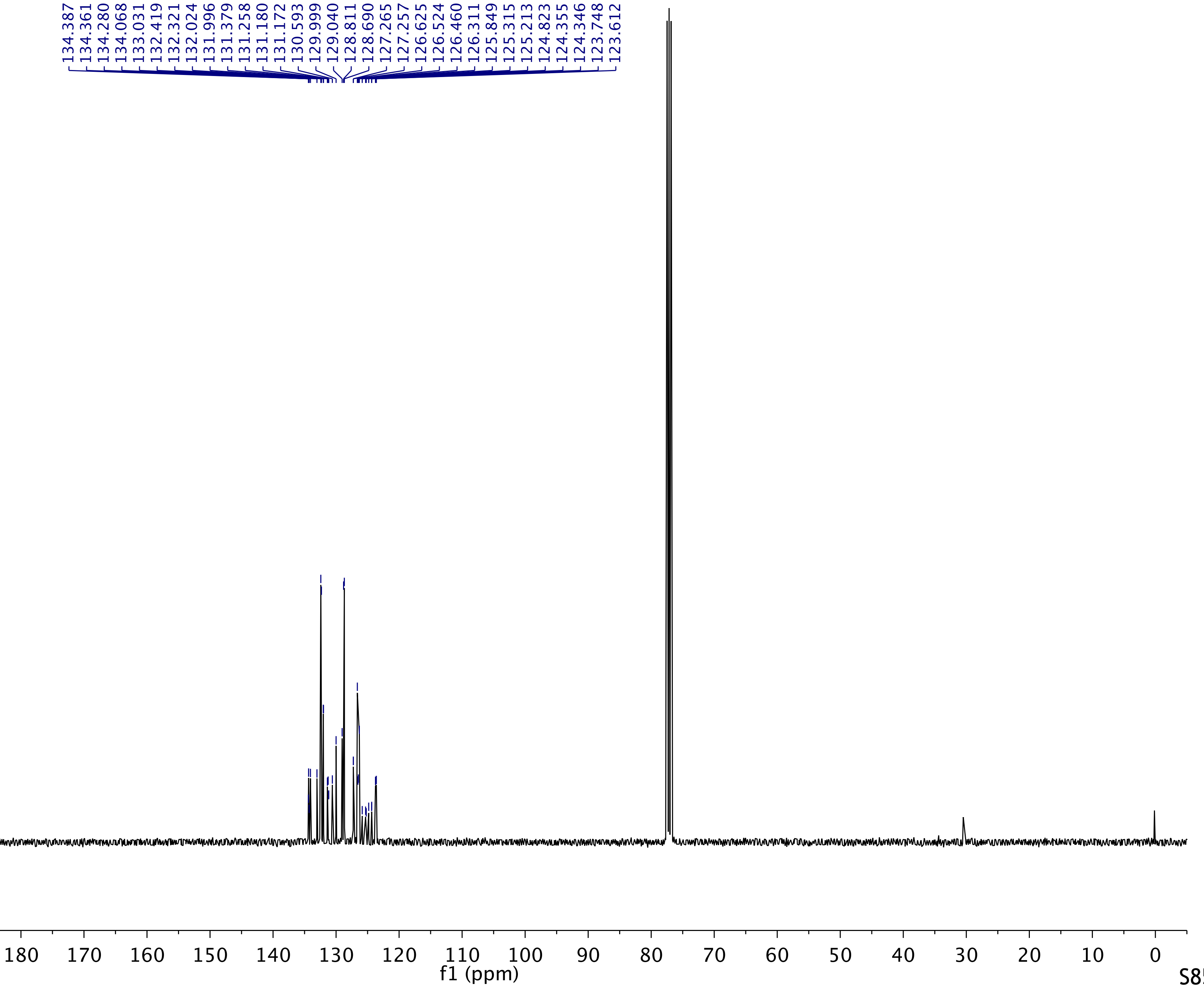

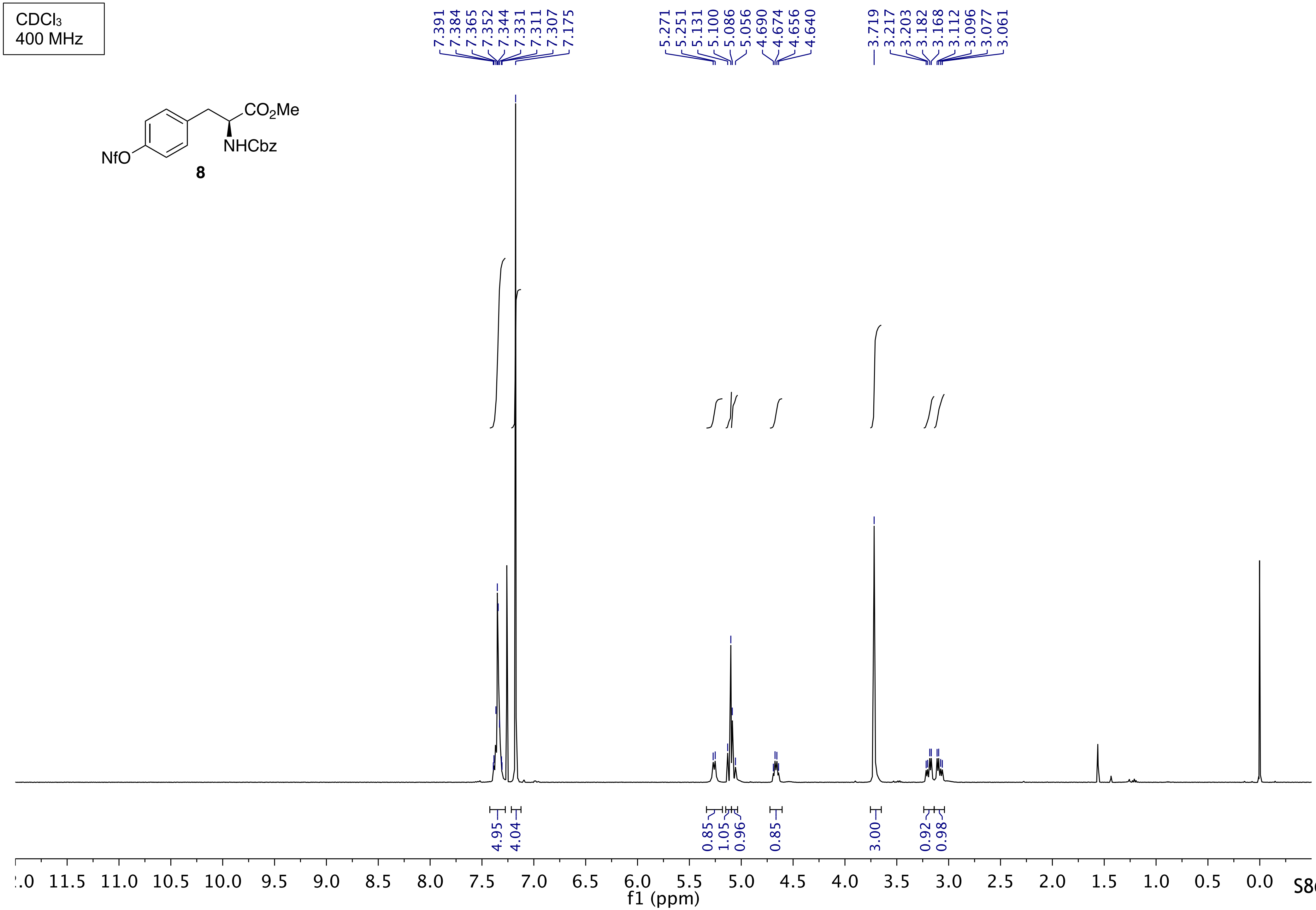
CDCl$_3$
400 MHz
CO$_2$Me
NHCbz
NfO
8
7.391
7.384
7.365
7.352
7.344
7.331
7.311
7.307
7.175
5.271
5.251
5.131
5.100
5.086
5.056
4.690
4.674
4.656
4.640
3.719
3.217
3.203
3.182
3.168
3.112
3.096
3.077
3.061
4.95
4.04
0.85
1.05
0.96
0.85
3.00
0.92
0.98
f1 (ppm)

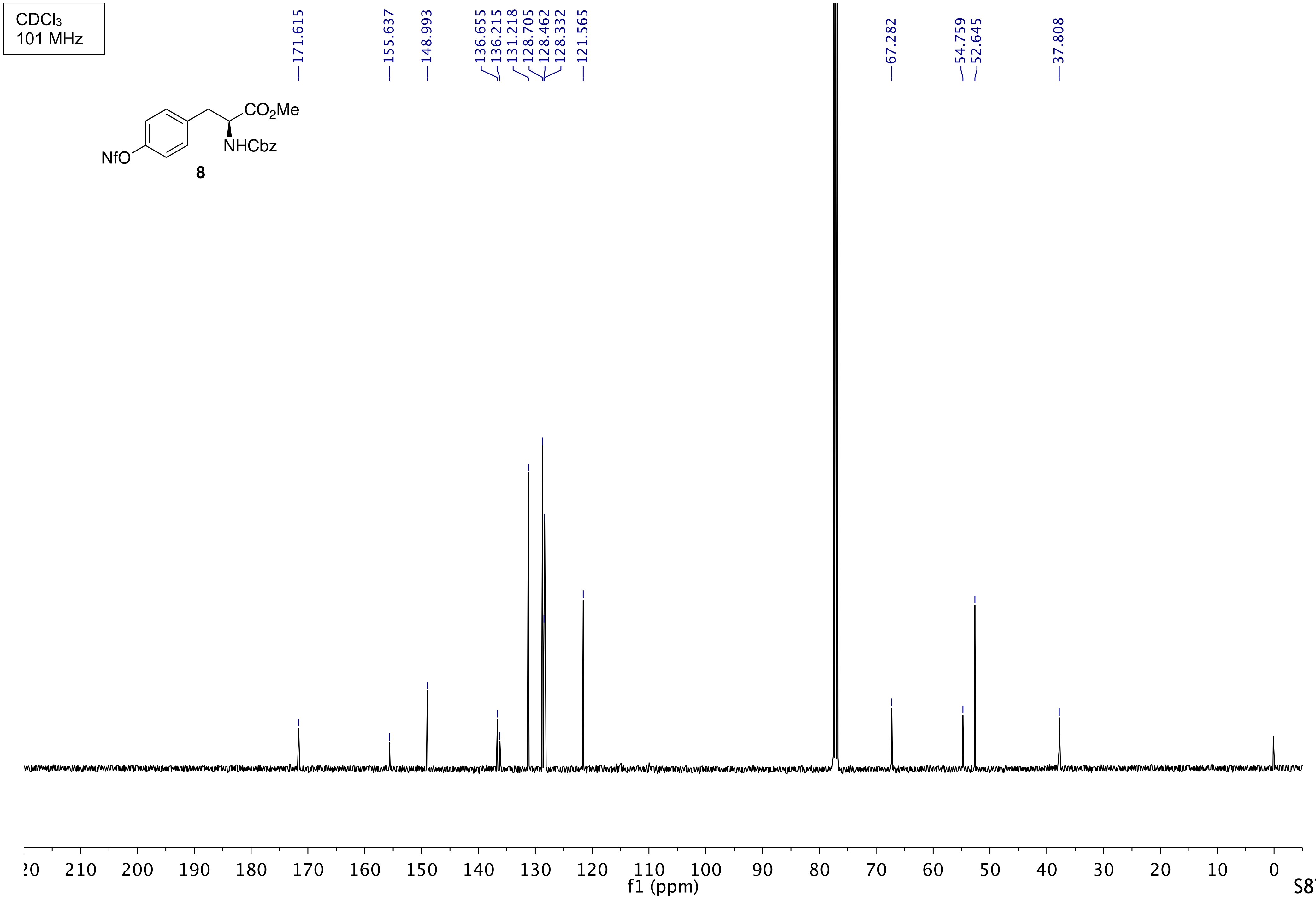

CDCl3
101 MHz
CO2Me
NHCbz
NfO
8
171.615
155.637
148.993
136.655
136.215
131.218
128.705
128.462
128.332
121.565
67.282
54.759
52.645
37.808
20 210 200 190 180 170 160 150 140 130 120 110 100 90 80 70 60 50 40 30 20 10 0
f1 (ppm)

CDCl$_3$
400 MHz

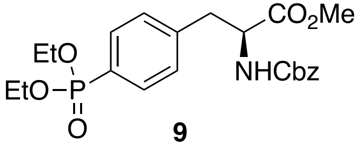

9

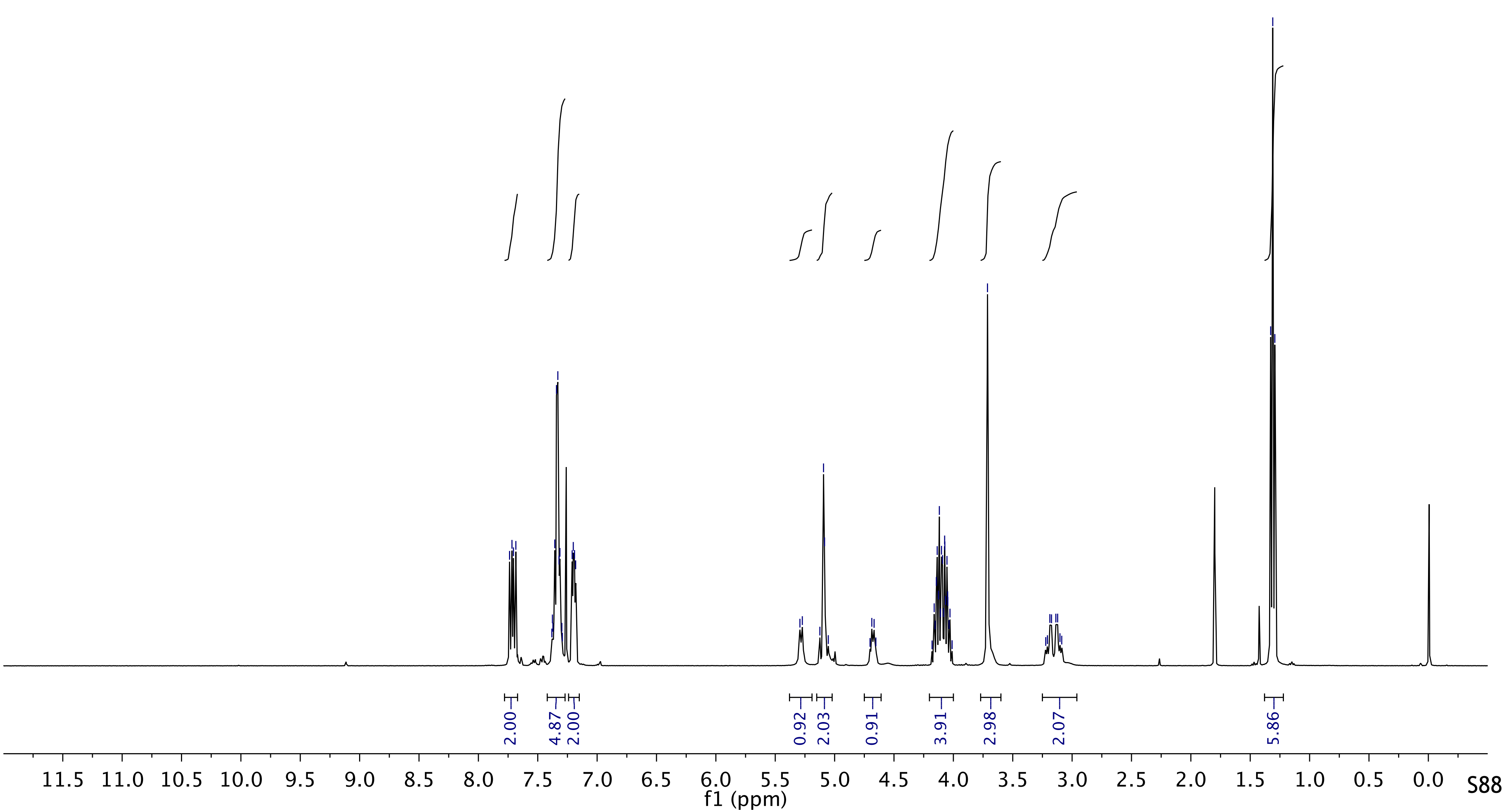

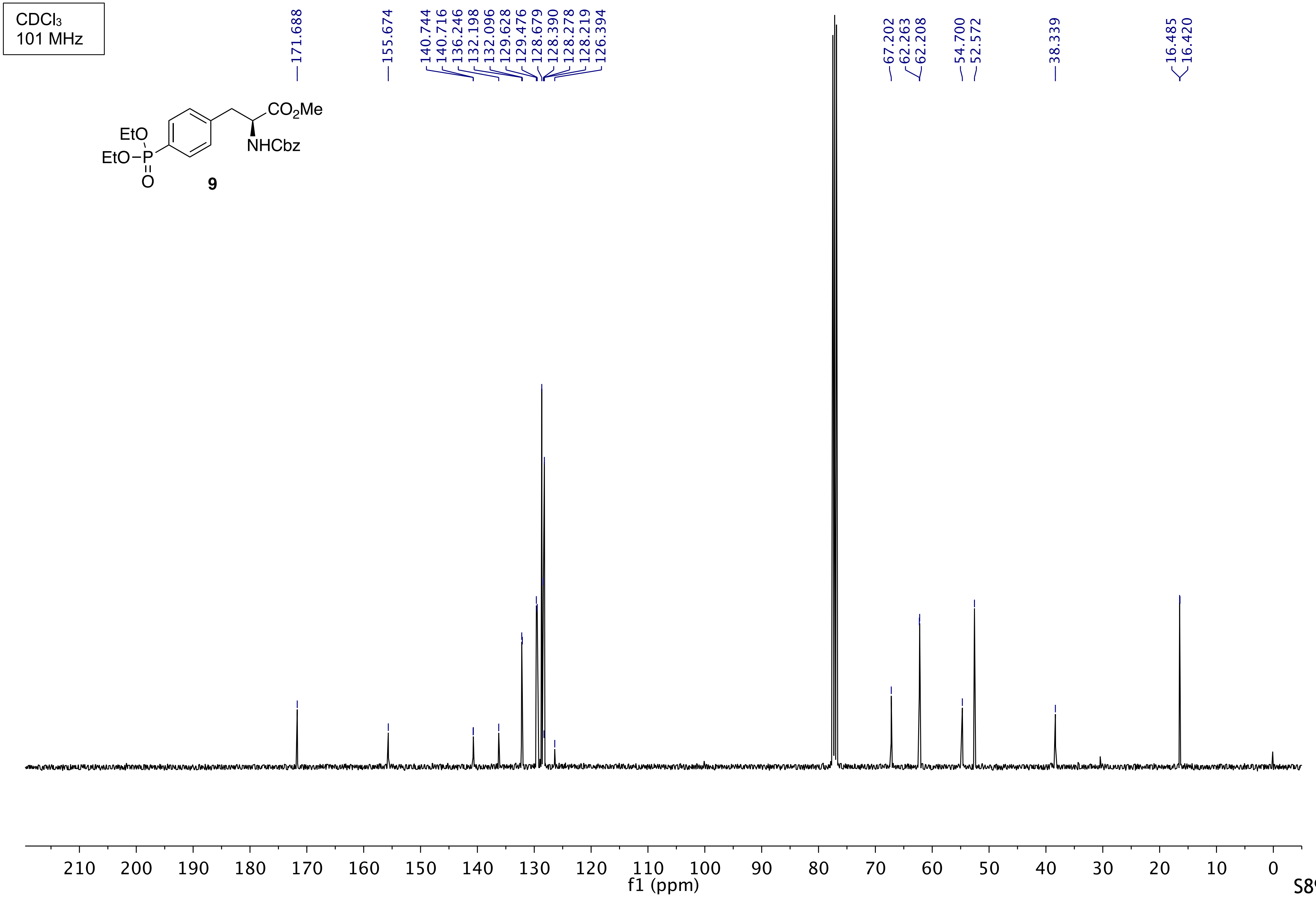
CDCl3
101 MHz
171.688
155.674
140.744
140.716
136.246
132.198
132.096
129.628
129.476
128.679
128.390
128.278
128.219
126.394
67.202
62.263
62.208
54.700
52.572
38.339
16.485
16.420
CO2Me
EtO
EtO–P
O
NHCbz
9
210 200 190 180 170 160 150 140 130 120 110 100 90 80 70 60 50 40 30 20 10 0
f1 (ppm)

D$_2$O
400 MHz

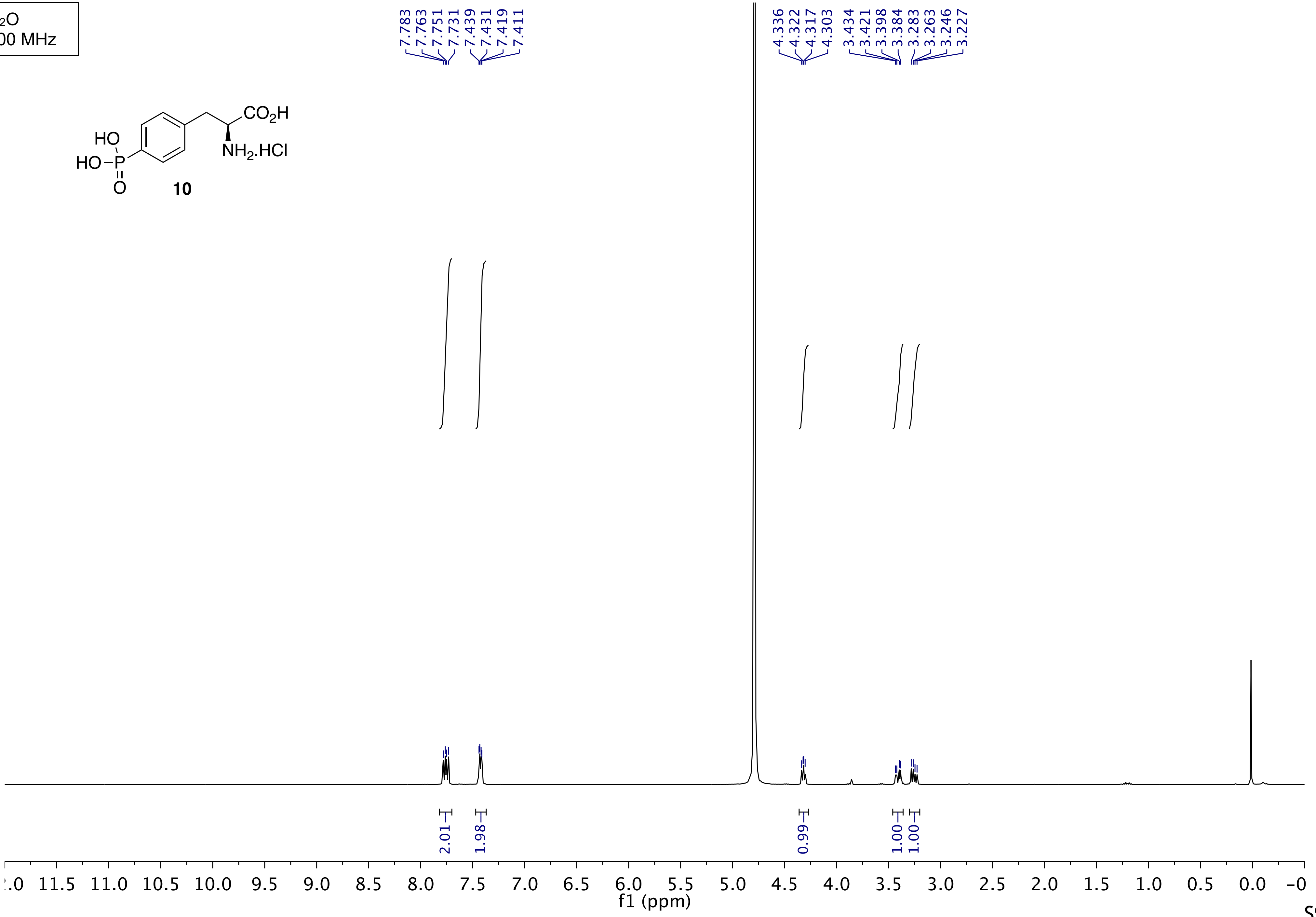

HO
HO–P
O
CO$_2$H
NH$_2$.HCl
10
7.783
7.763
7.751
7.731
7.439
7.431
7.419
7.411
4.336
4.322
4.317
4.303
3.434
3.421
3.398
3.384
3.283
3.263
3.246
3.227
2.01
1.98
0.99
1.00
1.00
12.0 11.5 11.0 10.5 10.0 9.5 9.0 8.5 8.0 7.5 7.0 6.5 6.0 5.5 5.0 4.5 4.0 3.5 3.0 2.5 2.0 1.5 1.0 0.5 0.0 -0
f1 (ppm)

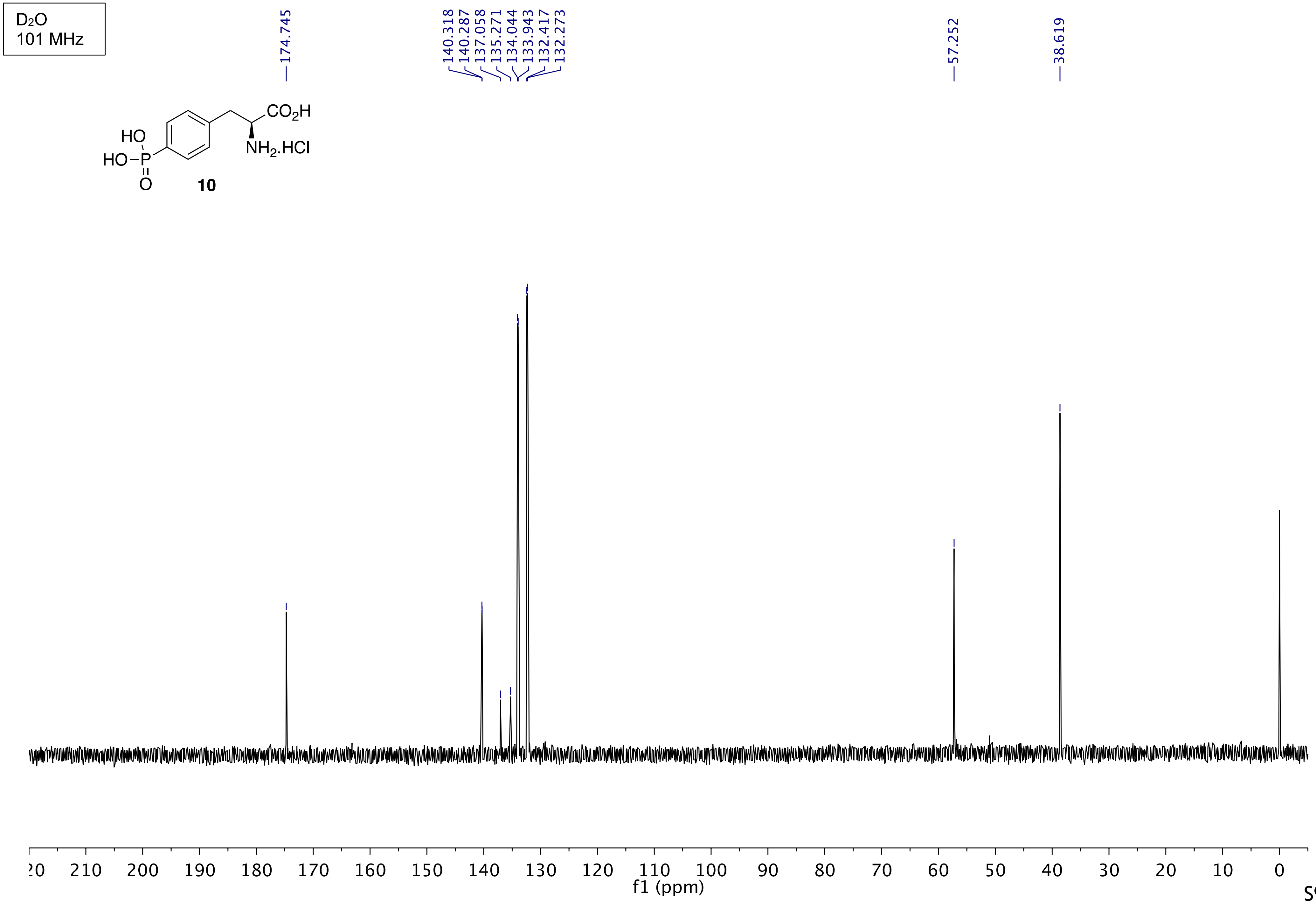

D₂O
101 MHz
174.745
140.318
140.287
137.058
135.271
134.044
133.943
132.417
132.273
57.252
38.619
HO
HO–P
O
CO₂H
NH₂.HCl
10
20 210 200 190 180 170 160 150 140 130 120 110 100 90 80 70 60 50 40 30 20 10 0
f1 (ppm)
